# Supplementary material for: Protocol for a systematic review on the methodological and reporting quality of prediction model studies using machine learning techniques
Source: BMJ Open. 2020 Nov 11;10(11):e038832. doi: 10.1136/bmjopen-2020-038832 (PMC7661369; doi:10.1136/bmjopen-2020-038832)
Supplement: Supplementary data [file bmjopen-2020-038832supp001.pdf]

Appendix 1

PubMed search strategy

Draft for the search strategy for systematic review of the reporting of the development and validation of machine learning models using Pubmed interface.

Search date: 19 November 2019

1. Machine Learning[MeSH Terms]
2. Deep learning[MeSH Terms]
3. supervised machine learning[MeSH Terms]
4. "Neural Networks, Computer"[Mesh]
5. data mining[MeSH Terms]
6. machine[tiab] AND (learn* OR model*)
7. (statistical[tiab] OR "statistical-learning"[tiab]) AND (strateg*[tiab])
8. multilayer perceptron*[tiab] OR random forest*[tiab] OR bayes* network*[tiab] OR support vector machine*[tiab] OR nearest neighbor*[tiab] OR k nearest neighbor*[tiab] OR elastic net[tiab] OR naive bayes*[tiab]
9. (classification[tiab] OR regression[tiab] OR estimation[tiab] OR decision[tiab]) AND tree[tiab]
10. ridge[tiab] OR kernel[tiab] OR ensemble[tiab] OR bagging[tiab] OR bagged[tiab] OR boosting[tiab] OR boosted[tiab] OR fuzzy[tiab]
11. #1 OR #2 OR #3 OR #4 OR #5 OR #6 OR #7 OR #8 OR #9 OR #10
12. (Validat* OR Predict* OR Rule*). [tiab]
13. (Predict* AND (Outcome* OR Risk* OR Model*). [tiab]
14. ((History OR Variable* OR Criteria OR Scor* OR Characteristic* OR Finding* OR Factor*) AND (Predict* OR Model* OR Decision* OR Identif* OR Prognos*)). [tiab]
15. (Decision* AND (Model* OR Clinical*). [tiab]
16. (Prognostic AND (History OR Variable* OR Criteria OR Scor* OR Characteristic* OR Finding* OR Factor* OR Model*). [tiab]
17. #12 OR #13 OR #14 OR #15 OR #16
18. (discrimination[tiab] OR discriminative[tiab] OR discriminatory[tiab]) AND (accuracy[tiab] OR ability[tiab] OR performance[tiab] OR value[tiab] OR model[tiab] OR models[tiab] OR power[tiab] OR capacity[tiab] OR capabilit*[tiab] OR efficiency[tiab])

19. (discriminability[tiab] OR c-index[tiab] OR c-statistic[tiab] OR concordance[tiab] OR DCA[tiab])
20. "decision curve"[tiab]
21. calibrat*[tiab] AND (plot*[tiab] OR curve*[tiab] OR slope*[tiab] OR model[tiab] OR models[tiab])
22. performance[tiab] AND (classification[tiab] OR classifier[tiab] OR clinical[tiab] OR accuracy[tiab] OR validation[tiab] OR metrics[tiab] OR diagnostic[tiab] OR AUC[tiab])
23. (sensitivity[tiab] OR specificity[tiab] OR PPV[tiab] OR NPV[tiab])
24. "correctly classified"[tiab]
25. "clinical accuracy"[tiab]
26. positive predictive value*[tiab]
27. negative predictive value*[tiab]
28. classification[tiab] OR classifier[tiab]
29. Area Under Curve[Mesh]
30. "Area under the curve"[tiab]
31. "Area under the ROC"[tiab]
32. “Area Under the Receiver”[tiab]
33. (ROC[tiab] OR AUC[tiab] OR AUROC[tiab])
34. ROC Curve [Mesh]
35. "Hosmer-Lemeshow"[tiab] OR "H-L test"[tiab]
36. "expected ratio"[tiab] OR "observed ratio"[tiab] OR "E:O ratio"[tiab]
37. #18 OR #19 OR #20 OR #21 OR #22 OR #23 OR #24 OR #25 OR #26 OR #27 OR #28 OR #29 OR #30 OR #31 OR #32 OR #33 OR #34 OR #35 OR #36

---

38. #11 AND #17
39. #11 AND (#17 OR #37)
40. #39 AND (“2018/01/01”[PDat]: “2019/12/31”[PDat])
41. #40 NOT “review”[pt]
42. #39 AND (“2019/01/01”[PDat]: “2019/12/31”[PDat])
43. #42 NOT “review”[pt]

Results #41= **24732**

Results #43=**12977**
