# Supplementary material for: Protocol for a systematic review on the methodological and reporting quality of prediction model studies using machine learning techniques
Source: BMJ Open. 2020 Nov 11;10(11):e038832. doi: 10.1136/bmjopen-2020-038832 (PMC7661369; doi:10.1136/bmjopen-2020-038832)
Supplement: Supplementary data [file bmjopen-2020-038832supp002.pdf]

Confidential

Article Information

Article ID

____

Article available for data extraction

Endnote number

____

Confidential

Background and objectives

Article ID	__________________________
The background and rationale are presented	◯ Yes<br>◯ No
Reference to existing models is included (or stated that there are no existing models)	◯ Yes<br>◯ No
Any description of why ML techniques are being used to address the objective is reported	◯ Yes<br>◯ No
If yes, please provide the statement below	__________________________
It is stated whether the study describes development and/or validation and/or incremental (added) value	◯ Yes<br>◯ No
Any additional comment about the "background and objectives" section of this article?	__________________________<br>(If there is something in the "Background" that does not fit into the questions of this form - please use this space to detail. Also use this space to detail anything you are unsure about.)

Andaur Navarro CL, *et al. BMJ Open* 2020; 10:e038832. doi: 10.1136/bmjopen-2020-038832

Confidential

Reviewer Information

Article ID ________________________________

Reviewer Information

Reviewer Name ________________________________
(Provide your initials)

Date of extraction ________________________________

Confidential

General Information

Article ID ______________________

General study information

Title ______________________

Journal ______________________

Publication year ______________________

Number of authors listed ______________________

Name of the first author ______________________
(Initials, Surname (e.g. EW Steyerberg))

What is the affiliation of the 1st author?
- ☐ Clinical
- ☐ Epidemiology
- ☐ Health informatics
- ☐ Data sciences
- ☐ Other

If other, please specify ______________________

What is the clinical area being investigated?
- ◯ Oncology
- ◯ Cardiovascular medicine
- ◯ Critical care
- ◯ Endocrinology
- ◯ Healthcare services
- ◯ Geriatric
- ◯ Hepatology
- ◯ Psychiatry
- ◯ Immunology
- ◯ Neonatology
- ◯ Nutrition
- ◯ Obstetrics & Gynaecology
- ◯ Physical medicine
- ◯ Primary care
- ◯ Surgery
- ◯ Infectious diseases
- ◯ Neurology
- ◯ Ophthalmology
- ◯ Pediatrics
- ◯ Nephrology
- ◯ Medical imaging
- ◯ Other

If other, please specify ______________________

Confidential

What is the type of study?	◯ Diagnosis<br>◯ Prognosis
What is the type of study?	◯ Classification<br>◯ Risk prediction<br>◯ Unclear<br>(We need to distinguish if the problem is a prediction problem or a classification problem. A classification problem is about predicting a label and a prediction problems is about predicting a quantity.)
What is the purpose of the article?	◯ Clinical use<br>◯ Simulation/tutoring<br>◯ Contest/Challenge<br>(If tutoring, please finish this form and don't follow with the extraction.)
What is the study design?	◯ Development only (including internal validation)<br>◯ Development with external validation (same model)<br>◯ Development with external validation (different model)<br>◯ External validation only
What is the primary outcome for the model?	__________________________________<br>(Please include timing of primary outcome. Extract on primary outcome only.)
What is the format of the primary outcome?	◯ Continuous<br>◯ Binary<br>◯ Ordinal<br>◯ Multinomial<br>◯ Time to event<br>◯ Count<br>◯ Other
If other, please specify	__________________________________
Any additional comment about the article?	____<br>(If there is information in "General Information" that does not fit into the questions of this form - please use this space to detail. Also use this space to detail anything you are unsure about.)

Andaur Navarro CL, *et al. BMJ Open* 2020; 10:e038832. doi: 10.1136/bmjopen-2020-038832

Confidential

Methods

Article ID	__________________________________
Is adherence to a reporting guideline mentioned?	◯ Yes<br>◯ No
If yes, to which guideline?	☐ TRIPOD<br>☐ CONSORT<br>☐ STROBE<br>☐ Other
If other, please specify	__________________________________
What type of study is reported?	◯ Development (including internal validation)<br>◯ Development with external validation (same model)<br>◯ Development with external validation (different model)<br>◯ External validation only<br>(This questions is repeated due to branching logic. Please answer again.)
What is the type of external validation?	☐ Temporal<br>☐ Geographical<br>☐ Independent data<br>☐ Fully independent<br>☐ Unclear<br>☐ Other
If other, please specify	__________________________________<br>(E.g. different setting, different participants population (pediatric/adult))
Differences or similarities in definitions with the development study are described	◯ Yes<br>◯ No<br>◯ NA<br>(Mentioning of any differences in all four (setting, eligibility criteria, predictors and outcome) is required to score Yes. If it is explicitly mentioned that there were no differences in setting, eligibility criteria, predictors and outcomes, score Yes. For incremental value reports, in case additional predictors are not added to a previously developed prediction model but rather added to conventional predictors in a newly fitted model, score Not applicable.)
In which domains are differences?	☐ Setting<br>☐ Eligibility criteria<br>☐ Predictors<br>☐ Outcomes<br>☐ No differences were reported<br>☐ Other

Confidential

If other, please specify	__________________ (list using (;) to separate if more than 1.)
Is there a diagram/draw to clarify the methods used?	◯ Yes<br>◯ No

Source of data

Development<br>The study design or source of data is reported	◯ Yes<br>◯ No<br>(E.g. Prospectively designed, existing cohort, existing RCT, registry/medical records, case control, case series. This needs to be explicitly reported; reference to this information in another article alone is insufficient.)
External validation<br>The study design or source of data is reported	◯ Yes<br>◯ No<br>(E.g. Prospectively designed, existing cohort, existing RCT, registry/medical records, case control, case series. This needs to be explicitly reported; reference to this information in another article alone is insufficient.)
External validation<br>The study design or source of data is reported	◯ Yes<br>◯ No<br>(E.g. Prospectively designed, existing cohort, existing RCT, registry/medical records, case control, case series. This needs to be explicitly reported; reference to this information in another article alone is insufficient.)
Development<br>If yes, what was the data source origin?	☐ RCT<br>☐ Prospective cohort<br>☐ Retrospective cohort<br>☐ Registry<br>☐ Electronic medical records<br>☐ Case-control/case-cohort study<br>☐ Individual patient data - meta analysis<br>☐ Claims<br>☐ Other<br>(Multiples answers are possible)
External validation<br>If yes, what was the data source origin?	☐ RCT<br>☐ Prospective cohort<br>☐ Retrospective cohort<br>☐ Registry<br>☐ Electronic medical records<br>☐ Case-control/case-cohort study<br>☐ Individual patient data - meta analysis<br>☐ Claims<br>☐ Other<br>(Multiples answers are possible)

Andaur Navarro CL, *et al. BMJ Open* 2020; 10:e038832. doi: 10.1136/bmjopen-2020-038832

Confidential

External validation<br>If yes, what was the data source origin?	☐ RCT<br>☐ Prospective cohort<br>☐ Retrospective cohort<br>☐ Registry<br>☐ Electronic medical records<br>☐ Case-control/case-cohort study<br>☐ Individual patient data - meta analysis<br>☐ Claims<br>☐ Other<br>(Multiples answers are possible)
If other, please specify	__________________________________
If other, please specify	__________________________________
If other, please specify	__________________________________
Development<br>The starting date of accrual is reported	◯ Yes<br>◯ No
External validation<br>The starting date of accrual is reported	◯ Yes<br>◯ No
External validation<br>The starting date of accrual is reported	◯ Yes<br>◯ No
If yes, what is the start data of data collection?	__________________________________
If yes, what is the start data of data collection?	__________________________________
If yes, what is the start data of data collection?	__________________________________
Development<br>The end date of accrual is reported	◯ Yes<br>◯ No
External validation<br>The end date of accrual is reported	◯ Yes<br>◯ No
External validation<br>The end date of accrual is reported	◯ Yes<br>◯ No
If yes, what is the end date of data collection?	__________________________________
If yes, what is the end date of data collection?	__________________________________
If yes, what is the end date of data collection?	__________________________________

Andaur Navarro CL, *et al. BMJ Open* 2020; 10:e038832. doi: 10.1136/bmjopen-2020-038832

Development<br>The length of follow-up is reported	◯ Yes<br>◯ No<br>◯ NA<br>(E.g. "Patients were followed from baseline for 10 years" and "10-year prediction of..."; notably for prognostic studies with long term follow-up. If this is not applicable for an article (i.e. diagnostic study or no follow-up), then score Not applicable.)
External validation<br>The length of follow-up is reported	◯ Yes<br>◯ No<br>◯ NA<br>(E.g. "Patients were followed from baseline for 10 years" and "10-year prediction of..."; notably for prognostic studies with long term follow-up. If this is not applicable for an article (i.e. diagnostic study or no follow-up), then score Not applicable.)
External validation<br>The length of follow-up is reported	◯ Yes<br>◯ No<br>◯ NA<br>(E.g. "Patients were followed from baseline for 10 years" and "10-year prediction of..."; notably for prognostic studies with long term follow-up. If this is not applicable for an article (i.e. diagnostic study or no follow-up), then score Not applicable.)
If yes, what is the length of follow up?	__________________________________
If yes, what is the length of follow up?	__________________________________
If yes, what is the length of follow up?	__________________________________
Development<br>The length of the prediction horizon/time frame is reported	◯ Yes<br>◯ No<br>◯ NA
External validation<br>The length of the prediction horizon/time frame is reported	◯ Yes<br>◯ No<br>◯ NA
External validation<br>The length of the prediction horizon/time frame is reported	◯ Yes<br>◯ No<br>◯ NA
If yes, what is the length of the prediction horizon/time frame?	__________________________________
If yes, what is the length of the prediction horizon/time frame?	__________________________________
If yes, what is the length of the prediction horizon/time frame?	__________________________________

Confidential

Participants

Development<br>The study setting is reported	◯ Yes<br>◯ No<br>(E.g.: 'surgery for endometrial cancer patients' is considered to be enough information about the study setting.)
External validation<br>The study setting is reported	◯ Yes<br>◯ No<br>(E.g.: 'surgery for endometrial cancer patients' is considered to be enough information about the study setting.)
External validation<br>The study setting is reported	◯ Yes<br>◯ No<br>(E.g.: 'surgery for endometrial cancer patients' is considered to be enough information about the study setting.)
Development<br>What is the setting for the model?	☐ Primary care<br>☐ Secondary care<br>☐ Tertiary care<br>☐ General population<br>☐ Other<br>(Primary care = GPs, dentists and pharmacists (often first point of care). Secondary care = hospital or clinic based care - can be planned (e.g., cataract operation) or emergency (e.g., fracture). Tertiary care = highly specialised treatments (e.g., transplant, hip replacement).)
External validation<br>What is the setting for the model?	☐ Primary care<br>☐ Secondary care<br>☐ Tertiary care<br>☐ General population<br>☐ Other<br>(Primary care = GPs, dentists and pharmacists (often first point of care). Secondary care = hospital or clinic based care - can be planned (e.g., cataract operation) or emergency (e.g., fracture). Tertiary care = highly specialised treatments (e.g., transplant, hip replacement).)
External validation<br>What is the setting for the model?	☐ Primary care<br>☐ Secondary care<br>☐ Tertiary care<br>☐ General population<br>☐ Other<br>(Primary care = GPs, dentists and pharmacists (often first point of care). Secondary care = hospital or clinic based care - can be planned (e.g., cataract operation) or emergency (e.g., fracture). Tertiary care = highly specialised treatments (e.g., transplant, hip replacement).)
If other, please specify	__________________________________
If other, please specify	__________________________________

Andaur Navarro CL, *et al. BMJ Open* 2020; 10:e038832. doi: 10.1136/bmjopen-2020-038832

If other, please specify	__________________________________
Development<br>The number of centres involved is reported	◯ Yes<br>◯ No<br>(If the number is not reported explicitly, but can be concluded from the name of the centre/centres, or if clearly a single centre study, score Yes.)
External validation<br>The number of centres involved is reported	◯ Yes<br>◯ No<br>(If the number is not reported explicitly, but can be concluded from the name of the centre/centres, or if clearly a single centre study, score Yes.)
External validation<br>The number of centres involved is reported	◯ Yes<br>◯ No<br>(If the number is not reported explicitly, but can be concluded from the name of the centre/centres, or if clearly a single centre study, score Yes.)
How many centres involved?	__________________________________
How many centres involved?	__________________________________
How many centres involved?	__________________________________
Development<br>The geographical location (at least country) of centres involved is reported	◯ Yes<br>◯ No<br>(If no geographical location is specified, but the location can be concluded from the name of the centre(s), score Yes.)
External validation<br>The geographical location (at least country) of centres involved is reported	◯ Yes<br>◯ No<br>(If no geographical location is specified, but the location can be concluded from the name of the centre(s), score Yes.)
External validation<br>The geographical location (at least country) of centres involved is reported	◯ Yes<br>◯ No<br>(If no geographical location is specified, but the location can be concluded from the name of the centre(s), score Yes.)
If yes, what was the geographic location of the data collection?	☐ Europe<br>☐ North America<br>☐ Latin America<br>☐ Asia<br>☐ Africa<br>☐ Oceania<br>(Multiples answers are possible)

If yes, what was the geographic location of the data collection?	☐ Europe<br>☐ North America<br>☐ Latin America<br>☐ Asia<br>☐ Africa<br>☐ Oceania<br>(Multiples answers are possible)
If yes, what was the geographic location of the data collection?	☐ Europe<br>☐ North America<br>☐ Latin America<br>☐ Asia<br>☐ Africa<br>☐ Oceania<br>(Multiples answers are possible)

Eligibility criteria

Development<br>In-/exclusion criteria are stated	◯ Yes<br>◯ No<br>(These should explicitly be stated. Reasons for exclusion only described in a participant flow is not sufficient.)
External validation<br>In-/exclusion criteria are stated	◯ Yes<br>◯ No<br>(These should explicitly be stated. Reasons for exclusion only described in a participant flow is not sufficient.)
External validation<br>In-/exclusion criteria are stated	◯ Yes<br>◯ No<br>(These should explicitly be stated. Reasons for exclusion only described in a participant flow is not sufficient.)
Development<br>What was the participant population?	________________________________
External validation<br>What was the participant population?	________________________________
External validation<br>What was the participant population?	________________________________
Development<br>Details of any treatments received are described	◯ Yes<br>◯ No<br>◯ NA<br>(This item is notably for prognostic modelling studies and is about treatment at baseline or during follow-up. The 'if relevant' judgment of treatment requires clinical knowledge and interpretation. If you are certain that treatment was not relevant, e.g. in some diagnostic model studies, score Not applicable)

External validation<br>Details of any treatments received are described	◯ Yes<br>◯ No<br>◯ NA<br>(This item is notably for prognostic modelling studies and is about treatment at baseline or during follow-up. The 'if relevant' judgment of treatment requires clinical knowledge and interpretation. If you are certain that treatment was not relevant, e.g. in some diagnostic model studies, score Not applicable)
External validation<br>Details of any treatments received are described	◯ Yes<br>◯ No<br>◯ NA<br>(This item is notably for prognostic modelling studies and is about treatment at baseline or during follow-up. The 'if relevant' judgment of treatment requires clinical knowledge and interpretation. If you are certain that treatment was not relevant, e.g. in some diagnostic model studies, score Not applicable)

Outcome

Development<br>The outcome definition is clearly presented	◯ Yes<br>◯ No
External validation<br>The outcome definition is clearly presented	◯ Yes<br>◯ No
External validation<br>The outcome definition is clearly presented	◯ Yes<br>◯ No
Development<br>What is the type of primary outcome?	◯ Death<br>◯ Complications<br>◯ Recurrence<br>◯ Survival<br>◯ Other
External validation<br>What is the type of primary outcome?	◯ Death<br>◯ Complications<br>◯ Recurrence<br>◯ Survival<br>◯ Other
External validation<br>What is the type of primary outcome?	◯ Death<br>◯ Complications<br>◯ Recurrence<br>◯ Survival<br>◯ Other
If other, please specify	__________________________________
If other, please specify	__________________________________
If other, please specify	__________________________________

Development<br>It is described how outcome was assessed	◯ Yes<br>◯ No<br>(Including all elements of any composite, for example CVD [e.g. MI, HF, stroke])
External validation<br>It is described how outcome was assessed	◯ Yes<br>◯ No<br>(Including all elements of any composite, for example CVD [e.g. MI, HF, stroke])
External validation<br>It is described how outcome was assessed	◯ Yes<br>◯ No<br>(Including all elements of any composite, for example CVD [e.g. MI, HF, stroke])
Development<br>It is described when the outcome was assessed (time point(s) since T0)	◯ Yes<br>◯ No
External validation<br>It is described when the outcome was assessed (time point(s) since T0)	◯ Yes<br>◯ No
External validation<br>It is described when the outcome was assessed (time point(s) since T0)	◯ Yes<br>◯ No
Development<br>Actions to blind assessment of outcome to be predicted are reported	◯ Yes<br>◯ No<br>(If it is clearly a non-issue (e.g. all-cause mortality or an outcome not requiring interpretation), score Yes. In all other instances, an explicit mention is expected.)
External validation<br>Actions to blind assessment of outcome to be predicted are reported	◯ Yes<br>◯ No<br>(If it is clearly a non-issue (e.g. all-cause mortality or an outcome not requiring interpretation), score Yes. In all other instances, an explicit mention is expected.)
External validation<br>Actions to blind assessment of outcome to be predicted are reported	◯ Yes<br>◯ No<br>(If it is clearly a non-issue (e.g. all-cause mortality or an outcome not requiring interpretation), score Yes. In all other instances, an explicit mention is expected.)

**Predictors**	
Development<br>All predictors are reported	◯ Yes<br>◯ No<br>(For development, "all predictors" refers to all predictors that potentially could have been included in the 'final' model (including those considered in any univariable analyses).)
External validation<br>All predictors are reported	◯ Yes<br>◯ No<br>(For validation, "all predictors" means the predictors in the model being evaluated.)
External validation<br>All predictors are reported	◯ Yes<br>◯ No<br>(For validation, "all predictors" means the predictors in the model being evaluated.)
Development<br>Number of candidate predictors considered	______________________________<br>(If the number of candidate predictors is unclear, please fill this question with "UN")
External validation<br>External validation<br>Number of candidate predictors considered	______________________________<br>(If the number of candidate predictors is unclear, please fill this question with 'UN')
Development<br>What are the categories of the candidate predictors?	☐ Demography<br>☐ Clinical history<br>☐ Physical examination<br>☐ Blood and Urine parameters<br>☐ Imaging<br>☐ Genetic Risk Score<br>☐ Pathology<br>☐ Scale Score (e.g. pain, wellbeing, QoL)<br>☐ Questionnaires<br>☐ Other<br>(Multiples answers are possible)
External validation<br>External validation<br>What are the categories of the candidate predictors?	☐ Demography<br>☐ Clinical history<br>☐ Physical examination<br>☐ Blood and Urine parameters<br>☐ Imaging<br>☐ Genetic Risk Score<br>☐ Pathology<br>☐ Scale Score (e.g. pain, wellbeing, QoL)<br>☐ Questionnaires<br>☐ Other<br>(Multiples answers are possible)
If other, please specify	______________________________<br>(list using (;) to separate if more than 1)
If other, please specify	______________________________<br>(list using (;) to separate if more than 1)

Andaur Navarro CL, *et al. BMJ Open* 2020; 10:e038832. doi: 10.1136/bmjopen-2020-038832

Development<br>Were a-priori predictors considered/forced into the model?	◯ Yes<br>◯ No<br>◯ Unclear<br>(E.g. Clinical reasoning, literature review, money constraints)
Development<br>Predictor definitions are clearly presented	◯ Yes<br>◯ No
External validation<br>Predictor definitions are clearly presented	◯ Yes<br>◯ No
External validation<br>Predictor definitions are clearly presented	◯ Yes<br>◯ No
Development<br>It is clearly described how the predictors were measured	◯ Yes<br>◯ No
External validation<br>It is clearly described how the predictors were measured	◯ Yes<br>◯ No
External validation<br>It is clearly described how the predictors were measured	◯ Yes<br>◯ No
Development<br>It is clearly described when the predictors were measured	◯ Yes<br>◯ No
External validation<br>It is clearly described when the predictors were measured	◯ Yes<br>◯ No
External validation<br>It is clearly described when the predictors were measured	◯ Yes<br>◯ No
Development<br>It is clearly described whether predictor assessments were blinded for outcome	◯ Yes<br>◯ No<br>(For predictors for which it is clearly a non-issue (e.g. automatic blood pressure measurement, age, sex) and for instances where the predictors were clearly assessed before outcome assessment, score Yes. For all other predictors an explicit mention is expected.)
External validation<br>It is clearly described whether predictor assessments were blinded for outcome	◯ Yes<br>◯ No<br>(For predictors for which it is clearly a non-issue (e.g. automatic blood pressure measurement, age, sex) and for instances where the predictors were clearly assessed before outcome assessment, score Yes. For all other predictors an explicit mention is expected.)

Andaur Navarro CL, *et al. BMJ Open* 2020; 10:e038832. doi: 10.1136/bmjopen-2020-038832

External validation<br>It is clearly described whether predictor assessments were blinded for outcome	◯ Yes<br>◯ No<br>(For predictors for which it is clearly a non-issue (e.g. automatic blood pressure measurement, age, sex) and for instances where the predictors were clearly assessed before outcome assessment, score Yes. For all other predictors an explicit mention is expected.)
Development<br>It is clearly described whether predictor assessments were blinded for the other predictors	◯ Yes<br>◯ No
External validation<br>It is clearly described whether predictor assessments were blinded for the other predictors	◯ Yes<br>◯ No
External validation<br>It is clearly described whether predictor assessments were blinded for the other predictors	◯ Yes<br>◯ No

Sample Size

Development<br>It is explained how the sample size was arrived at	◯ Yes<br>◯ No<br>(Is there any mention of sample size, e.g. whether this was done on statistical grounds or practical/logistical grounds (e.g. an existing study cohort or data set of a RCT was used)?)
External validation<br>It is explained how the sample size was arrived at	◯ Yes<br>◯ No<br>(Is there any mention of sample size, e.g. whether this was done on statistical grounds or practical/logistical grounds (e.g. an existing study cohort or data set of a RCT was used)?)
External validation<br>It is explained how the sample size was arrived at	◯ Yes<br>◯ No<br>(Is there any mention of sample size, e.g. whether this was done on statistical grounds or practical/logistical grounds (e.g. an existing study cohort or data set of a RCT was used)?)
Development<br>What is the reason for the sample?	◯ Power<br>◯ Justified time interval<br>◯ Size of existing/available data<br>◯ Events per variable<br>◯ Other
External validation<br>What is the reason for the sample?	◯ Power<br>◯ Justified time interval<br>◯ Size of existing/available data<br>◯ Events per variable<br>◯ Other

Confidential

External validation<br>What is the reason for the sample?	◯ Power<br>◯ Justified time interval<br>◯ Size of existing/available data<br>◯ Events per variable<br>◯ Other
Development<br>If other, please specify	__________________________________
External validation<br>If other, please specify	__________________________________
External validation<br>If other, please specify	__________________________________
Development<br>What was the initial sample size of the study?	__________________________________<br>(If unclear, please fill this with "UN")
Development<br>What was the final sample size of the study?	__________________________________<br>(If unclear, please fill this with "UN")
External validation<br>What was the initial sample size of the study?	__________________________________<br>(If unclear, please fill this with "UN")
External validation<br>What was the final sample size of the study?	__________________________________<br>(If unclear, please fill this with "UN")
External validation<br>What was the initial sample size of the study?	__________________________________<br>(If unclear, please fill this with "UN")
External validation<br>What was the final sample size of the study?	__________________________________<br>(If unclear, please fill this with "UN")

Missing Data

Development<br>Was missingness an explicit exclusion criterion for the data?	◯ Yes<br>◯ No<br>◯ Unclear
External validation<br>Was missingness an explicit exclusion criterion for the data?	◯ Yes<br>◯ No<br>◯ Unclear
External validation<br>Was missingness an explicit exclusion criterion for the data?	◯ Yes<br>◯ No<br>◯ Unclear
Development<br>If yes, how many patients were excluded due to missing data?	__________________________________<br>(If not reported, please fill this with "NR")

Confidential

External validation<br>If yes, how many patients were excluded due to missing data?	________________________________<br>(If not reported, please fill this with "NR")
External validation<br>If yes, how many patients were excluded due to missing data?	________________________________<br>(If not reported, fill this with "NR")
Development<br>The method for handling missing data (predictors and outcome) is mentioned	◯ Yes<br>◯ No<br>(E.g. Complete case (explicit mention that individuals with missing values have been excluded), single imputation, multiple imputation, mean/median imputation. If there is no missing data, there should be an explicit mention that there is no missing data for all predictors and outcome. If so, score Yes. If it is unclear whether there is missing data (from e.g. the reported methods or results), score No. If it is clear there is missing data, but the method for handling missing data is unclear, score No.)
External validation<br>The method for handling missing data (predictors and outcome) is mentioned	◯ Yes<br>◯ No<br>(E.g. Complete case (explicit mention that individuals with missing values have been excluded), single imputation, multiple imputation, mean/median imputation. If there is no missing data, there should be an explicit mention that there is no missing data for all predictors and outcome. If so, score Yes. If it is unclear whether there is missing data (from e.g. the reported methods or results), score No. If it is clear there is missing data, but the method for handling missing data is unclear, score No.)
External validation<br>The method for handling missing data (predictors and outcome) is mentioned	◯ Yes<br>◯ No<br>(E.g. Complete case (explicit mention that individuals with missing values have been excluded), single imputation, multiple imputation, mean/median imputation. If there is no missing data, there should be an explicit mention that there is no missing data for all predictors and outcome. If so, score Yes. If it is unclear whether there is missing data (from e.g. the reported methods or results), score No. If it is clear there is missing data, but the method for handling missing data is unclear, score No.)

Confidential

Development<br>In presence of missing data, how was this dealt with?	☐ No missing data<br>☐ No imputation<br>☐ Multiple Imputation<br>☐ Complete case analysis<br>☐ Mean imputation<br>☐ Median imputation<br>☐ Other<br>(If there is any discrepancy between how missing values were handled for outcome and predictors, please specify so it in the comments below the methods form.)
External validation<br>In presence of missing data, how was this dealt with?	☐ No missing data<br>☐ No imputation<br>☐ Multiple Imputation<br>☐ Complete case analysis<br>☐ Mean imputation<br>☐ Median imputation<br>☐ Other<br>(If there is any discrepancy between how missing values were handled for outcome and predictors, please specify so it in the comments below the methods form.)
External validation<br>In presence of missing data, how was this dealt with?	☐ No missing data<br>☐ No imputation<br>☐ Multiple Imputation<br>☐ Complete case analysis<br>☐ Mean imputation<br>☐ Median imputation<br>☐ Other<br>(If there is any discrepancy between how missing values were handled for outcome and predictors, please specify so it in the comments below the methods form. Report here imputation for predictors .)
If other, please specify	______________________________
If other, please specify	______________________________
If other, please specify	______________________________
Development<br>If missing data were imputed, details of the software used are given	◯ Yes<br>◯ No<br>◯ NA
External validation<br>If missing data were imputed, details of the software used are given	◯ Yes<br>◯ No<br>◯ NA
External validation<br>If missing data were imputed, details of the software used are given	◯ Yes<br>◯ No<br>◯ NA

Confidential

Development<br>If missing data were imputed, a description of which variables were included in the imputation procedure is given.	◯ Yes<br>◯ No<br>◯ NA
External validation<br>If missing data were imputed, a description of which variables were included in the imputation procedure is given.	◯ Yes<br>◯ No<br>◯ NA
External validation<br>If missing data were imputed, a description of which variables were included in the imputation procedure is given.	◯ Yes<br>◯ No<br>◯ NA
Development<br>If multiple imputation was used, the number of imputations is reported	◯ Yes<br>◯ No
External validation<br>If multiple imputation was used, the number of imputations is reported	◯ Yes<br>◯ No
External validation<br>If multiple imputation was used, the number of imputations is reported	◯ Yes<br>◯ No
Development<br>How is missing data presented in the paper or supplemental material?	◯ Overall<br>◯ By all candidate variables<br>◯ By all final model variables<br>◯ By number of variables<br>◯ Not summarised
External validation<br>How is missing data presented in the paper or supplemental material?	◯ Overall<br>◯ By all candidate variables<br>◯ By all final model variables<br>◯ By number of variables<br>◯ Not summarised
External validation<br>How is missing data presented in the paper or supplemental material?	◯ Overall<br>◯ By all candidate variables<br>◯ By all final model variables<br>◯ By number of variables<br>◯ Not summarised
Development<br>If missing data is presented/summarised, what is the percentage/number of individuals have missing data (overall)	______________________________<br>(If this is unclear, please fill this with "UN")
External validation<br>If missing data is presented/summarised, what is the percentage/number of individuals have missing data (overall)	______________________________<br>(If this is unclear, please fill this with "UN")

Confidential

External validation<br>If missing data is presented/summarised, what is the percentage/number of individuals have missing data (overall)	__________________________________<br>(If this is unclear, please fill this with "UN")

Statistical Analysis

Data pre-processing

Did the candidate predictors include continuous variables?	◯ Yes<br>◯ No<br>◯ Unclear
For continuous predictors it is described whether they were modelled as linear, nonlinear (type of transformation specified) or categorized.	◯ Yes<br>◯ No<br>◯ Unclear<br>(A general statement is sufficient, no need to describe this for each predictors.)
How were continuous predictors handled?	☐ Linear (no change)<br>☐ Non-linear (explicity/planned)<br>☐ Non-linear (implicitly/unplanned)<br>☐ Categorised (some)<br>☐ Categorised (all)<br>☐ Other<br>(Non linear terms may not be explicitly reported/planned but handled within the model building process for ML and maybe implicit/unplanned. If an ML model is being evaluated and non-linear terms are not explicitly reported - choose 'Yes (implicit/unplanned)'. A general statement is sufficient, no need to describe this for each predictor separately.)
If other, please specify	__________________________________
For categorical or categorized predictors, the cut-points were reported	◯ Yes<br>◯ No
For categorized predictors the method to choose the cut-points was clearly described	◯ Yes<br>◯ No<br>◯ NA<br>(If no categorized predictors, score Not applicable.)
If categorised/dichotomised, how was this done?	◯ Quantiles<br>◯ Data dependent<br>◯ Mixture<br>◯ No rationale<br>◯ Based on previous literature or/and standarization

Is there any other data pre-processing methods used?	☐ Cleaning<br>☐ Aggregation<br>☐ Transformation<br>☐ Sampling<br>☐ Standardization<br>☐ Integration<br>☐ Reduction<br>☐ Other<br>☐ No<br>(Multiples answers are possible. E.g. data aggregation (calculating predictors from other collected data), other predictor transformations, data sampling (only using part of a dataset), predictor standardisation.)
If other, please specify	__________________________________<br>(list using (;) to separate if more then 1)
Is class imbalance addressed?	◯ Yes<br>◯ No<br>◯ NA<br>(There is a disproportionate ratio of observations in each class/group -most machine learning algorithms work best when the number of samples in each class are about equal.)
If yes, how?	__________________________________<br>(list using (;) to separate if more than 1)
Is class imbalance handling justified?	◯ Yes<br>◯ No
Is there any data reduction techniques used?	◯ Yes<br>◯ No<br>(E.g. Missing values ratio, Low variance filter, High correlation filter, Random Forest / Ensembles tress, Principal Component Analysis (PCA), Backward feature elimination, Forward feature construction, autoencoder, Non-negative matrix factorization, Kernel PCA, Graph-based kernel PCA, etc.)
If yes, what techniques were used?	__________________________________<br>(list using (;) to separate if more then 1)
Is data reduction justified?	◯ Yes<br>◯ No

Was collinearity assessed?	◯ Yes, implicitly<br>◯ Yes, explicitly<br>◯ No<br>◯ NA<br>(Collinearity may not be explicitly reported/planned but handled within the model building process for ML and maybe implicit/unplanned. If an ML model is being evaluated and collinearity are not explicitly reported - choose 'Yes (implicit/unplanned)'. A general statement is sufficient, no need to describe this for each predictor separately.)

Model building

Instructions

-Please extract the models in the order they are presented in the article.

-If more than 10 models were developed for the main outcome, only refer to the first 10.

-If a comparison with logistic regression was made, please included this model in the final count and extract information.

How many models were developed for the primary outcome?	__________________________________<br>(This should reflect the number of models you are going to extract on - primary outcome and primary timepoint (If more than 10 models were developed, only refer to the first 10). If a comparison with logistic regression was made, please included this model in the final count.)
External validation<br>It is described how predictions for individuals (in the validation set) were obtained from the model being validated	◯ Yes<br>◯ No<br>(E.g. Using the original reported model coefficients with or without the intercept, and/or using updated or refitted model coefficients, or using a nomogram, spreadsheet or web calculator.)

Model 1

The type of statistical modelling approach is reported	◯ Yes<br>◯ No<br>(E.g. Neural Network)
What is the ML technique being used?	◯ Neural network<br>◯ Random forest<br>◯ Classification and regression tree (CART)<br>◯ Support vector machine<br>◯ Gradient boosting machine<br>◯ Logistic regression<br>◯ Other
If other, please specify	__________________________________

Do they clearly state why the technique is selected?	__________________________________ (E.g. R code availability, clinical question, previous literature, comparison. If not reported, fill this with "NR")
The approach used for predictor selection before modelling is described	◯ Yes<br>◯ No<br>◯ NA<br>(Before modelling' means before any univariable or multivariable analysis of predictor-outcome associations. If no predictor selection before modelling is done, score Not applicable. If it is unclear whether predictor selection before modelling is done, score No. If it is clear there was predictor selection before modelling but the method was not described, score No.)
The approach used for predictor selection during modelling is described	◯ Yes<br>◯ No<br>◯ NA<br>(E.g. Univariable analysis, stepwise selection, bootstrap, Lasso. 'During modelling' includes both univariable or multivariable analysis of predictor-outcome associations. If no predictor selection during modelling is done (so-called full model approach), score Not applicable. If it is unclear whether predictor selection during modelling is done, score No. If it is clear there was predictor selection during modelling but the method was not described, score No.)
What was the model building strategy?	◯ Stepwise<br>◯ Forward selection<br>◯ Backward selection<br>◯ All predictors<br>◯ All significant in univariable<br>◯ Data-driven<br>◯ Other<br>◯ Unclear
If other, please specify	__________________________________ (list using (;) to separate if more than 1)
Are hyper-parameters tuning reported?	◯ Yes<br>◯ No<br>◯ NA<br>(Answer yes if any information is giving about how the models were set-up. Term as number of layers, node, optimization, hyperparameters, etc.)
Is predictor importance assessed?	◯ Yes<br>◯ No<br>(E.g. Importance, Mean decrease/Increase in accuracy, Mean decrease Gini, Gini Index, Average impurity decrease, etc.)

Andaur Navarro CL, *et al. BMJ Open* 2020; 10:e038832. doi: 10.1136/bmjopen-2020-038832

If yes, how?	______________________________<br>(list using (;) to separate if more then 1)
Is there any ML technique being used for predictors/feature selection?	◯ Yes<br>◯ No<br>(E.g. SVM)
If yes, which technique?	______________________________<br>(list using (;) to separate if more then 1)
Testing of interaction terms is described	◯ Yes, explicitly.<br>◯ Yes, implicitly.<br>◯ No<br>(Interaction terms may not be explicitly reported/planned but handled within the model building process for ML and maybe implicit/unplanned. If an ML model is being evaluated and interactions are not explicitly reported - choose 'Yes (implicit/unplanned)'. A general statement is sufficient, no need to describe this for each predictor separately.)
If applicable, how was censoring accounted for?	______________________________<br>(If not applicable, fill this with "NA")
Testing of the proportionality of hazards in survival models is described	◯ Yes<br>◯ No<br>◯ NA<br>(If no proportional hazard model is used, score Not applicable.)
What shrinkage/penalisation methods were used?	◯ None<br>◯ Uniform shrinkage<br>◯ Penalised estimation<br>◯ Other
If other, please specify	______________________________

Model 2

The type of statistical modelling approach is reported	◯ Yes<br>◯ No<br>(E.g. Neural Network)
What is the ML technique being used?	◯ Neural network<br>◯ Random forest<br>◯ Classification and regression tree (CART)<br>◯ Support vector machine<br>◯ Gradient boosting machine<br>◯ Logistic regression<br>◯ Other
If other, please specify	______________________________

Do they clearly state why the technique is selected?	__________________________________<br>(E.g. R code availability, clinical question, previous literature, comparison. If not reported, fill this with "NR")
The approach used for predictor selection before modelling is described	◯ Yes<br>◯ No<br>◯ NA<br>(Before modelling' means before any univariable or multivariable analysis of predictor-outcome associations. If no predictor selection before modelling is done, score Not applicable. If it is unclear whether predictor selection before modelling is done, score No. If it is clear there was predictor selection before modelling but the method was not described, score No.)
The approach used for predictor selection during modelling is described	◯ Yes<br>◯ No<br>◯ NA<br>(E.g. Univariable analysis, stepwise selection, bootstrap, Lasso. 'During modelling' includes both univariable or multivariable analysis of predictor-outcome associations. If no predictor selection during modelling is done (so-called full model approach), score Not applicable. If it is unclear whether predictor selection during modelling is done, score No. If it is clear there was predictor selection during modelling but the method was not described, score No.)
What was the model building strategy?	◯ Stepwise<br>◯ Forward selection<br>◯ Backward selection<br>◯ All predictors<br>◯ All significant in univariable<br>◯ Data-driven<br>◯ Other<br>◯ Unclear
If other, please specify	__________________________________<br>(list using (;) to separate if more than 1)
Are hyper-parameters tuning reported?	◯ Yes<br>◯ No<br>◯ NA<br>(Answer yes if any information is giving about how the models were set-up. Term as number of layers, node, optimization, hyperparameters, etc.)
Is predictor importance assessed?	◯ Yes<br>◯ No<br>(E.g. Importance, Mean decrease/Increase in accuracy, Mean decrease Gini, Gini Index, Average impurity decrease, etc.)

If yes, how?	__________________________________<br>(list using (;) to separate if more than 1)
Is there any ML technique being used for predictors/feature selection?	◯ Yes<br>◯ No<br>(E.g. SVM)
If yes, which technique?	__________________________________<br>(list using (;) to separate if more than 1)
Testing of interaction terms is described	◯ Yes, explicitly.<br>◯ Yes, implicitly.<br>◯ No<br>(Interaction terms may not be explicitly reported/planned but handled within the model building process for ML and maybe implicit/unplanned. If an ML model is being evaluated and interactions are not explicitly reported - choose 'Yes (implicit/unplanned)'. A general statement is sufficient, no need to describe this for each predictor separately.)
If applicable, how was censoring accounted for?	__________________________________<br>(If not applicable, fill this with "NA")
Testing of the proportionality of hazards in survival models is described	◯ Yes<br>◯ No<br>◯ NA
What shrinkage/penalisation methods were used?	◯ None<br>◯ Uniform shrinkage<br>◯ Penalised estimation<br>◯ Other
If other, please specify	__________________________________

Model 3

The type of statistical modelling approach is reported	◯ Yes<br>◯ No<br>(E.g. Neural Network)
What is the ML technique being used?	◯ Neural network<br>◯ Random forest<br>◯ Classification and regression tree (CART)<br>◯ Support vector machine<br>◯ Gradient boosting machine<br>◯ Logistic regression<br>◯ Other
If other, please specify	__________________________________

Do they clearly state why the technique is selected?	______________________________<br>(E.g. R code availability, clinical question, previous literature, comparison. If not reported, fill this with "NR")
The approach used for predictor selection before modelling is described	◯ Yes<br>◯ No<br>◯ NA<br>(Before modelling' means before any univariable or multivariable analysis of predictor-outcome associations. If no predictor selection before modelling is done, score Not applicable. If it is unclear whether predictor selection before modelling is done, score No. If it is clear there was predictor selection before modelling but the method was not described, score No.)
The approach used for predictor selection during modelling is described	◯ Yes<br>◯ No<br>◯ NA<br>(E.g. Univariable analysis, stepwise selection, bootstrap, Lasso. 'During modelling' includes both univariable or multivariable analysis of predictor-outcome associations. If no predictor selection during modelling is done (so-called full model approach), score Not applicable. If it is unclear whether predictor selection during modelling is done, score No. If it is clear there was predictor selection during modelling but the method was not described, score No.)
What was the model building strategy?	◯ Stepwise<br>◯ Forward selection<br>◯ Backward selection<br>◯ All predictors<br>◯ All significant in univariable<br>◯ Data-driven<br>◯ Other<br>◯ Unclear
If other, please specify	______________________________<br>(list using (;) to separate if more than 1)
Are hyper-parameters tuning reported?	◯ Yes<br>◯ No<br>◯ NA<br>(Answer yes if any information is giving about how the models were set-up. Term as number of layers, node, optimization, hyperparameters, etc.)
Is predictor importance assessed?	◯ Yes<br>◯ No<br>(E.g. Importance, Mean decrease/Increase in accuracy, Mean decrease Gini, Gini Index, Average impurity decrease, etc.)

Andaur Navarro CL, *et al. BMJ Open* 2020; 10:e038832. doi: 10.1136/bmjopen-2020-038832

If yes, how?	______________________________<br>(list using (;) to separate if more than 1)
Is there any ML technique being used for predictors/feature selection?	◯ Yes<br>◯ No<br>(E.g. SVM)
If yes, which technique?	______________________________<br>(list using (;) to separate if more than 1)
Testing of interaction terms is described	◯ Yes, explicitly.<br>◯ Yes, implicitly.<br>◯ No<br>(Interaction terms may not be explicitly reported/planned but handled within the model building process for ML and maybe implicit/unplanned. If an ML model is being evaluated and interactions are not explicitly reported - choose 'Yes (implicit/unplanned)'. A general statement is sufficient, no need to describe this for each predictor separately.)
If applicable, how was censoring accounted for?	______________________________<br>(If not applicable, fill this with "NA")
Testing of the proportionality of hazards in survival models is described	◯ Yes<br>◯ No<br>◯ NA
What shrinkage/penalisation methods were used?	◯ None<br>◯ Uniform shrinkage<br>◯ Penalised estimation<br>◯ Other
If other, please specify	______________________________

Model 4

The type of statistical modelling approach is reported	◯ Yes<br>◯ No<br>(E.g. Neural Network)
What is the ML technique being used?	◯ Neural network<br>◯ Random forest<br>◯ Classification and regression tree (CART)<br>◯ Support vector machine<br>◯ Gradient boosting machine<br>◯ Logistic regression<br>◯ Other
If other, please specify	______________________________

Confidential

Do they clearly state why the technique is selected?	__________________________________<br>(E.g. R code availability, clinical question, previous literature, comparison. If not reported, fill this with "NR")
The approach used for predictor selection before modelling is described	◯ Yes<br>◯ No<br>◯ NA<br>(Before modelling' means before any univariable or multivariable analysis of predictor-outcome associations. If no predictor selection before modelling is done, score Not applicable. If it is unclear whether predictor selection before modelling is done, score No. If it is clear there was predictor selection before modelling but the method was not described, score No.)
The approach used for predictor selection during modelling is described	◯ Yes<br>◯ No<br>◯ NA<br>(E.g. Univariable analysis, stepwise selection, bootstrap, Lasso. 'During modelling' includes both univariable or multivariable analysis of predictor-outcome associations. If no predictor selection during modelling is done (so-called full model approach), score Not applicable. If it is unclear whether predictor selection during modelling is done, score No. If it is clear there was predictor selection during modelling but the method was not described, score No.)
What was the model building strategy?	◯ Stepwise<br>◯ Forward selection<br>◯ Backward selection<br>◯ All predictors<br>◯ All significant in univariable<br>◯ Data-driven<br>◯ Other<br>◯ Unclear
If other, please specify	__________________________________<br>(list using (;) to separate if more than 1)
Are hyper-parameters tuning reported?	◯ Yes<br>◯ No<br>◯ NA<br>(Answer yes if any information is giving about how the models were set-up. Term as number of layers, node, optimization, hyperparameters, etc.)
Is predictor importance assessed?	◯ Yes<br>◯ No<br>(E.g. Importance, Mean decrease/Increase in accuracy, Mean decrease Gini, Gini Index, Average impurity decrease, etc.)

Andaur Navarro CL, *et al. BMJ Open* 2020; 10:e038832. doi: 10.1136/bmjopen-2020-038832

If yes, how?	___________________________________<br>(list using (;) to separate if more than 1)
Is there any ML technique being used for predictors/feature selection?	◯ Yes<br>◯ No<br>(E.g. SVM)
If yes, which technique?	___________________________________<br>(list using (;) to separate if more than 1)
Testing of interaction terms is described	◯ Yes, explicitly.<br>◯ Yes, implicitly.<br>◯ No<br>(Interaction terms may not be explicitly reported/planned but handled within the model building process for ML and maybe implicit/unplanned. If an ML model is being evaluated and interactions are not explicitly reported - choose 'Yes (implicit/unplanned)'. A general statement is sufficient, no need to describe this for each predictor separately.)
If applicable, how was censoring accounted for?	___________________________________<br>(If not applicable, fill this with "NA")
Testing of the proportionality of hazards in survival models is described	◯ Yes<br>◯ No<br>◯ NA
What shrinkage/penalisation methods were used?	◯ None<br>◯ Uniform shrinkage<br>◯ Penalised estimation<br>◯ Other
If other, please specify	___________________________________

Model 5

The type of statistical modelling approach is reported	◯ Yes<br>◯ No<br>(E.g. Neural Network)
What is the ML technique being used?	◯ Neural network<br>◯ Random forest<br>◯ Classification and regression tree (CART)<br>◯ Support vector machine<br>◯ Gradient boosting machine<br>◯ Logistic regression<br>◯ Other
If other, please specify	___________________________________

Do they clearly state why the technique is selected?	__________________________________<br>(E.g. R code availability, clinical question, previous literature, comparison. If not reported, fill this with "NR")
The approach used for predictor selection before modelling is described	◯ Yes<br>◯ No<br>◯ NA<br>(Before modelling' means before any univariable or multivariable analysis of predictor-outcome associations. If no predictor selection before modelling is done, score Not applicable. If it is unclear whether predictor selection before modelling is done, score No. If it is clear there was predictor selection before modelling but the method was not described, score No.)
The approach used for predictor selection during modelling is described	◯ Yes<br>◯ No<br>◯ NA<br>(E.g. Univariable analysis, stepwise selection, bootstrap, Lasso. 'During modelling' includes both univariable or multivariable analysis of predictor-outcome associations. If no predictor selection during modelling is done (so-called full model approach), score Not applicable. If it is unclear whether predictor selection during modelling is done, score No. If it is clear there was predictor selection during modelling but the method was not described, score No.)
What was the model building strategy?	◯ Stepwise<br>◯ Forward selection<br>◯ Backward selection<br>◯ All predictors<br>◯ All significant in univariable<br>◯ Data-driven<br>◯ Other<br>◯ Unclear
If other, please specify	__________________________________<br>(list using (;) to separate if more than 1)
Are hyper-parameters tuning reported?	◯ Yes<br>◯ No<br>◯ NA<br>(Answer yes if any information is giving about how the models were set-up. Term as number of layers, node, optimization, hyperparameters, etc.)
Is predictor importance assessed?	◯ Yes<br>◯ No<br>(E.g. Importance, Mean decrease/Increase in accuracy, Mean decrease Gini, Gini Index, Average impurity decrease, etc.)

Confidential

If yes, how?	______________________________<br>(list using (;) to separate if more than 1)
Is there any ML technique being used for predictors/feature selection?	◯ Yes<br>◯ No<br>(E.g. SVM)
If yes, which technique?	______________________________<br>(list using (;) to separate if more than 1)
Testing of interaction terms is described	◯ Yes, explicitly.<br>◯ Yes, implicitly.<br>◯ No<br>(Interaction terms may not be explicitly reported/planned but handled within the model building process for ML and maybe implicit/unplanned. If an ML model is being evaluated and interactions are not explicitly reported - choose 'Yes (implicit/unplanned)'. A general statement is sufficient, no need to describe this for each predictor separately.)
If applicable, how was censoring accounted for?	______________________________<br>(If not applicable, fill this with "NA")
Testing of the proportionality of hazards in survival models is described	◯ Yes<br>◯ No<br>◯ NA
What shrinkage/penalisation methods were used?	◯ None<br>◯ Uniform shrinkage<br>◯ Penalised estimation<br>◯ Other
If other, please specify	______________________________

Model 6

The type of statistical modelling approach is reported	◯ Yes<br>◯ No<br>(E.g. Neural Network)
What is the ML technique being used?	◯ Neural network<br>◯ Random forest<br>◯ Classification and regression tree (CART)<br>◯ Support vector machine<br>◯ Gradient boosting machine<br>◯ Logistic regression<br>◯ Other
If other, please specify	______________________________

Do they clearly state why the technique is selected?	________________________________<br>(E.g. R code availability, clinical question, previous literature, comparison. If not reported, fill this with "NR")
The approach used for predictor selection before modelling is described	◯ Yes<br>◯ No<br>◯ NA<br>(Before modelling' means before any univariable or multivariable analysis of predictor-outcome associations. If no predictor selection before modelling is done, score Not applicable. If it is unclear whether predictor selection before modelling is done, score No. If it is clear there was predictor selection before modelling but the method was not described, score No.)
The approach used for predictor selection during modelling is described	◯ Yes<br>◯ No<br>◯ NA<br>(E.g. Univariable analysis, stepwise selection, bootstrap, Lasso. 'During modelling' includes both univariable or multivariable analysis of predictor-outcome associations. If no predictor selection during modelling is done (so-called full model approach), score Not applicable. If it is unclear whether predictor selection during modelling is done, score No. If it is clear there was predictor selection during modelling but the method was not described, score No.)
What was the model building strategy?	◯ Stepwise<br>◯ Forward selection<br>◯ Backward selection<br>◯ All predictors<br>◯ All significant in univariable<br>◯ Data-driven<br>◯ Other<br>◯ Unclear
If other, please specify	________________________________<br>(list using (;) to separate if more than 1)
Are hyper-parameters tuning reported?	◯ Yes<br>◯ No<br>◯ NA<br>(Answer yes if any information is giving about how the models were set-up. Term as number of layers, node, optimization, hyperparameters, etc.)
Is predictor importance assessed?	◯ Yes<br>◯ No<br>(E.g. Importance, Mean decrease/Increase in accuracy, Mean decrease Gini, Gini Index, Average impurity decrease, etc.)

Andaur Navarro CL, *et al. BMJ Open* 2020; 10:e038832. doi: 10.1136/bmjopen-2020-038832

If yes, how?	______________________________<br>(list using (;) to separate if more than 1)
Is there any ML technique being used for predictors/feature selection?	◯ Yes<br>◯ No<br>(E.g. SVM)
If yes, which technique?	______________________________<br>(list using (;) to separate if more than 1)
Testing of interaction terms is described	◯ Yes, implicitly.<br>◯ Yes, explicitly.<br>◯ No<br>(Interaction terms may not be explicitly reported/planned but handled within the model building process for ML and maybe implicit/unplanned. If an ML model is being evaluated and interactions are not explicitly reported - choose 'Yes (implicit/unplanned)'. A general statement is sufficient, no need to describe this for each predictor separately.)
If applicable, how was censoring accounted for?	______________________________<br>(If not applicable, fill this with "NA")
Testing of the proportionality of hazards in survival models is described	◯ Yes<br>◯ No<br>◯ NA
What shrinkage/penalisation methods were used?	◯ None<br>◯ Uniform shrinkage<br>◯ Penalised estimation<br>◯ Other
If other, please specify	______________________________

Model 7

The type of statistical modelling approach is reported	◯ Yes<br>◯ No<br>(E.g. Neural Network)
What is the ML technique being used?	◯ Neural network<br>◯ Random forest<br>◯ Classification and regression tree (CART)<br>◯ Support vector machine<br>◯ Gradient boosting machine<br>◯ Logistic regression<br>◯ Other
If other, please specify	______________________________

Confidential

Do they clearly state why the technique is selected?	__________________________________<br>(E.g. R code availability, clinical question, previous literature, comparison. If not reported, fill this with "NR")
The approach used for predictor selection before modelling is described	◯ Yes<br>◯ No<br>◯ NA<br>(Before modelling' means before any univariable or multivariable analysis of predictor-outcome associations. If no predictor selection before modelling is done, score Not applicable. If it is unclear whether predictor selection before modelling is done, score No. If it is clear there was predictor selection before modelling but the method was not described, score No.)
The approach used for predictor selection during modelling is described	◯ Yes<br>◯ No<br>◯ NA<br>(E.g. Univariable analysis, stepwise selection, bootstrap, Lasso. 'During modelling' includes both univariable or multivariable analysis of predictor-outcome associations. If no predictor selection during modelling is done (so-called full model approach), score Not applicable. If it is unclear whether predictor selection during modelling is done, score No. If it is clear there was predictor selection during modelling but the method was not described, score No.)
What was the model building strategy?	◯ Stepwise<br>◯ Forward selection<br>◯ Backward selection<br>◯ All predictors<br>◯ All significant in univariable<br>◯ Data-driven<br>◯ Other<br>◯ Unclear
If other, please specify	__________________________________<br>(list using (;) to separate if more than 1)
Are hyper-parameters tuning reported?	◯ Yes<br>◯ No<br>◯ NA<br>(Answer yes if any information is giving about how the models were set-up. Term as number of layers, node, optimization, hyperparameters, etc.)
Is predictor importance assessed?	◯ Yes<br>◯ No<br>(E.g. Importance, Mean decrease/Increase in accuracy, Mean decrease Gini, Gini Index, Average impurity decrease, etc.)

Andaur Navarro CL, *et al. BMJ Open* 2020; 10:e038832. doi: 10.1136/bmjopen-2020-038832

If yes, how?	__________________________________<br>(list using (;) to separate if more than 1)
Is there any ML technique being used for predictors/feature selection?	◯ Yes<br>◯ No<br>(E.g. SVM)
If yes, which technique?	__________________________________<br>(list using (;) to separate if more than 1)
Testing of interaction terms is described	◯ Yes, implicitly.<br>◯ Yes, explicitly.<br>◯ No<br>(Interaction terms may not be explicitly reported/planned but handled within the model building process for ML and maybe implicit/unplanned. If an ML model is being evaluated and interactions are not explicitly reported - choose 'Yes (implicit/unplanned)'. A general statement is sufficient, no need to describe this for each predictor separately.)
If applicable, how was censoring accounted for?	__________________________________<br>(If not applicable, fill this with "NA")
Testing of the proportionality of hazards in survival models is described	◯ Yes<br>◯ No<br>◯ NA
What shrinkage/penalisation methods were used?	◯ None<br>◯ Uniform shrinkage<br>◯ Penalised estimation<br>◯ Other
If other, please specify	__________________________________

Model 8

The type of statistical modelling approach is reported	◯ Yes<br>◯ No<br>(E.g. Neural Network)
What is the ML technique being used?	◯ Neural network<br>◯ Random forest<br>◯ Classification and regression tree (CART)<br>◯ Support vector machine<br>◯ Gradient boosting machine<br>◯ Logistic regression<br>◯ Other
If other, please specify	__________________________________

Do they clearly state why the technique is selected?	__________________________________<br>(E.g. R code availability, clinical question, previous literature, comparison. If not reported, fill this with "NR")
The approach used for predictor selection before modelling is described	◯ Yes<br>◯ No<br>◯ NA<br>(Before modelling' means before any univariable or multivariable analysis of predictor-outcome associations. If no predictor selection before modelling is done, score Not applicable. If it is unclear whether predictor selection before modelling is done, score No. If it is clear there was predictor selection before modelling but the method was not described, score No.)
The approach used for predictor selection during modelling is described	◯ Yes<br>◯ No<br>◯ NA<br>(E.g. Univariable analysis, stepwise selection, bootstrap, Lasso. 'During modelling' includes both univariable or multivariable analysis of predictor-outcome associations. If no predictor selection during modelling is done (so-called full model approach), score Not applicable. If it is unclear whether predictor selection during modelling is done, score No. If it is clear there was predictor selection during modelling but the method was not described, score No.)
What was the model building strategy?	◯ Stepwise<br>◯ Forward selection<br>◯ Backward selection<br>◯ All predictors<br>◯ All significant in univariable<br>◯ Data-driven<br>◯ Other<br>◯ Unclear
If other, please specify	__________________________________<br>(list using (;) to separate if more than 1)
Are hyper-parameters tuning reported?	◯ Yes<br>◯ No<br>◯ NA<br>(Answer yes if any information is giving about how the models were set-up. Term as number of layers, node, optimization, hyperparameters, etc.)
Is predictor importance assessed?	◯ Yes<br>◯ No<br>(E.g. Importance, Mean decrease/Increase in accuracy, Mean decrease Gini, Gini Index, Average impurity decrease, etc.)

If yes, how?	__________________________________ (list using (;) to separate if more than 1)
Is there any ML technique being used for predictors/feature selection?	◯ Yes<br>◯ No<br>(E.g. SVM)
If yes, which technique?	__________________________________ (list using (;) to separate if more than 1)
Testing of interaction terms is described	◯ Yes, implicitly.<br>◯ Yes, explicitly.<br>◯ No<br>(Interaction terms may not be explicitly reported/planned but handled within the model building process for ML and maybe implicit/unplanned. If an ML model is being evaluated and interactions are not explicitly reported - choose 'Yes (implicit/unplanned)'. A general statement is sufficient, no need to describe this for each predictor separately.)
If applicable, how was censoring accounted for?	__________________________________ (If not applicable, fill this with "NA")
Testing of the proportionality of hazards in survival models is described	◯ Yes<br>◯ No<br>◯ NA
What shrinkage/penalisation methods were used?	◯ None<br>◯ Uniform shrinkage<br>◯ Penalised estimation<br>◯ Other
If other, please specify	__________________________________

Model 9

The type of statistical modelling approach is reported	◯ Yes<br>◯ No<br>(E.g. Neural Network)
What is the ML technique being used?	◯ Neural network<br>◯ Random forest<br>◯ Classification and regression tree (CART)<br>◯ Support vector machine<br>◯ Gradient boosting machine<br>◯ Logistic regression<br>◯ Other
If other, please specify	__________________________________

Do they clearly state why the technique is selected?	________________________________<br>(E.g. R code availability, clinical question, previous literature, comparison. If not reported, fill this with "NR")
The approach used for predictor selection before modelling is described	◯ Yes<br>◯ No<br>◯ NA<br>(Before modelling' means before any univariable or multivariable analysis of predictor-outcome associations. If no predictor selection before modelling is done, score Not applicable. If it is unclear whether predictor selection before modelling is done, score No. If it is clear there was predictor selection before modelling but the method was not described, score No.)
The approach used for predictor selection during modelling is described	◯ Yes<br>◯ No<br>◯ NA<br>(E.g. Univariable analysis, stepwise selection, bootstrap, Lasso. 'During modelling' includes both univariable or multivariable analysis of predictor-outcome associations. If no predictor selection during modelling is done (so-called full model approach), score Not applicable. If it is unclear whether predictor selection during modelling is done, score No. If it is clear there was predictor selection during modelling but the method was not described, score No.)
What was the model building strategy?	◯ Stepwise<br>◯ Forward selection<br>◯ Backward selection<br>◯ All predictors<br>◯ All significant in univariable<br>◯ Data-driven<br>◯ Other<br>◯ Unclear
If other, please specify	________________________________<br>(list using (;) to separate if more than 1)
Are hyper-parameters tuning reported?	◯ Yes<br>◯ No<br>◯ NA<br>(Answer yes if any information is giving about how the models were set-up. Term as number of layers, node, optimization, hyperparameters, etc.)
Is predictor importance assessed?	◯ Yes<br>◯ No<br>(E.g. Importance, Mean decrease/Increase in accuracy, Mean decrease Gini, Gini Index, Average impurity decrease, etc.)

If yes, how?	______________________ (list using (;) to separate if more than 1)
Is there any ML technique being used for predictors/feature selection?	◯ Yes<br>◯ No<br>(E.g. SVM)
If yes, which technique?	______________________ (list using (;) to separate if more than 1)
Testing of interaction terms is described	◯ Yes, implicitly.<br>◯ Yes, explicitly.<br>◯ No<br>(Interaction terms may not be explicitly reported/planned but handled within the model building process for ML and maybe implicit/unplanned. If an ML model is being evaluated and interactions are not explicitly reported - choose 'Yes (implicit/unplanned)'. A general statement is sufficient, no need to describe this for each predictor separately.)
If applicable, how was censoring accounted for?	______________________ (If not applicable, fill this with "NA")
Testing of the proportionality of hazards in survival models is described	◯ Yes<br>◯ No<br>◯ NA
What shrinkage/penalisation methods were used?	◯ None<br>◯ Uniform shrinkage<br>◯ Penalised estimation<br>◯ Other
If other, please specify	______________________

Model 10

The type of statistical modelling approach is reported	◯ Yes<br>◯ No<br>(E.g. Neural Network)
What is the ML technique being used?	◯ Neural network<br>◯ Random forest<br>◯ Classification and regression tree (CART)<br>◯ Support vector machine<br>◯ Gradient boosting machine<br>◯ Logistic regression<br>◯ Other
If other, please specify	______________________

Do they clearly state why the technique is selected?	__________________________________<br>(E.g. R code availability, clinical question, previous literature, comparison. If not reported, fill this with "NR")
The approach used for predictor selection before modelling is described	◯ Yes<br>◯ No<br>◯ NA<br>(Before modelling' means before any univariable or multivariable analysis of predictor-outcome associations. If no predictor selection before modelling is done, score Not applicable. If it is unclear whether predictor selection before modelling is done, score No. If it is clear there was predictor selection before modelling but the method was not described, score No.)
The approach used for predictor selection during modelling is described	◯ Yes<br>◯ No<br>◯ NA<br>(E.g. Univariable analysis, stepwise selection, bootstrap, Lasso. 'During modelling' includes both univariable or multivariable analysis of predictor-outcome associations. If no predictor selection during modelling is done (so-called full model approach), score Not applicable. If it is unclear whether predictor selection during modelling is done, score No. If it is clear there was predictor selection during modelling but the method was not described, score No.)
What was the model building strategy?	◯ Stepwise<br>◯ Forward selection<br>◯ Backward selection<br>◯ All predictors<br>◯ All significant in univariable<br>◯ Data-driven<br>◯ Other<br>◯ Unclear
If other, please specify	__________________________________<br>(list using (;) to separate if more than 1)
Are hyper-parameters tuning reported?	◯ Yes<br>◯ No<br>◯ NA<br>(Answer yes if any information is giving about how the models were set-up. Term as number of layers, node, optimization, hyperparameters, etc.)
Is predictor importance assessed?	◯ Yes<br>◯ No<br>(E.g. Importance, Mean decrease/Increase in accuracy, Mean decrease Gini, Gini Index, Average impurity decrease, etc.)

If yes, how?	______________________________<br>(list using (;) to separate if more than 1)
Is there any ML technique being used for predictors/feature selection?	◯ Yes<br>◯ No<br>(E.g. SVM)
If yes, which technique?	______________________________<br>(list using (;) to separate if more than 1)
Testing of interaction terms is described	◯ Yes, implicitly.<br>◯ Yes, explicitly.<br>◯ No<br>(Interaction terms may not be explicitly reported/planned but handled within the model building process for ML and maybe implicit/unplanned. If an ML model is being evaluated and interactions are not explicitly reported - choose 'Yes (implicit/unplanned)'. A general statement is sufficient, no need to describe this for each predictor separately.)
If applicable, how was censoring accounted for?	______________________________<br>(If not applicable, fill this with "NA")
Testing of the proportionality of hazards in survival models is described	◯ Yes<br>◯ No<br>◯ NA
What shrinkage/penalisation methods were used?	◯ None<br>◯ Uniform shrinkage<br>◯ Penalised estimation<br>◯ Other
If other, please specify	______________________________

Internal validation

Internal validation is reported	◯ Yes<br>◯ No<br>(If the use of internal validation is clearly a non-issue (e.g. in case of very large data sets), score Yes. For all other situations an explicit mention is expected.)
How is the model internally validated?	☐ Split sample<br>☐ Bootstrapping<br>☐ Cross-validation<br>☐ Other<br>☐ Unclear
If split sample, what % split was used for the development?	______________________________<br>(If not reported, please fill this with "UN")

If split sample, was it a random or non-random split?	◯ Random<br>◯ Non-random<br>◯ Unclear
If non-random split was used, how?	__________________<br>(E.g. Temporal splitting)
If bootstrap method, how many were performed?	__________________<br>(If not reported, please fill this with "UN")
If bootstrap method, were selection of variables included in the bootstrap?	◯ Yes<br>◯ No
If cross validation, please specify the method used	__________________<br>(E.g. ten-fold. If not reported, fill this with "UN")

Model Performance

Development<br>Measures for model discrimination are described	◯ Yes<br>◯ No
External validation<br>Measures for model discrimination are described	◯ Yes<br>◯ No
External validation<br>Measures for model discrimination are described	◯ Yes<br>◯ No
Development<br>How was discrimination assessed?	☐ AUC/ AUROC/Area under the curve<br>☐ C-statistic<br>☐ Harrell's C-index<br>☐ D-Statistic<br>☐ Other
External validation<br>How was discrimination assessed?	☐ AUC/ AUROC/Area under the curve<br>☐ C-statistic<br>☐ Harrell's C-index<br>☐ D-Statistic<br>☐ Other
External validation<br>How was discrimination assessed?	☐ AUC/ AUROC/Area under the curve<br>☐ C-statistic<br>☐ Harrell's C-index<br>☐ D-Statistic<br>☐ Other
If other, please specify	__________________<br>(list using (;) to separate if more than 1)
If other, please specify	__________________<br>(list using (;) to separate if more than 1)

If other, please specify

____
(list using (;) to separate if more than 1)

Development
Measures for model calibration are described

◯ Yes
◯ No

External validation
Measures for model calibration are described

◯ Yes
◯ No

External validation
Measures for model calibration are described

◯ Yes
◯ No

Development
How was calibration assessed?

☐ H-L
☐ Calibration plot
☐ Calibration slope
☐ Calibration intercept
☐ Calibration in the large
☐ Calibration table
☐ Kappa
☐ Observed/expected ratio
☐ Other

External validation
How was calibration assessed?

☐ H-L
☐ Calibration plot
☐ Calibration slope
☐ Calibration intercept
☐ Calibration in the large
☐ Calibration table
☐ Kappa
☐ Observed/expected ratio
☐ Other

External validation
How was calibration assessed?

☐ H-L
☐ Calibration plot
☐ Calibration slope
☐ Calibration intercept
☐ Calibration in the large
☐ Calibration table
☐ Kappa
☐ Observed/expected ratio
☐ Other

If other, please specify

____

If other, please specify

____

If other, please specify

____

Andaur Navarro CL, *et al. BMJ Open* 2020; 10:e038832. doi: 10.1136/bmjopen-2020-038832

Development<br>Other performance measures are described	◯ Yes<br>◯ No<br>(E.g. R2, Brier score, predictive values, sensitivity, specificity, AUC difference, decision curve analysis, net reclassification improvement, integrated discrimination improvement, AIC)
External validation<br>Other performance measures are described	◯ Yes<br>◯ No<br>(E.g. R2, Brier score, predictive values, sensitivity, specificity, AUC difference, decision curve analysis, net reclassification improvement, integrated discrimination improvement, AIC)
External validation<br>Other performance measures are described	◯ Yes<br>◯ No<br>(E.g. R2, Brier score, predictive values, sensitivity, specificity, AUC difference, decision curve analysis, net reclassification improvement, integrated discrimination improvement, AIC)
Development<br>If yes, please specify	☐ R2<br>☐ Brier score<br>☐ predictive values<br>☐ sensitivity<br>☐ specificity<br>☐ AUC difference<br>☐ decision curve analysis<br>☐ net reclassification improvement<br>☐ integrated discrimination improvement<br>☐ AIC<br>☐ Other
External validation<br>If yes, please specify	☐ R2<br>☐ Brier score<br>☐ predictive values<br>☐ sensitivity<br>☐ specificity<br>☐ AUC difference<br>☐ decision curve analysis<br>☐ net reclassification improvement<br>☐ integrated discrimination improvement<br>☐ AIC<br>☐ Other
External validation<br>If yes, please specify	☐ R2<br>☐ Brier score<br>☐ predictive values<br>☐ sensitivity<br>☐ specificity<br>☐ AUC difference<br>☐ decision curve analysis<br>☐ net reclassification improvement<br>☐ integrated discrimination improvement<br>☐ AIC<br>☐ Other

If other, please specify	__________________________________
If other, please specify	__________________________________
If other, please specify	__________________________________

Model Updating

A description of model-updating is given	◯ Yes<br>◯ No<br>◯ NA<br>(E.g. Intercept recalibration, regression coefficient recalibration, refitting the whole model, adding a new predictor If updating was done, it should be clear which updating method was applied to score Yes. If it is not explicitly mentioned that updating was applied in the study, score this item as 'Not applicable'.)
If yes, please specify	__________________________________<br>(E.g. Intercept recalibration, regression coefficient recalibration, refitting the whole model, adding a new predictor.)

Risk groups

Development<br>Were risk groups created?	◯ Yes<br>◯ No
External validation<br>Were risk groups created?	◯ Yes<br>◯ No
External validation<br>Were risk groups created?	◯ Yes<br>◯ No
How many risk groups were created?	__________________________________
How many risk groups were created?	__________________________________
How many risk groups were created?	__________________________________
Development<br>What method was used to create these risk groups?	☐ Count factors present<br>☐ Data driven<br>☐ Equal size<br>☐ Other data dependent<br>☐ Unclear

External validation<br>What method was used to create these risk groups?	☐ Count factors present<br>☐ Data driven<br>☐ Equal size<br>☐ Other data dependent<br>☐ Unclear
External validation<br>What method was used to create these risk groups?	☐ Count factors present<br>☐ Data driven<br>☐ Equal size<br>☐ Other data dependent<br>☐ Unclear
Development<br>If risk groups were created, risk group boundaries (risk thresholds) are specified	◯ Yes<br>◯ No<br>(Score this item separately for development and validation if a study includes both development and validation.)
External validation<br>If risk groups were created, risk group boundaries (risk thresholds) are specified	◯ Yes<br>◯ No<br>(Score this item separately for development and validation if a study includes both development and validation.)
External validation<br>If risk groups were created, risk group boundaries (risk thresholds) are specified	◯ Yes<br>◯ No<br>(Score this item separately for development and validation if a study includes both development and validation.)
Development<br>Is any subgroup analysis prespecified?	◯ Yes<br>◯ No
External validation<br>Is any subgroup analysis prespecified?	◯ Yes<br>◯ No
External validation<br>Is any subgroup analysis prespecified?	◯ Yes<br>◯ No
If yes, how many subgroup criteria were defined?	________________________
If yes, how many subgroup criteria were defined?	________________________
If yes, how many subgroup criteria were defined?	________________________
Development<br>Is any sensitivity/subpopulation analysis prespecified?	◯ Yes<br>◯ No
External validation<br>Is any sensitivity/subpopulation analysis prespecified?	◯ Yes<br>◯ No

External validation<br>Is any sensitivity/subpopulation analysis prespecified?	◯ Yes<br>◯ No
Is yes, how many subpopulation criteria area defined?	__________________________________
Is yes, how many subpopulation criteria area defined?	__________________________________
Is yes, how many subpopulation criteria area defined?	__________________________________

Comments

Any additional comment about the methods section of this article?	____<br>(If there is something in the "Methods" section that does not fit into the questions of this form - please use this space to detail. Also use this space to detail anything you are unsure about.)

Confidential

Results

Article ID	________________________
What type of study is reported?	◯ Development (including internal validation)<br>◯ Development with external validation (same model)<br>◯ Development with external validation (different model)<br>◯ External validation only<br>(This questions is repeated due to branching logic. Please answer again.)
Is there any diagram/draw to clarify the results?	◯ Yes<br>◯ No

Participants

Development<br>The flow of participants is reported	◯ Yes<br>◯ No
External validation<br>The flow of participants is reported	◯ Yes<br>◯ No
External validation<br>The flow of participants is reported	◯ Yes<br>◯ No
Development<br>The number of participants with and without the outcome is reported	◯ Yes<br>◯ No<br>◯ NA<br>(If outcomes are continuous, score Not applicable.)
External validation<br>The number of participants with and without the outcome is reported	◯ Yes<br>◯ No<br>◯ NA<br>(If outcomes are continuous, score Not applicable.)
External validation<br>The number of participants with and without the outcome is reported	◯ Yes<br>◯ No<br>◯ NA<br>(If outcomes are continuous, score Not applicable.)
Development<br>A summary of follow-up time is presented	◯ Yes (median)<br>◯ Yes (average)<br>◯ Other<br>◯ No<br>◯ NA<br>(This notably applies to prognosis studies and diagnostic studies with follow-up as diagnostic outcome. If this is not applicable for an article (i.e. diagnostic study or no follow-up), then score Not applicable.)

External validation<br>A summary of follow-up time is presented	◯ Yes (median)<br>◯ Yes (average)<br>◯ Other<br>◯ No<br>◯ NA<br>(This notably applies to prognosis studies and diagnostic studies with follow-up as diagnostic outcome. If this is not applicable for an article (i.e. diagnostic study or no follow-up), then score Not applicable.)
External validation<br>A summary of follow-up time is presented	◯ Yes (median)<br>◯ Yes (average)<br>◯ Other<br>◯ No<br>◯ NA<br>(This notably applies to prognosis studies and diagnostic studies with follow-up as diagnostic outcome. If this is not applicable for an article (i.e. diagnostic study or no follow-up), then score Not applicable.)
If other, please specify	__________________________________<br>(list using (;) to separate if more than 1)
If other, please specify	__________________________________<br>(list using (;) to separate if more than 1)
If other, please specify	__________________________________<br>(list using (;) to separate if more than 1)
Development<br>For time-to-event endpoints, do the authors report how many have X-years of follow-up?	◯ Yes<br>◯ No<br>◯ NA
External validation<br>For time-to-event endpoints, do the authors report how many have X-years of follow-up?	◯ Yes<br>◯ No<br>◯ NA
Development<br>Basic demographics are reported	◯ Yes<br>◯ No<br>(At least age and sex are reported.)
External validation<br>Basic demographics are reported	◯ Yes<br>◯ No<br>(At least age and sex are reported.)
External validation<br>Basic demographics are reported	◯ Yes<br>◯ No<br>(At least age and sex are reported.)
Development<br>Number of predictors in the final model	__________________________________<br>(If the final predictors are unclear, please fill this question with a 'UN')

Andaur Navarro CL, *et al. BMJ Open* 2020; 10:e038832. doi: 10.1136/bmjopen-2020-038832

Confidential

External validation<br>Number of predictors in the final model	__________________________________<br>(If the final predictors are unclear, please fill this question with a 'UN')
External validation<br>Number of predictors in the final model	__________________________________<br>(If the final predictors are unclear, please fill this question with a 'UN')
Development<br>Summary information is provided for all predictors included in the final developed/validated model	◯ Yes<br>◯ No
External validation<br>Summary information is provided for all predictors included in the final developed/validated model	◯ Yes<br>◯ No
External validation<br>Summary information is provided for all predictors included in the final developed/validated model	◯ Yes<br>◯ No
Development<br>The number of participants with missing data for predictors is reported	◯ Yes<br>◯ No<br>◯ NA<br>(When no missing values is reported, fill this with "NA")
External validation<br>The number of participants with missing data for predictors is reported	◯ Yes<br>◯ No<br>◯ NA<br>(When no missing values is reported, fill this with "NA")
External validation<br>The number of participants with missing data for predictors is reported	◯ Yes<br>◯ No<br>◯ NA<br>(When no missing values is reported, fill this with "NA")
Development<br>Final number of models developed/validated reported	__________________________________<br>(Please provide the number. If this is unclear, please fill this with "UN")
External validation<br>Final number of models developed/validated reported	__________________________________<br>(Please provide the number. If this is unclear, please fill this with "UN")
External validation<br>Final number of models developed/validated reported	__________________________________<br>(Please provide the number. If this is unclear, please fill this with "UN")

External validation<br>Demographic characteristics (at least age and gender) of the validation study participants are reported along with those of the original development study	◯ Yes<br>◯ No<br>◯ NA<br>(For incremental value reports, in case additional predictors are not added to a previously developed prediction model but rather added to conventional predictors in a newly fitted model, score Not applicable.)
External validation<br>Distributions of predictors in the model of the validation study participants are reported along with those of the original development study	◯ Yes<br>◯ No<br>◯ NA<br>(For incremental value reports, in case additional predictors are not added to a previously developed prediction model but rather added to conventional predictors in a newly fitted model, score Not applicable.)
External validation<br>Outcomes of the validation study participants are reported along with those of the original development study	◯ Yes<br>◯ No<br>◯ NA<br>(For incremental value reports, in case additional predictors are not added to a previously developed prediction model but rather added to conventional predictors in a newly fitted model, score Not applicable)

Model development

The number of participants in each analysis is specified	◯ Yes<br>◯ No<br>(e.g. in the analysis of each model if more than one model is developed)
What is the number of participants (included in the analysis) reported in the main model?	________________________________<br>(If the number of participants is not reported, please fill this with 'NR'.)
The number of outcome events in each analysis is specified	◯ Yes<br>◯ No<br>◯ NA<br>(e.g. in the analysis of each model if more than one model is developed. If outcomes are continuous, score Not applicable.)
What is the number of events (initial) reported in the main outcome?	________________________________<br>(If the number of events is not reported, please fill this with 'NR'.)
What is the number of events (included in the analysis) reported in the main model?	________________________________<br>(If the number of events is not reported, please fill this with 'NR'.)

Confidential

The unadjusted associations between each predictor and outcome are reported	◯ Yes<br>◯ No<br>◯ NA<br>(If any univariable analysis is mentioned in the methods but not in the results, score No. If nothing on univariable analysis (in methods or results) is reported, score this item as Not applicable)

Model specification

Development<br>The regression coefficient (or a derivative such as hazard ratio, odds ratio, risk ratio) for each predictor in the model is reported	◯ Yes<br>◯ No<br>◯ NA
Development<br>The intercept or the cumulative baseline hazard (or baseline survival) for at least one time point is reported	◯ Yes<br>◯ No<br>◯ NA
Development<br>An explanation (e.g. a simplified scoring rule, chart, nomogram of the model, reference to online calculator, or worked example) is provided to explain how to use the model for individualised predictions.	◯ Yes<br>◯ No
Development<br>Is there enough information to calculate the risk of the outcome in a new individual?	◯ Yes<br>◯ No

Model Performance

Instructions

-Please extract the models in the order they are presented in the article.

-If more than 10 models were developed for the main outcome, only refer to the first 10.

-If a comparison with logistic regression was made, please include this model in the final count and extract information.

How many models were developed for the primary outcome?	________________________<br>(This should reflect the number of models you are going to extract on - primary outcome and primary timepoint. If more than 10 models were developed, please refer to the first 10 models. If a Logistic regression model was performed, please also extract data from this model)

Andaur Navarro CL, *et al. BMJ Open* 2020; 10:e038832. doi: 10.1136/bmjopen-2020-038832

**Model 1**	
Is this the recommended model?	◯ Yes<br>◯ No<br>(If there is only one model, please state 'Yes')
Development<br>A discrimination measure is presented	◯ Yes<br>◯ No<br>(E.g. C-index / area under the ROC curve)
External validation<br>A discrimination measure is presented	◯ Yes<br>◯ No<br>(E.g. C-index / area under the ROC curve)
Development<br>Which discrimination measures are described?	☐ AUC/AUROC/Area under the curve<br>☐ C-statistic<br>☐ Harrell's c-index<br>☐ D-statistic
External validation<br>Which discrimination measures are described?	☐ AUC/AUROC/Area under the curve<br>☐ C-statistic<br>☐ Harrell's c-index<br>☐ D-statistic
The confidence interval (or standard error) of the AUC/AUROC/Area under the curve is presented	◯ Yes<br>◯ No
The confidence interval (or standard error) of the AUC/AUROC/Area under the curve is presented	◯ Yes<br>◯ No
What was the AUC/AUROC/Area under the curve apparent discrimination estimate (+95% CI, if given)?	__________________________________<br>(Please use the following format - estimate (lower CI - upper CI). For example 0.79 (0.75 - 0.81). If not reported fill this with "NR")
What was the AUC/AUROC/Area under the curve apparent discrimination estimate (+95% CI, if given)?	__________________________________<br>(Please use the following format - estimate (lower CI - upper CI). For example 0.79 (0.75 - 0.81). If not reported fill this with "NR")
Was the AUC/AUROC/Area under the curve corrected for optimism?	◯ Yes<br>◯ No<br>◯ Unclear
What was the optimism corrected AUC/AUROC/Area under the curve (+95% CI, if given)?	__________________________________<br>(Please use the following format - estimate (lower CI - upper CI). For example 0.79 (0.75 - 0.81). If not reported fill this with "NR")
The confidence interval (or standard error) of the C-statistic is presented	◯ Yes<br>◯ No
The confidence interval (or standard error) of the C-statistic is presented	◯ Yes<br>◯ No

Confidential

What was the C-statistic apparent discrimination estimate (+95% CI, if given)?	__________________ (Please use the following format - estimate (lower CI - upper CI). For example 0.79 (0.75 - 0.81). If not reported fill this with "NR")
What was the C-statistic apparent discrimination estimate (+95% CI, if given)?	__________________ (Please use the following format - estimate (lower CI - upper CI). For example 0.79 (0.75 - 0.81). If not reported fill this with "NR")
Was the C-statistic corrected for optimism?	◯ Yes<br>◯ No<br>◯ Unclear
What was the optimism corrected C-statistic (+95% CI, if given)?	__________________ (Please use the following format - estimate (lower CI - upper CI). For example 0.79 (0.75 - 0.81). If not reported fill this with "NR")
The confidence interval (or standard error) of the Harrell's c-index is presented	◯ Yes<br>◯ No
The confidence interval (or standard error) of the Harrell's c-index is presented	◯ Yes<br>◯ No
What was the Harrell's c-index apparent discrimination estimate (+95% CI, if given)?	__________________ (Please use the following format - estimate (lower CI - upper CI). For example 0.79 (0.75 - 0.81). If not reported fill this with "NR")
What was the Harrell's c-index apparent discrimination estimate (+95% CI, if given)?	__________________ (Please use the following format - estimate (lower CI - upper CI). For example 0.79 (0.75 - 0.81). If not reported fill this with "NR")
Was the Harrell's c-index corrected for optimism?	◯ Yes<br>◯ No<br>◯ Unclear
What was the optimism corrected Harrell's c-index (+95% CI, if given)?	__________________ (Please use the following format - estimate (lower CI - upper CI). For example 0.79 (0.75 - 0.81). If not reported fill this with "NR")
The confidence interval (or standard error) of the d-statistic presented	◯ Yes<br>◯ No
The confidence interval (or standard error) of the d-statistic presented	◯ Yes<br>◯ No

Confidential

What was the d-statistic apparent discrimination estimate (+95% CI, if given)?	__________________ (Please use the following format - estimate (lower CI - upper CI). For example 0.79 (0.75 - 0.81). If not reported fill this with "NR")
What was the d-statistic apparent discrimination estimate (+95% CI, if given)?	__________________ (Please use the following format - estimate (lower CI - upper CI). For example 0.79 (0.75 - 0.81). If not reported fill this with "NR")
Was the d-statistic corrected for optimism?	◯ Yes<br>◯ No<br>◯ Unclear
What was the optimism corrected d-statistic (+95% CI, if given)?	__________________ (Please use the following format - estimate (lower CI - upper CI). For example 0.79 (0.75 - 0.81). If not reported fill this with "NR")
Development<br>Measures for model calibration are described	◯ Yes<br>◯ No<br>(E.g. calibration plot, calibration slope or intercept, calibration table, Hosmer Lemeshow test, O/E ratio.)
External validation<br>Measures for model calibration are described	◯ Yes<br>◯ No<br>(E.g. calibration plot, calibration slope or intercept, calibration table, Hosmer Lemeshow test, O/E ratio.)
Development<br>How was calibration assessed?	☐ Calibration plot<br>☐ Calibration slope<br>☐ Calibration intercept<br>☐ Calibration in the large<br>☐ Calibration table<br>☐ Kappa<br>☐ Observed/expected ratio<br>☐ H-L
External validation<br>How was calibration assessed?	☐ Calibration plot<br>☐ Calibration slope<br>☐ Calibration intercept<br>☐ Calibration in the large<br>☐ Calibration table<br>☐ Kappa<br>☐ Observed/expected ratio<br>☐ H-L
What was the apparent [gen_methods_103:checked] estimate (+95% CI, if given)?	__________________ (list using (;) to separate if more than 1. If not reported, fill this with "NR")

Confidential

What was the apparent [gen_methods_117:checked] estimate (+95% CI, if given)?	__________________________________<br>(list using (;) to separate if more than 1. If not reported, fill this with "NR")
Was the [gen_methods_103:checked] measure corrected for optimism?	◯ Yes<br>◯ No<br>◯ Unclear
What was the optimism corrected [gen_methods_103:checked] estimate (+95% CI, if given)?	__________________________________<br>(list using (;) to separate if more than 1. If measures were corrected for optimism but not reported, please fill this with "NR".)
Development<br>Other model performance measures are presented	◯ Yes<br>◯ No<br>(E.g. R2, Brier score, predictive values, sensitivity, specificity, AUC difference, decision curve analysis, net reclassification improvement, integrated discrimination improvement, AIC.)
External validation<br>Other model performance measures are presented	◯ Yes<br>◯ No<br>(E.g. R2, Brier score, predictive values, sensitivity, specificity, AUC difference, decision curve analysis, net reclassification improvement, integrated discrimination improvement, AIC.)
Development<br>If yes, please specify	☐ R2<br>☐ Brier score<br>☐ predictive values<br>☐ sensitivity<br>☐ specificity<br>☐ AUC difference<br>☐ decision curve analysis<br>☐ net reclassification improvement<br>☐ integrated discrimination improvement<br>☐ AIC<br>☐ Accuracy<br>☐ Other<br>(Multiples answers are possible)
External validation<br>If yes, please specify	☐ R2<br>☐ Brier score<br>☐ predictive values<br>☐ sensitivity<br>☐ specificity<br>☐ AUC difference<br>☐ decision curve analysis<br>☐ net reclassification improvement<br>☐ integrated discrimination improvement<br>☐ AIC<br>☐ Accuracy<br>☐ Other<br>(Multiples answers are possible)

Andaur Navarro CL, *et al. BMJ Open* 2020; 10:e038832. doi: 10.1136/bmjopen-2020-038832

If other, please specify	__________________ (list the names using (;) to separate if more than 1)
If other, please specify	__________________ (list the names using (;) to separate if more than 1)
What was the apparent [gen_methods_104:checked] estimate (+95% CI, if given)?	__________________ (list using (;) to separate if more than 1. If not reported, fill this with "NR")
What was the apparent [gen_methods_118:checked] estimate (+95% CI, if given)?	__________________ (list using (;) to separate if more than 1. If not reported, fill this with "NR")
Was the [gen_methods_104:checked] measure corrected for optimism?	◯ Yes<br>◯ No<br>◯ Unclear
What was the optimism corrected [gen_methods_104:checked] estimate (+95% CI, if given)?	__________________ (list using (;) to separate if more than 1. If not reported, fill this with "NR")

Model 2

Is this the recommended model?	◯ Yes<br>◯ No<br>(If there is only one model, please state 'Yes')
Development<br>A discrimination measure is presented	◯ Yes<br>◯ No<br>(E.g. C-index / area under the ROC curve)
External validation<br>A discrimination measure is presented	◯ Yes<br>◯ No<br>(E.g. C-index / area under the ROC curve)
Development<br>Which performance measures are described?	☐ AUC/AUROC/Area under the curve<br>☐ C-statistic<br>☐ Harrell's c-index<br>☐ D-statistic<br>(Multiples answers are possible)
External validation<br>Which performance measures are described?	☐ AUC/AUROC/Area under the curve<br>☐ C-statistic<br>☐ Harrell's c-index<br>☐ D-statistic<br>(Multiples answers are possible)

Andaur Navarro CL, *et al. BMJ Open* 2020; 10:e038832. doi: 10.1136/bmjopen-2020-038832

The confidence interval (or standard error) of the AUC/AUROC/Area under the curve is presented	◯ Yes<br>◯ No
The confidence interval (or standard error) of the AUC/AUROC/Area under the curve is presented	◯ Yes<br>◯ No
What was the AUC/AUROC/Area under the curve apparent discrimination estimate (+95%CI, if given)?	__________________________<br>(Please use the following format - estimate (lower CI - upper CI). For example 0.79 (0.75 - 0.81). If not reported, fill this with "NR")
What was the AUC/AUROC/Area under the curve apparent discrimination estimate (+95%CI, if given)?	__________________________<br>(Please use the following format - estimate (lower CI - upper CI). For example 0.79 (0.75 - 0.81). If not reported fill this with "NR")
Was the AUC/AUROC/Area under the curve corrected for optimism?	◯ Yes<br>◯ No<br>◯ Unclear
What was the optimism corrected AUC/AUROC/Area under the curve (95% CI, if given)?	__________________________<br>(Please use the following format - estimate (lower CI - upper CI). For example 0.79 (0.75 - 0.81). If not reported fill this with "NR")
The confidence interval (or standard error) of the C-statistics is presented	◯ Yes<br>◯ No
The confidence interval (or standard error) of the C-statistics is presented	◯ Yes<br>◯ No
What was the C-statistics apparent discrimination estimate (+95%CI, if given)?	__________________________<br>(Please use the following format - estimate (lower CI - upper CI). For example 0.79 (0.75 - 0.81). If not reported fill this with "NR")
What was the C-statistics apparent discrimination estimate (+95%CI, if given)?	__________________________<br>(Please use the following format - estimate (lower CI - upper CI). For example 0.79 (0.75 - 0.81). If not reported fill this with "NR")
Was the C-statistics corrected for optimism?	◯ Yes<br>◯ No<br>◯ Unclear
What was the optimism corrected C-statistic (95% CI, if given)?	__________________________<br>(Please use the following format - estimate (lower CI - upper CI). For example 0.79 (0.75 - 0.81). If not reported fill this with "NR")
The confidence interval (or standard error) of the Harrel's c-index is presented	◯ Yes<br>◯ No

The confidence interval (or standard error) of the Harrel's c-index is presented	◯ Yes<br>◯ No
What was the Harrel's c-index apparent discrimination estimate (+95%CI, if given)?	__________________________________<br>(Please use the following format - estimate (lower CI - upper CI). For example 0.79 (0.75 - 0.81). If not reported fill this with "NR")
What was the Harrel's c-index apparent discrimination estimate (+95%CI, if given)?	__________________________________<br>(Please use the following format - estimate (lower CI - upper CI). For example 0.79 (0.75 - 0.81). If not reported fill this with "NR")
Was the Harrel's c-index corrected for optimism?	◯ Yes<br>◯ No<br>◯ Unclear
What was the optimism corrected Harrel's c-index (95% CI, if given)?	__________________________________<br>(Please use the following format - estimate (lower CI - upper CI). For example 0.79 (0.75 - 0.81). If not reported fill this with "NR")
The confidence interval (or standard error) of the D-statistic is presented	◯ Yes<br>◯ No
The confidence interval (or standard error) of the D-statistic is presented	◯ Yes<br>◯ No
What was the D-statistics apparent discrimination estimate (+95%CI, if given)?	__________________________________<br>(Please use the following format - estimate (lower CI - upper CI). For example 0.79 (0.75 - 0.81). If not reported fill this with "NR")
What was the D-statistics apparent discrimination estimate (+95%CI, if given)?	__________________________________<br>(Please use the following format - estimate (lower CI - upper CI). For example 0.79 (0.75 - 0.81). If not reported fill this with "NR")
Was the D-statistics corrected for optimism?	◯ Yes<br>◯ No<br>◯ Unclear
What was the optimism corrected D-statistics (95% CI, if given)?	__________________________________<br>(Please use the following format - estimate (lower CI - upper CI). For example 0.79 (0.75 - 0.81). If not reported fill this with "NR")
Development<br>Measures for model calibration are described	◯ Yes<br>◯ No<br>(E.g. calibration plot, calibration slope or intercept, calibration table, Hosmer Lemeshow test, O/E ratio.)

Confidential

External validation<br>Measures for model calibration are described	◯ Yes<br>◯ No<br>(E.g. calibration plot, calibration slope or intercept, calibration table, Hosmer Lemeshow test, O/E ratio.)
Development<br>How was calibration assessed?	☐ Calibration plot<br>☐ Calibration slope<br>☐ Calibration intercept<br>☐ Calibration in the large<br>☐ Calibration table<br>☐ Kappa<br>☐ Observed/expected ratio<br>☐ H-L
External validation<br>How was calibration assessed?	☐ Calibration plot<br>☐ Calibration slope<br>☐ Calibration intercept<br>☐ Calibration in the large<br>☐ Calibration table<br>☐ Kappa<br>☐ Observed/expected ratio<br>☐ H-L
What was the apparent [gen_methods_119:checked] estimate (+95% CI, if given)?	__________________________________<br>(list using (;) to separate if more than 1. If not reported, fill this with "NR")
What was the apparent [gen_methods_120:checked] estimate (+95% CI, if given)?	__________________________________<br>(list using (;) to separate if more than 1. If not reported, fill this with "NR")
Was the [gen_methods_119:checked] measure corrected for optimism?	◯ Yes<br>◯ No<br>◯ Unclear
What was the optimism corrected [gen_methods_119:checked] estimate (+95% CI, if given)?	__________________________________<br>(list using (;) to separate if more than 1. If not reported, fill this with "NR")
Development<br>Other model performance measures are presented	◯ Yes<br>◯ No<br>(E.g. R2, Brier score, predictive values, sensitivity, specificity, AUC difference, decision curve analysis, net reclassification improvement, integrated discrimination improvement, AIC.)
External validation<br>Other model performance measures are presented	◯ Yes<br>◯ No<br>(E.g. R2, Brier score, predictive values, sensitivity, specificity, AUC difference, decision curve analysis, net reclassification improvement, integrated discrimination improvement, AIC.)

Confidential

Development<br>If yes, please specify	☐ R2<br>☐ Brier score<br>☐ predictive values<br>☐ sensitivity<br>☐ specificity<br>☐ AUC difference<br>☐ decision curve analysis<br>☐ net reclassification improvement<br>☐ integrated discrimination improvement<br>☐ AIC<br>☐ Accuracy<br>☐ Other<br>(Multiples answers are possible)
External validation<br>If yes, please specify	☐ R2<br>☐ Brier score<br>☐ predictive values<br>☐ sensitivity<br>☐ specificity<br>☐ AUC difference<br>☐ decision curve analysis<br>☐ net reclassification improvement<br>☐ integrated discrimination improvement<br>☐ AIC<br>☐ Accuracy<br>☐ Other<br>(Multiples answers are possible)
If other, please specify	______________________<br>(list using (;) to separate if more than 1)
If other, please specify	______________________<br>(list using (;) to separate if more than 1)
What was the apparent [gen_methods_121:checked] estimate (+95% CI, if given)?	______________________<br>(list using (;) to separate if more than 1. If not reported, fill this with "NR")
What was the apparent [gen_methods_122:checked] estimate (+95% CI, if given)?	______________________<br>(list using (;) to separate if more than 1. If not reported, fill this with "NR")
Was the [gen_methods_121:checked] measure corrected for optimism?	◯ Yes<br>◯ No<br>◯ Unclear
What was the optimism corrected [gen_methods_121:checked] estimate (+95% CI, if given)?	______________________<br>(list using (;) to separate if more than 1. If not reported, fill this with "NR")

Model 3

Is this the recommended model?	◯ Yes<br>◯ No<br>(If there is only one model, please state 'Yes')
Development<br>A discrimination measure is presented	◯ Yes<br>◯ No<br>(E.g. C-index / area under the ROC curve)
External validation<br>A discrimination measure is presented	◯ Yes<br>◯ No<br>(E.g. C-index / area under the ROC curve)
Development<br>Which performance measures are described?	☐ AUC/AUROC/Area under the curve<br>☐ C-statistic<br>☐ Harrell's c-index<br>☐ D-statistic<br>(Multiples answers are possible)
External validation<br>Which performance measures are described?	☐ AUC/AUROC/Area under the curve<br>☐ C-statistic<br>☐ Harrell's c-index<br>☐ D-statistic<br>(Multiples answers are possible)
The confidence interval (or standard error) of the AUC/AUROC/Area under the curve is presented	◯ Yes<br>◯ No
The confidence interval (or standard error) of the AUC/AUROC/Area under the curve is presented	◯ Yes<br>◯ No
What was the AUC/AUROC/Area under the curve apparent discrimination estimate (+95%CI, if given)?	______________________________<br>(Please use the following format - estimate (lower CI - upper CI). For example 0.79 (0.75 - 0.81). If not reported fill this with "NR")
What was the AUC/AUROC/Area under the curve apparent discrimination estimate (+95%CI, if given)?	______________________________<br>(Please use the following format - estimate (lower CI - upper CI). For example 0.79 (0.75 - 0.81). If not reported fill this with "NR")
Was the AUC/AUROC/Area under the curve corrected for optimism?	◯ Yes<br>◯ No<br>◯ Unclear
What was the optimism corrected AUC/AUROC/Area under the curve (95% CI, if given)?	______________________________<br>(Please use the following format - estimate (lower CI - upper CI). For example 0.79 (0.75 - 0.81). If not reported fill this with "NR")
The confidence interval (or standard error) of the C-statistics is presented	◯ Yes<br>◯ No

The confidence interval (or standard error) of the C-statistics is presented	◯ Yes<br>◯ No
What was the C-statistics apparent discrimination estimate (+95%CI, if given)?	__________________________________<br>(Please use the following format - estimate (lower CI - upper CI). For example 0.79 (0.75 - 0.81). If not reported fill this with "NR")
What was the C-statistics apparent discrimination estimate (+95%CI, if given)?	__________________________________<br>(Please use the following format - estimate (lower CI - upper CI). For example 0.79 (0.75 - 0.81). If not reported fill this with "NR")
Was the C-statistics corrected for optimism?	◯ Yes<br>◯ No<br>◯ Unclear
What was the optimism corrected C-statistics (95% CI, if given)?	__________________________________<br>(Please use the following format - estimate (lower CI - upper CI). For example 0.79 (0.75 - 0.81). If not reported fill this with "NR")
The confidence interval (or standard error) of the Harrel's c-index is presented	◯ Yes<br>◯ No
The confidence interval (or standard error) of the Harrel's c-index is presented	◯ Yes<br>◯ No
What was the Harrel's c-index apparent discrimination estimate (+95%CI, if given)?	__________________________________<br>(Please use the following format - estimate (lower CI - upper CI). For example 0.79 (0.75 - 0.81). If not reported fill this with "NR")
What was the Harrel's c-index apparent discrimination estimate (+95%CI, if given)?	__________________________________<br>(Please use the following format - estimate (lower CI - upper CI). For example 0.79 (0.75 - 0.81). If not reported fill this with "NR")
Was the Harrel's c-index corrected for optimism?	◯ Yes<br>◯ No<br>◯ Unclear
What was the optimism corrected Harrel's c-index (95% CI, if given)?	__________________________________<br>(Please use the following format - estimate (lower CI - upper CI). For example 0.79 (0.75 - 0.81). If not reported fill this with "NR")
The confidence interval (or standard error) of the D-statistics is presented	◯ Yes<br>◯ No

The confidence interval (or standard error) of the D-statistics is presented	◯ Yes<br>◯ No
What was the D-statistics apparent discrimination estimate (+95%CI, if given)?	__________________________________<br>(Please use the following format - estimate (lower CI - upper CI). For example 0.79 (0.75 - 0.81). If not reported fill this with "NR")
What was the D-statistics apparent discrimination estimate (+95%CI, if given)?	__________________________________<br>(Please use the following format - estimate (lower CI - upper CI). For example 0.79 (0.75 - 0.81). If not reported fill this with "NR")
Was the D-statistics corrected for optimism?	◯ Yes<br>◯ No<br>◯ Unclear
What was the optimism corrected D-statistics (95% CI, if given)?	__________________________________<br>(Please use the following format - estimate (lower CI - upper CI). For example 0.79 (0.75 - 0.81). If not reported fill this with "NR")
Development<br>Measures for model calibration are described	◯ Yes<br>◯ No<br>(E.g. calibration plot, calibration slope or intercept, calibration table, Hosmer Lemeshow test, O/E ratio.)
External validation<br>Measures for model calibration are described	◯ Yes<br>◯ No<br>(E.g. calibration plot, calibration slope or intercept, calibration table, Hosmer Lemeshow test, O/E ratio.)
Development<br>How was calibration assessed?	☐ Calibration plot<br>☐ Calibration slope<br>☐ Calibration intercept<br>☐ Calibration in the large<br>☐ Calibration table<br>☐ Kappa<br>☐ Observed/expected ratio<br>☐ H-L
External validation<br>How was calibration assessed?	☐ Calibration plot<br>☐ Calibration slope<br>☐ Calibration intercept<br>☐ Calibration in the large<br>☐ Calibration table<br>☐ Kappa<br>☐ Observed/expected ratio<br>☐ H-L

What was the apparent [gen_methods_139:checked] estimate (+95% CI, if given)?	__________________________ (Please use the following format - estimate (lower CI - upper CI). For example 0.79 (0.75 - 0.81). List using (;) if more than 1. If not reported, fill this with "NR")
What was the apparent [gen_methods_140:checked] estimate (+95% CI, if given)?	__________________________ (Please use the following format - estimate (lower CI - upper CI). For example 0.79 (0.75 - 0.81). List using (;) if more than 1. If not reported, fill this with "NR")
Was the [gen_methods_139:checked] measure corrected for optimism?	◯ Yes<br>◯ No<br>◯ Unclear
What was the optimism corrected [gen_methods_139:checked] estimate (+95% CI, if given)?	__________________________ (Please use the following format - estimate (lower CI - upper CI). For example 0.79 (0.75 - 0.81). List using (;) if more than 1. If not reported, fill this with "NR")
Development<br>Other model performance measures are presented	◯ Yes<br>◯ No<br>(E.g. R2, Brier score, predictive values, sensitivity, specificity, AUC difference, decision curve analysis, net reclassification improvement, integrated discrimination improvement, AIC.)
External validation<br>Other model performance measures are presented	◯ Yes<br>◯ No<br>(E.g. R2, Brier score, predictive values, sensitivity, specificity, AUC difference, decision curve analysis, net reclassification improvement, integrated discrimination improvement, AIC.)
Development<br>If yes, please specify	☐ R2<br>☐ Brier score<br>☐ predictive values<br>☐ sensitivity<br>☐ specificity<br>☐ AUC difference<br>☐ decision curve analysis<br>☐ net reclassification improvement<br>☐ integrated discrimination improvement<br>☐ AIC<br>☐ Accuracy<br>☐ Other<br>(Multiples answers are possible)

External validation<br>If yes, please specify	☐ R2<br>☐ Brier score<br>☐ predictive values<br>☐ sensitivity<br>☐ specificity<br>☐ AUC difference<br>☐ decision curve analysis<br>☐ net reclassification improvement<br>☐ integrated discrimination improvement<br>☐ AIC<br>☐ Accuracy<br>☐ Other<br>(Multiples answers are possible)
If other, please specify	__________________________________<br>(list using (;) to separate if more than 1)
If other, please specify	__________________________________<br>(list using (;) to separate if more than 1)
What was the apparent [gen_methods_123:checked] estimate (+95% CI, if given)?	__________________________________<br>(list using (;) to separate if more than 1. If not reported, fill this with "NR")
What was the apparent [gen_methods_123:checked] estimate (+95% CI, if given)?	__________________________________<br>(list using (;) to separate if more than 1. If not reported, fill this with "NR")
Was the [gen_methods_123:checked] measure corrected for optimism?	◯ Yes<br>◯ No<br>◯ Unclear
What was the optimism corrected [gen_methods_123:checked] estimate (+95% CI, if given)?	__________________________________<br>(list using (;) to separate if more than 1. If measures were corrected for optimism but not reported, please fill this with "NR".)

Model 4

Is this the recommended model?	◯ Yes<br>◯ No<br>(If there is only one model, please state 'Yes')
Development<br>A discrimination measure is presented	◯ Yes<br>◯ No<br>(E.g. C-index / area under the ROC curve)
External validation<br>A discrimination measure is presented	◯ Yes<br>◯ No<br>(E.g. C-index / area under the ROC curve)

Development<br>Which performance measures are described?	☐ AUC/AUROC/Area under the curve<br>☐ C-statistic<br>☐ Harrell's c-index<br>☐ D-statistic<br>(Multiples answers are possible)
External validation<br>Which performance measures are described?	☐ AUC/AUROC/Area under the curve<br>☐ C-statistic<br>☐ Harrell's c-index<br>☐ D-statistic<br>(Multiples answers are possible)
The confidence interval (or standard error) of the AUC/AUROC/Area under the curve is presented	◯ Yes<br>◯ No
The confidence interval (or standard error) of the AUC/AUROC/Area under the curve is presented	◯ Yes<br>◯ No
What was the AUC/AUROC/Area under the curve apparent discrimination estimate (+95%CI, if given)?	________________________<br>(Please use the following format - estimate (lower CI - upper CI). For example 0.79 (0.75 - 0.81). If not reported fill this with "NR")
What was the AUC/AUROC/Area under the curve apparent discrimination estimate (+95%CI, if given)?	________________________<br>(Please use the following format - estimate (lower CI - upper CI). For example 0.79 (0.75 - 0.81). If not reported fill this with "NR")
Was the AUC/AUROC/Area under the curve corrected for optimism?	◯ Yes<br>◯ No<br>◯ Unclear
What was the optimism corrected AUC/AUROC/Area under the curve (95% CI, if given)?	________________________<br>(Please use the following format - estimate (lower CI - upper CI). For example 0.79 (0.75 - 0.81). If not reported fill this with "NR")
The confidence interval (or standard error) of the C-statistics is presented	◯ Yes<br>◯ No
The confidence interval (or standard error) of the C-statistics is presented	◯ Yes<br>◯ No
What was the C-statistics apparent discrimination estimate (+95%CI, if given)?	________________________<br>(Please use the following format - estimate (lower CI - upper CI). For example 0.79 (0.75 - 0.81). If not reported fill this with "NR")

What was the C-statistics apparent discrimination estimate (+95%CI, if given)?	____________________________________ (Please use the following format - estimate (lower CI - upper CI). For example 0.79 (0.75 - 0.81). If not reported fill this with "NR")
Was the C-statistics corrected for optimism?	◯ Yes<br>◯ No<br>◯ Unclear
What was the optimism corrected C-statistics (95% CI, if given)?	____________________________________ (Please use the following format - estimate (lower CI - upper CI). For example 0.79 (0.75 - 0.81). If not reported fill this with "NR")
The confidence interval (or standard error) of the Harrel's c-index is presented	◯ Yes<br>◯ No
The confidence interval (or standard error) of the Harrel's c-index is presented	◯ Yes<br>◯ No
What was the Harrel's c-index apparent discrimination estimate (+95%CI, if given)?	____________________________________ (Please use the following format - estimate (lower CI - upper CI). For example 0.79 (0.75 - 0.81). If not reported fill this with "NR")
What was the Harrel's c-index apparent discrimination estimate (+95%CI, if given)?	____________________________________ (Please use the following format - estimate (lower CI - upper CI). For example 0.79 (0.75 - 0.81). If not reported fill this with "NR")
Was the Harrel's c-index corrected for optimism?	◯ Yes<br>◯ No<br>◯ Unclear
What was the optimism corrected Harrel's c-index (95% CI, if given)?	____________________________________ (Please use the following format - estimate (lower CI - upper CI). For example 0.79 (0.75 - 0.81). If not reported fill this with "NR")
The confidence interval (or standard error) of the D-statistic is presented	◯ Yes<br>◯ No
The confidence interval (or standard error) of the D-statistic is presented	◯ Yes<br>◯ No
What was the D-statistic apparent discrimination estimate (+95%CI, if given)?	____________________________________ (Please use the following format - estimate (lower CI - upper CI). For example 0.79 (0.75 - 0.81). If not reported fill this with "NR")

Confidential

What was the D-statistic apparent discrimination estimate (+95%CI, if given)?	______________________________ (Please use the following format - estimate (lower CI - upper CI). For example 0.79 (0.75 - 0.81). If not reported fill this with "NR")
Was the D-statistic corrected for optimism?	◯ Yes<br>◯ No<br>◯ Unclear
What was the optimism corrected D-statistic (95% CI, if given)?	______________________________ (Please use the following format - estimate (lower CI - upper CI). For example 0.79 (0.75 - 0.81). If not reported fill this with "NR")
Development<br>Measures for model calibration are described	◯ Yes<br>◯ No<br>(E.g. calibration plot, calibration slope or intercept, calibration table, Hosmer Lemeshow test, O/E ratio.)
External validation<br>Measures for model calibration are described	◯ Yes<br>◯ No<br>(E.g. calibration plot, calibration slope or intercept, calibration table, Hosmer Lemeshow test, O/E ratio.)
Development<br>How was calibration assessed?	☐ Calibration plot<br>☐ Calibration slope<br>☐ Calibration intercept<br>☐ Calibration in the large<br>☐ Calibration table<br>☐ Kappa<br>☐ Observed/expected ratio<br>☐ H-L
External validation<br>How was calibration assessed?	☐ Calibration plot<br>☐ Calibration slope<br>☐ Calibration intercept<br>☐ Calibration in the large<br>☐ Calibration table<br>☐ Kappa<br>☐ Observed/expected ratio<br>☐ H-L
What was the apparent [gen_methods_141:checked] estimate (+95% CI, if given)?	______________________________ (Please use the following format - estimate (lower CI - upper CI). For example 0.79 (0.75 - 0.81). List using (;) if more than 1. If not reported, fill this with "NR")
What was the apparent [gen_methods_142:checked] estimate (+95% CI, if given)?	______________________________ (Please use the following format - estimate (lower CI - upper CI). For example 0.79 (0.75 - 0.81). List using (;) if more than 1. If not reported, fill this with "NR")

Andaur Navarro CL, *et al. BMJ Open* 2020; 10:e038832. doi: 10.1136/bmjopen-2020-038832

Was the [gen_methods_141:checked] measure corrected for optimism?	◯ Yes<br>◯ No<br>◯ Unclear
What was the optimism corrected [gen_methods_141:checked] estimate (+95% CI, if given)?	______________________________<br>(Please use the following format - estimate (lower CI - upper CI). For example 0.79 (0.75 - 0.81). List using (;) if more than 1. If not reported, fill this with "NR")
Development<br>Other model performance measures are presented	◯ Yes<br>◯ No<br>(E.g. R2, Brier score, predictive values, sensitivity, specificity, AUC difference, decision curve analysis, net reclassification improvement, integrated discrimination improvement, AIC.)
External validation<br>Other model performance measures are presented	◯ Yes<br>◯ No<br>(E.g. R2, Brier score, predictive values, sensitivity, specificity, AUC difference, decision curve analysis, net reclassification improvement, integrated discrimination improvement, AIC.)
Development<br>If yes, please specify	☐ R2<br>☐ Brier score<br>☐ predictive values<br>☐ sensitivity<br>☐ specificity<br>☐ AUC difference<br>☐ decision curve analysis<br>☐ net reclassification improvement<br>☐ integrated discrimination improvement<br>☐ AIC<br>☐ Accuracy<br>☐ Other<br>(Multiples answers are possible)
External validation<br>If yes, please specify	☐ R2<br>☐ Brier score<br>☐ predictive values<br>☐ sensitivity<br>☐ specificity<br>☐ AUC difference<br>☐ decision curve analysis<br>☐ net reclassification improvement<br>☐ integrated discrimination improvement<br>☐ AIC<br>☐ Accuracy<br>☐ Other<br>(Multiples answers are possible)
If other, please specify	______________________________<br>(list using (;) to separate if more than 1.)

If other, please specify	__________________________________<br>(list using (;) to separate if more than 1.)
What was the apparent [gen_methods_125:checked] estimate (+95% CI, if given)?	__________________________________<br>(Please use the following format - estimate (lower CI - upper CI). For example 0.79 (0.75 - 0.81). List using (;) if more than 1. If not reported, fill this with "NR")
What was the apparent [gen_methods_126:checked] estimate (+95% CI, if given)?	__________________________________<br>(Please use the following format - estimate (lower CI - upper CI). For example 0.79 (0.75 - 0.81). List using (;) if more than 1. If not reported, fill this with "NR")
Was the [gen_methods_125:checked] measure corrected for optimism?	◯ Yes<br>◯ No<br>◯ Unclear
What was the optimism corrected [gen_methods_125:checked] estimate (+95% CI, if given)?	__________________________________<br>(Please use the following format - estimate (lower CI - upper CI). For example 0.79 (0.75 - 0.81). List using (;) if more than 1. If not reported, fill this with "NR")

Model 5

Is this the recommended model?	◯ Yes<br>◯ No<br>(If there is only one model, please state 'Yes')
Development<br>A discrimination measure is presented	◯ Yes<br>◯ No<br>(E.g. C-index / area under the ROC curve)
External validation<br>A discrimination measure is presented	◯ Yes<br>◯ No<br>(E.g. C-index / area under the ROC curve)
Development<br>Which performance measures are described?	☐ AUC/AUROC/Area under the curve<br>☐ C-statistic<br>☐ Harrell's c-index<br>☐ D-statistic<br>(Multiples answers are possible)
External validation<br>Which performance measures are described?	☐ AUC/AUROC/Area under the curve<br>☐ C-statistic<br>☐ Harrell's c-index<br>☐ D-statistic<br>(Multiples answers are possible)

Confidential

The confidence interval (or standard error) of the AUC/AUROC/Area under the curve is presented	◯ Yes<br>◯ No
The confidence interval (or standard error) of the AUC/AUROC/Area under the curve is presented	◯ Yes<br>◯ No
What was the AUC/AUROC/Area under the curve apparent discrimination estimate (+95%CI, if given)?	__________________________________<br>(Please use the following format - estimate (lower CI - upper CI). For example 0.79 (0.75 - 0.81). If not reported fill this with "NR")
What was the AUC/AUROC/Area under the curve apparent discrimination estimate (+95%CI, if given)?	__________________________________<br>(Please use the following format - estimate (lower CI - upper CI). For example 0.79 (0.75 - 0.81). If not reported fill this with "NR")
Was the AUC/AUROC/Area under the curve corrected for optimism?	◯ Yes<br>◯ No<br>◯ Unclear
What was the optimism corrected AUC/AUROC/Area under the curve (95% CI, if given)?	__________________________________<br>(Please use the following format - estimate (lower CI - upper CI). For example 0.79 (0.75 - 0.81). If not reported fill this with "NR")
The confidence interval (or standard error) of the C-statistic is presented	◯ Yes<br>◯ No
The confidence interval (or standard error) of the C-statistic is presented	◯ Yes<br>◯ No
What was the C-statistic apparent discrimination estimate (+95%CI, if given)?	__________________________________<br>(Please use the following format - estimate (lower CI - upper CI). For example 0.79 (0.75 - 0.81). If not reported fill this with "NR")
What was the C-statistic apparent discrimination estimate (+95%CI, if given)?	__________________________________<br>(Please use the following format - estimate (lower CI - upper CI). For example 0.79 (0.75 - 0.81). If not reported fill this with "NR")
Was the C-statistic corrected for optimism?	◯ Yes<br>◯ No<br>◯ Unclear
What was the optimism corrected C-statistic (95% CI, if given)?	__________________________________<br>(Please use the following format - estimate (lower CI - upper CI). For example 0.79 (0.75 - 0.81). If not reported fill this with "NR")
The confidence interval (or standard error) of the Harrel's c-index is presented	◯ Yes<br>◯ No

The confidence interval (or standard error) of the Harrel's c-index is presented	◯ Yes<br>◯ No
What was the Harrel's c-index apparent discrimination estimate (+95%CI, if given)?	__________________________________<br>(Please use the following format - estimate (lower CI - upper CI). For example 0.79 (0.75 - 0.81). If not reported fill this with "NR")
What was the Harrel's c-index apparent discrimination estimate (+95%CI, if given)?	__________________________________<br>(Please use the following format - estimate (lower CI - upper CI). For example 0.79 (0.75 - 0.81). If not reported fill this with "NR")
Was the Harrel's c-index corrected for optimism?	◯ Yes<br>◯ No<br>◯ Unclear
What was the optimism corrected Harrel's c-index (95% CI, if given)?	__________________________________<br>(Please use the following format - estimate (lower CI - upper CI). For example 0.79 (0.75 - 0.81). If not reported fill this with "NR")
The confidence interval (or standard error) of the D-statistic is presented	◯ Yes<br>◯ No
The confidence interval (or standard error) of the D-statistic is presented	◯ Yes<br>◯ No
What was the D-statistic apparent discrimination estimate (+95%CI, if given)?	__________________________________<br>(Please use the following format - estimate (lower CI - upper CI). For example 0.79 (0.75 - 0.81). If not reported fill this with "NR")
What was the D-statistic apparent discrimination estimate (+95%CI, if given)?	__________________________________<br>(Please use the following format - estimate (lower CI - upper CI). For example 0.79 (0.75 - 0.81). If not reported fill this with "NR")
Was the D-statistic corrected for optimism?	◯ Yes<br>◯ No<br>◯ Unclear
What was the optimism corrected D-statistic (95% CI, if given)?	__________________________________<br>(Please use the following format - estimate (lower CI - upper CI). For example 0.79 (0.75 - 0.81). If not reported fill this with "NR")
Development<br>Measures for model calibration are described	◯ Yes<br>◯ No<br>(E.g. calibration plot, calibration slope or intercept, calibration table, Hosmer Lemeshow test, O/E ratio.)

External validation<br>Measures for model calibration are described	◯ Yes<br>◯ No<br>(E.g. calibration plot, calibration slope or intercept, calibration table, Hosmer Lemeshow test, O/E ratio.)
Development<br>How was calibration assessed?	☐ Calibration plot<br>☐ Calibration slope<br>☐ Calibration intercept<br>☐ Calibration in the large<br>☐ Calibration table<br>☐ Kappa<br>☐ Observed/expected ratio<br>☐ H-L
External validation<br>How was calibration assessed?	☐ Calibration plot<br>☐ Calibration slope<br>☐ Calibration intercept<br>☐ Calibration in the large<br>☐ Calibration table<br>☐ Kappa<br>☐ Observed/expected ratio<br>☐ H-L
What was the apparent [gen_methods_143:checked] estimate (+95% CI, if given)?	________________________________<br>(Please use the following format - estimate (lower CI - upper CI). For example 0.79 (0.75 - 0.81). List using (;) if more than 1. If not reported, fill this with "NR")
What was the apparent [gen_methods_144:checked] estimate (+95% CI, if given)?	________________________________<br>(Please use the following format - estimate (lower CI - upper CI). For example 0.79 (0.75 - 0.81). List using (;) if more than 1. If not reported, fill this with "NR")
Was the [gen_methods_143:checked] measure corrected for optimism?	◯ Yes<br>◯ No<br>◯ Unclear
What was the optimism corrected [gen_methods_143:checked] estimate (+95% CI, if given)?	________________________________<br>(Please use the following format - estimate (lower CI - upper CI). For example 0.79 (0.75 - 0.81). List using (;) if more than 1. If not reported, fill this with "NR")
Development<br>Other model performance measures are presented	◯ Yes<br>◯ No<br>(E.g. R2, Brier score, predictive values, sensitivity, specificity, AUC difference, decision curve analysis, net reclassification improvement, integrated discrimination improvement, AIC.)

External validation<br>Other model performance measures are presented	◯ Yes<br>◯ No<br>(E.g. R2, Brier score, predictive values, sensitivity, specificity, AUC difference, decision curve analysis, net reclassification improvement, integrated discrimination improvement, AIC.)
Development<br>If yes, please specify	☐ R2<br>☐ Brier score<br>☐ predictive values<br>☐ sensitivity<br>☐ specificity<br>☐ AUC difference<br>☐ decision curve analysis<br>☐ net reclassification improvement<br>☐ integrated discrimination improvement<br>☐ AIC<br>☐ Accuracy<br>☐ Other<br>(Multiples answers are possible)
External validation<br>If yes, please specify	☐ R2<br>☐ Brier score<br>☐ predictive values<br>☐ sensitivity<br>☐ specificity<br>☐ AUC difference<br>☐ decision curve analysis<br>☐ net reclassification improvement<br>☐ integrated discrimination improvement<br>☐ AIC<br>☐ Accuracy<br>☐ Other<br>(Multiples answers are possible)
If other, please specify	__________________________<br>(list using (;) to separate if more than 1)
If other, please specify	__________________________<br>(list using (;) to separate if more than 1)
What was the apparent [gen_methods_127:checked] estimate (+95% CI, if given)?	________________________________<br>(Please use the following format - estimate (lower CI - upper CI). For example 0.79 (0.75 - 0.81). List using (;) if more than 1. If not reported, fill this with "NR")
What was the apparent [gen_methods_128:checked] estimate (+95% CI, if given)?	________________________________<br>(Please use the following format - estimate (lower CI - upper CI). For example 0.79 (0.75 - 0.81). List using (;) if more than 1. If not reported, fill this with "NR")

Was the [gen_methods_127:checked] measure corrected for optimism?	◯ Yes<br>◯ No<br>◯ Unclear
What was the optimism corrected [gen_methods_127:checked] estimate (+95% CI, if given)?	__________________________________<br>(Please use the following format - estimate (lower CI - upper CI). For example 0.79 (0.75 - 0.81). List using (;) if more than 1. If not reported, fill this with "NR")

Model 6

Is this the recommended model?	◯ Yes<br>◯ No<br>(If there is only one model, please state 'Yes')
Development<br>A discrimination measure is presented	◯ Yes<br>◯ No<br>(E.g. C-index / area under the ROC curve)
External validation<br>A discrimination measure is presented	◯ Yes<br>◯ No<br>(E.g. C-index / area under the ROC curve)
Development<br>Which performance measures are described?	☐ AUC/AUROC/Area under the curve<br>☐ C-statistic<br>☐ Harrell's c-index<br>☐ D-statistic<br>(Multiples answers are possible)
External validation<br>Which performance measures are described?	☐ AUC/AUROC/Area under the curve<br>☐ C-statistic<br>☐ Harrell's c-index<br>☐ D-statistic<br>(Multiples answers are possible)
The confidence interval (or standard error) of the AUC/AUROC/Area under the curve is presented	◯ Yes<br>◯ No
The confidence interval (or standard error) of the AUC/AUROC/Area under the curve is presented	◯ Yes<br>◯ No
What was the AUC/AUROC/Area under the curve apparent discrimination estimate (+95%CI, if given)?	______________________________<br>(Please use the following format - estimate (lower CI - upper CI). For example 0.79 (0.75 - 0.81). If not reported fill this with "NR")
What was the AUC/AUROC/Area under the curve apparent discrimination estimate (+95%CI, if given)?	______________________________<br>(Please use the following format - estimate (lower CI - upper CI). For example 0.79 (0.75 - 0.81). If not reported fill this with "NR")

Confidential

Was the AUC/AUROC/Area under the curve corrected for optimism?	◯ Yes<br>◯ No<br>◯ Unclear
What was the optimism corrected AUC/AUROC/Area under the curve (95% CI, if given)?	______________________<br>(Please use the following format - estimate (lower CI - upper CI). For example 0.79 (0.75 - 0.81). If not reported fill this with "NR")
The confidence interval (or standard error) of the C-statistic is presented	◯ Yes<br>◯ No
The confidence interval (or standard error) of the C-statistic is presented	◯ Yes<br>◯ No
What was the C-statistic apparent discrimination estimate (+95%CI, if given)?	______________________<br>(Please use the following format - estimate (lower CI - upper CI). For example 0.79 (0.75 - 0.81). If not reported fill this with "NR")
What was the C-statistic apparent discrimination estimate (+95%CI, if given)?	______________________<br>(Please use the following format - estimate (lower CI - upper CI). For example 0.79 (0.75 - 0.81). If not reported fill this with "NR")
Was the C-statistic corrected for optimism?	◯ Yes<br>◯ No<br>◯ Unclear
What was the optimism corrected C-statistic (95% CI, if given)?	______________________<br>(Please use the following format - estimate (lower CI - upper CI). For example 0.79 (0.75 - 0.81). If not reported fill this with "NR")
The confidence interval (or standard error) of the Harrel's c-index is presented	◯ Yes<br>◯ No
The confidence interval (or standard error) of the Harrel's c-index is presented	◯ Yes<br>◯ No
What was the Harrel's c-index apparent discrimination estimate (+95%CI, if given)?	______________________<br>(Please use the following format - estimate (lower CI - upper CI). For example 0.79 (0.75 - 0.81). If not reported fill this with "NR")
What was the Harrel's c-index apparent discrimination estimate (+95%CI, if given)?	______________________<br>(Please use the following format - estimate (lower CI - upper CI). For example 0.79 (0.75 - 0.81). If not reported fill this with "NR")

Was the Harrel's c-index corrected for optimism?	◯ Yes<br>◯ No<br>◯ Unclear
What was the optimism corrected Harrel's c-index (95% CI, if given)?	________________________<br>(Please use the following format - estimate (lower CI - upper CI). For example 0.79 (0.75 - 0.81). If not reported fill this with "NR")
The confidence interval (or standard error) of the D-statistic is presented	◯ Yes<br>◯ No
The confidence interval (or standard error) of the D-statistic is presented	◯ Yes<br>◯ No
What was the D-statistic apparent discrimination estimate (+95%CI, if given)?	________________________<br>(Please use the following format - estimate (lower CI - upper CI). For example 0.79 (0.75 - 0.81). If not reported fill this with "NR")
What was the D-statistic apparent discrimination estimate (+95%CI, if given)?	________________________<br>(Please use the following format - estimate (lower CI - upper CI). For example 0.79 (0.75 - 0.81). If not reported fill this with "NR")
Was the D-statistic corrected for optimism?	◯ Yes<br>◯ No<br>◯ Unclear
What was the optimism corrected D-statistic (95% CI, if given)?	________________________<br>(Please use the following format - estimate (lower CI - upper CI). For example 0.79 (0.75 - 0.81). If not reported fill this with "NR")
Development<br>Measures for model calibration are described	◯ Yes<br>◯ No<br>(E.g. calibration plot, calibration slope or intercept, calibration table, Hosmer Lemeshow test, O/E ratio.)
External validation<br>Measures for model calibration are described	◯ Yes<br>◯ No<br>(E.g. calibration plot, calibration slope or intercept, calibration table, Hosmer Lemeshow test, O/E ratio.)
Development<br>How was calibration assessed?	☐ Calibration plot<br>☐ Calibration slope<br>☐ Calibration intercept<br>☐ Calibration in the large<br>☐ Calibration table<br>☐ Kappa<br>☐ Observed/expected ratio<br>☐ H-L

External validation<br>How was calibration assessed?	☐ Calibration plot<br>☐ Calibration slope<br>☐ Calibration intercept<br>☐ Calibration in the large<br>☐ Calibration table<br>☐ Kappa<br>☐ Observed/expected ratio<br>☐ H-L
What was the apparent [gen_methods_145:checked] estimate (+95% CI, if given)?	__________________________________<br>(Please use the following format - estimate (lower CI - upper CI). For example 0.79 (0.75 - 0.81). List using (;) if more than 1. If not reported, fill this with "NR")
What was the apparent [gen_methods_146:checked] estimate (+95% CI, if given)?	__________________________________<br>(Please use the following format - estimate (lower CI - upper CI). For example 0.79 (0.75 - 0.81). List using (;) if more than 1. If not reported, fill this with "NR")
Was the [gen_methods_145:checked] measure corrected for optimism?	◯ Yes<br>◯ No<br>◯ Unclear
What was the optimism corrected [gen_methods_145:checked] estimate (+95% CI, if given)?	__________________________________<br>(Please use the following format - estimate (lower CI - upper CI). For example 0.79 (0.75 - 0.81). List using (;) if more than 1. If not reported, fill this with "NR")
Development<br>Other model performance measures are presented	◯ Yes<br>◯ No<br>(E.g. R2, Brier score, predictive values, sensitivity, specificity, AUC difference, decision curve analysis, net reclassification improvement, integrated discrimination improvement, AIC.)
External validation<br>Other model performance measures are presented	◯ Yes<br>◯ No<br>(E.g. R2, Brier score, predictive values, sensitivity, specificity, AUC difference, decision curve analysis, net reclassification improvement, integrated discrimination improvement, AIC.)

Development<br>If yes, please specify	☐ R2<br>☐ Brier score<br>☐ predictive values<br>☐ sensitivity<br>☐ specificity<br>☐ AUC difference<br>☐ decision curve analysis<br>☐ net reclassification improvement<br>☐ integrated discrimination improvement<br>☐ AIC<br>☐ Accuracy<br>☐ Other<br>(Multiples answers are possible)
External validation<br>If yes, please specify	☐ R2<br>☐ Brier score<br>☐ predictive values<br>☐ sensitivity<br>☐ specificity<br>☐ AUC difference<br>☐ decision curve analysis<br>☐ net reclassification improvement<br>☐ integrated discrimination improvement<br>☐ AIC<br>☐ Accuracy<br>☐ Other<br>(Multiples answers are possible)
If other, please specify	__________________________________<br>(list using (;) to separate if more than 1)
If other, please specify	__________________________________<br>(list using (;) to separate if more than 1)
What was the apparent [gen_methods_129:checked] estimate (+95% CI, if given)?	__________________________________<br>(Please use the following format - estimate (lower CI - upper CI). For example 0.79 (0.75 - 0.81). List using (;) if more than 1. If not reported, fill this with "NR")
What was the apparent [gen_methods_138:checked] estimate (+95% CI, if given)?	__________________________________<br>(Please use the following format - estimate (lower CI - upper CI). For example 0.79 (0.75 - 0.81). List using (;) if more than 1. If not reported, fill this with "NR")
Was the [gen_methods_129:checked] measure corrected for optimism?	◯ Yes<br>◯ No<br>◯ Unclear

What was the optimism corrected [gen_methods_129:checked] estimate (+95% CI, if given)?	__________________________________ (Please use the following format - estimate (lower CI - upper CI). For example 0.79 (0.75 - 0.81). List using (;) if more than 1. If not reported, fill this with "NR")

Model 7

Is this the recommended model?	◯ Yes<br>◯ No<br>(If there is only one model, please state 'Yes')
Development<br>A discrimination measure is presented	◯ Yes<br>◯ No<br>(E.g. C-index / area under the ROC curve)
External validation<br>A discrimination measure is presented	◯ Yes<br>◯ No<br>(E.g. C-index / area under the ROC curve)
Development<br>Which performance measures are described?	☐ AUC/AUROC/Area under the curve<br>☐ C-statistic<br>☐ Harrell's c-index<br>☐ D-statistic<br>(Multiples answers are possible)
External validation<br>Which performance measures are described?	☐ AUC/AUROC/Area under the curve<br>☐ C-statistic<br>☐ Harrell's c-index<br>☐ D-statistic<br>(Multiples answers are possible)
The confidence interval (or standard error) of the AUC/AUROC/Area under the curve is presented	◯ Yes<br>◯ No
The confidence interval (or standard error) of the AUC/AUROC/Area under the curve is presented	◯ Yes<br>◯ No
What was the AUC/AUROC/Area under the curve apparent discrimination estimate (+95%CI, if given)?	____________________________<br>(Please use the following format - estimate (lower CI - upper CI). For example 0.79 (0.75 - 0.81). If not reported fill this with "NR")
What was the AUC/AUROC/Area under the curve apparent discrimination estimate (+95%CI, if given)?	____________________________<br>(Please use the following format - estimate (lower CI - upper CI). For example 0.79 (0.75 - 0.81). If not reported fill this with "NR")
Was the AUC/AUROC/Area under the curve corrected for optimism?	◯ Yes<br>◯ No<br>◯ Unclear

What was the optimism corrected AUC/AUROC/Area under the curve (95% CI, if given)?	__________________________________ (Please use the following format - estimate (lower CI - upper CI). For example 0.79 (0.75 - 0.81). If not reported fill this with "NR")
The confidence interval (or standard error) of the C-statistic is presented	◯ Yes<br>◯ No
The confidence interval (or standard error) of the C-statistic is presented	◯ Yes<br>◯ No
What was the C-statistic apparent discrimination estimate (+95%CI, if given)?	__________________________________ (Please use the following format - estimate (lower CI - upper CI). For example 0.79 (0.75 - 0.81). If not reported fill this with "NR")
What was the C-statistic apparent discrimination estimate (+95%CI, if given)?	__________________________________ (Please use the following format - estimate (lower CI - upper CI). For example 0.79 (0.75 - 0.81). If not reported fill this with "NR")
Was the C-statistic corrected for optimism?	◯ Yes<br>◯ No<br>◯ Unclear
What was the optimism corrected C-statistic (95% CI, if given)?	__________________________________ (Please use the following format - estimate (lower CI - upper CI). For example 0.79 (0.75 - 0.81). If not reported fill this with "NR")
The confidence interval (or standard error) of the Harrel's c-index is presented	◯ Yes<br>◯ No
The confidence interval (or standard error) of the Harrel's c-index is presented	◯ Yes<br>◯ No
What was the Harrel's c-index apparent discrimination estimate (+95%CI, if given)?	__________________________________ (Please use the following format - estimate (lower CI - upper CI). For example 0.79 (0.75 - 0.81). If not reported fill this with "NR")
What was the Harrel's c-index apparent discrimination estimate (+95%CI, if given)?	__________________________________ (Please use the following format - estimate (lower CI - upper CI). For example 0.79 (0.75 - 0.81). If not reported fill this with "NR")
Was the Harrel's c-index corrected for optimism?	◯ Yes<br>◯ No<br>◯ Unclear

What was the optimism corrected Harrel's c-index (95% CI, if given)?	__________________________________ (Please use the following format - estimate (lower CI - upper CI). For example 0.79 (0.75 - 0.81). If not reported fill this with "NR")
The confidence interval (or standard error) of the D-statistic is presented	◯ Yes<br>◯ No
The confidence interval (or standard error) of the D-statistic is presented	◯ Yes<br>◯ No
What was the D-statistic apparent discrimination estimate (+95%CI, if given)?	__________________________________ (Please use the following format - estimate (lower CI - upper CI). For example 0.79 (0.75 - 0.81). If not reported fill this with "NR")
What was the D-statistic apparent discrimination estimate (+95%CI, if given)?	__________________________________ (Please use the following format - estimate (lower CI - upper CI). For example 0.79 (0.75 - 0.81). If not reported fill this with "NR")
Was the D-statistic corrected for optimism?	◯ Yes<br>◯ No<br>◯ Unclear
What was the optimism corrected D-statistic (95% CI, if given)?	__________________________________ (Please use the following format - estimate (lower CI - upper CI). For example 0.79 (0.75 - 0.81). If not reported fill this with "NR")
Development<br>Measures for model calibration are described	◯ Yes<br>◯ No<br>(E.g. calibration plot, calibration slope or intercept, calibration table, Hosmer Lemeshow test, O/E ratio.)
External validation<br>Measures for model calibration are described	◯ Yes<br>◯ No<br>(E.g. calibration plot, calibration slope or intercept, calibration table, Hosmer Lemeshow test, O/E ratio.)
Development<br>How was calibration assessed?	☐ Calibration plot<br>☐ Calibration slope<br>☐ Calibration intercept<br>☐ Calibration in the large<br>☐ Calibration table<br>☐ Kappa<br>☐ Observed/expected ratio<br>☐ H-L

Andaur Navarro CL, *et al. BMJ Open* 2020; 10:e038832. doi: 10.1136/bmjopen-2020-038832

Confidential

External validation<br>How was calibration assessed?	☐ Calibration plot<br>☐ Calibration slope<br>☐ Calibration intercept<br>☐ Calibration in the large<br>☐ Calibration table<br>☐ Kappa<br>☐ Observed/expected ratio<br>☐ H-L
What was the apparent [gen_methods_147:checked] estimate (+95% CI, if given)?	__________________________________<br>(Please use the following format - estimate (lower CI - upper CI). For example 0.79 (0.75 - 0.81). List using (;) if more than 1. If not reported, fill this with "NR")
What was the apparent [gen_methods_148:checked] estimate (+95% CI, if given)?	__________________________________<br>(Please use the following format - estimate (lower CI - upper CI). For example 0.79 (0.75 - 0.81). List using (;) if more than 1. If not reported, fill this with "NR")
Was the [gen_methods_147:checked] measure corrected for optimism?	◯ Yes<br>◯ No<br>◯ Unclear
What was the optimism corrected [gen_methods_147:checked] estimate (+95% CI, if given)?	__________________________________<br>(Please use the following format - estimate (lower CI - upper CI). For example 0.79 (0.75 - 0.81). List using (;) if more than 1. If not reported, fill this with "NR")
Development<br>Other model performance measures are presented	◯ Yes<br>◯ No<br>(E.g. R2, Brier score, predictive values, sensitivity, specificity, AUC difference, decision curve analysis, net reclassification improvement, integrated discrimination improvement, AIC.)
External validation<br>Other model performance measures are presented	◯ Yes<br>◯ No<br>(E.g. R2, Brier score, predictive values, sensitivity, specificity, AUC difference, decision curve analysis, net reclassification improvement, integrated discrimination improvement, AIC.)

Andaur Navarro CL, *et al. BMJ Open* 2020; 10:e038832. doi: 10.1136/bmjopen-2020-038832

Development<br>If yes, please specify	☐ R2<br>☐ Brier score<br>☐ predictive values<br>☐ sensitivity<br>☐ specificity<br>☐ AUC difference<br>☐ decision curve analysis<br>☐ net reclassification improvement<br>☐ integrated discrimination improvement<br>☐ AIC<br>☐ Accuracy<br>☐ Other<br>(Multiples answers are possible)
External validation<br>If yes, please specify	☐ R2<br>☐ Brier score<br>☐ predictive values<br>☐ sensitivity<br>☐ specificity<br>☐ AUC difference<br>☐ decision curve analysis<br>☐ net reclassification improvement<br>☐ integrated discrimination improvement<br>☐ AIC<br>☐ Accuracy<br>☐ Other<br>(Multiples answers are possible)
If other, please specify	______________________________<br>(list using (;) to separate if more than 1.)
If other, please specify	______________________________<br>(list using (;) to separate if more than 1.)
What was the apparent [gen_methods_130:checked] estimate (+95% CI, if given)?	______________________________<br>(Please use the following format - estimate (lower CI - upper CI). For example 0.79 (0.75 - 0.81). List using (;) if more than 1. If not reported, fill this with "NR")
What was the apparent [gen_methods_137:checked] estimate (+95% CI, if given)?	______________________________<br>(Please use the following format - estimate (lower CI - upper CI). For example 0.79 (0.75 - 0.81). List using (;) if more than 1. If not reported, fill this with "NR")
Was the [gen_methods_130:checked] measure corrected for optimism?	◯ Yes<br>◯ No<br>◯ Unclear

Andaur Navarro CL, *et al. BMJ Open* 2020; 10:e038832. doi: 10.1136/bmjopen-2020-038832

What was the optimism corrected [gen_methods_130:checked] estimate (+95% CI, if given)?	____________________ (Please use the following format - estimate (lower CI - upper CI). For example 0.79 (0.75 - 0.81). List using (;) if more than 1. If not reported, fill this with "NR")

Model 8

Is this the recommended model?	◯ Yes<br>◯ No<br>(If there is only one model, please state 'Yes')
Development<br>A discrimination measure is presented	◯ Yes<br>◯ No<br>(E.g. C-index / area under the ROC curve)
External validation<br>A discrimination measure is presented	◯ Yes<br>◯ No<br>(E.g. C-index / area under the ROC curve)
Development<br>Which performance measures are described?	☐ AUC/AUROC/Area under the curve<br>☐ C-statistic<br>☐ Harrell's c-index<br>☐ D-statistic<br>(Multiples answers are possible)
External validation<br>Which performance measures are described?	☐ AUC/AUROC/Area under the curve<br>☐ C-statistic<br>☐ Harrell's c-index<br>☐ D-statistic<br>(Multiples answers are possible)
The confidence interval (or standard error) of the AUC/AUROC/Area under the curve is presented	◯ Yes<br>◯ No
The confidence interval (or standard error) of the AUC/AUROC/Area under the curve is presented	◯ Yes<br>◯ No
What was the AUC/AUROC/Area under the curve apparent discrimination estimate (+95%CI, if given)?	____________________ (Please use the following format - estimate (lower CI - upper CI). For example 0.79 (0.75 - 0.81). If not reported fill this with "NR")
What was the AUC/AUROC/Area under the curve apparent discrimination estimate (+95%CI, if given)?	____________________ (Please use the following format - estimate (lower CI - upper CI). For example 0.79 (0.75 - 0.81). If not reported fill this with "NR")
Was the AUC/AUROC/Area under the curve corrected for optimism?	◯ Yes<br>◯ No<br>◯ Unclear

What was the optimism corrected AUC/AUROC/Area under the curve (95% CI, if given)?	__________________________________ (Please use the following format - estimate (lower CI - upper CI). For example 0.79 (0.75 - 0.81). If not reported fill this with "NR")
The confidence interval (or standard error) of the C-statistic is presented	◯ Yes<br>◯ No
The confidence interval (or standard error) of the C-statistic is presented	◯ Yes<br>◯ No
What was the C-statistic apparent discrimination estimate (+95%CI, if given)?	__________________________________ (Please use the following format - estimate (lower CI - upper CI). For example 0.79 (0.75 - 0.81). If not reported fill this with "NR")
What was the C-statistic apparent discrimination estimate (+95%CI, if given)?	__________________________________ (Please use the following format - estimate (lower CI - upper CI). For example 0.79 (0.75 - 0.81). If not reported fill this with "NR")
Was the C-statistic corrected for optimism?	◯ Yes<br>◯ No<br>◯ Unclear
What was the optimism corrected C-statistic (95% CI, if given)?	__________________________________ (Please use the following format - estimate (lower CI - upper CI). For example 0.79 (0.75 - 0.81). If not reported fill this with "NR")
The confidence interval (or standard error) of the Harrel's c-index is presented	◯ Yes<br>◯ No
The confidence interval (or standard error) of the Harrel's c-index is presented	◯ Yes<br>◯ No
What was the Harrel's c-index apparent discrimination estimate (+95%CI, if given)?	__________________________________ (Please use the following format - estimate (lower CI - upper CI). For example 0.79 (0.75 - 0.81). If not reported fill this with "NR")
What was the Harrel's c-index apparent discrimination estimate (+95%CI, if given)?	__________________________________ (Please use the following format - estimate (lower CI - upper CI). For example 0.79 (0.75 - 0.81). If not reported fill this with "NR")
Was the Harrel's c-index corrected for optimism?	◯ Yes<br>◯ No<br>◯ Unclear

Was the Harrel's c-index corrected for optimism?	◯ Yes<br>◯ No<br>◯ Unclear
What was the optimism corrected Harrel's c-index (95% CI, if given)?	______________________________<br>(Please use the following format - estimate (lower CI - upper CI). For example 0.79 (0.75 - 0.81). If not reported fill this with "NR")
What was the optimism corrected Harrel's c-index (95% CI, if given)?	______________________________<br>(Please use the following format - estimate (lower CI - upper CI). For example 0.79 (0.75 - 0.81). If not reported fill this with "NR")
The confidence interval (or standard error) of the D-statistic is presented	◯ Yes<br>◯ No
The confidence interval (or standard error) of the D-statistic is presented	◯ Yes<br>◯ No
What was the D-statistic apparent discrimination estimate (+95%CI, if given)?	______________________________<br>(Please use the following format - estimate (lower CI - upper CI). For example 0.79 (0.75 - 0.81). If not reported fill this with "NR")
What was the D-statistic apparent discrimination estimate (+95%CI, if given)?	______________________________<br>(Please use the following format - estimate (lower CI - upper CI). For example 0.79 (0.75 - 0.81). If not reported fill this with "NR")
Was the D-statistic corrected for optimism?	◯ Yes<br>◯ No<br>◯ Unclear
What was the optimism corrected D-statistic (95% CI, if given)?	______________________________<br>(Please use the following format - estimate (lower CI - upper CI). For example 0.79 (0.75 - 0.81). If not reported fill this with "NR")
Development<br>Measures for model calibration are described	◯ Yes<br>◯ No<br>(E.g. calibration plot, calibration slope or intercept, calibration table, Hosmer Lemeshow test, O/E ratio.)
External validation<br>Measures for model calibration are described	◯ Yes<br>◯ No<br>(E.g. calibration plot, calibration slope or intercept, calibration table, Hosmer Lemeshow test, O/E ratio.)

Development<br>How was calibration assessed?	☐ Calibration plot<br>☐ Calibration slope<br>☐ Calibration intercept<br>☐ Calibration in the large<br>☐ Calibration table<br>☐ Kappa<br>☐ Observed/expected ratio<br>☐ H-L
External validation<br>How was calibration assessed?	☐ Calibration plot<br>☐ Calibration slope<br>☐ Calibration intercept<br>☐ Calibration in the large<br>☐ Calibration table<br>☐ Kappa<br>☐ Observed/expected ratio<br>☐ H-L
What was the apparent [gen_methods_149:checked] estimate (+95% CI, if given)?	__________________________________<br>(Please use the following format - estimate (lower CI - upper CI). For example 0.79 (0.75 - 0.81). List using (;) if more than 1. If not reported, fill this with "NR")
What was the apparent [gen_methods_150:checked] estimate (+95% CI, if given)?	__________________________________<br>(Please use the following format - estimate (lower CI - upper CI). For example 0.79 (0.75 - 0.81). List using (;) if more than 1. If not reported, fill this with "NR")
Was the [gen_methods_149:checked] measure corrected for optimism?	◯ Yes<br>◯ No<br>◯ Unclear
What was the optimism corrected [gen_methods_149:checked] estimate (+95% CI, if given)?	__________________________________<br>(Please use the following format - estimate (lower CI - upper CI). For example 0.79 (0.75 - 0.81). List using (;) if more than 1. If not reported, fill this with "NR")
Development<br>Other model performance measures are presented	◯ Yes<br>◯ No<br>(E.g. R2, Brier score, predictive values, sensitivity, specificity, AUC difference, decision curve analysis, net reclassification improvement, integrated discrimination improvement, AIC.)
External validation<br>Other model performance measures are presented	◯ Yes<br>◯ No<br>(E.g. R2, Brier score, predictive values, sensitivity, specificity, AUC difference, decision curve analysis, net reclassification improvement, integrated discrimination improvement, AIC.)

Confidential

Development<br>If yes, please specify	☐ R2<br>☐ Brier score<br>☐ predictive values<br>☐ sensitivity<br>☐ specificity<br>☐ AUC difference<br>☐ decision curve analysis<br>☐ net reclassification improvement<br>☐ integrated discrimination improvement<br>☐ AIC<br>☐ Accuracy<br>☐ Other<br>(Multiples answers are possible)
External validation<br>If yes, please specify	☐ R2<br>☐ Brier score<br>☐ predictive values<br>☐ sensitivity<br>☐ specificity<br>☐ AUC difference<br>☐ decision curve analysis<br>☐ net reclassification improvement<br>☐ integrated discrimination improvement<br>☐ AIC<br>☐ Accuracy<br>☐ Other<br>(Multiples answers are possible)
If other, please specify	__________________________<br>(list using (;) to separate if more than 1.)
If other, please specify	__________________________<br>(list using (;) to separate if more than 1.)
What was the apparent [gen_methods_131:checked] estimate (+95% CI, if given)?	________________________________<br>(Please use the following format - estimate (lower CI - upper CI). For example 0.79 (0.75 - 0.81). List using (;) if more than 1. If not reported, fill this with "NR")
What was the apparent [gen_methods_136:checked] estimate (+95% CI, if given)?	________________________________<br>(Please use the following format - estimate (lower CI - upper CI). For example 0.79 (0.75 - 0.81). List using (;) if more than 1. If not reported, fill this with "NR")
Was the [gen_methods_131:checked] measure corrected for optimism?	◯ Yes<br>◯ No<br>◯ Unclear

What was the optimism corrected [gen_methods_131:checked] estimate (+95% CI, if given)?	______________________________ (Please use the following format - estimate (lower CI - upper CI). For example 0.79 (0.75 - 0.81). List using (;) if more than 1. If not reported, fill this with "NR")

Model 9

Is this the recommended model?	◯ Yes<br>◯ No<br>(If there is only one model, please state 'Yes')
Development<br>A discrimination measure is presented	◯ Yes<br>◯ No<br>(E.g. C-index / area under the ROC curve)
External validation<br>A discrimination measure is presented	◯ Yes<br>◯ No<br>(E.g. C-index / area under the ROC curve)
Development<br>Which performance measures are described?	☐ AUC/AUROC/Area under the curve<br>☐ C-statistic<br>☐ Harrell's c-index<br>☐ D-statistic<br>(Multiples answers are possible)
External validation<br>Which performance measures are described?	☐ AUC/AUROC/Area under the curve<br>☐ C-statistic<br>☐ Harrell's c-index<br>☐ D-statistic<br>(Multiples answers are possible)
The confidence interval (or standard error) of the AUC/AUROC/Area under the curve is presented	◯ Yes<br>◯ No
The confidence interval (or standard error) of the AUC/AUROC/Area under the curve is presented	◯ Yes<br>◯ No
What was the AUC/AUROC/Area under the curve apparent discrimination estimate (+95%CI, if given)?	______________________________ (Please use the following format - estimate (lower CI - upper CI). For example 0.79 (0.75 - 0.81). If not reported fill this with "NR")
What was the AUC/AUROC/Area under the curve apparent discrimination estimate (+95%CI, if given)?	______________________________ (Please use the following format - estimate (lower CI - upper CI). For example 0.79 (0.75 - 0.81). If not reported fill this with "NR")
Was the AUC/AUROC/Area under the curve corrected for optimism?	◯ Yes<br>◯ No<br>◯ Unclear

Confidential

What was the optimism corrected AUC/AUROC/Area under the curve (95% CI, if given)?	__________________ (Please use the following format - estimate (lower CI - upper CI). For example 0.79 (0.75 - 0.81). If not reported fill this with "NR")
The confidence interval (or standard error) of the C-statistic is presented	◯ Yes<br>◯ No
The confidence interval (or standard error) of the C-statistic is presented	◯ Yes<br>◯ No
What was the C-statistic apparent discrimination estimate (+95%CI, if given)?	__________________ (Please use the following format - estimate (lower CI - upper CI). For example 0.79 (0.75 - 0.81). If not reported fill this with "NR")
What was the C-statistic apparent discrimination estimate (+95%CI, if given)?	__________________ (Please use the following format - estimate (lower CI - upper CI). For example 0.79 (0.75 - 0.81). If not reported fill this with "NR")
Was the C-statistic corrected for optimism?	◯ Yes<br>◯ No<br>◯ Unclear
What was the optimism corrected C-statistic (95% CI, if given)?	__________________ (Please use the following format - estimate (lower CI - upper CI). For example 0.79 (0.75 - 0.81). If not reported fill this with "NR")
The confidence interval (or standard error) of the Harrel's c-index is presented	◯ Yes<br>◯ No
The confidence interval (or standard error) of the Harrel's c-index is presented	◯ Yes<br>◯ No
What was the Harrel's c-index apparent discrimination estimate (+95%CI, if given)?	__________________ (Please use the following format - estimate (lower CI - upper CI). For example 0.79 (0.75 - 0.81). If not reported fill this with "NR")
What was the Harrel's c-index apparent discrimination estimate (+95%CI, if given)?	__________________ (Please use the following format - estimate (lower CI - upper CI). For example 0.79 (0.75 - 0.81). If not reported fill this with "NR")
Was the Harrel's c-index corrected for optimism?	◯ Yes<br>◯ No<br>◯ Unclear

What was the optimism corrected Harrel's c-index (95% CI, if given)?	__________________ (Please use the following format - estimate (lower CI - upper CI). For example 0.79 (0.75 - 0.81). If not reported fill this with "NR")
The confidence interval (or standard error) of the D-statistic is presented	○ Yes<br>○ No
The confidence interval (or standard error) of the D-statistic is presented	○ Yes<br>○ No
What was the D-statistic apparent discrimination estimate (+95%CI, if given)?	__________________ (Please use the following format - estimate (lower CI - upper CI). For example 0.79 (0.75 - 0.81). If not reported fill this with "NR")
What was the D-statistic apparent discrimination estimate (+95%CI, if given)?	__________________ (Please use the following format - estimate (lower CI - upper CI). For example 0.79 (0.75 - 0.81). If not reported fill this with "NR")
Was the D-statistic corrected for optimism?	○ Yes<br>○ No<br>○ Unclear
What was the optimism corrected D-statistic (95% CI, if given)?	__________________ (Please use the following format - estimate (lower CI - upper CI). For example 0.79 (0.75 - 0.81). If not reported fill this with "NR")
Development<br>Measures for model calibration are described	○ Yes<br>○ No<br>(E.g. calibration plot, calibration slope or intercept, calibration table, Hosmer Lemeshow test, O/E ratio.)
External validation<br>Measures for model calibration are described	○ Yes<br>○ No<br>(E.g. calibration plot, calibration slope or intercept, calibration table, Hosmer Lemeshow test, O/E ratio.)
Development<br>How was calibration assessed?	☐ Calibration plot<br>☐ Calibration slope<br>☐ Calibration intercept<br>☐ Calibration in the large<br>☐ Calibration table<br>☐ Kappa<br>☐ Observed/expected ratio<br>☐ H-L

External validation<br>How was calibration assessed?	☐ Calibration plot<br>☐ Calibration slope<br>☐ Calibration intercept<br>☐ Calibration in the large<br>☐ Calibration table<br>☐ Kappa<br>☐ Observed/expected ratio<br>☐ H-L
What was the apparent [gen_methods_155:checked] estimate (+95% CI, if given)?	__________________<br>(Please use the following format - estimate (lower CI - upper CI). For example 0.79 (0.75 - 0.81). List using (;) if more than 1. If not reported, fill this with "NR")
What was the apparent [gen_methods_166:checked] estimate (+95% CI, if given)?	__________________<br>(Please use the following format - estimate (lower CI - upper CI). For example 0.79 (0.75 - 0.81). List using (;) if more than 1. If not reported, fill this with "NR")
Was the [gen_methods_155:checked] measure corrected for optimism?	◯ Yes<br>◯ No<br>◯ Unclear
What was the optimism corrected [gen_methods_155:checked] estimate (+95% CI, if given)?	__________________<br>(Please use the following format - estimate (lower CI - upper CI). For example 0.79 (0.75 - 0.81). List using (;) if more than 1. If not reported, fill this with "NR")
Development<br>Other model performance measures are presented	◯ Yes<br>◯ No<br>(E.g. R2, Brier score, predictive values, sensitivity, specificity, AUC difference, decision curve analysis, net reclassification improvement, integrated discrimination improvement, AIC.)
External validation<br>Other model performance measures are presented	◯ Yes<br>◯ No<br>(E.g. R2, Brier score, predictive values, sensitivity, specificity, AUC difference, decision curve analysis, net reclassification improvement, integrated discrimination improvement, AIC.)

Development<br>If yes, please specify	☐ R2<br>☐ Brier score<br>☐ predictive values<br>☐ sensitivity<br>☐ specificity<br>☐ AUC difference<br>☐ decision curve analysis<br>☐ net reclassification improvement<br>☐ integrated discrimination improvement<br>☐ AIC<br>☐ Accuracy<br>☐ Other<br>(Multiples answers are possible)
External validation<br>If yes, please specify	☐ R2<br>☐ Brier score<br>☐ predictive values<br>☐ sensitivity<br>☐ specificity<br>☐ AUC difference<br>☐ decision curve analysis<br>☐ net reclassification improvement<br>☐ integrated discrimination improvement<br>☐ AIC<br>☐ Accuracy<br>☐ Other<br>(Multiples answers are possible)
If other, please specify	______________________<br>(list using (;) to separate if more than 1.)
If other, please specify	______________________<br>(list using (;) to separate if more than 1.)
What was the apparent [gen_methods_132:checked] estimate (+95% CI, if given)?	______________________<br>(Please use the following format - estimate (lower CI - upper CI). For example 0.79 (0.75 - 0.81). List using (;) if more than 1. If not reported, fill this with "NR")
What was the apparent [gen_methods_135:checked] estimate (+95% CI, if given)?	______________________<br>(Please use the following format - estimate (lower CI - upper CI). For example 0.79 (0.75 - 0.81). List using (;) if more than 1. If not reported, fill this with "NR")
Was the [gen_methods_132:checked] measure corrected for optimism?	◯ Yes<br>◯ No<br>◯ Unclear

What was the optimism corrected [gen_methods_132:checked] estimate (+95% CI, if given)?	__________________________________ (Please use the following format - estimate (lower CI - upper CI). For example 0.79 (0.75 - 0.81). List using (;) if more than 1. If not reported, fill this with "NR")

Model 10

Is this the recommended model?	◯ Yes<br>◯ No<br>(If there is only one model, please state 'Yes')
Development<br>A discrimination measure is presented	◯ Yes<br>◯ No<br>(E.g. C-index / area under the ROC curve)
External validation<br>A discrimination measure is presented	◯ Yes<br>◯ No<br>(E.g. C-index / area under the ROC curve)
Development<br>Which performance measures are described?	☐ AUC/AUROC/Area under the curve<br>☐ C-statistic<br>☐ Harrell's c-index<br>☐ D-statistic<br>(Multiples answers are possible)
External validation<br>Which performance measures are described?	☐ AUC/AUROC/Area under the curve<br>☐ C-statistic<br>☐ Harrell's c-index<br>☐ D-statistic<br>(Multiples answers are possible)
The confidence interval (or standard error) of the AUC/AUROC/Area under the curve is presented	◯ Yes<br>◯ No
The confidence interval (or standard error) of the AUC/AUROC/Area under the curve is presented	◯ Yes<br>◯ No
What was the AUC/AUROC/Area under the curve apparent discrimination estimate (+95%CI, if given)?	__________________________ (Please use the following format - estimate (lower CI - upper CI). For example 0.79 (0.75 - 0.81). If not reported fill this with "NR")
What was the AUC/AUROC/Area under the curve apparent discrimination estimate (+95%CI, if given)?	__________________________ (Please use the following format - estimate (lower CI - upper CI). For example 0.79 (0.75 - 0.81). If not reported fill this with "NR")
Was the AUC/AUROC/Area under the curve corrected for optimism?	◯ Yes<br>◯ No<br>◯ Unclear

What was the optimism corrected AUC/AUROC/Area under the curve (95% CI, if given)?	__________________________________ (Please use the following format - estimate (lower CI - upper CI). For example 0.79 (0.75 - 0.81). If not reported fill this with "NR")
The confidence interval (or standard error) of the C-statistic is presented	◯ Yes<br>◯ No
The confidence interval (or standard error) of the C-statistic is presented	◯ Yes<br>◯ No
What was the C-statistic apparent discrimination estimate (+95%CI, if given)?	__________________________________ (Please use the following format - estimate (lower CI - upper CI). For example 0.79 (0.75 - 0.81). If not reported fill this with "NR")
What was the C-statistic apparent discrimination estimate (+95%CI, if given)?	__________________________________ (Please use the following format - estimate (lower CI - upper CI). For example 0.79 (0.75 - 0.81). If not reported fill this with "NR")
Was the C-statistic corrected for optimism?	◯ Yes<br>◯ No<br>◯ Unclear
What was the optimism corrected C-statistic (95% CI, if given)?	__________________________________ (Please use the following format - estimate (lower CI - upper CI). For example 0.79 (0.75 - 0.81). If not reported fill this with "NR")
The confidence interval (or standard error) of the Harrel's c-index is presented	◯ Yes<br>◯ No
The confidence interval (or standard error) of the Harrel's c-index is presented	◯ Yes<br>◯ No
What was the Harrel's c-index apparent discrimination estimate (+95%CI, if given)?	__________________________________ (Please use the following format - estimate (lower CI - upper CI). For example 0.79 (0.75 - 0.81). If not reported fill this with "NR")
What was the Harrel's c-index apparent discrimination estimate (+95%CI, if given)?	__________________________________ (Please use the following format - estimate (lower CI - upper CI). For example 0.79 (0.75 - 0.81). If not reported fill this with "NR")
Was the Harrel's c-index corrected for optimism?	◯ Yes<br>◯ No<br>◯ Unclear

What was the optimism corrected Harrel's c-index (95% CI, if given)?	______________________ (Please use the following format - estimate (lower CI - upper CI). For example 0.79 (0.75 - 0.81). If not reported fill this with "NR")
The confidence interval (or standard error) of the D-statistic is presented	◯ Yes<br>◯ No
The confidence interval (or standard error) of the D-statistic is presented	◯ Yes<br>◯ No
What was the D-statistic apparent discrimination estimate (+95%CI, if given)?	______________________ (Please use the following format - estimate (lower CI - upper CI). For example 0.79 (0.75 - 0.81). If not reported fill this with "NR")
What was the D-statistic apparent discrimination estimate (+95%CI, if given)?	______________________ (Please use the following format - estimate (lower CI - upper CI). For example 0.79 (0.75 - 0.81). If not reported fill this with "NR")
Was the D-statistic corrected for optimism?	◯ Yes<br>◯ No<br>◯ Unclear
What was the optimism corrected D-statistic (95% CI, if given)?	______________________ (Please use the following format - estimate (lower CI - upper CI). For example 0.79 (0.75 - 0.81). If not reported fill this with "NR")
Development<br>Measures for model calibration are described	◯ Yes<br>◯ No<br>(E.g. calibration plot, calibration slope or intercept, calibration table, Hosmer Lemeshow test, O/E ratio.)
External validation<br>Measures for model calibration are described	◯ Yes<br>◯ No<br>(E.g. calibration plot, calibration slope or intercept, calibration table, Hosmer Lemeshow test, O/E ratio.)
Development<br>How was calibration assessed?	☐ Calibration plot<br>☐ Calibration slope<br>☐ Calibration intercept<br>☐ Calibration in the large<br>☐ Calibration table<br>☐ Kappa<br>☐ Observed/expected ratio<br>☐ H-L

Andaur Navarro CL, *et al. BMJ Open* 2020; 10:e038832. doi: 10.1136/bmjopen-2020-038832

Confidential

External validation<br>How was calibration assessed?	☐ Calibration plot<br>☐ Calibration slope<br>☐ Calibration intercept<br>☐ Calibration in the large<br>☐ Calibration table<br>☐ Kappa<br>☐ Observed/expected ratio<br>☐ H-L
What was the apparent [gen_methods_167:checked] estimate (+95% CI, if given)?	__________________________________<br>(Please use the following format - estimate (lower CI - upper CI). For example 0.79 (0.75 - 0.81). List using (;) if more than 1. If not reported, fill this with "NR")
What was the apparent [gen_methods_168:checked] estimate (+95% CI, if given)?	__________________________________<br>(Please use the following format - estimate (lower CI - upper CI). For example 0.79 (0.75 - 0.81). List using (;) if more than 1. If not reported, fill this with "NR")
Was the [gen_methods_167:checked] measure corrected for optimism?	◯ Yes<br>◯ No<br>◯ Unclear
What was the optimism corrected [gen_methods_167:checked] estimate (+95% CI, if given)?	__________________________________<br>(Please use the following format - estimate (lower CI - upper CI). For example 0.79 (0.75 - 0.81). List using (;) if more than 1. If not reported, fill this with "NR")
Development<br>Other model performance measures are presented	◯ Yes<br>◯ No<br>(E.g. R2, Brier score, predictive values, sensitivity, specificity, AUC difference, decision curve analysis, net reclassification improvement, integrated discrimination improvement, AIC.)
External validation<br>Other model performance measures are presented	◯ Yes<br>◯ No<br>(E.g. R2, Brier score, predictive values, sensitivity, specificity, AUC difference, decision curve analysis, net reclassification improvement, integrated discrimination improvement, AIC.)

Andaur Navarro CL, *et al. BMJ Open* 2020; 10:e038832. doi: 10.1136/bmjopen-2020-038832

Development<br>If yes, please specify	☐ R2<br>☐ Brier score<br>☐ predictive values<br>☐ sensitivity<br>☐ specificity<br>☐ AUC difference<br>☐ decision curve analysis<br>☐ net reclassification improvement<br>☐ integrated discrimination improvement<br>☐ AIC<br>☐ Accuracy<br>☐ Other<br>(Multiples answers are possible)
External validation<br>If yes, please specify	☐ R2<br>☐ Brier score<br>☐ predictive values<br>☐ sensitivity<br>☐ specificity<br>☐ AUC difference<br>☐ decision curve analysis<br>☐ net reclassification improvement<br>☐ integrated discrimination improvement<br>☐ AIC<br>☐ Accuracy<br>☐ Other<br>(Multiples answers are possible)
If other, please specify	______________________________<br>(list using (;) to separate if more than 1.)
If other, please specify	______________________________<br>(list using (;) to separate if more than 1.)
What was the apparent [gen_methods_133:checked] estimate (+95% CI, if given)?	____________________________________<br>(Please use the following format - estimate (lower CI - upper CI). For example 0.79 (0.75 - 0.81). List using (;) if more than 1. If not reported, fill this with "NR")
What was the apparent [gen_methods_134:checked] estimate (+95% CI, if given)?	____________________________________<br>(Please use the following format - estimate (lower CI - upper CI). For example 0.79 (0.75 - 0.81). List using (;) if more than 1. If not reported, fill this with "NR")
Was the [gen_methods_133:checked] measure corrected for optimism?	◯ Yes<br>◯ No<br>◯ Unclear

Andaur Navarro CL, *et al. BMJ Open* 2020; 10:e038832. doi: 10.1136/bmjopen-2020-038832

What was the optimism corrected [gen_methods_133:checked] estimate (+95% CI, if given)?	__________________ (Please use the following format - estimate (lower CI - upper CI). For example 0.79 (0.75 - 0.81). List using (;) if more than 1. If not reported, fill this with "NR")
**SPIN**	
Is there use of leading words/strong statement in the results section to describe model and/or model performance/accuracy/effectiveness?	◯ Yes<br>◯ No<br>(The prediction estimate is described with a value judgement like "statistically significant", "significant")
If yes, please specify the leading word/strong statement	☐ Novel<br>☐ Excellent<br>☐ Accurate<br>☐ Optimal<br>☐ Perfect<br>☐ Significant<br>☐ Other
If other, please specify	__________________ (list using (;) to separate if more than 1)
If yes, please copy the statement below	__________________
Is at least ONE non-significant/non relevant model reported?	◯ Yes<br>◯ No<br>◯ NA<br>(Select NA when only 1 model was developed)
If yes, did the authors make use of leading word to reject those non predictive models reported?	◯ Yes<br>◯ No<br>(E.g. The effect is said to be significant, although the 95% confidence interval of the adjusted odds ratio crosses 1; OR Words like "trend" or "borderline, "significance", "statistically significant" are used)
If yes, please copy the statement below	__________________
Is the prediction model defined in multiples ways?	◯ Yes<br>◯ No<br>(E.g. Different thresholds of categorization AND continuous, or absolute value and relative value)

Confidential

Presence of spin in the presentation of tables and figures	◯ Yes<br>◯ No<br>(E.g. Non-significant p values adjusted for multiple comparisons are written below the table, whereas significant unadjusted p values are highly visible)

Model Updating

The updated regression coefficients for each predictor in the model are reported	◯ Yes<br>◯ No<br>◯ NA<br>(If model updating was described as 'not needed', score Yes)
The updated intercept or cumulative baseline hazard or baseline survival (for at least one time point) is reported	◯ Yes<br>◯ No<br>◯ NA<br>(If model updating was described as 'not needed', score Yes)
The discrimination of the updated model is reported	◯ Yes<br>◯ No
The confidence interval (or standard error) of the discrimination measure of the updated model is reported	◯ Yes<br>◯ No
The calibration of the updated model is reported	◯ Yes<br>◯ No
The confidence interval (or standard error) of the calibration measure of the updated model is reported	◯ Yes<br>◯ No

Comments

Any additional comment about the results section of this article?	________________________________<br>(If there is something in the "Results" section that does not fit into the questions of this form - please use this space to detail. Also use this space to detail anything you are unsure about.)

Confidential

Discussion / Conclusion

Article ID

____

Limitation

Limitations of the study are discussed

◯ Yes
◯ No
(Discuss any limitations of the study is sufficient (such as non-representative sample, few events per predictor, missing data).)

Interpretation

Comparison of results to reported performance in previous development studies and/or other validation studies is given

◯ Yes
◯ No

If yes, is the comparison in favor of similar prediction models?

◯ Yes
◯ No
◯ Some outcomes in favour and not in favour for others
◯ Unclear

Overall Interpretation

An overall interpretation of the results is given

◯ Yes
◯ No
(Considering objectives, limitations, results from similar studies, and other relevant evidence.)

Is there emphasis on model relevance while there is not enough information given to concluded results are predictive?

◯ Yes
◯ No

Is there use of leading words/strong statement in the discussion section to describe model and/or model performance/accuracy/effectiveness?

◯ Yes
◯ No

If yes, which leading word/strong statement?

☐ Novel
☐ Excellent
☐ Accurate
☐ Optimal
☐ Perfect
☐ Significant
☐ Other
(Multiples answers are possible)

If other, please specify

____
(list using (;) to separate if more than 1)

If yes, plese copy the statement below

____

Andaur Navarro CL, *et al. BMJ Open* 2020; 10:e038832. doi: 10.1136/bmjopen-2020-038832

Is at least (1) non-significant/non relevant model discussed?	◯ Yes<br>◯ No<br>◯ NA<br>(Select NA when only 1 model was developed)
**Implications**	
The potential clinical use is discussed	◯ Yes<br>◯ No<br>(An explicit description of the context in which the prediction model is to be used (e.g. to identify high risk groups to help direct treatment, or to triage patients for referral to subsequent care).)
Does the recommendation include using the model in a difference clinical setting/population?	◯ Yes<br>◯ No
If yes, please provide the statement below	__________________________________
Implications for future research are discussed	◯ Yes<br>◯ No<br>(E.g. a description of what the next stage of investigation of the prediction model should be, such as "We suggest further external validation".)
If yes, please provide the statement below	__________________________________
Were there any other perspectives addressed in the discussion section?	◯ Yes<br>◯ No<br>(E.g. unexpected finding from the analyses.)
If yes, please provide the statement below or a short description	__________________________________
Is uncertainty reported in the discussion?	◯ Yes<br>◯ No<br>(The use of any verbs as "may" or "could", or any words as "likely to" or "maybe")
Is there any other misleading strategy reported in the discussion/conclusion section?	◯ Yes<br>◯ No
If yes, please provide the statement below	__________________________________

Confidential

**Other Information**	
Information about supplementary resources is provided	◯ Yes<br>◯ No
If yes, what type of supplementary material?	☐ Web calculator<br>☐ repository for dataset<br>☐ repository for R code<br>☐ extra results<br>☐ main results<br>☐ missing data<br>☐ predictors<br>☐ Protocol<br>☐ Details on statistics<br>☐ Other<br>(Multiples answers are possible. Protocol includes registered/published protocol as well.)
If other, please specify	__________________________________<br>(list using (;) to separate if more than 1)

**Funding**	
The source of funding is reported or there is explicit mention that there was no external funding involved	◯ Yes<br>◯ No
If yes, which type of funding?	◯ Profit<br>◯ Non-Profit<br>◯ Both<br>◯ Unclear
The role of funders is reported	◯ Yes<br>◯ No<br>◯ NA<br>(If there is no external funding, please select "NA")
Is there a "Disclosure of authors' potential conflicts of interest (COI)" section in the journal?	◯ Yes<br>◯ No
If yes, are COI reported?	◯ Yes<br>◯ No
If yes, how many authors declared COI?	__________________________________<br>(Number only the ones who declare having conflicts)
Any additional comment about the discussion/conclusion section of this article?	____<br>(If there is something in the "Discussion/conclusion" section that does not fit into the questions of this form - please use this space to detail. Also use this space to detail anything you are unsure about.)

Confidential

Title

Article ID	______________________
The words developing/development, validation/validating, incremental/added value (or synonyms) are reported in the title	◯ Yes<br>◯ No
Is the title supportive of the clinical relevance of the model, despite the study reporting non-significant/relevant measures?	◯ Yes<br>◯ No<br>(Title is inconsistent with the study results)
Is there use of leading words/strong statement in the title to describe model and/or model performance?	◯ Yes<br>◯ No<br>(E.g. Novel, excellent, acurrate, significant, promising, breakthrough, etc.)
If yes, please select the leading word(s)/strong statement(s) used	☐ Novel<br>☐ Excellent<br>☐ Accurate<br>☐ Optimal<br>☐ Perfect<br>☐ Significant<br>☐ Other<br>(Multiples answers are possible)
If other, please specify	______________________<br>(list using (;) to separate if more than 1)
The words prediction, risk prediction, prediction model, risk models, prognostic models, prognostic indices, risk scores (or synonyms) are reported in the title	◯ Yes<br>◯ No
The target population is reported in the title	◯ Yes<br>◯ No
The outcome to be predicted is reported in the title	◯ Yes<br>◯ No
Any additional comments about the title of this article?	______________________<br>(If there is something in the "Title" section that does not fit into the questions of this form - please use this space to detail. Also use this space to detail anything you are unsure about.)

Andaur Navarro CL, *et al. BMJ Open* 2020; 10:e038832. doi: 10.1136/bmjopen-2020-038832

Confidential

Abstract

Article ID	____________________
The objectives are reported in the abstract	◯ Yes<br>◯ No
Sources of data are reported in the abstract	◯ Yes<br>◯ No<br>(E.g. Prospective cohort, registry data, RCT data.)
The setting is reported in the abstract	◯ Yes<br>◯ No<br>(E.g. Primary care, secondary care, general population, adult care, or paediatric care. The setting should be reported for both the development and validation datasets, if applicable.)
A general definition of the study participants is reported in the abstract	◯ Yes<br>◯ No<br>(E.g. patients with suspicion of certain disease, patients with a specific disease, or general eligibility criteria.)
The overall sample size is reported in the abstract	◯ Yes<br>◯ No
The number of events (or % outcome together with overall sample size) is reported in the abstract	◯ Yes<br>◯ No<br>◯ NA<br>(If a continuous outcome was studied, score Not applicable)
Predictors included in the final model are reported in the abstract. For validation studies of well-known models, at least the name/acronym of the validated model is reported	◯ Yes<br>◯ No<br>(Broad descriptions are sufficient, e.g. 'all information from patient history and physical examination'. Check in the main text whether all predictors of the final model are indeed reported in the abstract.)
The outcome is reported in the abstract	◯ Yes<br>◯ No
Statistical methods are described in the abstract	◯ Yes<br>◯ No<br>(For model development, at least the type of statistical model should be reported. For validation studies a quote like "model's discrimination and calibration was assessed" is considered adequate. If done, methods of updating should be reported.)
ML techniques that will be used are reported in the abstract	◯ Yes<br>◯ No

Confidential

Results for model discrimination are reported in the abstract	◯ Yes<br>◯ No<br>(This should be reported separately for development and validation if a study includes both development and validation.)
If yes, which measures are described in the abstract?	________________________________<br>(list the names using (;) to separate if more than 1)
If yes, are confidence interval reported?	◯ Yes<br>◯ No
Results for model calibration are reported in the abstract	◯ Yes<br>◯ No<br>(This should be reported separately for development and validation if a study includes both development and validation.)
If yes, which measures are described in the abstract?	________________________________<br>(list the names using (;) to separate if more than 1)
If yes, are precision estimates reported?	◯ Yes<br>◯ No
Is there any other type of measures reported?	◯ Yes<br>◯ No
If yes, please specify	________________________________<br>(list the names using (;) to separate if more than 1)
Conclusions are reported in the abstract	◯ Yes<br>◯ No<br>(In publications addressing both model development and validation, there is no need for separate conclusions for both; one conclusion is sufficient.)
If yes, are the conclusion consistent with the reported results in the abstract section?	◯ Yes<br>◯ No
Does the conclusion statement focus solely on significant/relevant results?	◯ Yes<br>◯ No<br>(absence of non signicant results reports)
Is there emphasis on model relevance in the conclusion section of the abstract while there is not enough information given to concluded results are predictive?	◯ Yes<br>◯ No

Confidential

Is there discrepancies between the full-text (discussion) and abstract (conclusion) explanations of the study findings?	◯ Yes<br>◯ No<br>(The discussion is consistent with the study findings, whereas the abstract conclusion is not [+/−]; OR The discussion is not consistent with the study findings, whereas the abstract conclusion is [−/+])
If yes, please copy the statement below	__________________________________
What is the recommended next step for the prediction model?	☐ To be used in clinical practice<br>☐ Validate the models in a different setting/population<br>☐ Other recommendations for further studies<br>☐ None reported<br>(Multiples answers are possible)
If other recommendations, please specify	____________________________<br>(list using (;) to separate if more than 1)
Is there any reference to previous prediction model in literature in the abstract section?	◯ Yes<br>◯ No
If yes, please copy the statement below	__________________________________
Is there use of leading words/strong statement in the abstract section to describe model and/or model performance/accuracy/effectiveness?	◯ Yes<br>◯ No<br>(E.g. Novel, excellent, acurrate, significant, promising, breakthrough, etc.)
If yes, which leading words/strong statement?	☐ Novel<br>☐ Excellent<br>☐ Accurate<br>☐ Optimal<br>☐ Perfect<br>☐ Significant<br>☐ Other<br>(Multiples answers are possible)
If other, please specify	____________________________<br>(list using (;) to separate if more than 1)
Please copy the statement below	__________________________________
Is uncertainty reported in the abstract?	◯ Yes<br>◯ No<br>(The use of any verbs as "may" or "could", nor any words as "likely to" or "maybe")
Limitations are reported in the abstract	◯ Yes<br>◯ No

Confidential

Please copy the statement below	__________________________________
Any additional comment about the "abstract" section of this article?	__________________________________<br>(If there is something in the "Abstract" section that does not fit into the questions of this form - please use this space to detail. Also use this space to detail anything you are unsure about.)

Andaur Navarro CL, *et al. BMJ Open* 2020; 10:e038832. doi: 10.1136/bmjopen-2020-038832

Confidential

Probast

Article ID ______________________________

Instructions When assessing ROB using PROBAST, please refer to the "best performance" model for the primary outcome suggested by the authors

What type of study is reported?	◯ Development (including internal validation)<br>◯ Development with external validation (same model)<br>◯ Development with external validation (different model)<br>◯ External validation only<br>(This questions is repeated due to branching logic. Please answer again.)

Domain 1 : Participants

Development<br>Were appropriate data sources used, e.g. cohort, RCT or nested case-control study data?	◯ Yes / Probably yes<br>◯ No / Probably no<br>◯ No information
External validation<br>Were appropriate data sources used, e.g. cohort, RCT or nested case-control study data?	◯ Yes / Probably yes<br>◯ No / Probably no<br>◯ No information
Development<br>Were all inclusions and exclusions of participants appropriate?	◯ Yes / Probably yes<br>◯ No / Probably no<br>◯ No information<br>(Yes/probably yes: If inclusion and exclusion of participants was appropriate, so participants correspond to unselected participants of interest. No/probably no: If participants are included who would already have been identified as having the outcome and so are no longer participants at suspicion of disease (diagnostic studies) or at risk of developing outcome (prognostic studies), or if specific subgroups are excluded that may have altered the performance of the prediction model for the intended target population. No information: When there is no information on whether inappropriate inclusions or exclusions took place.)

Andaur Navarro CL, *et al. BMJ Open* 2020; 10:e038832. doi: 10.1136/bmjopen-2020-038832

Confidential

External validation<br>Were all inclusions and exclusions of participants appropriate?	◯ Yes / Probably yes<br>◯ No / Probably no<br>◯ No information<br>(Yes/probably yes: If inclusion and exclusion of participants was appropriate, so participants correspond to unselected participants of interest. No/probably no: If participants are included who would already have been identified as having the outcome and so are no longer participants at suspicion of disease (diagnostic studies) or at risk of developing outcome (prognostic studies), or if specific subgroups are excluded that may have altered the performance of the prediction model for the intended target population. No information: When there is no information on whether inappropriate inclusions or exclusions took place.)
Development<br>Risk of bias introduced by Participants	◯ Low ROB<br>◯ High ROB<br>◯ Unclear ROB<br>(Low risk of bias: If the answer to all signaling questions is "Yes" or "Probably yes," then risk of bias can be considered low. If ≥1 of the answers is "No" or "Probably no," the judgment could still be "Low risk of bias" but specific reasons should be provided why the risk of bias can be considered low. High risk of bias: If the answer to any of the signaling questions is "No" or "Probably no," there is a potential for bias, except if defined at low risk of bias above. Unclear risk of bias: If relevant information is missing for some of the signaling questions and none of the signaling questions is judged to put this domain at high risk of bias.)
External validation<br>Risk of bias introduced by Participants	◯ Low ROB<br>◯ High ROB<br>◯ Unclear ROB<br>(Low risk of bias: If the answer to all signaling questions is "Yes" or "Probably yes," then risk of bias can be considered low. If ≥1 of the answers is "No" or "Probably no," the judgment could still be "Low risk of bias" but specific reasons should be provided why the risk of bias can be considered low. High risk of bias: If the answer to any of the signaling questions is "No" or "Probably no," there is a potential for bias, except if defined at low risk of bias above. Unclear risk of bias: If relevant information is missing for some of the signaling questions and none of the signaling questions is judged to put this domain at high risk of bias.)
Development<br>Support for Judgement	__________________________________
External validation<br>Support for Judgement	__________________________________

Andaur Navarro CL, *et al. BMJ Open* 2020; 10:e038832. doi: 10.1136/bmjopen-2020-038832

Domain 2 : Predictors

Development<br>Were predictors defined and assessed in a similar way for all participants?	◯ Yes / Probably yes<br>◯ No / Probably No<br>◯ No information<br>(Yes/probably yes: If definitions of predictors and their assessment were similar for all participants. No/probably no: If different definitions were used for the same predictor or if predictors requiring subjective interpretation were assessed by differently experienced assessors. No information: If there is no information on how predictors were defined or assessed.)
External validation<br>Were predictors defined and assessed in a similar way for all participants?	◯ Yes / Probably yes<br>◯ No / Probably No<br>◯ No information<br>(Yes/probably yes: If definitions of predictors and their assessment were similar for all participants. No/probably no: If different definitions were used for the same predictor or if predictors requiring subjective interpretation were assessed by differently experienced assessors. No information: If there is no information on how predictors were defined or assessed.)
Development<br>Were predictor assessments made without knowledge of outcome data?	◯ Yes / Probably yes<br>◯ No / Probably no<br>◯ No information
External validation<br>Were predictor assessments made without knowledge of outcome data?	◯ Yes / Probably yes<br>◯ No / Probably no<br>◯ No information
Development<br>Are all predictors available at the time the model is intended to be used?	◯ Yes / Probably Yes<br>◯ No / Probably No<br>◯ No information
External validation<br>Are all predictors available at the time the model is intended to be used?	◯ Yes / Probably Yes<br>◯ No / Probably No<br>◯ No information

Andaur Navarro CL, *et al. BMJ Open* 2020; 10:e038832. doi: 10.1136/bmjopen-2020-038832

Development<br>Risk of bias introduced by predictors	◯ Low ROB<br>◯ High ROB<br>◯ Unclear ROB<br>(Low risk of bias: If the answer to all signaling questions is "Yes" or "Probably Yes," then risk of bias can be considered low. If ≥1 of the answers is "No" or "Probably no," the judgment could still be "Low risk of bias" but specific reasons should be provided why the risk of bias can be considered low, e.g., use of objective predictors not requiring subjective interpretation. High risk of bias: If the answer to any of the signaling questions is "No" or "Probably no," there is a potential for bias. Unclear risk of bias: If relevant information is missing for some of the signaling questions and none of the signaling questions is judged to put the domain at high risk of bias.)
External validation<br>Risk of bias introduced by predictors	◯ Low ROB<br>◯ High ROB<br>◯ Unclear ROB<br>(Low risk of bias: If the answer to all signaling questions is "Yes" or "Probably Yes," then risk of bias can be considered low. If ≥1 of the answers is "No" or "Probably no," the judgment could still be "Low risk of bias" but specific reasons should be provided why the risk of bias can be considered low, e.g., use of objective predictors not requiring subjective interpretation. High risk of bias: If the answer to any of the signaling questions is "No" or "Probably no," there is a potential for bias. Unclear risk of bias: If relevant information is missing for some of the signaling questions and none of the signaling questions is judged to put the domain at high risk of bias.)
Development<br>Support for Judgement	______________________________
External validation<br>Support for Judgement	______________________________
**Domain 3 : Outcome**	
Development<br>Was the outcome determined appropriately?	◯ Yes / Probably yes<br>◯ No / Probably no<br>◯ No information
External validation<br>Was the outcome determined appropriately?	◯ Yes / Probably yes<br>◯ No / Probably no<br>◯ No information
Development<br>Was a pre-specified or standard outcome definition used?	◯ Yes / Probably yes<br>◯ No / Probably no<br>◯ No information

Confidential

External validation<br>Was a pre-specified or standard outcome definition used?	◯ Yes / Probably yes<br>◯ No / Probably no<br>◯ No information
Development<br>Were predictors excluded from the outcome definition?	◯ Yes / Probably yes<br>◯ No / Probably no<br>◯ No information
External validation<br>Were predictors excluded from the outcome definition?	◯ Yes / Probably yes<br>◯ No / Probably no<br>◯ No information
Development<br>Was the outcome defined and determined in a similar way for all participants?	◯ Yes / Probably yes<br>◯ No / Probably no<br>◯ No information
External validation<br>Was the outcome defined and determined in a similar way for all participants?	◯ Yes / Probably yes<br>◯ No / Probably no<br>◯ No information
Development<br>Was the outcome determined without knowledge of predictor information?	◯ Yes / Probably yes<br>◯ No / Probably no<br>◯ No information
External validation<br>Was the outcome determined without knowledge of predictor information?	◯ Yes / Probably yes<br>◯ No / Probably no<br>◯ No information
Development<br>Was the time interval between predictor assessment and outcome determination appropriate?	◯ Yes / Probably yes<br>◯ No / Probably no<br>◯ No information
External validation<br>Was the time interval between predictor assessment and outcome determination appropriate?	◯ Yes / Probably yes<br>◯ No / Probably no<br>◯ No information
Development<br>Risk of bias introduced by the outcome	◯ Low ROB<br>◯ High ROB<br>◯ Unclear ROB<br>(Low risk of bias: If the answer to all signaling questions is "Yes" or "Probably yes," then risk of bias can be considered low. If ≥1 of the answers is "No" or "Probably no," the judgment could still be low risk of bias, but specific reasons should be provided why the risk of bias can be considered low, e.g., when the outcome was determined with knowledge of predictor information but the outcome assessment did not require much interpretation by the assessor (e.g., death regardless of cause). High risk of bias: If the answer to any of the signaling questions is "No" or "Probably no," there is a potential for bias. Unclear risk of bias: If relevant information about the outcome is missing for some of the signaling questions and none of the signaling questions is judged to put this domain at high risk of bias.)

Confidential

External validation<br>Risk of bias introduced by the outcome	◯ Low ROB<br>◯ High ROB<br>◯ Unclear ROB<br>(Low risk of bias: If the answer to all signaling questions is "Yes" or "Probably yes," then risk of bias can be considered low. If ≥1 of the answers is "No" or "Probably no," the judgment could still be low risk of bias, but specific reasons should be provided why the risk of bias can be considered low, e.g., when the outcome was determined with knowledge of predictor information but the outcome assessment did not require much interpretation by the assessor (e.g., death regardless of cause). High risk of bias: If the answer to any of the signaling questions is "No" or "Probably no," there is a potential for bias. Unclear risk of bias: If relevant information about the outcome is missing for some of the signaling questions and none of the signaling questions is judged to put this domain at high risk of bias.)
Development<br>Support for Judgement	____
External validation<br>Support for Judgement	____

Domain 4 : Analysis

Development<br>Were there a reasonable number of participants with the outcome?	◯ Yes / Probably yes<br>◯ No / Probably no<br>◯ No information<br>(Yes/probably yes: For model development studies, if the number of participants with the outcome relative to the number of candidate predictor parameters is ≥20 (EPV ≥20).* For model validation studies, if the number of participants with the outcome is ≥100. No/probably no: For model development studies, if the number of participants with the outcome relative to the number of candidate predictor parameters is < 10 (EPV < 10).* For model validation studies, if the number of participants with the outcome is < 100. No information: For model development studies, no information on the number of candidate predictor parameters or number of participants with the outcome, such that the EPV cannot be calculated. For model validation studies, no information on the number of participants with the outcome.)

Andaur Navarro CL, *et al. BMJ Open* 2020; 10:e038832. doi: 10.1136/bmjopen-2020-038832

Confidential

External validation<br>Were there a reasonable number of participants with the outcome?	◯ Yes / Probably yes<br>◯ No / Probably no<br>◯ No information<br>(Yes/probably yes: For model development studies, if the number of participants with the outcome relative to the number of candidate predictor parameters is ≥20 (EPV ≥20).* For model validation studies, if the number of participants with the outcome is ≥100. No/probably no: For model development studies, if the number of participants with the outcome relative to the number of candidate predictor parameters is < 10 (EPV < 10).* For model validation studies, if the number of participants with the outcome is < 100. No information: For model development studies, no information on the number of candidate predictor parameters or number of participants with the outcome, such that the EPV cannot be calculated. For model validation studies, no information on the number of participants with the outcome.)
Development<br>Were continuous and categorical handled appropriately?	◯ Yes / Probably yes<br>◯ No / Probably no<br>◯ No information<br>(Yes/probably yes: If continuous predictors are not converted into ≥2 categories when included in the model (i.e., dichotomized or categorized), or if continuous predictors are examined for nonlinearity using, for example, fractional polynomials or restricted cubic splines, or if categorical predictor groups are defined using a prespecified method. For model validation studies, if continuous predictors are included using the same definitions or transformations, and categorical variables are categorized using the same cut points, as compared with the development study. No/probably no: If categorical predictor group definitions do not use a prespecified method. For model development studies, if continuous predictors are converted into ≥2 categories when included in the model. For model validation studies, if continuous predictors are included using different definitions or transformations, or categorical variables are categorized using different cut points, as compared with the development study. No information: No information on whether continuous predictors are examined for nonlinearity and no information on how categorical predictor groups are defined. For model validation studies, no information on whether the same definitions or transformations and the same cut points are used, as compared with the development study.)

Confidential

External validation<br>Were continuous and categorical handled appropriately?	◯ Yes / Probably yes<br>◯ No / Probably no<br>◯ No information<br>(Yes/probably yes: If continuous predictors are not converted into ≥2 categories when included in the model (i.e., dichotomized or categorized), or if continuous predictors are examined for nonlinearity using, for example, fractional polynomials or restricted cubic splines, or if categorical predictor groups are defined using a prespecified method. For model validation studies, if continuous predictors are included using the same definitions or transformations, and categorical variables are categorized using the same cut points, as compared with the development study. No/probably no: If categorical predictor group definitions do not use a prespecified method. For model development studies, if continuous predictors are converted into ≥2 categories when included in the model. For model validation studies, if continuous predictors are included using different definitions or transformations, or categorical variables are categorized using different cut points, as compared with the development study. No information: No information on whether continuous predictors are examined for nonlinearity and no information on how categorical predictor groups are defined. For model validation studies, no information on whether the same definitions or transformations and the same cut points are used, as compared with the development study.)
Development<br>Were enrolled participants included in the analysis?	◯ Yes / Probably yes<br>◯ No / Probably no<br>◯ No information
External validation<br>Were enrolled participants included in the analysis?	◯ Yes / Probably yes<br>◯ No / Probably no<br>◯ No information
Development<br>Were participants with missing data handled appropriately?	◯ Yes / Probably yes<br>◯ No / Probably no<br>◯ No information
External validation<br>Were participants with missing data handled appropriately?	◯ Yes / Probably yes<br>◯ No / Probably no<br>◯ No information
Development<br>Was selection of predictors based on univariable analysis avoided?	◯ Yes / Probably yes<br>◯ No / Probably no<br>◯ No information
Development<br>Were complexities in the data (e.g., censoring, competing risks, sampling of control participants) accounted appropriately?	◯ Yes / Probably yes<br>◯ No / Probably no<br>◯ No information

Andaur Navarro CL, *et al. BMJ Open* 2020; 10:e038832. doi: 10.1136/bmjopen-2020-038832

External validation<br>Were complexities in the data (e.g., censoring, competing risks, sampling of control participants) accounted appropriately?	◯ Yes / Probably yes<br>◯ No / Probably no<br>◯ No information
Development<br>Were relevant model performance measures evaluated appropriately?	◯ Yes / Probably yes<br>◯ No / Probably no<br>◯ No information<br>(Yes/probably yes: If both calibration and discrimination are evaluated appropriately (including relevant measures tailored for models predicting survival outcomes). No/probably no: If both calibration and discrimination are not evaluated, or if only goodness-of-fit tests, such as the Hosmer-Lemeshow test, are used to evaluate calibration, or if for models predicting survival outcomes performance measures accounting for censoring are not used, or if classification measures (like sensitivity, specificity, or predictive values) were presented using predicted probability thresholds derived from the data set at hand. No information: Either calibration or discrimination are not reported, or no information is provided as to whether appropriate performance measures for survival outcomes are used (e.g., references to relevant literature or specific mention of methods, such as using Kaplan-Meier estimates), or no information on thresholds for estimating classification measures is given.)
External validation<br>Were relevant model performance measures evaluated appropriately?	◯ Yes / Probably yes<br>◯ No / Probably no<br>◯ No information<br>(Yes/probably yes: If both calibration and discrimination are evaluated appropriately (including relevant measures tailored for models predicting survival outcomes). No/probably no: If both calibration and discrimination are not evaluated, or if only goodness-of-fit tests, such as the Hosmer-Lemeshow test, are used to evaluate calibration, or if for models predicting survival outcomes performance measures accounting for censoring are not used, or if classification measures (like sensitivity, specificity, or predictive values) were presented using predicted probability thresholds derived from the data set at hand. No information: Either calibration or discrimination are not reported, or no information is provided as to whether appropriate performance measures for survival outcomes are used (e.g., references to relevant literature or specific mention of methods, such as using Kaplan-Meier estimates), or no information on thresholds for estimating classification measures is given.)
Development<br>Were model overfitting, under-fitting, and optimism in model performance accounted for?	◯ Yes / Probably yes<br>◯ No / Probably no<br>◯ No information

Confidential

External validation<br>Were model overfitting, under-fitting, and optimism in model performance accounted for?	◯ Yes / Probably yes<br>◯ No / Probably no<br>◯ No information
Development<br>Do predictors and their assigned weights in the final model correspond to the results from the reported multivariable analysis	◯ Yes / Probably yes<br>◯ No / Probably no<br>◯ No information
External validation<br>Do predictors and their assigned weights in the final model correspond to the results from the reported multivariable analysis	◯ Yes / Probably yes<br>◯ No / Probably no<br>◯ No information
Development<br>Risk of bias introduced by the analysis	◯ Low ROB<br>◯ High ROB<br>◯ Unclear ROB<br>(Low risk of bias: If the answer to all signaling questions is "Yes" or "Probably yes," then risk of bias can be considered low. If ≥1 of the answers is "No" or "Probably no," the judgment could still be low risk of bias, but specific reasons should be provided why the risk of bias can be considered low. High risk of bias: If the answer to any of the signaling questions is "No" or "Probably no," there is a potential for bias. Unclear risk of bias: If relevant information about the analysis is missing for some of the signaling questions but none of the signaling question answers is judged to put the analysis at high risk of bias)
External validation<br>Risk of bias introduced by the analysis	◯ Low ROB<br>◯ High ROB<br>◯ Unclear ROB<br>(Low risk of bias: If the answer to all signaling questions is "Yes" or "Probably yes," then risk of bias can be considered low. If ≥1 of the answers is "No" or "Probably no," the judgment could still be low risk of bias, but specific reasons should be provided why the risk of bias can be considered low. High risk of bias: If the answer to any of the signaling questions is "No" or "Probably no," there is a potential for bias. Unclear risk of bias: If relevant information about the analysis is missing for some of the signaling questions but none of the signaling question answers is judged to put the analysis at high risk of bias)
Development<br>Support for Judgement	__________________________________
External validation<br>Support for Judgement	__________________________________

Andaur Navarro CL, *et al. BMJ Open* 2020; 10:e038832. doi: 10.1136/bmjopen-2020-038832

Overall assessment of ROB

Development<br>Overall risk of bias	◯ Low risk of bias<br>◯ High risk of bias<br>◯ Unclear risk of bias<br>(Low ROB: If all domains were rated low risk of bias. If a prediction model was developed without any external validation, and it was rated as low risk of bias for all domains, consider downgrading to high risk of bias. Such a model evaluation can only be considered as low risk of bias, if the development was based on a very large data set and included some form of internal validation. High ROB: If ≥1 domain is judged to be at high risk of bias. Unclear ROB: If an unclear risk of bias was noted in ≥1 domain and it was low risk for all other domains.)
External validation<br>Overall risk of bias	◯ Low risk of bias<br>◯ High risk of bias<br>◯ Unclear risk of bias<br>(Low ROB: If all domains were rated low risk of bias. If a prediction model was developed without any external validation, and it was rated as low risk of bias for all domains, consider downgrading to high risk of bias. Such a model evaluation can only be considered as low risk of bias, if the development was based on a very large data set and included some form of internal validation. High ROB: If ≥1 domain is judged to be at high risk of bias. Unclear ROB: If an unclear risk of bias was noted in ≥1 domain and it was low risk for all other domains.)
Any additional comments about ROB on this article?	__________________________________<br>(If there is something in the "PROBAST" that does not fit into the questions of this form - please use this space to detail. Also use this space to detail anything you are unsure about.)
