# Supplementary material for: Protocol for a systematic review on the methodological and reporting quality of prediction model studies using machine learning techniques
Source: BMJ Open. 2020 Nov 11;10(11):e038832. doi: 10.1136/bmjopen-2020-038832 (PMC7661369; doi:10.1136/bmjopen-2020-038832)
Supplement: Author's manuscript [file bmjopen-2020-038832.draft_revisions.pdf]

BMJ Open

BMJ Open is committed to open peer review. As part of this commitment we make the peer review history of every article we publish publicly available.

When an article is published we post the peer reviewers' comments and the authors' responses online. We also post the versions of the paper that were used during peer review. These are the versions that the peer review comments apply to.

The versions of the paper that follow are the versions that were submitted during the peer review process. They are not the versions of record or the final published versions. They should not be cited or distributed as the published version of this manuscript.

BMJ Open is an open access journal and the full, final, typeset and author-corrected version of record of the manuscript is available on our site with no access controls, subscription charges or pay-per-view fees (http://bmjopen.bmj.com).

If you have any questions on BMJ Open's open peer review process please email info.bmjopen@bmj.com

BMJ Open

Protocol for a systematic review on the methodological and reporting quality of prediction model studies using machine learning techniques

Journal:	*BMJ Open*
Manuscript ID	bmjopen-2020-038832
Article Type:	Protocol
Date Submitted by the Author:	25-Mar-2020

Complete List of Authors:	Andaur Navarro, Constanza L.; Julius Center for Health Sciences and Primary Care, University Medical Center Utrecht, Utrecht University; Cochrane Netherlands, University Medical Center Utrecht, Utrecht University<br>Damen, Johanna; Julius Center for Health Scienes and Primary Care, University Medical Center Utrecht, Utrecht University; Cochrane Netherlands, University Medical Center Utrecht, Utrecht University<br>Takada, Toshihiko; Julius Center for Health Sciences and Primary Care, University Medical Center Utrecht, Utrecht University<br>Nijman, Steven W.J. ; Julius Center for Health Sciences and Primary Care, University Medical Center Utrecht, Utrecht University<br>Dhiman, Paula; Center for Statistics in Medicine, University of Oxford<br>Ma, Jie; Center for Statistics in Medicine, University of Oxford<br>Collins, Gary; Centre for Statistics in Medicine, University of Oxford<br>Bajpai, Ram; School of Primary, Community and Social Care, Keele Univeristy<br>Riley, Richard; School of Primary, Community and Social Care, Keele University<br>Moons, Karel; Julius Center for Health Sciences and Primary Care, University Medical Center Utrecht, Utrecht University; Cochrane Netherlands, University Medical Center Utrecht, Utrecht University<br>Hooft, Lotty; Julius Center for Health Sciences and Primary Care, University Medical Center Utrecht, Utrecht University; Cochrane Netherlands, University Medical Center Utrecht, Utrecht University

Keywords:	EPIDEMIOLOGY, STATISTICS & RESEARCH METHODS, PREVENTIVE MEDICINE

SCHOLARONE™ Manuscripts

I, the Submitting Author has the right to grant and does grant on behalf of all authors of the Work (as defined in the below author licence), an exclusive licence and/or a non-exclusive licence for contributions from authors who are: i) UK Crown employees; ii) where BMJ has agreed a CC-BY licence shall apply, and/or iii) in accordance with the terms applicable for US Federal Government officers or employees acting as part of their official duties; on a worldwide, perpetual, irrevocable, royalty-free basis to BMJ Publishing Group Ltd ("BMJ") its licensees and where the relevant Journal is co-owned by BMJ to the co-owners of the Journal, to publish the Work in this journal and any other BMJ products and to exploit all rights, as set out in our licence.

The Submitting Author accepts and understands that any supply made under these terms is made by BMJ to the Submitting Author unless you are acting as an employee on behalf of your employer or a postgraduate student of an affiliated institution which is paying any applicable article publishing charge ("APC") for Open Access articles. Where the Submitting Author wishes to make the Work available on an Open Access basis (and intends to pay the relevant APC), the terms of reuse of such Open Access shall be governed by a Creative Commons licence – details of these licences and which Creative Commons licence will apply to this Work are set out in our licence referred to above.

Other than as permitted in any relevant BMJ Author's Self Archiving Policies, I confirm this Work has not been accepted for publication elsewhere, is not being considered for publication elsewhere and does not duplicate material already published. I confirm all authors consent to publication of this Work and authorise the granting of this licence.

For peer review only

Original Article

Protocol for a systematic review on the methodological and reporting quality of prediction model studies using machine learning techniques

Constanza L Andaur Navarro[1,2] (c.l.andaurnavarro@umcutrecht.nl), Johanna A A Damen[1,2] (j.a.a.damen@umcutrecht.nl), Toshihiko Takada[1] (t.takada-3@umcutrecht.nl), Steven W J Nijman[1] (S.W.J.Nijman@umcutrecht.nl), Paula Dhiman[3] (paula.dihman@ndorms.ox.ac.uk), Jie Ma[3] (jie.ma@csm.ox.ac.uk), Gary S Collins[3] (gary.collins@csm.ox.ac.uk), Ram Bajpai[4] (r.bajpai@keele.ac.uk), Richard D Riley[4] (r.riley@keele.ac.uk), Karel GM Moons[1,2] (k.g.m.moons@umcutrecht.nl), Lotty Hooft[1,2] (l.hooft@umcutrecht.nl)

To be submitted to BMJ Open

Word Count: 2847/4000

Keywords: protocol, systematic review, machine learning, prediction, diagnosis, prognosis, TRIPOD, PROBAST, risk of bias, applicability.

Correspondence to: Constanza L Andaur Navarro, Julius Center for Health Sciences and Primary Care, Universiteitsweg 100, P.O. Box 85500, 3508 GA, Utrecht, The Netherland, +31 8875 68640. c.l.andaurnavarro@umcutrecht.nl

[1]Julius Center for Health Sciences and Primary Care, University Medical Center Utrecht, Utrecht University, Utrecht, The Netherlands.

[2]Cochrane Netherlands, University Medical Center Utrecht, Utrecht University, Utrecht, The Netherlands.

[3]Center for Statistics in Medicine, University of Oxford, Oxford, United Kingdom.

[4]School of Primary, Community and Social Care, Keele University, Keele, United Kingdom.

For peer review only - http://bmjopen.bmj.com/site/about/guidelines.xhtml

ABSTRACT

Introduction. Studies addressing the development and/or validation of diagnostic and prognostic prediction models are abundant in most clinical domains. Systematic reviews have shown that the methodological and reporting quality of prediction model studies is suboptimal. Due to the increasing availability of larger, routinely collected and complex medical data, and the rising application of Artificial Intelligence (AI) or Machine Learning (ML) techniques, the number of prediction model studies is expected to increase even further. Prediction models developed using AI or ML techniques are often labeled as a "black box" and little is known about their methodological and reporting quality. Therefore, this comprehensive systematic review aims to evaluate the reporting quality, the methodological conduct, and the risk of bias of prediction model studies that applied ML techniques for model development and/or validation.

Methods and Analysis. A search will be performed in PubMed to identify studies developing and/or validating prediction models using any ML methodology and across all medical fields. Studies will be included if they were published between 2018 and 2019, predict patient-related outcomes, use any study design or data source, and available in English. Screening of search results and data extraction from included articles will be performed by two independent reviewers. The primary outcomes of this systematic review are: (1) the adherence of ML based prediction model studies to the Transparent Reporting of a multivariable prediction model for individual prognosis or diagnosis (TRIPOD), and (2) the risk of bias in such studies as assessed using the Prediction model Risk Of Bias ASsessment Tool (PROBAST). A narrative synthesis will be conducted for all included studies. Findings will be stratified by study type, medical field, and prevalent machine learning methods, and will inform necessary extensions or updates of TRIPOD and PROBAST to better address prediction model studies that used AI or ML techniques.

Ethics and dissemination. Ethical approval is not required for this study because only available published data will be analyzed. Findings will be disseminated through peer-reviewed publications and scientific conferences.

Systematic review registration: PROSPERO, CRD42019161764.

For peer review only

For peer review only - http://bmjopen.bmj.com/site/about/guidelines.xhtml

ARTICLE SUMMARY

Strengths and Limitation of this study

- This protocol increases transparency to the methods that will be applied to assess prediction model studies using Artificial Intelligence or Machine Learning, reducing possible biases and allowing peer review.
- The systematic review will provide an overview and critical appraisal of the methodological and reporting quality, and risk of bias of prediction model studies using Machine Learning.
- The findings of the review will provide the needed evidence for the development of tailored methodological and reporting guidelines for prediction model studies based on Machine Learning techniques for individualized prediction.
- Machine Learning is a newly developed concept without a clear known definition yet. The terminology used to describe such techniques is often inconsistent with the one used in conventional prediction studies. Therefore, a sensitive search strategy is hard to build, which may result in a large number of abstracts to screen at initial stages.
- Language restriction to English might exclude additional studies published in other languages.

For peer review only

For peer review only - http://bmjopen.bmj.com/site/about/guidelines.xhtml

INTRODUCTION

Clinical prediction models aim to estimate the individualized probability that a particular outcome, e.g. condition or disease, is present (diagnostic models) or whether a specific outcome will occur in the future (prognostic models).[1-4] Studies addressing the development, validation, and updating of prediction models are abundant in most clinical domains. For example, in cardiovascular disease, more than 350 prediction models have been developed and only a few have been validated.[5] Moreover, systematic reviews have shown that, within different medical domains, the methodological and reporting quality of prediction model studies is suboptimal.[6-10] Due to the increasing availability of larger, routinely collected and complex medical data, and the rising application of Artificial Intelligence (AI) or Machine Learning (ML) techniques for clinical prediction, the number of prediction model studies is expected to increase even further.

Machine Learning can be described as techniques that directly and automatically learn from data without being explicitly programmed for that task, and often without any prior assumption.[11,12,13] Thus ML relies on patterns and inferences from the data itself. A perceived advantage of ML over conventional statistical techniques is its ability to analyze "big", non-linear and high dimensional data, and thus its ability to model complex associations and scenarios. Due to the novelty, diversity, flexibility, and complexity of ML techniques, ML based prediction model studies are often considered as a "black box" for many users. Inadequate reporting of, e.g. data sources, study design, modeling processes, number of predictors, and other data assumptions, makes prediction models developed with ML techniques published in medical journals difficult to interpret and to be validated by other researchers, creating barriers to their use in daily clinical practice.

Complete reporting is essential to judge the validity of any prediction model as it facilitates: study replication, independent validation of the prediction model, risk of bias assessments, interpretation of the results, meta-analysis of prediction models, and the judgment of the value and applicability of such model in real clinical settings for individualized predictions.[14] While complete reporting reveals the strengths and limitations of a prediction model, it also enhances the use and implementation of prediction model in clinical practice. The "Transparent Reporting

For peer review only

For peer review only - http://bmjopen.bmj.com/site/about/guidelines.xhtml

of a multivariable prediction model for Individual Prognosis Or Diagnosis (TRIPOD)" statement has been available since 2015, providing a checklist of 22 items considered essential for informative reporting of diagnostic or prognostic prediction model studies.[15,16] Similarly, the Prediction model Risk Of Bias ASsessment Tool (PROBAST) was published in 2019 to guide the critical appraisal of prediction model studies.[17,18] PROBAST provides signaling questions to facilitate both the applicability and risk of bias assessment of prediction model studies across four domains: participants, predictors, outcome, and analysis. This assessment can only be correctly implemented if prediction model studies are properly reported. Although TRIPOD and PROBAST both covered all types of prediction modelling studies, including those using ML techniques, their focus was on regression-based modeling. Thus, the challenges and necessity for reporting and quality assessment guidelines in the AI/ML field has been addressed by several authors and has led to initiatives such as, CONSORT-AI, SPIRIT-AI, TRIPOD-ML and PROBAST-ML.[19-21]

To improve the quality, transparency, and usability of ML based prediction models in medicine, it is important to explore the current use and reporting of ML techniques in prediction model studies, to evaluate the methodological conduct and risk of bias using PROBAST, and assess the adherence to TRIPOD by performing a comprehensive systematic review.[3,15-18, 22]

Study aim

The primary aim of this systematic review is to evaluate the reporting and the methodological conduct of studies reporting on prediction models developed with ML techniques, across all medical fields. Specific objectives are to:

1. Evaluate the reporting quality of prediction models developed using ML techniques based on TRIPOD.
2. Assess the methodological quality and the risks of bias in prediction model development or validation studies using ML techniques based on PROBAST.
3. Identify key and emerging concepts for the development of tailored adaptations or extensions of both TRIPOD and PROBAST.

For peer review only - http://bmjopen.bmj.com/site/about/guidelines.xhtml

134 ## METHODS AND ANALYSIS

135 Our systematic review protocol was registered with the International Prospective Register of Systematic Reviews (PROSPERO) on 19 December 2019 (CRD42019161764). This protocol was prepared using the Preferred Reporting Items for Systematic Reviews and Meta-Analysis Protocols (PRISMA-P) 2015 statement.[23]

139 ### Eligibility criteria

140 Articles will be eligible for this review when describing primary studies on the development and/or validation of a multivariable prediction model with at least 2 predictors, using any ML methodology within all medical fields, and published in 2018-2019. We will include studies with any study design and data source, all patient-related health outcomes, diagnostic and prognostic outcomes, all outcome formats, and restricted to humans only. Further details about inclusion criteria are given in Table 1.

Table 1 Definition of inclusion criteria

Inclusion criteria	Definition
Any study design	Articles that report the development and/or validation of a prediction model based on experimental studies or observational studies. This includes randomized controlled trials, prospective and, retrospective cohort, case-control studies, and case-cohort studies.
Using at least 2 predictors for risk prediction	Articles that report the development and/or validation of a prediction model using at least 2 predictors. Articles that use imaging or speech parameters as structured data plus other predictors such as e.g. clinical, demographics, histological and genetic risk scores features will be included.
Any data sources	Articles that report the development and/or validation of a prediction model using any structured data source, e.g. electronic medical records, claims data, and individual patient data meta-analysis data.
Any ML technique	Articles that report the use of any ML technique for development and/or validation of a prediction model. We will consider as a ML technique, a statistical technique based on advance computational capacity and lower human intervention.
Patient health-related outcomes	Articles that report the development and/or validation of a prediction model whose main outcome is on an individual. We will also include studies assessing health services performance such as length of stay or triage assessment.

For peer review only - http://bmjopen.bmj.com/site/about/guidelines.xhtml

All outcome measures format	Articles that report the development and/or validation of a prediction model whose main outcome has the following measure formats: continuous, binary, ordinal, multinomial, and time-to-event.

Articles will be excluded from this review when reporting models that make predictions for enhancing the reading of images or signals (rather than for prediction of health outcomes in individuals) or, use only genetic or molecular markers as candidate predictors. Furthermore, prognostic factor studies, secondary research, conference abstracts, and studies for which no full-text is available will also be excluded. The search will be restricted to articles available in English only. Further details about exclusion criteria are given in Table 2.

Table 2 Definition of exclusion criteria

Exclusion criteria	Definition
Images or signal studies	Articles that report the development and/or validation of a prediction model for enhancing the reading of images, pathological samples or signals.
Only genetic and/or molecular predictors	Articles that report the development and/or validation of a prediction model using only genetic and/or molecular candidate predictors.
Prognostic factors studies	Articles that report the identification of factors associated with a clinical outcome in an individual.
Secondary research	Articles that report narrative reviews, systematic reviews about prediction model studies in a specific medical field. Guidelines, expert's opinions and letters to the editor will also be excluded.
Conference abstract	Articles that report the development and/or validation of a prediction model presented in a conference. Such articles, by definition, do not report all the information required for assessment.
Full-text not available	Articles that report the development and/or validation of a prediction model for which full-text is not accessible online.

Information sources

A literature search will be systematically applied in one major public-available electronic medical literature databases (PubMed) from 01 January 2018 to 31 December 2019.

Search strategy

For peer review only

For peer review only - http://bmjopen.bmj.com/site/about/guidelines.xhtml

The search strategy was built using keywords including 'machine learning', 'prediction', and several performance measures for prediction modelling. For search refinement, we selected 30 articles aligned with our inclusion/exclusion criteria to create a "golden bullet" set. This set was analyzed using SWIFT-Reviewer to obtain the most frequent words in the included articles by topic modelling.[24] In MedlinerRanker, the analysis of the included and excluded golden bullets articles allowed us to obtain the most discriminative words to be considered in the search strategy.[25] The final search strategy is presented in supplemental file 1.

Study records

Data management

Study record information including title and abstract from the searched online database will be imported into Endnote Citation Manager and Rayyan systematic review software.[26] These platforms will track and backup all activities when authors conduct the literature review process. Once eligible studies are identified, full-text articles will be downloaded for full-text screening and data extraction. Data items (below) will be extracted from the final included studies for review using Research Data Capture (REDCap) software.[27]

Selection process

Two researchers, from a combination of seven (CLAN, TT, SWJN, PD, JM, RB, JAAD) will independently screen the titles and abstracts to identify eligible studies according to the eligibility criteria. Two independent researchers, from the combination of the previous seven reviewers, will review the full text for potentially eligible articles; one researcher (CLAN) will screen all articles and six researchers (TT, SWJN, PD, JM, RB, JAAD) will collectively screen a portion of the same articles for agreement. Disagreements between reviewers will be solved by consensus or consultation with a third investigator, if necessary (JAAD). The study flow will be presented in a PRISMA flowchart.[28]

Data collection process

For peer review only

For peer review only - http://bmjopen.bmj.com/site/about/guidelines.xhtml

We will perform a double data extraction for all included articles. Two reviewers will independently extract data from each article using a standardized data extraction form. One researcher (CLAN) will extract data from all articles and six other researchers (TT, SWJN, PD, JM, RB, JAAD) will collectively extract data from the same articles. The data extraction form will be piloted on five papers and amended if necessary. Disagreements in data extraction will be discussed between the two reviewers, and adjudicated by a third reviewer (KGMM, GSC, RDR or LH), if necessary. The authors of the articles will be contacted for further information and clarification if needed. Data and records will be maintained by the lead investigator (CLAN) and stored on a shared secure platform for access by all investigators (REDCap).

Data items

Data to be extracted will be informed by TRIPOD using the TRIPOD data extraction adherence guidance, PROBAST and the CHecklist for critical Appraisal and data extraction for systematic Reviews of prediction Modelling Studies (CHARMS).[15-18,22,29] Additional items specifically relating to ML techniques for prediction model purposes, will also be extracted.

Extracted data will include study design for the development and validation of the model, outcomes to be predicted, setting, the intended use of the prediction model, study population, data source, patient characteristics, total study sample size, number of individuals with the outcome, number of predictors (candidate and final), internal validation type, predictive performance measures (discrimination and calibration), number of models developed, and the details of the machine learning technique used to develop each model (e.g. technique, pre-processing, data cleaning, optimization algorithm, predictors selection, penalization techniques, hyperparameters, code, and data availability, etc.).

Outcomes

The primary outcomes of this systematic review are the adherence to the TRIPOD reporting guideline and the risk of bias assessed using PROBAST.[17,18,22]

Assessment of risk of bias

For peer review only

For peer review only - http://bmjopen.bmj.com/site/about/guidelines.xhtml

The risk of bias of individual studies is one of our outcomes of interest and will be assessed using PROBAST.[17,18]

Data synthesis

We will conduct a narrative synthesis of the extracted data. Data will be summarized using descriptive statistics and visual plots. Numbers and percentages will be used to describe categorical data about the reporting, methodological conduct, and risks of bias of the studies. The distribution of continuous data, such as sample size and the number of predictors, will be assessed and described using mean and standard deviation for normally distributed data and using median and 25th and 75th percentiles for non-normally distributed data. The risk of bias assessment will be summarized and graphically presented for each PROBAST domain and as an overall risk of bias judgement. Results will be stratified by study type (development with internal validation and/or external validation), medical field, and prevalent ML techniques.

Meta-bias(es)

Meta-bias will not be investigated in this study.

Confidence in cumulative evidence

The strength of the body of evidence will not be assessed in this study.

Amendments

Protocol amendments will be listed and made available on the PROSPERO registration. The date, description, and rationale will be given for each amendment.

Dissemination

The findings of this systematic review will be published in an open-access journal to ensure access for all stakeholders and disseminated in various scientific conferences.

DISCUSSION

For peer review only

The use of ML has been increasingly recognized as a powerful tool to improve healthcare by enabling related professionals to make decisions based on the increasingly available and diverse sources of (bio)medical data. Particularly, ML based prediction algorithms are considered the key to unlock the increasingly available data sources, are intended to better inform real-time clinical decisions, support early-warning systems, and provide superhuman imaging diagnostics.[30] However, published research about this topic rarely provides adequate information about the final predictive model, and its estimates and performance. Even more scarce is research where the prediction model is accessible for patients and healthcare professionals alike. Hence, ML based prediction model studies are often seen as a black box. This black box aspect of ML techniques is problematic especially in medical diagnosis and prognosis, hampering the judgement of quality, clinical acceptance, and implementation.

At present, there is a limited number of systematic reviews regarding the reporting and methodological quality of ML based prediction model studies and their risks of bias.[31,32,33] In this systematic review, we will review across all medical fields, the current use of ML techniques in prediction model development, validation and updating studies, the methodological conduct and risks of bias of using PROBAST, and the adherence to the reporting guideline for such studies using TRIPOD. Particularly, we will assess the extent to which risks of bias and reporting of ML based prediction model studies match the current recommendations from TRIPOD and PROBAST,[22] and the implications of these results to update or extend them to TRIPOD-ML and PROBAST-ML.

So far, our findings should be considered within limitations. Machine Learning is a recently developed concept and without a clear scope yet. Therefore, a sensitive search strategy is hard to build, which may result in a large number of abstracts to screen at initial stages. Additionally, we are only able to include articles in English, which will underrepresent research available in other languages.

Footnotes

Acknowledgements

For peer review only

For peer review only - http://bmjopen.bmj.com/site/about/guidelines.xhtml

The authors would like to thank and acknowledge the support of Rene Spijker, information specialist.

Ethics approval and consent to participate: Not applicable.

Consent for publication: Not applicable.

Patient and public involvement: Not applicable.

Authors Contributions: The study concept and design were conceived by CLAN, JD, KGM, LH, PD, GSC, and RDR. CLAN, JD, TT, SN, PD, JM and RB will conduct article screening and data extraction. CLAN will perform data analysis. All authors drafted this manuscript, revised it for important content, and have provided the final approval of this version. CLAN, the corresponding author, is the guarantor of the review.

Funding: GSC is funded by the National Institute for Health Research (NIHR) Oxford Biomedical Research Centre (BRC) and by Cancer Research UK program grant (C49297/A27294). PD is funded by the NIHR Oxford BRC. The funder was not involved in the development of this protocol.

Competing interests: None declared.

Provenance and peer review: Not commissioned; externally peer-reviewed.

Data sharing statement: The final dataset that supports the findings of this study will be available from the corresponding author upon reasonable request.

Abbreviations: AI: Artificial Intelligence; ML: Machine Learning; TRIPOD: Transparent Reporting of multivariable prediction model for Individual Prognosis Or Diagnosis; PROBAST: Prediction model Risk Of Bias Assessment Tool.

REFERENCES

1. Moons KGM, Royston P, Vergouwe Y, *et al*. Prognosis and prognostic research: What, why, and how? *BMJ* 2009; doi:10.1136/bmj.b375.

2. Steyerberg EW, Moons KGM, van der Windt DA, *et al*. Prognosis Research Strategy (PROGRESS) 3: Prognostic

For peer review only - http://bmjopen.bmj.com/site/about/guidelines.xhtml

Model Research. *PLoS Med* 2013; doi:10.1371/journal.pmed.1001381.

3. Riley RD, van der Windt D, Croft P, Moons KGM. Prognosis Research in Healthcare: Concepts, Methods, and Impact. First ed. Oxford University Press; 2019.

4. Steyerberg EW. Clinical Prediction Models: A Practical Approach to Development, Validation, and Updating. Second ed. Cham, Switzerland: Springer; 2019

5. Damen JAAG, Hooft L, Schuit E, *et al*. Prediction models for cardiovascular disease risk in the general population: Systematic review. *BMJ* 2016; doi:10.1136/bmj.i2416.

6. Heus P, Damen JAAG, Pajouheshnia R, *et al*. Poor reporting of multivariable prediction model studies: Towards a targeted implementation strategy of the TRIPOD statement. *BMC Med* 2018; doi:10.1186/s12916-018-1099-2.

7. Collins GS, de Groot JA, Dutton S, *et al*. External validation of multivariable prediction models: a systematic review of methodological conduct and reporting. *BMC Med Res Methodol* 2014; doi:10.1186/1471-2288-14-40.

8. Bouwmeester W, Zuithoff NPA, Mallett S, *et al*. Reporting and methods in clinical prediction research: A systematic review. *PLoS Med* 2012; doi:10.1371/journal.pmed.1001221

9. Collins GS, Mallet S, Omar O, *et al*. Developing risk prediction models for type 2 diabetes: a systematic review of methodology and reporting. BMC med. 2011; doi:10.1186/1741-9-103.

10. Wen Z, Guo Y, Xu B, *et al*. Developing risk prediction models for postoperative pancreatic fistula: a systematic review of methodology and reporting quality. *Indian J Surg* 2016; doi: 10.1007/s12262-015-1439-9

11. Mitchell TM. Machine Learning. New York, NY: McGraw Hill; 1997.

12. Bi Q, Goodman KE, Kaminsky J, *et al*. What is Machine Learning? A Primer for the Epidemiologist. *Am J Epidemiol* 2019; doi: 10.1093/aje/kwz189.

13. Sidey-Gibbons JAM, Sidey-Gibbons CJ. Machine learning in medicine: a practical introduction. *BMC Med Res Methodol* 2019; doi: 10.1186/s12874-019-0681-4.

Beam AL, Kohane IS. Big Data and Machine Learning in Health Care. *JAMA* 2018; doi: 10.1001/jama.2017.18391.

14. Harrell FE Jr, Lee KL, Mark DB. Multivariable prognostic models: issues in developing models, evaluating assumptions and adequacy, and measuring and reducing errors. *Stat Med* 1996; doi: 10.1002/(SICI)1097-0258(19960229)15:4<361::AID-SIM168>3.0.CO;2.-4.

15. Collins GS, Reitsma JB, Altman DG, *et al*. Transparent Reporting of a multivariable prediction model for Individual Prognosis Or Diagnosis (TRIPOD): The TRIPOD Statement. *Ann Intern Med* 2015 doi:10.7326/M14-069717.

16. Moons KGM, Altman DG, Reitsma JB, et al. Transparent reporting of a multivariable prediction model for individual prognosis or diagnosis (TRIPOD): Explanation and elaboration. *Ann Intern Med* 2015; doi:10.7326/M14-0698

17. Wolff RF, Moons KGM, Riley RD; PROBAST Group. PROBAST: A tool to assess the risk of bias and applicability of prediction model studies. *Ann Intern Med* 2019; doi:10.7326/M18-1376

18. Moons KGM, Wolff RF, Riley RD, *et al*. PROBAST: A tool to assess risk of bias and applicability of prediction model studies: Explanation and elaboration. *Ann Intern Med* 2019; doi:10.7326/M18-1377

For peer review only

For peer review only - http://bmjopen.bmj.com/site/about/guidelines.xhtml

19. Liu Y, Chen PC, Krause J, *et al*. How to read articles that use Machine Learning: Users' guides to the medical literature. *JAMA* 2019; doi: 10.1001/jama.2019.16489.

20. CONSORT-AI and SPIRIT-AI Steering Group. Reporting guidelines for clinical trials evaluating artificial intelligence interventions are needed. *Nat Med* 2019; doi: 10.1038/s41591-019-0603-3.

21. Collins GS, Moons KGM. Reporting of artificial inteligence prediction models. *Lancet* 2019; doi: 10.1016/S0140-6736(19)30037-6.

22. Heus P, Damen JAAG, Pajouheshnia R, *et al*. Uniformity in measuring adherence to reporting guidelines: the example of TRIPOD for assessing completeness of reporting of prediction model studies. *BMJ open* 2019;9(4): e025611

23. Moher D, Shamseer L, Clarke M, *et al*. Preferred Reporting Items for Systematic Review and Meta-Analysis Protocols (PRISMA-P) 2015 statement. *Syst Rev* 2015; doi: 10.1186/2046-4053-4-1.

24. Howard BE, Phillips J, Miller K, et al. SWIFT-Review: a text-mining workbench for systematic review. *Syst Rev* 2016; doi: 10.1186s/s13643-016-0263-z.

25. Fontaine JF, Barbosa-Silva A, Schaefer M, *et al*. MedlineRanker: flexible ranking of biomedical literature. *Nucleic Acids Res* 2009; doi: 10.1093/nar/gkp353.

26. Ouzzani M, Hammady H, Fedorowicz Z, *et al*. Rayyan- a web and mobile app for systematic reviews. *Syst Rev* 2016; doi: 0.1186/s13643-016-0384-4.

27. Harris PA, Taylor R, Thielker R, *et al*. Research electronic data capture (REDCap). A metadata-drien methodology and workflow process for providing translational research informatics support. *J Biomed Inform* 2009; doi: 10.1016/j.jbi.2008.08.010.

28. Liberati A, Altman DG, Tetzlaff J, *et al*. The PRISMA Statement for Reporting Systematic Reviews and Meta-Analyses of Studies That Evaluate Health Care Interventions: Explanation and Elaboration. *Ann Intern Med* 2009; doi: 10.1371/journal.pmed.1000100.

29. Moons KGM, de Groot JAH, Bouwmeester W, *et al*. Critical Appraisal and Data Extraction for Systematic Reviews of Prediction Modelling Studies: The CHARMS Checklist. *PLoS Med* 2014; doi:10.1371/journal.pmed.1001744.

30. Chen JH, Asch SM. Machine Learning and Prediction in Medicine - Beyond the peak of inflated expectations. *N Engl J Med* 2018; doi: 10.1056/NEJMp1702071.

31. Christodoulou E, Ma J, Collins GS, et al. A systematic review shows no performance benefit of machine learning over logistic regression for clinical prediction models. *J Clin Epidemiol* 2019; doi: 10.1016/j.jclinepi.2019.02.004.

32. Shillan D, Sterne JAC, Champneys A, *et al*. Use of machine learning to analyse routinely collected intensive care unit data: a systematic review. *Crit Care* 2019; doi: 10.1186/s13054-019-2564-9.

33. Wang W, Kiik M, Peek N, *et al*. A systematic review of Machine Learning models for predicting outcomes of stroke with structured data. *Lancet* 2020. Published online first at SSRN: https://ssrn.com/abstract=3520073.

For peer review only

Appendix 1

PubMed search strategy

Draft for the search strategy for systematic review of the reporting of the development and validation of machine learning models using Pubmed interface.

Search date: 19 November 2019

1. Machine Learning[MeSH Terms]
2. Deep learning[MeSH Terms]
3. supervised machine learning[MeSH Terms]
4. "Neural Networks, Computer"[Mesh]
5. data mining[MeSH Terms]
6. machine[tiab] AND (learn* OR model*)
7. (statistical[tiab] OR "statistical-learning"[tiab]) AND (strateg*[tiab])
8. multilayer perceptron*[tiab] OR random forest*[tiab] OR bayes* network*[tiab] OR support vector machine*[tiab] OR nearest neighbor*[tiab] OR k nearest neighbor*[tiab] OR elastic net[tiab] OR naive bayes*[tiab]
9. (classification[tiab] OR regression[tiab] OR estimation[tiab] OR decision[tiab]) AND tree[tiab]
10. ridge[tiab] OR kernel[tiab] OR ensemble[tiab] OR bagging[tiab] OR bagged[tiab] OR boosting[tiab] OR boosted[tiab] OR fuzzy[tiab]
11. #1 OR #2 OR #3 OR #4 OR #5 OR #6 OR #7 OR #8 OR #9 OR #10
12. (Validat* OR Predict* OR Rule*). [tiab]
13. (Predict* AND (Outcome* OR Risk* OR Model*). [tiab]
14. ((History OR Variable* OR Criteria OR Scor* OR Characteristic* OR Finding* OR Factor*) AND (Predict* OR Model* OR Decision* OR Identif* OR Prognos*)). [tiab]
15. (Decision* AND (Model* OR Clinical*). [tiab]
16. (Prognostic AND (History OR Variable* OR Criteria OR Scor* OR Characteristic* OR Finding* OR Factor* OR Model*). [tiab]
17. #12 OR #13 OR #14 OR #15 OR #16
18. (discrimination[tiab] OR discriminative[tiab] OR discriminatory[tiab]) AND (accuracy[tiab] OR ability[tiab] OR performance[tiab] OR value[tiab] OR model[tiab] OR models[tiab] OR power[tiab] OR capacity[tiab] OR capabilit*[tiab] OR efficiency[tiab])
19. (discriminability[tiab] OR c-index[tiab] OR c-statistic[tiab] OR concordance[tiab] OR

For peer review only

DCA[tiab])

20. "decision curve"[tiab]

21. calibrat*[tiab] AND (plot*[tiab] OR curve*[tiab] OR slope*[tiab] OR model[tiab] OR models[tiab])

22. performance[tiab] AND (classification[tiab] OR classifier[tiab] OR clinical[tiab] OR accuracy[tiab] OR validation[tiab] OR metrics[tiab] OR diagnostic[tiab] OR AUC[tiab])

23. (sensitivity[tiab] OR specificity[tiab] OR PPV[tiab] OR NPV[tiab])

24. "correctly classified"[tiab]

25. "clinical accuracy"[tiab]

26. positive predictive value*[tiab]

27. negative predictive value*[tiab]

28. classification[tiab] OR classifier[tiab]

29. Area Under Curve[Mesh]

30. "Area under the curve"[tiab]

31. "Area under the ROC"[tiab]

32. “Area Under the Receiver”[tiab]

33. (ROC[tiab] OR AUC[tiab] OR AUROC[tiab])

34. ROC Curve [Mesh]

35. "Hosmer-Lemeshow"[tiab] OR "H-L test"[tiab]

36. "expected ratio"[tiab] OR "observed ratio"[tiab] OR "E:O ratio"[tiab]

37. #18 OR #19 OR #20 OR #21 OR #22 OR #23 OR #24 OR #25 OR #26 OR #27 OR #28 OR #29 OR #30 OR #31 OR #32 OR #33 OR #34 OR #35 OR #36

---

38. #11 AND #17

39. #11 AND (#17 OR #37)

40. #39 AND (“2018/01/01”[PDat]: “2019/12/31”[PDat])

41. #40 NOT “review”[pt]

42. #39 AND (“2019/01/01”[PDat]: “2019/12/31”[PDat])

43. #42 NOT “review”[pt]

Results #41= **24732**

For peer review only

Results #43=**12977**

For peer review only

Reporting checklist for protocol of a systematic review.

Based on the PRISMA-P guidelines.

Instructions to authors

Complete this checklist by entering the page numbers from your manuscript where readers will find each of the items listed below.

Your article may not currently address all the items on the checklist. Please modify your text to include the missing information. If you are certain that an item does not apply, please write "n/a" and provide a short explanation.

Upload your completed checklist as an extra file when you submit to a journal.

In your methods section, say that you used the PRISMA-Preporting guidelines, and cite them as:

Moher D, Shamseer L, Clarke M, Ghersi D, Liberati A, Petticrew M, Shekelle P, Stewart LA. Preferred Reporting Items for Systematic Review and Meta-Analysis Protocols (PRISMA-P) 2015 statement. Syst Rev. 2015;4(1):1.

		Reporting Item	Page Number
**Title**			
Identification	#1a	Identify the report as a protocol of a systematic review	1
Update	#1b	If the protocol is for an update of a previous systematic review, identify as such	NA
**Registration**			
	#2	If registered, provide the name of the registry (such as PROSPERO) and registration number	2
**Authors**			
Contact	#3a	Provide name, institutional affiliation, e-mail address of all protocol authors; provide physical mailing address of corresponding author	1
Contribution	#3b	Describe contributions of protocol authors and identify the	11-12

For peer review only

		guarantor of the review	
**Amendments**			
	#4	If the protocol represents an amendment of a previously completed or published protocol, identify as such and list changes; otherwise, state plan for documenting important protocol amendments	10
**Support**			
Sources	#5a	Indicate sources of financial or other support for the review	12
Sponsor	#5b	Provide name for the review funder and / or sponsor	12
Role of sponsor or funder	#5c	Describe roles of funder(s), sponsor(s), and / or institution(s), if any, in developing the protocol	12
**Introduction**			
Rationale	#6	Describe the rationale for the review in the context of what is already known	4-5
Objectives	#7	Provide an explicit statement of the question(s) the review will address with reference to participants, interventions, comparators, and outcomes (PICO)	5
**Methods**			
Eligibility criteria	#8	Specify the study characteristics (such as PICO, study design, setting, time frame) and report characteristics (such as years considered, language, publication status) to be used as criteria for eligibility for the review	6-7
Information sources	#9	Describe all intended information sources (such as electronic databases, contact with study authors, trial registers or other grey literature sources) with planned dates of coverage	7
Search strategy	#10	Present draft of search strategy to be used for at least one electronic database, including planned limits, such that it could be repeated	7-8
Study records - data management	#11a	Describe the mechanism(s) that will be used to manage records and data throughout the review	8
Study records -	#11b	State the process that will be used for selecting studies (such	8-9

For peer review only

selection process		as two independent reviewers) through each phase of the review (that is, screening, eligibility and inclusion in meta-analysis)	
Study records - data collection process	#11c	Describe planned method of extracting data from reports (such as piloting forms, done independently, in duplicate), any processes for obtaining and confirming data from investigators	8-9
Data items	#12	List and define all variables for which data will be sought (such as PICO items, funding sources), any pre-planned data assumptions and simplifications	9
Outcomes and prioritization	#13	List and define all outcomes for which data will be sought, including prioritization of main and additional outcomes, with rationale	9
Risk of bias in individual studies	#14	Describe anticipated methods for assessing risk of bias of individual studies, including whether this will be done at the outcome or study level, or both; state how this information will be used in data synthesis	9
Data synthesis	#15a	Describe criteria under which study data will be quantitatively synthesised	9-10
Data synthesis	#15b	If data are appropriate for quantitative synthesis, describe planned summary measures, methods of handling data and methods of combining data from studies, including any planned exploration of consistency (such as I2, Kendall's τ)	9-10
Data synthesis	#15c	Describe any proposed additional analyses (such as sensitivity or subgroup analyses, meta-regression)	10
Data synthesis	#15d	If quantitative synthesis is not appropriate, describe the type of summary planned	9-10
Meta-bias(es)	#16	Specify any planned assessment of meta-bias(es) (such as publication bias across studies, selective reporting within studies)	10
Confidence in cumulative evidence	#17	Describe how the strength of the body of evidence will be assessed (such as GRADE)	10

For peer review only

The PRISMA-P checklist is distributed under the terms of the Creative Commons Attribution License CC-BY 4.0. This checklist was completed on 06. March 2020 using https://www.goodreports.org/, a tool made by the EQUATOR Network in collaboration with Penelope.ai

For peer review only

Protocol for a systematic review on the methodological and reporting quality of prediction model studies using machine learning techniques

Journal:	*BMJ Open*
Manuscript ID	bmjopen-2020-038832.R1
Article Type:	Protocol
Date Submitted by the Author:	09-Jul-2020

Complete List of Authors:	Andaur Navarro, Constanza L.; Julius Center for Health Sciences and Primary Care, University Medical Center Utrecht, Utrecht University; Cochrane Netherlands, University Medical Center Utrecht, Utrecht University<br>Damen, Johanna; Julius Center for Health Sciences and Primary Care, University Medical Center Utrecht, Utrecht University ; Cochrane Netherlands, University Medical Center Utrecht, Utrecht University<br>Takada, Toshihiko; Julius Center for Health Sciences and Primary Care, University Medical Center Utrecht, Utrecht University<br>Nijman, Steven W.J. ; Julius Center for Health Sciences and Primary Care, University Medical Center Utrecht, Utrecht University<br>Dhiman, Paula; Center for Statistics in Medicine, University of Oxford<br>Ma, Jie; Center for Statistics in Medicine, University of Oxford<br>Collins, Gary; Centre for Statistics in Medicine, University of Oxford<br>Bajpai, Ram; School of Primary, Community and Social Care, Keele Univeristy<br>Riley, Richard; School of Primary, Community and Social Care, Keele University<br>Moons, Karel; Julius Center for Health Sciences and Primary Care, University Medical Center Utrecht, Utrecht University; Cochrane Netherlands, University Medical Center Utrecht, Utrecht University<br>Hooft, Lotty; Julius Center for Health Sciences and Primary Care, University Medical Center Utrecht, Utrecht University; Cochrane Netherlands, University Medical Center Utrecht, Utrecht University

**Primary Subject Heading**:	Epidemiology
Secondary Subject Heading:	Research methods
Keywords:	EPIDEMIOLOGY, STATISTICS & RESEARCH METHODS, PREVENTIVE MEDICINE

SCHOLARONE™ Manuscripts

I, the Submitting Author has the right to grant and does grant on behalf of all authors of the Work (as defined in the below author licence), an exclusive licence and/or a non-exclusive licence for contributions from authors who are: i) UK Crown employees; ii) where BMJ has agreed a CC-BY licence shall apply, and/or iii) in accordance with the terms applicable for US Federal Government officers or employees acting as part of their official duties; on a worldwide, perpetual, irrevocable, royalty-free basis to BMJ Publishing Group Ltd ("BMJ") its licensees and where the relevant Journal is co-owned by BMJ to the co-owners of the Journal, to publish the Work in this journal and any other BMJ products and to exploit all rights, as set out in our licence.

The Submitting Author accepts and understands that any supply made under these terms is made by BMJ to the Submitting Author unless you are acting as an employee on behalf of your employer or a postgraduate student of an affiliated institution which is paying any applicable article publishing charge ("APC") for Open Access articles. Where the Submitting Author wishes to make the Work available on an Open Access basis (and intends to pay the relevant APC), the terms of reuse of such Open Access shall be governed by a Creative Commons licence – details of these licences and which Creative Commons licence will apply to this Work are set out in our licence referred to above.

Other than as permitted in any relevant BMJ Author's Self Archiving Policies, I confirm this Work has not been accepted for publication elsewhere, is not being considered for publication elsewhere and does not duplicate material already published. I confirm all authors consent to publication of this Work and authorise the granting of this licence.

For peer review only

Original Article

Protocol for a systematic review on the methodological and reporting quality of prediction model studies using machine learning techniques

Constanza L Andaur Navarro[1,2] (c.l.andaurnavarro@umcutrecht.nl), Johanna A A Damen[1,2] (j.a.a.damen@umcutrecht.nl), Toshihiko Takada[1] (t.takada-3@umcutrecht.nl), Steven W J Nijman[1] (S.W.J.Nijman@umcutrecht.nl), Paula Dhiman[3] (paula.dihman@ndorms.ox.ac.uk), Jie Ma[3] (jie.ma@csm.ox.ac.uk), Gary S Collins[3] (gary.collins@csm.ox.ac.uk), Ram Bajpai[4] (r.bajpai@keele.ac.uk), Richard D Riley[4] (r.riley@keele.ac.uk), Karel GM Moons[1,2] (k.g.m.moons@umcutrecht.nl), Lotty Hooft[1,2] (l.hooft@umcutrecht.nl)

To be submitted to BMJ Open

Keywords: protocol, systematic review, machine learning, prediction, diagnosis, prognosis, TRIPOD, PROBAST, risk of bias, applicability.

Correspondence to: Constanza L Andaur Navarro, Julius Center for Health Sciences and Primary Care, Universiteitsweg 100, P.O. Box 85500, 3508 GA, Utrecht, The Netherland, +31 8875 68640. c.l.andaurnavarro@umcutrecht.nl

[1]Julius Center for Health Sciences and Primary Care, University Medical Center Utrecht, Utrecht University, Utrecht, The Netherlands.

[2]Cochrane Netherlands, University Medical Center Utrecht, Utrecht University, Utrecht, The Netherlands.

[3]Center for Statistics in Medicine, University of Oxford, Oxford, United Kingdom.

[4]School of Primary, Community and Social Care, Keele University, Keele, United Kingdom.

For peer review only - http://bmjopen.bmj.com/site/about/guidelines.xhtml

ABSTRACT

Introduction. Studies addressing the development and/or validation of diagnostic and prognostic prediction models are abundant in most clinical domains. Systematic reviews have shown that the methodological and reporting quality of prediction model studies is suboptimal. Due to the increasing availability of larger, routinely collected and complex medical data, and the rising application of Artificial Intelligence (AI) or Machine Learning (ML) techniques, the number of prediction model studies is expected to increase even further. Prediction models developed using AI or ML techniques are often labeled as a "black box" and little is known about their methodological and reporting quality. Therefore, this comprehensive systematic review aims to evaluate the reporting quality, the methodological conduct, and the risk of bias of prediction model studies that applied ML techniques for model development and/or validation.

Methods and Analysis. A search will be performed in PubMed to identify studies developing and/or validating prediction models using any ML methodology and across all medical fields. Studies will be included if they were published between January 2018 and December 2019, predict patient-related outcomes, use any study design or data source, and available in English. Screening of search results and data extraction from included articles will be performed by two independent reviewers. The primary outcomes of this systematic review are: (1) the adherence of ML based prediction model studies to the Transparent Reporting of a multivariable prediction model for individual prognosis or diagnosis (TRIPOD), and (2) the risk of bias in such studies as assessed using the Prediction model Risk Of Bias ASsessment Tool (PROBAST). A narrative synthesis will be conducted for all included studies. Findings will be stratified by study type, medical field, and prevalent machine learning methods, and will inform necessary extensions or updates of TRIPOD and PROBAST to better address prediction model studies that used AI or ML techniques.

Ethics and dissemination. Ethical approval is not required for this study because only available published data will be analyzed. Findings will be disseminated through peer-reviewed publications and scientific conferences.

Systematic review registration: PROSPERO, CRD42019161764.

For peer review only - http://bmjopen.bmj.com/site/about/guidelines.xhtml

For peer review only

ARTICLE SUMMARY

Strengths and Limitation of this study

- This protocol increases transparency to the methods and definitions that we used in our review and are applied to develop prediction model studies using Artificial Intelligence or Machine Learning.
- The systematic review will provide an overview and critical appraisal of the methodological and reporting quality, and risk of bias of prediction model studies using Machine Learning.
- The findings of the review will provide the needed evidence for the development of tailored methodological and reporting guidelines for prediction model studies based on Machine Learning techniques.
- We will build a sensitivity search strategy by using terms related to Machine Learning techniques, as well, as conventional prediction techniques.
- Language restriction to English might exclude additional studies published in other languages.

For peer review only

For peer review only - http://bmjopen.bmj.com/site/about/guidelines.xhtml

INTRODUCTION

Clinical prediction models aim to estimate the individualized probability that a particular outcome, e.g. condition or disease, is present (diagnostic models) or whether a specific outcome will occur in the future (prognostic models).[1-4] Studies addressing the development, validation, and updating of prediction models are abundant in most clinical domains. For example, in cardiovascular disease, more than 350 prediction models have been developed and only a few have been validated.[5] Moreover, systematic reviews have shown that, within different medical domains, the methodological and reporting quality of prediction model studies is suboptimal.[6-10] Due to the increasing availability of larger, routinely collected and complex medical data, and the rising application of Artificial Intelligence (AI) or Machine Learning (ML) techniques for clinical prediction, the number of prediction model studies is expected to increase even further.

Machine Learning can be described as techniques that directly and automatically learn from data without being explicitly programmed for that task, and often without any prior assumption.[11,12,13] Thus ML relies on patterns and inferences from the data itself. A perceived advantage of ML over conventional statistical techniques is its ability to analyze "big", non-linear and high dimensional data, and thus its ability to model complex associations and scenarios. Due to the novelty, diversity, flexibility, and complexity of ML techniques, ML based prediction model studies are often considered as uninterpretable for many users. Inadequate reporting of, e.g. data sources, study design, modeling processes, number of predictors, and other data assumptions, makes prediction models developed with ML techniques published in medical journals difficult to interpret and to be validated by other researchers, creating barriers to their use in daily clinical practice.

Complete reporting is essential to judge the validity of any prediction model as it facilitates: study replication, independent validation of the prediction model, risk of bias assessments, interpretation of the results, meta-analysis of prediction models, and the judgment of the value and applicability of such model in real clinical settings for individualized predictions.[14] While complete reporting reveals the strengths and limitations of a prediction model, it also enhances the use and implementation of prediction model in clinical practice. The "Transparent Reporting

For peer review only - http://bmjopen.bmj.com/site/about/guidelines.xhtml

of a multivariable prediction model for Individual Prognosis Or Diagnosis (TRIPOD)” statement has been available since 2015, providing a checklist of 22 items considered essential for informative reporting of diagnostic or prognostic prediction model studies.[15,16] Similarly, the Prediction model Risk Of Bias ASsessment Tool (PROBAST) was published in 2019 to guide the critical appraisal of prediction model studies.[17,18] PROBAST provides signaling questions to facilitate both the applicability and risk of bias assessment of prediction model studies across four domains: participants, predictors, outcome, and analysis. This assessment can only be correctly implemented if prediction model studies are properly reported. Although TRIPOD and PROBAST both covered all types of prediction modelling studies, including those using ML techniques, their focus was on regression-based modeling. The challenges and necessity for reporting and quality assessment guidelines in the AI/ML field has been addressed by several authors and this has led to initiatives such as, CONSORT-AI (for randomized controlled trials), and SPIRIT-AI (for clinical trial protocols). Similarly, for prediction model studies using ML, TRIPOD-ML and PROBAST-ML have been announced.[19-21]

To improve the quality, transparency, and usability of ML based prediction models in medicine, it is important to explore the current use and reporting of ML techniques in prediction model studies, to evaluate the methodological conduct and risk of bias using PROBAST, and assess the adherence to TRIPOD by performing a comprehensive systematic review.[3,15-18, 22]

Study aim

The primary aim of this systematic review is to evaluate the reporting and the methodological conduct of studies reporting on prediction models developed with supervised ML techniques, across all medical fields. Specific objectives are to:

1. Evaluate the reporting quality of prediction models developed using ML techniques based on TRIPOD.
2. Assess the methodological quality and the risks of bias in prediction model development or validation studies using ML techniques based on PROBAST.

For peer review only - http://bmjopen.bmj.com/site/about/guidelines.xhtml
For peer review only

3. Identify key and emerging concepts for the development of tailored adaptations or extensions of both TRIPOD and PROBAST.

METHODS AND ANALYSIS

Our systematic review protocol was registered with the International Prospective Register of Systematic Reviews (PROSPERO) on 19 December 2019 (CRD42019161764). This protocol was prepared using the Preferred Reporting Items for Systematic Reviews and Meta-Analysis Protocols (PRISMA-P) 2015 statement.[23]

Eligibility criteria

Articles will be eligible for this review when describing primary studies on the development and/or validation of a multivariable diagnostic or prognostic prediction model with at least 2 predictors, using any supervised ML methodology within all medical fields, and published between January 2018 and December 2019. This last inclusion criterion is to obtain the most contemporary sample of articles that would reflect the current practices of applied methods in the ML prediction model field to date. We will include studies with any study design and data source, all patient-related health outcomes, all outcome formats, and restricted to humans only. Further details about inclusion criteria are given in Table 1.

Table 1 Definition of inclusion criteria

Inclusion criteria	Definition
Any study design	Articles that report the development and/or validation of a prediction model based on experimental studies or observational studies. This includes randomized controlled trials, prospective and, retrospective cohort, case-control studies, and case-cohort studies.
Using at least 2 predictors for risk prediction	Articles that report the development and/or validation of a prediction model using at least 2 predictors. Articles that use imaging or speech parameters as structured data plus other predictors such as e.g. clinical, demographics, histological and genetic risk scores features will be included.
Any data sources	Articles that report the development and/or validation of a prediction model using any structured data source, e.g. electronic medical records, claims data, and individual patient data meta-analysis data.
Any supervised ML technique	Articles that report the use of any ML technique for development and/or validation of a prediction model. We will consider as a ML technique, a statistical

For peer review only - http://bmjopen.bmj.com/site/about/guidelines.xhtml

	technique based on advanced computational capacity and lower human intervention. More specifically, we will focus on supervised ML technique.
Patient health-related outcomes	Articles that report the development and/or validation of a prediction model whose main outcome is on an individual patient level. We will include articles assessing diagnosis, prognosis, and health services performance, such as length of stay or triage assessment.
All outcome measures format	Articles that report the development and/or validation of a prediction model whose main outcome has one of the following formats: continuous, binary, ordinal, multinomial, and time-to-event.

Articles will be excluded from this review when reporting models that make predictions for enhancing the reading of images or signals (rather than for prediction of health outcomes in individuals) or, use only genetic or molecular markers as candidate predictors. Furthermore, prognostic factor studies, secondary research, conference abstracts, and studies for which no full-text is available will also be excluded. The search will be restricted to articles available in English only. Further details about exclusion criteria are given in Table 2.

Table 2 Definition of exclusion criteria

Exclusion criteria	Definition
Images or signal studies	Articles that report the development and/or validation of a prediction model for enhancing the reading of images, pathological samples, or signals. The purpose of this articles is to improve the accuracy of an instrument rather than providing a clinical outcome.
Only genetic and/or molecular predictors	Articles that report the development and/or validation of a prediction model using only genetic and/or molecular candidate predictors. These articles are often based on high-dimensional data and unsupervised ML techniques.
Prognostic factors studies	Articles that report the identification of prognostic factors associated with a clinical outcome in an individual.
Secondary research	Articles that report narrative reviews, systematic reviews about prediction model studies in a specific medical field. Guidelines, expert’s opinions. and letters to the editor will also be excluded.
Conference abstract	Articles that report the development and/or validation of a prediction model presented in a conference. Such articles, by definition, do not report all the information required for assessment.
Full-text not available	Articles that report the development and/or validation of a prediction model for which full-text is not accessible online.

For peer review only

For peer review only - http://bmjopen.bmj.com/site/about/guidelines.xhtml

Information sources

A literature search will be systematically applied in one major public-available electronic medical literature databases (PubMed) from 01 January 2018 to 31 December 2019.

Search strategy

The search strategy was built using keywords including ML-related terms (i.e. 'supervised learning', 'support vector machine', 'neural network'), prediction-related terms[24] (i.e. 'risk', 'prognosis'), and several performance measures for prediction modelling (i.e. 'AUC', 'O:E ratio'). For search refinement, we selected 30 articles aligned with our inclusion/exclusion criteria to create a "golden bullet" set. This set was analyzed using SWIFT-Reviewer to obtain the most frequent words in the included articles by topic modelling.[25] In MedlinerRanker, the analysis of the included and excluded golden bullets articles allowed us to obtain the most discriminative words to be considered in the search strategy.[26] The final search strategy is presented in supplemental file 1.

Study records

Data management

Study record information including title and abstract from the searched online database will be imported into Endnote Citation Manager and Rayyan systematic review software.[27] These platforms will track and backup all activities when authors conduct the literature review process. Once eligible studies are identified, full-text articles will be downloaded for full-text screening and data extraction. Data items (below) will be extracted from the final included studies for review using Research Data Capture (REDCap) software.[28]

Selection process

Two researchers, from a combination of seven (CLAN, TT, SWJN, PD, JM, RB, JAAD) will independently screen the titles and abstracts to identify eligible studies according to the eligibility criteria. Two independent researchers, from the combination of the previous seven reviewers, will review the full text for potentially eligible articles; one researcher (CLAN) will

For peer review only - http://bmjopen.bmj.com/site/about/guidelines.xhtml

For peer review only

screen all articles and six researchers (TT, SWJN, PD, JM, RB, JAAD) will collectively screen a portion of the same articles for agreement. Disagreements between reviewers will be solved by consensus or consultation with a third investigator, if necessary (JAAD). The study flow will be presented in a PRISMA flowchart.[29]

Data collection process

We will perform a double data extraction for all included articles. Two reviewers will independently extract data from each article using a standardized data extraction form. One researcher (CLAN) will extract data from all articles and six other researchers (TT, SWJN, PD, JM, RB, JAAD) will collectively extract data from the same articles. The data extraction form will be piloted on five papers and amended if necessary. Disagreements in data extraction will be discussed between the two reviewers, and adjudicated by a third reviewer (KGMM, GSC, RDR or LH), if necessary. The authors of the articles will be contacted for further information and clarification if needed. Data and records will be maintained by the lead investigator (CLAN) and stored on a shared secure platform for access by all investigators (REDCap).

Data items

Data to be extracted will be informed by TRIPOD using the TRIPOD data extraction adherence guidance, PROBAST and the CHecklist for critical Appraisal and data extraction for systematic Reviews of prediction Modelling Studies (CHARMS).[15-18,22,30] Additional items specifically relating to ML techniques for prediction model purposes, will also be extracted.

Extracted data will include study design for the development and validation of the model, outcomes to be predicted, setting, the intended use of the prediction model, study population, data source, patient characteristics, total study sample size, number of individuals with the outcome, number of predictors (candidate and final), internal validation type, predictive performance measures (discrimination and calibration), number of models developed, and the details of the machine learning technique used to develop each model (e.g. technique, pre-processing, data cleaning, optimization algorithm, predictors selection, penalization techniques, hyperparameters, code, and data availability, etc.). This form will contain instructions for the

For peer review only - http://bmjopen.bmj.com/site/about/guidelines.xhtml

reviewers on how to assess the models presented in the articles. For example, the number of models developed will be based on how many ML techniques were used, including if several hyperparameters are tuned. We will set a limit to the number of models for data extraction to 10. The number of predictors will be counted based on what is reported in the article and/or supplemental file. If not stated, the number of predictors will be reported as unclear. The final data extraction form is presented in supplemental file 2.

Outcomes

The primary outcomes of this systematic review are the adherence to the TRIPOD reporting guideline and the risk of bias assessed using PROBAST.[17,18,22]

Assessment of risk of bias

The risk of bias of individual studies is one of our outcomes of interest and will be assessed using PROBAST.[17,18]

Data synthesis

We will conduct a narrative synthesis of the extracted data. Data will be summarized using descriptive statistics and visual plots. Numbers and percentages will be used to describe categorical data about the reporting, methodological conduct, and risks of bias of the studies. The distribution of continuous data, such as sample size and the number of predictors, will be assessed and described using mean and standard deviation for normally distributed data and using median and 25th and 75th percentiles for non-normally distributed data. The risk of bias assessment will be summarized and graphically presented for each PROBAST domain and as an overall risk of bias judgement. Results will be stratified by study type (development with internal validation and/or external validation), medical field, and prevalent ML techniques.

Meta-bias(es)

Meta-bias will not be investigated in this study.

Confidence in cumulative evidence

For peer review only

For peer review only - http://bmjopen.bmj.com/site/about/guidelines.xhtml

The strength of the body of evidence will not be assessed in this study.

Amendments

Protocol amendments will be listed and made available on the PROSPERO registration. The date, description, and rationale will be given for each amendment.

Ethics and Dissemination

Ethical approval is not required for this study because only available published data will be analyzed. The findings of this systematic review will be published in an open-access journal to ensure access for all stakeholders and disseminated in various scientific conferences.

Patient and public involvement: Not applicable.

Ethics approval and consent to participate: Not applicable.

Consent for publication: Not applicable.

DISCUSSION

The use of ML has been increasingly recognized as a powerful tool to improve healthcare by enabling related professionals to make decisions based on the increasingly available and diverse sources of (bio)medical data. Particularly, ML based prediction algorithms are considered the key to unlock the increasingly available data sources, are intended to better inform real-time clinical decisions, support early-warning systems, and provide superhuman imaging diagnostics.[31] However, published research about this topic rarely provides adequate information about the final predictive model, and its estimates and performance. Even more scarce is research where the prediction model is accessible for patients and healthcare professionals alike. Hence, ML based prediction model studies are often seen as uninterpretable. This aspect of ML techniques is problematic especially in medical diagnosis and prognosis, hampering the judgement of quality, clinical acceptance, and implementation.

At present, there is a limited number of systematic reviews regarding the reporting and methodological quality of ML based prediction model studies and their risks of bias.[32,33,34] In this

For peer review only - http://bmjopen.bmj.com/site/about/guidelines.xhtml

systematic review, we will review across all medical fields, the current use of ML techniques in prediction model development, validation and updating studies, the methodological conduct and risks of bias of using PROBAST, and the adherence to the reporting guideline for such studies using TRIPOD. Particularly, we will assess the extent to which risks of bias and reporting of ML based prediction model studies match the current recommendations from TRIPOD and PROBAST,[22] and the implications of these results to update or extend them to TRIPOD-ML and PROBAST-ML.

So far, our findings should be considered within limitations. Machine Learning is a recently developed concept and without a clear scope yet. Therefore, a sensitive search strategy is hard to build, which may result in a large number of abstracts to screen at initial stages. Additionally, we are only able to include articles in English, which will underrepresent research available in other languages.

Acknowledgements

The authors would like to thank and acknowledge the support of Rene Spijker, information specialist.

Authors Contributions: The study concept and design were conceived by CLAN, JD, KGMM, LH, PD, GSC, and RDR. CLAN, JD, TT, SN, PD, JM and RB will conduct article screening and data extraction. CLAN will perform data analysis. All authors drafted this manuscript, revised it for important content, and have provided the final approval of this version. CLAN, the corresponding author, is the guarantor of the review.

Funding: GSC is funded by the National Institute for Health Research (NIHR) Oxford Biomedical Research Centre (BRC) and by Cancer Research UK program grant (C49297/A27294). PD is funded by the NIHR Oxford BRC. The funder was not involved in the development of this protocol.

Competing interests: None declared.

Provenance and peer review: Not commissioned; externally peer-reviewed.

For peer review only - http://bmjopen.bmj.com/site/about/guidelines.xhtml

For peer review only

285 **Data sharing statement:** The final dataset that supports the findings of this study will be
286 available from the corresponding author upon reasonable request.

287 **Abbreviations:** AI: Artificial Intelligence; ML: Machine Learning; TRIPOD: Transparent Reporting
288 of multivariable prediction model for Individual Prognosis Or Diagnosis; PROBAST: Prediction
289 model Risk Of Bias Assessment Tool.

REFERENCES

1. Moons KGM, Royston P, Vergouwe Y, *et al*. Prognosis and prognostic research: What, why, and how? *BMJ* 2009; doi:10.1136/bmj.b375.

2. Steyerberg EW, Moons KGM, van der Windt DA, *et al*. Prognosis Research Strategy (PROGRESS) 3: Prognostic Model Research. *PLoS Med* 2013; doi:10.1371/journal.pmed.1001381.

3. Riley RD, van der Windt D, Croft P, Moons KGM. Prognosis Research in Healthcare: Concepts, Methods, and Impact. First ed. Oxford University Press; 2019.

4. Steyerberg EW. Clinical Prediction Models: A Practical Approach to Development, Validation, and Updating. Second ed. Cham, Switzerland: Springer; 2019

5. Damen JAAG, Hooft L, Schuit E, *et al*. Prediction models for cardiovascular disease risk in the general population: Systematic review. *BMJ* 2016; doi:10.1136/bmj.i2416.

6. Heus P, Damen JAAG, Pajouheshnia R, *et al*. Poor reporting of multivariable prediction model studies: Towards a targeted implementation strategy of the TRIPOD statement. *BMC Med* 2018; doi:10.1186/s12916-018-1099-2.

7. Collins GS, de Groot JA, Dutton S, *et al*. External validation of multivariable prediction models: a systematic review of methodological conduct and reporting. *BMC Med Res Methodol* 2014; doi:10.1186/1471-2288-14-40.

8. Bouwmeester W, Zuithoff NPA, Mallett S, *et al*. Reporting and methods in clinical prediction research: A systematic review. *PLoS Med* 2012; doi:10.1371/journal.pmed.1001221

9. Collins GS, Mallet S, Omar O, *et al*. Developing risk prediction models for type 2 diabetes: a systematic review of methodology and reporting. BMC med. 2011; doi:10.1186/1741-9-103.

10. Wen Z, Guo Y, Xu B, *et al*. Developing risk prediction models for postoperative pancreatic fistula: a systematic review of methodology and reporting quality. *Indian J Surg* 2016; doi: 10.1007/s12262-015-1439-9

11. Mitchell TM. Machine Learning. New York, NY: McGraw Hill; 1997.

12. Bi Q, Goodman KE, Kaminsky J, *et al*. What is Machine Learning? A Primer for the Epidemiologist. *Am J Epidemiol* 2019; doi: 10.1093/aje/kwz189.

13. Sidey-Gibbons JAM, Sidey-Gibbons CJ. Machine learning in medicine: a practical introduction. *BMC Med Res Methodol* 2019; doi: 10.1186/s12874-019-0681-4.

Beam AL, Kohane IS. Big Data and Machine Learning in Health Care. *JAMA* 2018; doi: 10.1001/jama.2017.18391.

For peer review only - http://bmjopen.bmj.com/site/about/guidelines.xhtml

14. Harrell FE Jr, Lee KL, Mark DB. Multivariable prognostic models: issues in developing models, evaluating assumptions and adequacy, and measuring and reducing errors. *Stat Med* 1996; doi: 10.1002/(SICI)1097-0258(19960229)15:4<361::AID-SIM168>3.0.CO;2.-4.

15. Collins GS, Reitsma JB, Altman DG, *et al*. Transparent Reporting of a multivariable prediction model for Individual Prognosis Or Diagnosis (TRIPOD): The TRIPOD Statement. *Ann Intern Med* 2015 doi:10.7326/M14-069717.

16. Moons KGM, Altman DG, Reitsma JB, et al. Transparent reporting of a multivariable prediction model for individual prognosis or diagnosis (TRIPOD): Explanation and elaboration. *Ann Intern Med* 2015; doi:10.7326/M14-0698

17. Wolff RF, Moons KGM, Riley RD; PROBAST Group. PROBAST: A tool to assess the risk of bias and applicability of prediction model studies. *Ann Intern Med* 2019; doi:10.7326/M18-1376

18. Moons KGM, Wolff RF, Riley RD, *et al*. PROBAST: A tool to assess risk of bias and applicability of prediction model studies: Explanation and elaboration. *Ann Intern Med* 2019; doi:10.7326/M18-1377

19. Liu Y, Chen PC, Krause J, *et al*. How to read articles that use Machine Learning: Users' guides to the medical literature. *JAMA* 2019; doi: 10.1001/jama.2019.16489.

20. CONSORT-AI and SPIRIT-AI Steering Group. Reporting guidelines for clinical trials evaluating artificial intelligence interventions are needed. *Nat Med* 2019; doi: 10.1038/s41591-019-0603-3.

21. Collins GS, Moons KGM. Reporting of artificial inteligence prediction models. *Lancet* 2019; doi: 10.1016/S0140-6736(19)30037-6.

22. Heus P, Damen JAAG, Pajouheshnia R, *et al*. Uniformity in measuring adherence to reporting guidelines: the example of TRIPOD for assessing completeness of reporting of prediction model studies. *BMJ open* 2019; doi: 10.1136/bmjopen-2018-025611.

23. Moher D, Shamseer L, Clarke M, *et al*. Preferred Reporting Items for Systematic Review and Meta-Analysis Protocols (PRISMA-P) 2015 statement. *Syst Rev* 2015; doi: 10.1186/2046-4053-4-1.

24. Geersing GJ, Bouwmeester W, Zuithoff P, *et al*. Search Filters for Finding Prognostic and Diagnostic Prediction Studies in Medline to Enhance Systematic Reviews. *PLoS one 2012*; doi: 10.1371/journal.pone.0032844.

25. Howard BE, Phillips J, Miller K, et al. SWIFT-Review: a text-mining workbench for systematic review. *Syst Rev* 2016; doi: 10.1186s/s13643-016-0263-z.

26. Fontaine JF, Barbosa-Silva A, Schaefer M, *et al*. MedlineRanker: flexible ranking of biomedical literature. *Nucleic Acids Res* 2009; doi: 10.1093/nar/gkp353.

27. Ouzzani M, Hammady H, Fedorowicz Z, *et al*. Rayyan- a web and mobile app for systematic reviews. *Syst Rev* 2016; doi: 0.1186/s13643-016-0384-4.

28. Harris PA, Taylor R, Thielker R, *et al*. Research electronic data capture (REDCap). A metadata-drien methodology and workflow process for providing translational research informatics support. *J Biomed Inform* 2009; doi: 10.1016/j.jbi.2008.08.010.

For peer review only

For peer review only - http://bmjopen.bmj.com/site/about/guidelines.xhtml

29. Liberati A, Altman DG, Tetzlaff J, *et al*. The PRISMA Statement for Reporting Systematic Reviews and Meta-Analyses of Studies That Evaluate Health Care Interventions: Explanation and Elaboration. *Ann Intern Med* 2009; doi: 10.1371/journal.pmed.1000100.

30. Moons KGM, de Groot JAH, Bouwmeester W, *et al*. Critical Appraisal and Data Extraction for Systematic Reviews of Prediction Modelling Studies: The CHARMS Checklist. *PLoS Med* 2014; doi:10.1371/journal.pmed.1001744.

31. Chen JH, Asch SM. Machine Learning and Prediction in Medicine - Beyond the peak of inflated expectations. *N Engl J Med* 2018; doi: 10.1056/NEJMp1702071.

32. Christodoulou E, Ma J, Collins GS, et al. A systematic review shows no performance benefit of machine learning over logistic regression for clinical prediction models. *J Clin Epidemiol* 2019; doi: 10.1016/j.jclinepi.2019.02.004.

33. Shillan D, Sterne JAC, Champneys A, *et al*. Use of machine learning to analyse routinely collected intensive care unit data: a systematic review. *Crit Care* 2019; doi: 10.1186/s13054-019-2564-9.

34. Wang W, Kiik M, Peek N, *et al*. A systematic review of Machine Learning models for predicting outcomes of stroke with structured data. *Lancet* 2020. Published online first at SSRN: https://ssrn.com/abstract=3520073.

For peer review only

For peer review only - http://bmjopen.bmj.com/site/about/guidelines.xhtml

Appendix 1

PubMed search strategy

Draft for the search strategy for systematic review of the reporting of the development and validation of machine learning models using Pubmed interface.

Search date: 19 November 2019

1. Machine Learning[MeSH Terms]
2. Deep learning[MeSH Terms]
3. supervised machine learning[MeSH Terms]
4. "Neural Networks, Computer"[Mesh]
5. data mining[MeSH Terms]
6. machine[tiab] AND (learn* OR model*)
7. (statistical[tiab] OR "statistical-learning"[tiab]) AND (strateg*[tiab])
8. multilayer perceptron*[tiab] OR random forest*[tiab] OR bayes* network*[tiab] OR support vector machine*[tiab] OR nearest neighbor*[tiab] OR k nearest neighbor*[tiab] OR elastic net[tiab] OR naive bayes*[tiab]
9. (classification[tiab] OR regression[tiab] OR estimation[tiab] OR decision[tiab]) AND tree[tiab]
10. ridge[tiab] OR kernel[tiab] OR ensemble[tiab] OR bagging[tiab] OR bagged[tiab] OR boosting[tiab] OR boosted[tiab] OR fuzzy[tiab]
11. #1 OR #2 OR #3 OR #4 OR #5 OR #6 OR #7 OR #8 OR #9 OR #10
12. (Validat* OR Predict* OR Rule*). [tiab]
13. (Predict* AND (Outcome* OR Risk* OR Model*). [tiab]
14. ((History OR Variable* OR Criteria OR Scor* OR Characteristic* OR Finding* OR Factor*) AND (Predict* OR Model* OR Decision* OR Identif* OR Prognos*)). [tiab]
15. (Decision* AND (Model* OR Clinical*). [tiab]
16. (Prognostic AND (History OR Variable* OR Criteria OR Scor* OR Characteristic* OR Finding* OR Factor* OR Model*). [tiab]
17. #12 OR #13 OR #14 OR #15 OR #16
18. (discrimination[tiab] OR discriminative[tiab] OR discriminatory[tiab]) AND (accuracy[tiab] OR ability[tiab] OR performance[tiab] OR value[tiab] OR model[tiab] OR models[tiab] OR power[tiab] OR capacity[tiab] OR capabilit*[tiab] OR efficiency[tiab])

For peer review only

19. (discriminability[tiab] OR c-index[tiab] OR c-statistic[tiab] OR concordance[tiab] OR DCA[tiab])
20. "decision curve"[tiab]
21. calibrat*[tiab] AND (plot*[tiab] OR curve*[tiab] OR slope*[tiab] OR model[tiab] OR models[tiab])
22. performance[tiab] AND (classification[tiab] OR classifier[tiab] OR clinical[tiab] OR accuracy[tiab] OR validation[tiab] OR metrics[tiab] OR diagnostic[tiab] OR AUC[tiab])
23. (sensitivity[tiab] OR specificity[tiab] OR PPV[tiab] OR NPV[tiab])
24. "correctly classified"[tiab]
25. "clinical accuracy"[tiab]
26. positive predictive value*[tiab]
27. negative predictive value*[tiab]
28. classification[tiab] OR classifier[tiab]
29. Area Under Curve[Mesh]
30. "Area under the curve"[tiab]
31. "Area under the ROC"[tiab]
32. “Area Under the Receiver”[tiab]
33. (ROC[tiab] OR AUC[tiab] OR AUROC[tiab])
34. ROC Curve [Mesh]
35. "Hosmer-Lemeshow"[tiab] OR "H-L test"[tiab]
36. "expected ratio"[tiab] OR "observed ratio"[tiab] OR "E:O ratio"[tiab]
37. #18 OR #19 OR #20 OR #21 OR #22 OR #23 OR #24 OR #25 OR #26 OR #27 OR #28 OR #29 OR #30 OR #31 OR #32 OR #33 OR #34 OR #35 OR #36

---

38. #11 AND #17
39. #11 AND (#17 OR #37)
40. #39 AND (“2018/01/01”[PDat]: “2019/12/31”[PDat])
41. #40 NOT “review”[pt]
42. #39 AND (“2019/01/01”[PDat]: “2019/12/31”[PDat])
43. #42 NOT “review”[pt]

For peer review only

Results #41= **24732**

Results #43=**12977**

For peer review only

Article Information

Article ID

Article available for data extraction

Endnote number

For peer review only

Background and objectives

Article ID	__________________
The background and rationale are presented	◯ Yes<br>◯ No
Reference to existing models is included (or stated that there are no existing models)	◯ Yes<br>◯ No
Any description of why ML techniques are being used to address the objective is reported	◯ Yes<br>◯ No
If yes, please provide the statement below	__________________
It is stated whether the study describes development and/or validation and/or incremental (added) value	◯ Yes<br>◯ No
Any additional comment about the "background and objectives" section of this article?	__________________<br>(If there is something in the "Background" that does not fit into the questions of this form - please use this space to detail. Also use this space to detail anything you are unsure about.)

For peer review only

Reviewer Information

Article ID

Reviewer Information

Reviewer Name

(Provide your initials)

Date of extraction

For peer review only

General Information

Article ID

____

General study information

Title

____

Journal

____

Publication year

____

Number of authors listed

____

Name of the first author

____
(Initials, Surname (e.g. EW Steyerberg))

What is the affiliation of the 1st author?

- ☐ Clinical
- ☐ Epidemiology
- ☐ Health informatics
- ☐ Data sciences
- ☐ Other

If other, please specify

____

What is the clinical area being investigated?

- ◯ Oncology
- ◯ Cardiovascular medicine
- ◯ Critical care
- ◯ Endocrinology
- ◯ Healthcare services
- ◯ Geriatric
- ◯ Hepatology
- ◯ Psychiatry
- ◯ Immunology
- ◯ Neonatology
- ◯ Nutrition
- ◯ Obstetrics & Gynaecology
- ◯ Physical medicine
- ◯ Primary care
- ◯ Surgery
- ◯ Infectious diseases
- ◯ Neurology
- ◯ Ophthalmology
- ◯ Pediatrics
- ◯ Nephrology
- ◯ Medical imaging
- ◯ Other

If other, please specify

____

For peer review only

What is the type of study?	◯ Diagnosis<br>◯ Prognosis
What is the type of study?	◯ Classification<br>◯ Risk prediction<br>◯ Unclear<br>(We need to distinguish if the problem is a prediction problem or a classification problem. A classification problem is about predicting a label and a prediction problems is about predicting a quantity.)
What is the purpose of the article?	◯ Clinical use<br>◯ Simulation/tutoring<br>◯ Contest/Challenge<br>(If tutoring, please finish this form and don't follow with the extraction.)
What is the study design?	◯ Development only (including internal validation)<br>◯ Development with external validation (same model)<br>◯ Development with external validation (different model)<br>◯ External validation only
What is the primary outcome for the model?	__________________________________<br>(Please include timing of primary outcome. Extract on primary outcome only.)
What is the format of the primary outcome?	◯ Continuous<br>◯ Binary<br>◯ Ordinal<br>◯ Multinomial<br>◯ Time to event<br>◯ Count<br>◯ Other
If other, please specify	__________________________________
Any additional comment about the article?	__________________________________<br>(If there is information in "General Information" that does not fit into the questions of this form - please use this space to detail. Also use this space to detail anything you are unsure about.)

For peer review only

Methods

Article ID	__________________________________
Is adherence to a reporting guideline mentioned?	◯ Yes<br>◯ No
If yes, to which guideline?	☐ TRIPOD<br>☐ CONSORT<br>☐ STROBE<br>☐ Other
If other, please specify	__________________________________
What type of study is reported?	◯ Development (including internal validation)<br>◯ Development with external validation (same model)<br>◯ Development with external validation (different model)<br>◯ External validation only<br>(This questions is repeated due to branching logic. Please answer again.)
What is the type of external validation?	☐ Temporal<br>☐ Geographical<br>☐ Independent data<br>☐ Fully independent<br>☐ Unclear<br>☐ Other
If other, please specify	__________________________________<br>(E.g. different setting, different participants population (pediatric/adult))
Differences or similarities in definitions with the development study are described	◯ Yes<br>◯ No<br>◯ NA<br>(Mentioning of any differences in all four (setting, eligibility criteria, predictors and outcome) is required to score Yes. If it is explicitly mentioned that there were no differences in setting, eligibility criteria, predictors and outcomes, score Yes. For incremental value reports, in case additional predictors are not added to a previously developed prediction model but rather added to conventional predictors in a newly fitted model, score Not applicable.)
In which domains are differences?	☐ Setting<br>☐ Eligibility criteria<br>☐ Predictors<br>☐ Outcomes<br>☐ No differences were reported<br>☐ Other

For peer review only

If other, please specify	______________________ (list using (;) to separate if more than 1.)
Is there a diagram/draw to clarify the methods used?	◯ Yes<br>◯ No

Source of data

Development<br>The study design or source of data is reported	◯ Yes<br>◯ No<br>(E.g. Prospectively designed, existing cohort, existing RCT, registry/medical records, case control, case series. This needs to be explicitly reported; reference to this information in another article alone is insufficient.)
External validation<br>The study design or source of data is reported	◯ Yes<br>◯ No<br>(E.g. Prospectively designed, existing cohort, existing RCT, registry/medical records, case control, case series. This needs to be explicitly reported; reference to this information in another article alone is insufficient.)
External validation<br>The study design or source of data is reported	◯ Yes<br>◯ No<br>(E.g. Prospectively designed, existing cohort, existing RCT, registry/medical records, case control, case series. This needs to be explicitly reported; reference to this information in another article alone is insufficient.)
Development<br>If yes, what was the data source origin?	☐ RCT<br>☐ Prospective cohort<br>☐ Retrospective cohort<br>☐ Registry<br>☐ Electronic medical records<br>☐ Case-control/case-cohort study<br>☐ Individual patient data - meta analysis<br>☐ Claims<br>☐ Other<br>(Multiples answers are possible)
External validation<br>If yes, what was the data source origin?	☐ RCT<br>☐ Prospective cohort<br>☐ Retrospective cohort<br>☐ Registry<br>☐ Electronic medical records<br>☐ Case-control/case-cohort study<br>☐ Individual patient data - meta analysis<br>☐ Claims<br>☐ Other<br>(Multiples answers are possible)

For peer review only

External validation<br>If yes, what was the data source origin?	☐ RCT<br>☐ Prospective cohort<br>☐ Retrospective cohort<br>☐ Registry<br>☐ Electronic medical records<br>☐ Case-control/case-cohort study<br>☐ Individual patient data - meta analysis<br>☐ Claims<br>☐ Other<br>(Multiples answers are possible)
If other, please specify	__________________________________
If other, please specify	__________________________________
If other, please specify	__________________________________
Development<br>The starting date of accrual is reported	◯ Yes<br>◯ No
External validation<br>The starting date of accrual is reported	◯ Yes<br>◯ No
External validation<br>The starting date of accrual is reported	◯ Yes<br>◯ No
If yes, what is the start data of data collection?	__________________________________
If yes, what is the start data of data collection?	__________________________________
If yes, what is the start data of data collection?	__________________________________
Development<br>The end date of accrual is reported	◯ Yes<br>◯ No
External validation<br>The end date of accrual is reported	◯ Yes<br>◯ No
External validation<br>The end date of accrual is reported	◯ Yes<br>◯ No
If yes, what is the end date of data collection?	__________________________________
If yes, what is the end date of data collection?	__________________________________
If yes, what is the end date of data collection?	__________________________________

For peer review only

Development<br>The length of follow-up is reported	◯ Yes<br>◯ No<br>◯ NA<br>(E.g. "Patients were followed from baseline for 10 years" and "10-year prediction of..."; notably for prognostic studies with long term follow-up. If this is not applicable for an article (i.e. diagnostic study or no follow-up), then score Not applicable.)
External validation<br>The length of follow-up is reported	◯ Yes<br>◯ No<br>◯ NA<br>(E.g. "Patients were followed from baseline for 10 years" and "10-year prediction of..."; notably for prognostic studies with long term follow-up. If this is not applicable for an article (i.e. diagnostic study or no follow-up), then score Not applicable.)
External validation<br>The length of follow-up is reported	◯ Yes<br>◯ No<br>◯ NA<br>(E.g. "Patients were followed from baseline for 10 years" and "10-year prediction of..."; notably for prognostic studies with long term follow-up. If this is not applicable for an article (i.e. diagnostic study or no follow-up), then score Not applicable.)
If yes, what is the length of follow up?	__________________________________
If yes, what is the length of follow up?	__________________________________
If yes, what is the length of follow up?	__________________________________
Development<br>The length of the prediction horizon/time frame is reported	◯ Yes<br>◯ No<br>◯ NA
External validation<br>The length of the prediction horizon/time frame is reported	◯ Yes<br>◯ No<br>◯ NA
External validation<br>The length of the prediction horizon/time frame is reported	◯ Yes<br>◯ No<br>◯ NA
If yes, what is the length of the prediction horizon/time frame?	__________________________________
If yes, what is the length of the prediction horizon/time frame?	__________________________________
If yes, what is the length of the prediction horizon/time frame?	__________________________________

For peer review only

Participants

Development<br>The study setting is reported	◯ Yes<br>◯ No<br>(E.g.: 'surgery for endometrial cancer patients' is considered to be enough information about the study setting.)
External validation<br>The study setting is reported	◯ Yes<br>◯ No<br>(E.g.: 'surgery for endometrial cancer patients' is considered to be enough information about the study setting.)
External validation<br>The study setting is reported	◯ Yes<br>◯ No<br>(E.g.: 'surgery for endometrial cancer patients' is considered to be enough information about the study setting.)
Development<br>What is the setting for the model?	☐ Primary care<br>☐ Secondary care<br>☐ Tertiary care<br>☐ General population<br>☐ Other<br>(Primary care = GPs, dentists and pharmacists (often first point of care). Secondary care = hospital or clinic based care - can be planned (e.g., cataract operation) or emergency (e.g., fracture). Tertiary care = highly specialised treatments (e.g., transplant, hip replacement).)
External validation<br>What is the setting for the model?	☐ Primary care<br>☐ Secondary care<br>☐ Tertiary care<br>☐ General population<br>☐ Other<br>(Primary care = GPs, dentists and pharmacists (often first point of care). Secondary care = hospital or clinic based care - can be planned (e.g., cataract operation) or emergency (e.g., fracture). Tertiary care = highly specialised treatments (e.g., transplant, hip replacement).)
External validation<br>What is the setting for the model?	☐ Primary care<br>☐ Secondary care<br>☐ Tertiary care<br>☐ General population<br>☐ Other<br>(Primary care = GPs, dentists and pharmacists (often first point of care). Secondary care = hospital or clinic based care - can be planned (e.g., cataract operation) or emergency (e.g., fracture). Tertiary care = highly specialised treatments (e.g., transplant, hip replacement).)
If other, please specify	__________________________________
If other, please specify	__________________________________

For peer review only

If other, please specify	__________________________________
Development<br>The number of centres involved is reported	◯ Yes<br>◯ No<br>(If the number is not reported explicitly, but can be concluded from the name of the centre/centres, or if clearly a single centre study, score Yes.)
External validation<br>The number of centres involved is reported	◯ Yes<br>◯ No<br>(If the number is not reported explicitly, but can be concluded from the name of the centre/centres, or if clearly a single centre study, score Yes.)
External validation<br>The number of centres involved is reported	◯ Yes<br>◯ No<br>(If the number is not reported explicitly, but can be concluded from the name of the centre/centres, or if clearly a single centre study, score Yes.)
How many centres involved?	__________________________________
How many centres involved?	__________________________________
How many centres involved?	__________________________________
Development<br>The geographical location (at least country) of centres involved is reported	◯ Yes<br>◯ No<br>(If no geographical location is specified, but the location can be concluded from the name of the centre(s), score Yes.)
External validation<br>The geographical location (at least country) of centres involved is reported	◯ Yes<br>◯ No<br>(If no geographical location is specified, but the location can be concluded from the name of the centre(s), score Yes.)
External validation<br>The geographical location (at least country) of centres involved is reported	◯ Yes<br>◯ No<br>(If no geographical location is specified, but the location can be concluded from the name of the centre(s), score Yes.)
If yes, what was the geographic location of the data collection?	☐ Europe<br>☐ North America<br>☐ Latin America<br>☐ Asia<br>☐ Africa<br>☐ Oceania<br>(Multiples answers are possible)

For peer review only

If yes, what was the geographic location of the data collection?	☐ Europe<br>☐ North America<br>☐ Latin America<br>☐ Asia<br>☐ Africa<br>☐ Oceania<br>(Multiples answers are possible)
If yes, what was the geographic location of the data collection?	☐ Europe<br>☐ North America<br>☐ Latin America<br>☐ Asia<br>☐ Africa<br>☐ Oceania<br>(Multiples answers are possible)
**Eligibility criteria**	
Development<br>In-/exclusion criteria are stated	◯ Yes<br>◯ No<br>(These should explicitly be stated. Reasons for exclusion only described in a participant flow is not sufficient.)
External validation<br>In-/exclusion criteria are stated	◯ Yes<br>◯ No<br>(These should explicitly be stated. Reasons for exclusion only described in a participant flow is not sufficient.)
External validation<br>In-/exclusion criteria are stated	◯ Yes<br>◯ No<br>(These should explicitly be stated. Reasons for exclusion only described in a participant flow is not sufficient.)
Development<br>What was the participant population?	______________________
External validation<br>What was the participant population?	______________________
External validation<br>What was the participant population?	______________________
Development<br>Details of any treatments received are described	◯ Yes<br>◯ No<br>◯ NA<br>(This item is notably for prognostic modelling studies and is about treatment at baseline or during follow-up. The 'if relevant' judgment of treatment requires clinical knowledge and interpretation. If you are certain that treatment was not relevant, e.g. in some diagnostic model studies, score Not applicable)

For peer review only

External validation<br>Details of any treatments received are described	◯ Yes<br>◯ No<br>◯ NA<br>(This item is notably for prognostic modelling studies and is about treatment at baseline or during follow-up. The 'if relevant' judgment of treatment requires clinical knowledge and interpretation. If you are certain that treatment was not relevant, e.g. in some diagnostic model studies, score Not applicable)
External validation<br>Details of any treatments received are described	◯ Yes<br>◯ No<br>◯ NA<br>(This item is notably for prognostic modelling studies and is about treatment at baseline or during follow-up. The 'if relevant' judgment of treatment requires clinical knowledge and interpretation. If you are certain that treatment was not relevant, e.g. in some diagnostic model studies, score Not applicable)
**Outcome**	
Development<br>The outcome definition is clearly presented	◯ Yes<br>◯ No
External validation<br>The outcome definition is clearly presented	◯ Yes<br>◯ No
External validation<br>The outcome definition is clearly presented	◯ Yes<br>◯ No
Development<br>What is the type of primary outcome?	◯ Death<br>◯ Complications<br>◯ Recurrence<br>◯ Survival<br>◯ Other
External validation<br>What is the type of primary outcome?	◯ Death<br>◯ Complications<br>◯ Recurrence<br>◯ Survival<br>◯ Other
External validation<br>What is the type of primary outcome?	◯ Death<br>◯ Complications<br>◯ Recurrence<br>◯ Survival<br>◯ Other
If other, please specify	__________________________________
If other, please specify	__________________________________
If other, please specify	__________________________________

For peer review only

Development<br>It is described how outcome was assessed	◯ Yes<br>◯ No<br>(Including all elements of any composite, for example CVD [e.g. MI, HF, stroke])
External validation<br>It is described how outcome was assessed	◯ Yes<br>◯ No<br>(Including all elements of any composite, for example CVD [e.g. MI, HF, stroke])
External validation<br>It is described how outcome was assessed	◯ Yes<br>◯ No<br>(Including all elements of any composite, for example CVD [e.g. MI, HF, stroke])
Development<br>It is described when the outcome was assessed (time point(s) since T0)	◯ Yes<br>◯ No
External validation<br>It is described when the outcome was assessed (time point(s) since T0)	◯ Yes<br>◯ No
External validation<br>It is described when the outcome was assessed (time point(s) since T0)	◯ Yes<br>◯ No
Development<br>Actions to blind assessment of outcome to be predicted are reported	◯ Yes<br>◯ No<br>(If it is clearly a non-issue (e.g. all-cause mortality or an outcome not requiring interpretation), score Yes. In all other instances, an explicit mention is expected.)
External validation<br>Actions to blind assessment of outcome to be predicted are reported	◯ Yes<br>◯ No<br>(If it is clearly a non-issue (e.g. all-cause mortality or an outcome not requiring interpretation), score Yes. In all other instances, an explicit mention is expected.)
External validation<br>Actions to blind assessment of outcome to be predicted are reported	◯ Yes<br>◯ No<br>(If it is clearly a non-issue (e.g. all-cause mortality or an outcome not requiring interpretation), score Yes. In all other instances, an explicit mention is expected.)

For peer review only

Predictors

Development<br>All predictors are reported	◯ Yes<br>◯ No<br>(For development, "all predictors" refers to all predictors that potentially could have been included in the 'final' model (including those considered in any univariable analyses).)
External validation<br>All predictors are reported	◯ Yes<br>◯ No<br>(For validation, "all predictors" means the predictors in the model being evaluated.)
External validation<br>All predictors are reported	◯ Yes<br>◯ No<br>(For validation, "all predictors" means the predictors in the model being evaluated.)
Development<br>Number of candidate predictors considered	______________________<br>(If the number of candidate predictors is unclear, please fill this question with "UN")
External validation<br>External validation<br>Number of candidate predictors considered	______________________<br>(If the number of candidate predictors is unclear, please fill this question with 'UN')
Development<br>What are the categories of the candidate predictors?	☐ Demography<br>☐ Clinical history<br>☐ Physical examination<br>☐ Blood and Urine parameters<br>☐ Imaging<br>☐ Genetic Risk Score<br>☐ Pathology<br>☐ Scale Score (e.g. pain, wellbeing, QoL)<br>☐ Questionnaires<br>☐ Other<br>(Multiples answers are possible)
External validation<br>External validation<br>What are the categories of the candidate predictors?	☐ Demography<br>☐ Clinical history<br>☐ Physical examination<br>☐ Blood and Urine parameters<br>☐ Imaging<br>☐ Genetic Risk Score<br>☐ Pathology<br>☐ Scale Score (e.g. pain, wellbeing, QoL)<br>☐ Questionnaires<br>☐ Other<br>(Multiples answers are possible)
If other, please specify	______________________<br>(list using (;) to separate if more than 1)
If other, please specify	______________________<br>(list using (;) to separate if more than 1)

For peer review only

Development<br>Were a-priori predictors considered/forced into the model?	◯ Yes<br>◯ No<br>◯ Unclear<br>(E.g. Clinical reasoning, literature review, money constraints)
Development<br>Predictor definitions are clearly presented	◯ Yes<br>◯ No
External validation<br>Predictor definitions are clearly presented	◯ Yes<br>◯ No
External validation<br>Predictor definitions are clearly presented	◯ Yes<br>◯ No
Development<br>It is clearly described how the predictors were measured	◯ Yes<br>◯ No
External validation<br>It is clearly described how the predictors were measured	◯ Yes<br>◯ No
External validation<br>It is clearly described how the predictors were measured	◯ Yes<br>◯ No
Development<br>It is clearly described when the predictors were measured	◯ Yes<br>◯ No
External validation<br>It is clearly described when the predictors were measured	◯ Yes<br>◯ No
External validation<br>It is clearly described when the predictors were measured	◯ Yes<br>◯ No
Development<br>It is clearly described whether predictor assessments were blinded for outcome	◯ Yes<br>◯ No<br>(For predictors for which it is clearly a non-issue (e.g. automatic blood pressure measurement, age, sex) and for instances where the predictors were clearly assessed before outcome assessment, score Yes. For all other predictors an explicit mention is expected.)
External validation<br>It is clearly described whether predictor assessments were blinded for outcome	◯ Yes<br>◯ No<br>(For predictors for which it is clearly a non-issue (e.g. automatic blood pressure measurement, age, sex) and for instances where the predictors were clearly assessed before outcome assessment, score Yes. For all other predictors an explicit mention is expected.)

For peer review only

External validation<br>It is clearly described whether predictor assessments were blinded for outcome	◯ Yes<br>◯ No<br>(For predictors for which it is clearly a non-issue (e.g. automatic blood pressure measurement, age, sex) and for instances where the predictors were clearly assessed before outcome assessment, score Yes. For all other predictors an explicit mention is expected.)
Development<br>It is clearly described whether predictor assessments were blinded for the other predictors	◯ Yes<br>◯ No
External validation<br>It is clearly described whether predictor assessments were blinded for the other predictors	◯ Yes<br>◯ No
External validation<br>It is clearly described whether predictor assessments were blinded for the other predictors	◯ Yes<br>◯ No
**Sample Size**	
Development<br>It is explained how the sample size was arrived at	◯ Yes<br>◯ No<br>(Is there any mention of sample size, e.g. whether this was done on statistical grounds or practical/logistical grounds (e.g. an existing study cohort or data set of a RCT was used)?)
External validation<br>It is explained how the sample size was arrived at	◯ Yes<br>◯ No<br>(Is there any mention of sample size, e.g. whether this was done on statistical grounds or practical/logistical grounds (e.g. an existing study cohort or data set of a RCT was used)?)
External validation<br>It is explained how the sample size was arrived at	◯ Yes<br>◯ No<br>(Is there any mention of sample size, e.g. whether this was done on statistical grounds or practical/logistical grounds (e.g. an existing study cohort or data set of a RCT was used)?)
Development<br>What is the reason for the sample?	◯ Power<br>◯ Justified time interval<br>◯ Size of existing/available data<br>◯ Events per variable<br>◯ Other
External validation<br>What is the reason for the sample?	◯ Power<br>◯ Justified time interval<br>◯ Size of existing/available data<br>◯ Events per variable<br>◯ Other

For peer review only

External validation<br>What is the reason for the sample?	◯ Power<br>◯ Justified time interval<br>◯ Size of existing/available data<br>◯ Events per variable<br>◯ Other
Development<br>If other, please specify	__________________
External validation<br>If other, please specify	__________________
External validation<br>If other, please specify	__________________
Development<br>What was the initial sample size of the study?	__________________<br>(If unclear, please fill this with "UN")
Development<br>What was the final sample size of the study?	__________________<br>(If unclear, please fill this with "UN")
External validation<br>What was the initial sample size of the study?	__________________<br>(If unclear, please fill this with "UN")
External validation<br>What was the final sample size of the study?	__________________<br>(If unclear, please fill this with "UN")
External validation<br>What was the initial sample size of the study?	__________________<br>(If unclear, please fill this with "UN")
External validation<br>What was the final sample size of the study?	__________________<br>(If unclear, please fill this with "UN")

Missing Data

Development<br>Was missingness an explicit exclusion criterion for the data?	◯ Yes<br>◯ No<br>◯ Unclear
External validation<br>Was missingness an explicit exclusion criterion for the data?	◯ Yes<br>◯ No<br>◯ Unclear
External validation<br>Was missingness an explicit exclusion criterion for the data?	◯ Yes<br>◯ No<br>◯ Unclear
Development<br>If yes, how many patients were excluded due to missing data?	__________________<br>(If not reported, please fill this with "NR")

For peer review only

External validation<br>If yes, how many patients were excluded due to missing data?	________________________________<br>(If not reported, please fill this with "NR")
External validation<br>If yes, how many patients were excluded due to missing data?	________________________________<br>(If not reported, fill this with "NR")
Development<br>The method for handling missing data (predictors and outcome) is mentioned	◯ Yes<br>◯ No<br>(E.g. Complete case (explicit mention that individuals with missing values have been excluded), single imputation, multiple imputation, mean/median imputation. If there is no missing data, there should be an explicit mention that there is no missing data for all predictors and outcome. If so, score Yes. If it is unclear whether there is missing data (from e.g. the reported methods or results), score No. If it is clear there is missing data, but the method for handling missing data is unclear, score No.)
External validation<br>The method for handling missing data (predictors and outcome) is mentioned	◯ Yes<br>◯ No<br>(E.g. Complete case (explicit mention that individuals with missing values have been excluded), single imputation, multiple imputation, mean/median imputation. If there is no missing data, there should be an explicit mention that there is no missing data for all predictors and outcome. If so, score Yes. If it is unclear whether there is missing data (from e.g. the reported methods or results), score No. If it is clear there is missing data, but the method for handling missing data is unclear, score No.)
External validation<br>The method for handling missing data (predictors and outcome) is mentioned	◯ Yes<br>◯ No<br>(E.g. Complete case (explicit mention that individuals with missing values have been excluded), single imputation, multiple imputation, mean/median imputation. If there is no missing data, there should be an explicit mention that there is no missing data for all predictors and outcome. If so, score Yes. If it is unclear whether there is missing data (from e.g. the reported methods or results), score No. If it is clear there is missing data, but the method for handling missing data is unclear, score No.)

For peer review only

Development<br>In presence of missing data, how was this dealt with?	☐ No missing data<br>☐ No imputation<br>☐ Multiple Imputation<br>☐ Complete case analysis<br>☐ Mean imputation<br>☐ Median imputation<br>☐ Other<br>(If there is any discrepancy between how missing values were handled for outcome and predictors, please specify so it in the comments below the methods form.)
External validation<br>In presence of missing data, how was this dealt with?	☐ No missing data<br>☐ No imputation<br>☐ Multiple Imputation<br>☐ Complete case analysis<br>☐ Mean imputation<br>☐ Median imputation<br>☐ Other<br>(If there is any discrepancy between how missing values were handled for outcome and predictors, please specify so it in the comments below the methods form.)
External validation<br>In presence of missing data, how was this dealt with?	☐ No missing data<br>☐ No imputation<br>☐ Multiple Imputation<br>☐ Complete case analysis<br>☐ Mean imputation<br>☐ Median imputation<br>☐ Other<br>(If there is any discrepancy between how missing values were handled for outcome and predictors, please specify so it in the comments below the methods form. Report here imputation for predictors .)
If other, please specify	__________________________________
If other, please specify	__________________________________
If other, please specify	__________________________________
Development<br>If missing data were imputed, details of the software used are given	◯ Yes<br>◯ No<br>◯ NA
External validation<br>If missing data were imputed, details of the software used are given	◯ Yes<br>◯ No<br>◯ NA
External validation<br>If missing data were imputed, details of the software used are given	◯ Yes<br>◯ No<br>◯ NA

For peer review only

Development
If missing data were imputed, a description of which variables were included in the imputation procedure is given.

◯ Yes
◯ No
◯ NA

External validation
If missing data were imputed, a description of which variables were included in the imputation procedure is given.

◯ Yes
◯ No
◯ NA

External validation
If missing data were imputed, a description of which variables were included in the imputation procedure is given.

◯ Yes
◯ No
◯ NA

Development
If multiple imputation was used, the number of imputations is reported

◯ Yes
◯ No

External validation
If multiple imputation was used, the number of imputations is reported

◯ Yes
◯ No

External validation
If multiple imputation was used, the number of imputations is reported

◯ Yes
◯ No

Development
How is missing data presented in the paper or supplemental material?

◯ Overall
◯ By all candidate variables
◯ By all final model variables
◯ By number of variables
◯ Not summarised

External validation
How is missing data presented in the paper or supplemental material?

◯ Overall
◯ By all candidate variables
◯ By all final model variables
◯ By number of variables
◯ Not summarised

External validation
How is missing data presented in the paper or supplemental material?

◯ Overall
◯ By all candidate variables
◯ By all final model variables
◯ By number of variables
◯ Not summarised

Development
If missing data is presented/summarised, what is the percentage/number of individuals have missing data (overall)

____
(If this is unclear, please fill this with "UN")

External validation
If missing data is presented/summarised, what is the percentage/number of individuals have missing data (overall)

____
(If this is unclear, please fill this with "UN")

For peer review only

External validation<br>If missing data is presented/summarised, what is the percentage/number of individuals have missing data (overall)	______________________________<br>(If this is unclear, please fill this with "UN")

Statistical Analysis

Data pre-processing

Did the candidate predictors include continuous variables?	◯ Yes<br>◯ No<br>◯ Unclear
For continuous predictors it is described whether they were modelled as linear, nonlinear (type of transformation specified) or categorized.	◯ Yes<br>◯ No<br>◯ Unclear<br>(A general statement is sufficient, no need to describe this for each predictors.)
How were continuous predictors handled?	☐ Linear (no change)<br>☐ Non-linear (explicity/planned)<br>☐ Non-linear (implicitly/unplanned)<br>☐ Categorised (some)<br>☐ Categorised (all)<br>☐ Other<br>(Non linear terms may not be explicitly reported/planned but handled within the model building process for ML and maybe implicit/unplanned. If an ML model is being evaluated and non-linear terms are not explicitly reported - choose 'Yes (implicit/unplanned)'. A general statement is sufficient, no need to describe this for each predictor separately.)
If other, please specify	______________________________
For categorical or categorized predictors, the cut-points were reported	◯ Yes<br>◯ No
For categorized predictors the method to choose the cut-points was clearly described	◯ Yes<br>◯ No<br>◯ NA<br>(If no categorized predictors, score Not applicable.)
If categorised/dichotomised, how was this done?	◯ Quantiles<br>◯ Data dependent<br>◯ Mixture<br>◯ No rationale<br>◯ Based on previous literature or/and standarization

For peer review only

Is there any other data pre-processing methods used?	☐ Cleaning<br>☐ Aggregation<br>☐ Transformation<br>☐ Sampling<br>☐ Standardization<br>☐ Integration<br>☐ Reduction<br>☐ Other<br>☐ No<br>(Multiples answers are possible. E.g. data aggregation (calculating predictors from other collected data), other predictor transformations, data sampling (only using part of a dataset), predictor standardisation.)
If other, please specify	__________________________________<br>(list using (;) to separate if more then 1)
Is class imbalance addressed?	◯ Yes<br>◯ No<br>◯ NA<br>(There is a disproportionate ratio of observations in each class/group -most machine learning algorithms work best when the number of samples in each class are about equal.)
If yes, how?	__________________________________<br>(list using (;) to separate if more than 1)
Is class imbalance handling justified?	◯ Yes<br>◯ No
Is there any data reduction techniques used?	◯ Yes<br>◯ No<br>(E.g. Missing values ratio, Low variance filter, High correlation filter, Random Forest / Ensembles tress, Principal Component Analysis (PCA), Backward feature elimination, Forward feature construction, autoencoder, Non-negative matrix factorization, Kernel PCA, Graph-based kernel PCA, etc.)
If yes, what techniques were used?	__________________________________<br>(list using (;) to separate if more then 1)
Is data reduction justified?	◯ Yes<br>◯ No

For peer review only

Was collinearity assessed?	◯ Yes, implicitly<br>◯ Yes, explicitly<br>◯ No<br>◯ NA<br>(Collinearity may not be explicitly reported/planned but handled within the model building process for ML and maybe implicit/unplanned. If an ML model is being evaluated and collinearity are not explicitly reported - choose 'Yes (implicit/unplanned)'. A general statement is sufficient, no need to describe this for each predictor separately.)

Model building

Instructions

-Please extract the models in the order they are presented in the article.

-If more than 10 models were developed for the main outcome, only refer to the first 10.

-If a comparison with logistic regression was made, please included this model in the final count and extract information.

How many models were developed for the primary outcome?	__________________________________<br>(This should reflect the number of models you are going to extract on - primary outcome and primary timepoint (If more than 10 models were developed, only refer to the first 10). If a comparison with logistic regression was made, please included this model in the final count.)
External validation<br>It is described how predictions for individuals (in the validation set) were obtained from the model being validated	◯ Yes<br>◯ No<br>(E.g. Using the original reported model coefficients with or without the intercept, and/or using updated or refitted model coefficients, or using a nomogram, spreadsheet or web calculator.)

Model 1

The type of statistical modelling approach is reported	◯ Yes<br>◯ No<br>(E.g. Neural Network)
What is the ML technique being used?	◯ Neural network<br>◯ Random forest<br>◯ Classification and regression tree (CART)<br>◯ Support vector machine<br>◯ Gradient boosting machine<br>◯ Logistic regression<br>◯ Other
If other, please specify	__________________________________

For peer review only - http://bmjopen.bmj.com/site/about/guidelines.xhtml

Do they clearly state why the technique is selected?	__________________________________<br>(E.g. R code availability, clinical question, previous literature, comparison. If not reported, fill this with "NR")
The approach used for predictor selection before modelling is described	◯ Yes<br>◯ No<br>◯ NA<br>(Before modelling' means before any univariable or multivariable analysis of predictor-outcome associations. If no predictor selection before modelling is done, score Not applicable. If it is unclear whether predictor selection before modelling is done, score No. If it is clear there was predictor selection before modelling but the method was not described, score No.)
The approach used for predictor selection during modelling is described	◯ Yes<br>◯ No<br>◯ NA<br>(E.g. Univariable analysis, stepwise selection, bootstrap, Lasso. 'During modelling' includes both univariable or multivariable analysis of predictor-outcome associations. If no predictor selection during modelling is done (so-called full model approach), score Not applicable. If it is unclear whether predictor selection during modelling is done, score No. If it is clear there was predictor selection during modelling but the method was not described, score No.)
What was the model building strategy?	◯ Stepwise<br>◯ Forward selection<br>◯ Backward selection<br>◯ All predictors<br>◯ All significant in univariable<br>◯ Data-driven<br>◯ Other<br>◯ Unclear
If other, please specify	__________________________________<br>(list using (;) to separate if more than 1)
Are hyper-parameters tuning reported?	◯ Yes<br>◯ No<br>◯ NA<br>(Answer yes if any information is giving about how the models were set-up. Term as number of layers, node, optimization, hyperparameters, etc.)
Is predictor importance assessed?	◯ Yes<br>◯ No<br>(E.g. Importance, Mean decrease/Increase in accuracy, Mean decrease Gini, Gini Index, Average impurity decrease, etc.)

For peer review only

If yes, how?	______________________________ (list using (;) to separate if more then 1)
Is there any ML technique being used for predictors/feature selection?	◯ Yes<br>◯ No<br>(E.g. SVM)
If yes, which technique?	______________________________ (list using (;) to separate if more then 1)
Testing of interaction terms is described	◯ Yes, explicitly.<br>◯ Yes, implicitly.<br>◯ No<br>(Interaction terms may not be explicitly reported/planned but handled within the model building process for ML and maybe implicit/unplanned. If an ML model is being evaluated and interactions are not explicitly reported - choose 'Yes (implicit/unplanned)'. A general statement is sufficient, no need to describe this for each predictor separately.)
If applicable, how was censoring accounted for?	______________________________ (If not applicable, fill this with "NA")
Testing of the proportionality of hazards in survival models is described	◯ Yes<br>◯ No<br>◯ NA<br>(If no proportional hazard model is used, score Not applicable.)
What shrinkage/penalisation methods were used?	◯ None<br>◯ Uniform shrinkage<br>◯ Penalised estimation<br>◯ Other
If other, please specify	______________________________

Model 2

The type of statistical modelling approach is reported	◯ Yes<br>◯ No<br>(E.g. Neural Network)
What is the ML technique being used?	◯ Neural network<br>◯ Random forest<br>◯ Classification and regression tree (CART)<br>◯ Support vector machine<br>◯ Gradient boosting machine<br>◯ Logistic regression<br>◯ Other
If other, please specify	______________________________

For peer review only

Do they clearly state why the technique is selected?	__________________________________ (E.g. R code availability, clinical question, previous literature, comparison. If not reported, fill this with "NR")
The approach used for predictor selection before modelling is described	◯ Yes<br>◯ No<br>◯ NA<br>(Before modelling' means before any univariable or multivariable analysis of predictor-outcome associations. If no predictor selection before modelling is done, score Not applicable. If it is unclear whether predictor selection before modelling is done, score No. If it is clear there was predictor selection before modelling but the method was not described, score No.)
The approach used for predictor selection during modelling is described	◯ Yes<br>◯ No<br>◯ NA<br>(E.g. Univariable analysis, stepwise selection, bootstrap, Lasso. 'During modelling' includes both univariable or multivariable analysis of predictor-outcome associations. If no predictor selection during modelling is done (so-called full model approach), score Not applicable. If it is unclear whether predictor selection during modelling is done, score No. If it is clear there was predictor selection during modelling but the method was not described, score No.)
What was the model building strategy?	◯ Stepwise<br>◯ Forward selection<br>◯ Backward selection<br>◯ All predictors<br>◯ All significant in univariable<br>◯ Data-driven<br>◯ Other<br>◯ Unclear
If other, please specify	__________________________________ (list using (;) to separate if more than 1)
Are hyper-parameters tuning reported?	◯ Yes<br>◯ No<br>◯ NA<br>(Answer yes if any information is giving about how the models were set-up. Term as number of layers, node, optimization, hyperparameters, etc.)
Is predictor importance assessed?	◯ Yes<br>◯ No<br>(E.g. Importance, Mean decrease/Increase in accuracy, Mean decrease Gini, Gini Index, Average impurity decrease, etc.)

For peer review only

If yes, how?	______________________<br>(list using (;) to separate if more than 1)
Is there any ML technique being used for predictors/feature selection?	◯ Yes<br>◯ No<br>(E.g. SVM)
If yes, which technique?	______________________<br>(list using (;) to separate if more than 1)
Testing of interaction terms is described	◯ Yes, explicitly.<br>◯ Yes, implicitly.<br>◯ No<br>(Interaction terms may not be explicitly reported/planned but handled within the model building process for ML and maybe implicit/unplanned. If an ML model is being evaluated and interactions are not explicitly reported - choose 'Yes (implicit/unplanned)'. A general statement is sufficient, no need to describe this for each predictor separately.)
If applicable, how was censoring accounted for?	______________________<br>(If not applicable, fill this with "NA")
Testing of the proportionality of hazards in survival models is described	◯ Yes<br>◯ No<br>◯ NA
What shrinkage/penalisation methods were used?	◯ None<br>◯ Uniform shrinkage<br>◯ Penalised estimation<br>◯ Other
If other, please specify	______________________

Model 3

The type of statistical modelling approach is reported	◯ Yes<br>◯ No<br>(E.g. Neural Network)
What is the ML technique being used?	◯ Neural network<br>◯ Random forest<br>◯ Classification and regression tree (CART)<br>◯ Support vector machine<br>◯ Gradient boosting machine<br>◯ Logistic regression<br>◯ Other
If other, please specify	______________________

For peer review only

Do they clearly state why the technique is selected?	__________________ (E.g. R code availability, clinical question, previous literature, comparison. If not reported, fill this with "NR")
The approach used for predictor selection before modelling is described	◯ Yes<br>◯ No<br>◯ NA<br>(Before modelling' means before any univariable or multivariable analysis of predictor-outcome associations. If no predictor selection before modelling is done, score Not applicable. If it is unclear whether predictor selection before modelling is done, score No. If it is clear there was predictor selection before modelling but the method was not described, score No.)
The approach used for predictor selection during modelling is described	◯ Yes<br>◯ No<br>◯ NA<br>(E.g. Univariable analysis, stepwise selection, bootstrap, Lasso. 'During modelling' includes both univariable or multivariable analysis of predictor-outcome associations. If no predictor selection during modelling is done (so-called full model approach), score Not applicable. If it is unclear whether predictor selection during modelling is done, score No. If it is clear there was predictor selection during modelling but the method was not described, score No.)
What was the model building strategy?	◯ Stepwise<br>◯ Forward selection<br>◯ Backward selection<br>◯ All predictors<br>◯ All significant in univariable<br>◯ Data-driven<br>◯ Other<br>◯ Unclear
If other, please specify	__________________ (list using (;) to separate if more than 1)
Are hyper-parameters tuning reported?	◯ Yes<br>◯ No<br>◯ NA<br>(Answer yes if any information is giving about how the models were set-up. Term as number of layers, node, optimization, hyperparameters, etc.)
Is predictor importance assessed?	◯ Yes<br>◯ No<br>(E.g. Importance, Mean decrease/Increase in accuracy, Mean decrease Gini, Gini Index, Average impurity decrease, etc.)

For peer review only

If yes, how?	______________________ (list using (;) to separate if more than 1)
Is there any ML technique being used for predictors/feature selection?	◯ Yes<br>◯ No<br>(E.g. SVM)
If yes, which technique?	______________________ (list using (;) to separate if more than 1)
Testing of interaction terms is described	◯ Yes, explicitly.<br>◯ Yes, implicitly.<br>◯ No<br>(Interaction terms may not be explicitly reported/planned but handled within the model building process for ML and maybe implicit/unplanned. If an ML model is being evaluated and interactions are not explicitly reported - choose 'Yes (implicit/unplanned)'. A general statement is sufficient, no need to describe this for each predictor separately.)
If applicable, how was censoring accounted for?	______________________ (If not applicable, fill this with "NA")
Testing of the proportionality of hazards in survival models is described	◯ Yes<br>◯ No<br>◯ NA
What shrinkage/penalisation methods were used?	◯ None<br>◯ Uniform shrinkage<br>◯ Penalised estimation<br>◯ Other
If other, please specify	______________________

Model 4

The type of statistical modelling approach is reported	◯ Yes<br>◯ No<br>(E.g. Neural Network)
What is the ML technique being used?	◯ Neural network<br>◯ Random forest<br>◯ Classification and regression tree (CART)<br>◯ Support vector machine<br>◯ Gradient boosting machine<br>◯ Logistic regression<br>◯ Other
If other, please specify	______________________

For peer review only

Do they clearly state why the technique is selected?	__________________________________ (E.g. R code availability, clinical question, previous literature, comparison. If not reported, fill this with "NR")
The approach used for predictor selection before modelling is described	◯ Yes<br>◯ No<br>◯ NA<br>(Before modelling' means before any univariable or multivariable analysis of predictor-outcome associations. If no predictor selection before modelling is done, score Not applicable. If it is unclear whether predictor selection before modelling is done, score No. If it is clear there was predictor selection before modelling but the method was not described, score No.)
The approach used for predictor selection during modelling is described	◯ Yes<br>◯ No<br>◯ NA<br>(E.g. Univariable analysis, stepwise selection, bootstrap, Lasso. 'During modelling' includes both univariable or multivariable analysis of predictor-outcome associations. If no predictor selection during modelling is done (so-called full model approach), score Not applicable. If it is unclear whether predictor selection during modelling is done, score No. If it is clear there was predictor selection during modelling but the method was not described, score No.)
What was the model building strategy?	◯ Stepwise<br>◯ Forward selection<br>◯ Backward selection<br>◯ All predictors<br>◯ All significant in univariable<br>◯ Data-driven<br>◯ Other<br>◯ Unclear
If other, please specify	__________________________________ (list using (;) to separate if more than 1)
Are hyper-parameters tuning reported?	◯ Yes<br>◯ No<br>◯ NA<br>(Answer yes if any information is giving about how the models were set-up. Term as number of layers, node, optimization, hyperparameters, etc.)
Is predictor importance assessed?	◯ Yes<br>◯ No<br>(E.g. Importance, Mean decrease/Increase in accuracy, Mean decrease Gini, Gini Index, Average impurity decrease, etc.)

For peer review only

If yes, how?	______________________ (list using (;) to separate if more than 1)
Is there any ML technique being used for predictors/feature selection?	◯ Yes<br>◯ No<br>(E.g. SVM)
If yes, which technique?	______________________ (list using (;) to separate if more than 1)
Testing of interaction terms is described	◯ Yes, explicitly.<br>◯ Yes, implicitly.<br>◯ No<br>(Interaction terms may not be explicitly reported/planned but handled within the model building process for ML and maybe implicit/unplanned. If an ML model is being evaluated and interactions are not explicitly reported - choose 'Yes (implicit/unplanned)'. A general statement is sufficient, no need to describe this for each predictor separately.)
If applicable, how was censoring accounted for?	______________________ (If not applicable, fill this with "NA")
Testing of the proportionality of hazards in survival models is described	◯ Yes<br>◯ No<br>◯ NA
What shrinkage/penalisation methods were used?	◯ None<br>◯ Uniform shrinkage<br>◯ Penalised estimation<br>◯ Other
If other, please specify	______________________

Model 5

The type of statistical modelling approach is reported	◯ Yes<br>◯ No<br>(E.g. Neural Network)
What is the ML technique being used?	◯ Neural network<br>◯ Random forest<br>◯ Classification and regression tree (CART)<br>◯ Support vector machine<br>◯ Gradient boosting machine<br>◯ Logistic regression<br>◯ Other
If other, please specify	______________________

For peer review only

Do they clearly state why the technique is selected?	__________________________________ (E.g. R code availability, clinical question, previous literature, comparison. If not reported, fill this with "NR")
The approach used for predictor selection before modelling is described	◯ Yes<br>◯ No<br>◯ NA<br>(Before modelling' means before any univariable or multivariable analysis of predictor-outcome associations. If no predictor selection before modelling is done, score Not applicable. If it is unclear whether predictor selection before modelling is done, score No. If it is clear there was predictor selection before modelling but the method was not described, score No.)
The approach used for predictor selection during modelling is described	◯ Yes<br>◯ No<br>◯ NA<br>(E.g. Univariable analysis, stepwise selection, bootstrap, Lasso. 'During modelling' includes both univariable or multivariable analysis of predictor-outcome associations. If no predictor selection during modelling is done (so-called full model approach), score Not applicable. If it is unclear whether predictor selection during modelling is done, score No. If it is clear there was predictor selection during modelling but the method was not described, score No.)
What was the model building strategy?	◯ Stepwise<br>◯ Forward selection<br>◯ Backward selection<br>◯ All predictors<br>◯ All significant in univariable<br>◯ Data-driven<br>◯ Other<br>◯ Unclear
If other, please specify	__________________________________ (list using (;) to separate if more than 1)
Are hyper-parameters tuning reported?	◯ Yes<br>◯ No<br>◯ NA<br>(Answer yes if any information is giving about how the models were set-up. Term as number of layers, node, optimization, hyperparameters, etc.)
Is predictor importance assessed?	◯ Yes<br>◯ No<br>(E.g. Importance, Mean decrease/Increase in accuracy, Mean decrease Gini, Gini Index, Average impurity decrease, etc.)

For peer review only

If yes, how?	____________________<br>(list using (;) to separate if more than 1)
Is there any ML technique being used for predictors/feature selection?	◯ Yes<br>◯ No<br>(E.g. SVM)
If yes, which technique?	____________________<br>(list using (;) to separate if more than 1)
Testing of interaction terms is described	◯ Yes, explicitly.<br>◯ Yes, implicitly.<br>◯ No<br>(Interaction terms may not be explicitly reported/planned but handled within the model building process for ML and maybe implicit/unplanned. If an ML model is being evaluated and interactions are not explicitly reported - choose 'Yes (implicit/unplanned)'. A general statement is sufficient, no need to describe this for each predictor separately.)
If applicable, how was censoring accounted for?	____________________<br>(If not applicable, fill this with "NA")
Testing of the proportionality of hazards in survival models is described	◯ Yes<br>◯ No<br>◯ NA
What shrinkage/penalisation methods were used?	◯ None<br>◯ Uniform shrinkage<br>◯ Penalised estimation<br>◯ Other
If other, please specify	____________________

For peer review only

Model 6

The type of statistical modelling approach is reported	◯ Yes<br>◯ No<br>(E.g. Neural Network)
What is the ML technique being used?	◯ Neural network<br>◯ Random forest<br>◯ Classification and regression tree (CART)<br>◯ Support vector machine<br>◯ Gradient boosting machine<br>◯ Logistic regression<br>◯ Other
If other, please specify	____________________

Do they clearly state why the technique is selected?	______________________ (E.g. R code availability, clinical question, previous literature, comparison. If not reported, fill this with "NR")
The approach used for predictor selection before modelling is described	◯ Yes<br>◯ No<br>◯ NA<br>(Before modelling' means before any univariable or multivariable analysis of predictor-outcome associations. If no predictor selection before modelling is done, score Not applicable. If it is unclear whether predictor selection before modelling is done, score No. If it is clear there was predictor selection before modelling but the method was not described, score No.)
The approach used for predictor selection during modelling is described	◯ Yes<br>◯ No<br>◯ NA<br>(E.g. Univariable analysis, stepwise selection, bootstrap, Lasso. 'During modelling' includes both univariable or multivariable analysis of predictor-outcome associations. If no predictor selection during modelling is done (so-called full model approach), score Not applicable. If it is unclear whether predictor selection during modelling is done, score No. If it is clear there was predictor selection during modelling but the method was not described, score No.)
What was the model building strategy?	◯ Stepwise<br>◯ Forward selection<br>◯ Backward selection<br>◯ All predictors<br>◯ All significant in univariable<br>◯ Data-driven<br>◯ Other<br>◯ Unclear
If other, please specify	______________________ (list using (;) to separate if more than 1)
Are hyper-parameters tuning reported?	◯ Yes<br>◯ No<br>◯ NA<br>(Answer yes if any information is giving about how the models were set-up. Term as number of layers, node, optimization, hyperparameters, etc.)
Is predictor importance assessed?	◯ Yes<br>◯ No<br>(E.g. Importance, Mean decrease/Increase in accuracy, Mean decrease Gini, Gini Index, Average impurity decrease, etc.)

For peer review only

If yes, how?	(list using (;) to separate if more than 1)
Is there any ML technique being used for predictors/feature selection?	◯ Yes<br>◯ No<br>(E.g. SVM)
If yes, which technique?	(list using (;) to separate if more than 1)
Testing of interaction terms is described	◯ Yes, implicitly.<br>◯ Yes, explicitly.<br>◯ No<br>(Interaction terms may not be explicitly reported/planned but handled within the model building process for ML and maybe implicit/unplanned. If an ML model is being evaluated and interactions are not explicitly reported - choose 'Yes (implicit/unplanned)'. A general statement is sufficient, no need to describe this for each predictor separately.)
If applicable, how was censoring accounted for?	(If not applicable, fill this with "NA")
Testing of the proportionality of hazards in survival models is described	◯ Yes<br>◯ No<br>◯ NA
What shrinkage/penalisation methods were used?	◯ None<br>◯ Uniform shrinkage<br>◯ Penalised estimation<br>◯ Other
If other, please specify	

Model 7

The type of statistical modelling approach is reported	◯ Yes<br>◯ No<br>(E.g. Neural Network)
What is the ML technique being used?	◯ Neural network<br>◯ Random forest<br>◯ Classification and regression tree (CART)<br>◯ Support vector machine<br>◯ Gradient boosting machine<br>◯ Logistic regression<br>◯ Other
If other, please specify	

For peer review only

Do they clearly state why the technique is selected?

____
(E.g. R code availability, clinical question, previous literature, comparison. If not reported, fill this with "NR")

The approach used for predictor selection before modelling is described

◯ Yes
◯ No
◯ NA
(Before modelling' means before any univariable or multivariable analysis of predictor-outcome associations. If no predictor selection before modelling is done, score Not applicable. If it is unclear whether predictor selection before modelling is done, score No. If it is clear there was predictor selection before modelling but the method was not described, score No.)

The approach used for predictor selection during modelling is described

◯ Yes
◯ No
◯ NA
(E.g. Univariable analysis, stepwise selection, bootstrap, Lasso. 'During modelling' includes both univariable or multivariable analysis of predictor-outcome associations. If no predictor selection during modelling is done (so-called full model approach), score Not applicable. If it is unclear whether predictor selection during modelling is done, score No. If it is clear there was predictor selection during modelling but the method was not described, score No.)

What was the model building strategy?

◯ Stepwise
◯ Forward selection
◯ Backward selection
◯ All predictors
◯ All significant in univariable
◯ Data-driven
◯ Other
◯ Unclear

If other, please specify

____
(list using (;) to separate if more than 1)

Are hyper-parameters tuning reported?

◯ Yes
◯ No
◯ NA
(Answer yes if any information is giving about how the models were set-up. Term as number of layers, node, optimization, hyperparameters, etc.)

Is predictor importance assessed?

◯ Yes
◯ No
(E.g. Importance, Mean decrease/Increase in accuracy, Mean decrease Gini, Gini Index, Average impurity decrease, etc.)

For peer review only

If yes, how?	______________________ (list using (;) to separate if more than 1)
Is there any ML technique being used for predictors/feature selection?	◯ Yes<br>◯ No<br>(E.g. SVM)
If yes, which technique?	______________________ (list using (;) to separate if more than 1)
Testing of interaction terms is described	◯ Yes, implicitly.<br>◯ Yes, explicitly.<br>◯ No<br>(Interaction terms may not be explicitly reported/planned but handled within the model building process for ML and maybe implicit/unplanned. If an ML model is being evaluated and interactions are not explicitly reported - choose 'Yes (implicit/unplanned)'. A general statement is sufficient, no need to describe this for each predictor separately.)
If applicable, how was censoring accounted for?	______________________ (If not applicable, fill this with "NA")
Testing of the proportionality of hazards in survival models is described	◯ Yes<br>◯ No<br>◯ NA
What shrinkage/penalisation methods were used?	◯ None<br>◯ Uniform shrinkage<br>◯ Penalised estimation<br>◯ Other
If other, please specify	______________________

Model 8

The type of statistical modelling approach is reported	◯ Yes<br>◯ No<br>(E.g. Neural Network)
What is the ML technique being used?	◯ Neural network<br>◯ Random forest<br>◯ Classification and regression tree (CART)<br>◯ Support vector machine<br>◯ Gradient boosting machine<br>◯ Logistic regression<br>◯ Other
If other, please specify	______________________

For peer review only

Do they clearly state why the technique is selected?	__________________________ (E.g. R code availability, clinical question, previous literature, comparison. If not reported, fill this with "NR")
The approach used for predictor selection before modelling is described	◯ Yes<br>◯ No<br>◯ NA<br>(Before modelling' means before any univariable or multivariable analysis of predictor-outcome associations. If no predictor selection before modelling is done, score Not applicable. If it is unclear whether predictor selection before modelling is done, score No. If it is clear there was predictor selection before modelling but the method was not described, score No.)
The approach used for predictor selection during modelling is described	◯ Yes<br>◯ No<br>◯ NA<br>(E.g. Univariable analysis, stepwise selection, bootstrap, Lasso. 'During modelling' includes both univariable or multivariable analysis of predictor-outcome associations. If no predictor selection during modelling is done (so-called full model approach), score Not applicable. If it is unclear whether predictor selection during modelling is done, score No. If it is clear there was predictor selection during modelling but the method was not described, score No.)
What was the model building strategy?	◯ Stepwise<br>◯ Forward selection<br>◯ Backward selection<br>◯ All predictors<br>◯ All significant in univariable<br>◯ Data-driven<br>◯ Other<br>◯ Unclear
If other, please specify	__________________________ (list using (;) to separate if more than 1)
Are hyper-parameters tuning reported?	◯ Yes<br>◯ No<br>◯ NA<br>(Answer yes if any information is giving about how the models were set-up. Term as number of layers, node, optimization, hyperparameters, etc.)
Is predictor importance assessed?	◯ Yes<br>◯ No<br>(E.g. Importance, Mean decrease/Increase in accuracy, Mean decrease Gini, Gini Index, Average impurity decrease, etc.)

For peer review only

If yes, how?	______________________<br>(list using (;) to separate if more than 1)
Is there any ML technique being used for predictors/feature selection?	◯ Yes<br>◯ No<br>(E.g. SVM)
If yes, which technique?	______________________<br>(list using (;) to separate if more than 1)
Testing of interaction terms is described	◯ Yes, implicitly.<br>◯ Yes, explicitly.<br>◯ No<br>(Interaction terms may not be explicitly reported/planned but handled within the model building process for ML and maybe implicit/unplanned. If an ML model is being evaluated and interactions are not explicitly reported - choose 'Yes (implicit/unplanned)'. A general statement is sufficient, no need to describe this for each predictor separately.)
If applicable, how was censoring accounted for?	______________________<br>(If not applicable, fill this with "NA")
Testing of the proportionality of hazards in survival models is described	◯ Yes<br>◯ No<br>◯ NA
What shrinkage/penalisation methods were used?	◯ None<br>◯ Uniform shrinkage<br>◯ Penalised estimation<br>◯ Other
If other, please specify	______________________

Model 9

The type of statistical modelling approach is reported	◯ Yes<br>◯ No<br>(E.g. Neural Network)
What is the ML technique being used?	◯ Neural network<br>◯ Random forest<br>◯ Classification and regression tree (CART)<br>◯ Support vector machine<br>◯ Gradient boosting machine<br>◯ Logistic regression<br>◯ Other
If other, please specify	______________________

For peer review only

Do they clearly state why the technique is selected?	__________________________________<br>(E.g. R code availability, clinical question, previous literature, comparison. If not reported, fill this with "NR")
The approach used for predictor selection before modelling is described	◯ Yes<br>◯ No<br>◯ NA<br>(Before modelling' means before any univariable or multivariable analysis of predictor-outcome associations. If no predictor selection before modelling is done, score Not applicable. If it is unclear whether predictor selection before modelling is done, score No. If it is clear there was predictor selection before modelling but the method was not described, score No.)
The approach used for predictor selection during modelling is described	◯ Yes<br>◯ No<br>◯ NA<br>(E.g. Univariable analysis, stepwise selection, bootstrap, Lasso. 'During modelling' includes both univariable or multivariable analysis of predictor-outcome associations. If no predictor selection during modelling is done (so-called full model approach), score Not applicable. If it is unclear whether predictor selection during modelling is done, score No. If it is clear there was predictor selection during modelling but the method was not described, score No.)
What was the model building strategy?	◯ Stepwise<br>◯ Forward selection<br>◯ Backward selection<br>◯ All predictors<br>◯ All significant in univariable<br>◯ Data-driven<br>◯ Other<br>◯ Unclear
If other, please specify	__________________________________<br>(list using (;) to separate if more than 1)
Are hyper-parameters tuning reported?	◯ Yes<br>◯ No<br>◯ NA<br>(Answer yes if any information is giving about how the models were set-up. Term as number of layers, node, optimization, hyperparameters, etc.)
Is predictor importance assessed?	◯ Yes<br>◯ No<br>(E.g. Importance, Mean decrease/Increase in accuracy, Mean decrease Gini, Gini Index, Average impurity decrease, etc.)

For peer review only

If yes, how?	______________________ (list using (;) to separate if more than 1)
Is there any ML technique being used for predictors/feature selection?	◯ Yes<br>◯ No<br>(E.g. SVM)
If yes, which technique?	______________________ (list using (;) to separate if more than 1)
Testing of interaction terms is described	◯ Yes, implicitly.<br>◯ Yes, explicitly.<br>◯ No<br>(Interaction terms may not be explicitly reported/planned but handled within the model building process for ML and maybe implicit/unplanned. If an ML model is being evaluated and interactions are not explicitly reported - choose 'Yes (implicit/unplanned)'. A general statement is sufficient, no need to describe this for each predictor separately.)
If applicable, how was censoring accounted for?	______________________ (If not applicable, fill this with "NA")
Testing of the proportionality of hazards in survival models is described	◯ Yes<br>◯ No<br>◯ NA
What shrinkage/penalisation methods were used?	◯ None<br>◯ Uniform shrinkage<br>◯ Penalised estimation<br>◯ Other
If other, please specify	______________________

Model 10

The type of statistical modelling approach is reported	◯ Yes<br>◯ No<br>(E.g. Neural Network)
What is the ML technique being used?	◯ Neural network<br>◯ Random forest<br>◯ Classification and regression tree (CART)<br>◯ Support vector machine<br>◯ Gradient boosting machine<br>◯ Logistic regression<br>◯ Other
If other, please specify	______________________

For peer review only

Do they clearly state why the technique is selected?	______________________ (E.g. R code availability, clinical question, previous literature, comparison. If not reported, fill this with "NR")
The approach used for predictor selection before modelling is described	◯ Yes<br>◯ No<br>◯ NA<br>(Before modelling' means before any univariable or multivariable analysis of predictor-outcome associations. If no predictor selection before modelling is done, score Not applicable. If it is unclear whether predictor selection before modelling is done, score No. If it is clear there was predictor selection before modelling but the method was not described, score No.)
The approach used for predictor selection during modelling is described	◯ Yes<br>◯ No<br>◯ NA<br>(E.g. Univariable analysis, stepwise selection, bootstrap, Lasso. 'During modelling' includes both univariable or multivariable analysis of predictor-outcome associations. If no predictor selection during modelling is done (so-called full model approach), score Not applicable. If it is unclear whether predictor selection during modelling is done, score No. If it is clear there was predictor selection during modelling but the method was not described, score No.)
What was the model building strategy?	◯ Stepwise<br>◯ Forward selection<br>◯ Backward selection<br>◯ All predictors<br>◯ All significant in univariable<br>◯ Data-driven<br>◯ Other<br>◯ Unclear
If other, please specify	______________________ (list using (;) to separate if more than 1)
Are hyper-parameters tuning reported?	◯ Yes<br>◯ No<br>◯ NA<br>(Answer yes if any information is giving about how the models were set-up. Term as number of layers, node, optimization, hyperparameters, etc.)
Is predictor importance assessed?	◯ Yes<br>◯ No<br>(E.g. Importance, Mean decrease/Increase in accuracy, Mean decrease Gini, Gini Index, Average impurity decrease, etc.)

For peer review only

If yes, how?	______________________ (list using (;) to separate if more than 1)
Is there any ML technique being used for predictors/feature selection?	◯ Yes<br>◯ No<br>(E.g. SVM)
If yes, which technique?	______________________ (list using (;) to separate if more than 1)
Testing of interaction terms is described	◯ Yes, implicitly.<br>◯ Yes, explicitly.<br>◯ No<br>(Interaction terms may not be explicitly reported/planned but handled within the model building process for ML and maybe implicit/unplanned. If an ML model is being evaluated and interactions are not explicitly reported - choose 'Yes (implicit/unplanned)'. A general statement is sufficient, no need to describe this for each predictor separately.)
If applicable, how was censoring accounted for?	______________________ (If not applicable, fill this with "NA")
Testing of the proportionality of hazards in survival models is described	◯ Yes<br>◯ No<br>◯ NA
What shrinkage/penalisation methods were used?	◯ None<br>◯ Uniform shrinkage<br>◯ Penalised estimation<br>◯ Other
If other, please specify	______________________

Internal validation

Internal validation is reported	◯ Yes<br>◯ No<br>(If the use of internal validation is clearly a non-issue (e.g. in case of very large data sets), score Yes. For all other situations an explicit mention is expected.)
How is the model internally validated?	☐ Split sample<br>☐ Bootstrapping<br>☐ Cross-validation<br>☐ Other<br>☐ Unclear
If split sample, what % split was used for the development?	______________________ (If not reported, please fill this with "UN")

For peer review only

If split sample, was it a random or non-random split?	◯ Random<br>◯ Non-random<br>◯ Unclear
If non-random split was used, how?	__________________<br>(E.g. Temporal splitting)
If bootstrap method, how many were performed?	__________________<br>(If not reported, please fill this with "UN")
If bootstrap method, were selection of variables included in the bootstrap?	◯ Yes<br>◯ No
If cross validation, please specify the method used	__________________<br>(E.g. ten-fold. If not reported, fill this with "UN")

Model Performance

Development<br>Measures for model discrimination are described	◯ Yes<br>◯ No
External validation<br>Measures for model discrimination are described	◯ Yes<br>◯ No
External validation<br>Measures for model discrimination are described	◯ Yes<br>◯ No
Development<br>How was discrimination assessed?	☐ AUC/ AUROC/Area under the curve<br>☐ C-statistic<br>☐ Harrell's C-index<br>☐ D-Statistic<br>☐ Other
External validation<br>How was discrimination assessed?	☐ AUC/ AUROC/Area under the curve<br>☐ C-statistic<br>☐ Harrell's C-index<br>☐ D-Statistic<br>☐ Other
External validation<br>How was discrimination assessed?	☐ AUC/ AUROC/Area under the curve<br>☐ C-statistic<br>☐ Harrell's C-index<br>☐ D-Statistic<br>☐ Other
If other, please specify	__________________<br>(list using (;) to separate if more than 1)
If other, please specify	__________________<br>(list using (;) to separate if more than 1)

For peer review only

If other, please specify	______________________________ (list using (;) to separate if more than 1)
Development<br>Measures for model calibration are described	◯ Yes<br>◯ No
External validation<br>Measures for model calibration are described	◯ Yes<br>◯ No
External validation<br>Measures for model calibration are described	◯ Yes<br>◯ No
Development<br>How was calibration assessed?	☐ H-L<br>☐ Calibration plot<br>☐ Calibration slope<br>☐ Calibration intercept<br>☐ Calibration in the large<br>☐ Calibration table<br>☐ Kappa<br>☐ Observed/expected ratio<br>☐ Other
External validation<br>How was calibration assessed?	☐ H-L<br>☐ Calibration plot<br>☐ Calibration slope<br>☐ Calibration intercept<br>☐ Calibration in the large<br>☐ Calibration table<br>☐ Kappa<br>☐ Observed/expected ratio<br>☐ Other
External validation<br>How was calibration assessed?	☐ H-L<br>☐ Calibration plot<br>☐ Calibration slope<br>☐ Calibration intercept<br>☐ Calibration in the large<br>☐ Calibration table<br>☐ Kappa<br>☐ Observed/expected ratio<br>☐ Other
If other, please specify	______________________________
If other, please specify	______________________________
If other, please specify	______________________________

For peer review only

Development<br>Other performance measures are described	◯ Yes<br>◯ No<br>(E.g. R2, Brier score, predictive values, sensitivity, specificity, AUC difference, decision curve analysis, net reclassification improvement, integrated discrimination improvement, AIC)
External validation<br>Other performance measures are described	◯ Yes<br>◯ No<br>(E.g. R2, Brier score, predictive values, sensitivity, specificity, AUC difference, decision curve analysis, net reclassification improvement, integrated discrimination improvement, AIC)
External validation<br>Other performance measures are described	◯ Yes<br>◯ No<br>(E.g. R2, Brier score, predictive values, sensitivity, specificity, AUC difference, decision curve analysis, net reclassification improvement, integrated discrimination improvement, AIC)
Development<br>If yes, please specify	☐ R2<br>☐ Brier score<br>☐ predictive values<br>☐ sensitivity<br>☐ specificity<br>☐ AUC difference<br>☐ decision curve analysis<br>☐ net reclassification improvement<br>☐ integrated discrimination improvement<br>☐ AIC<br>☐ Other
External validation<br>If yes, please specify	☐ R2<br>☐ Brier score<br>☐ predictive values<br>☐ sensitivity<br>☐ specificity<br>☐ AUC difference<br>☐ decision curve analysis<br>☐ net reclassification improvement<br>☐ integrated discrimination improvement<br>☐ AIC<br>☐ Other
External validation<br>If yes, please specify	☐ R2<br>☐ Brier score<br>☐ predictive values<br>☐ sensitivity<br>☐ specificity<br>☐ AUC difference<br>☐ decision curve analysis<br>☐ net reclassification improvement<br>☐ integrated discrimination improvement<br>☐ AIC<br>☐ Other

For peer review only

If other, please specify	________________________
If other, please specify	________________________
If other, please specify	________________________

Model Updating

A description of model-updating is given	◯ Yes<br>◯ No<br>◯ NA<br>(E.g. Intercept recalibration, regression coefficient recalibration, refitting the whole model, adding a new predictor If updating was done, it should be clear which updating method was applied to score Yes. If it is not explicitly mentioned that updating was applied in the study, score this item as 'Not applicable'.)
If yes, please specify	________________________<br>(E.g. Intercept recalibration, regression coefficient recalibration, refitting the whole model, adding a new predictor.)

Risk groups

Development<br>Were risk groups created?	◯ Yes<br>◯ No
External validation<br>Were risk groups created?	◯ Yes<br>◯ No
External validation<br>Were risk groups created?	◯ Yes<br>◯ No
How many risk groups were created?	________________________
How many risk groups were created?	________________________
How many risk groups were created?	________________________
Development<br>What method was used to create these risk groups?	☐ Count factors present<br>☐ Data driven<br>☐ Equal size<br>☐ Other data dependent<br>☐ Unclear

For peer review only

External validation<br>What method was used to create these risk groups?	☐ Count factors present<br>☐ Data driven<br>☐ Equal size<br>☐ Other data dependent<br>☐ Unclear
External validation<br>What method was used to create these risk groups?	☐ Count factors present<br>☐ Data driven<br>☐ Equal size<br>☐ Other data dependent<br>☐ Unclear
Development<br>If risk groups were created, risk group boundaries (risk thresholds) are specified	◯ Yes<br>◯ No<br>(Score this item separately for development and validation if a study includes both development and validation.)
External validation<br>If risk groups were created, risk group boundaries (risk thresholds) are specified	◯ Yes<br>◯ No<br>(Score this item separately for development and validation if a study includes both development and validation.)
External validation<br>If risk groups were created, risk group boundaries (risk thresholds) are specified	◯ Yes<br>◯ No<br>(Score this item separately for development and validation if a study includes both development and validation.)
Development<br>Is any subgroup analysis prespecified?	◯ Yes<br>◯ No
External validation<br>Is any subgroup analysis prespecified?	◯ Yes<br>◯ No
External validation<br>Is any subgroup analysis prespecified?	◯ Yes<br>◯ No
If yes, how many subgroup criteria were defined?	______________________
If yes, how many subgroup criteria were defined?	______________________
If yes, how many subgroup criteria were defined?	______________________
Development<br>Is any sensitivity/subpopulation analysis prespecified?	◯ Yes<br>◯ No
External validation<br>Is any sensitivity/subpopulation analysis prespecified?	◯ Yes<br>◯ No

For peer review only

External validation<br>Is any sensitivity/subpopulation analysis prespecified?	◯ Yes<br>◯ No
Is yes, how many subpopulation criteria area defined?	__________________________________
Is yes, how many subpopulation criteria area defined?	__________________________________
Is yes, how many subpopulation criteria area defined?	__________________________________

Comments

Any additional comment about the methods section of this article?	__________________________________<br>(If there is something in the "Methods" section that does not fit into the questions of this form - please use this space to detail. Also use this space to detail anything you are unsure about.)

For peer review only

Results

Article ID	__________________________
What type of study is reported?	◯ Development (including internal validation)<br>◯ Development with external validation (same model)<br>◯ Development with external validation (different model)<br>◯ External validation only<br>(This questions is repeated due to branching logic. Please answer again.)
Is there any diagram/draw to clarify the results?	◯ Yes<br>◯ No
**Participants**	
Development<br>The flow of participants is reported	◯ Yes<br>◯ No
External validation<br>The flow of participants is reported	◯ Yes<br>◯ No
External validation<br>The flow of participants is reported	◯ Yes<br>◯ No
Development<br>The number of participants with and without the outcome is reported	◯ Yes<br>◯ No<br>◯ NA<br>(If outcomes are continuous, score Not applicable.)
External validation<br>The number of participants with and without the outcome is reported	◯ Yes<br>◯ No<br>◯ NA<br>(If outcomes are continuous, score Not applicable.)
External validation<br>The number of participants with and without the outcome is reported	◯ Yes<br>◯ No<br>◯ NA<br>(If outcomes are continuous, score Not applicable.)
Development<br>A summary of follow-up time is presented	◯ Yes (median)<br>◯ Yes (average)<br>◯ Other<br>◯ No<br>◯ NA<br>(This notably applies to prognosis studies and diagnostic studies with follow-up as diagnostic outcome. If this is not applicable for an article (i.e. diagnostic study or no follow-up), then score Not applicable.)

For peer review only

External validation<br>A summary of follow-up time is presented	◯ Yes (median)<br>◯ Yes (average)<br>◯ Other<br>◯ No<br>◯ NA<br>(This notably applies to prognosis studies and diagnostic studies with follow-up as diagnostic outcome. If this is not applicable for an article (i.e. diagnostic study or no follow-up), then score Not applicable.)
External validation<br>A summary of follow-up time is presented	◯ Yes (median)<br>◯ Yes (average)<br>◯ Other<br>◯ No<br>◯ NA<br>(This notably applies to prognosis studies and diagnostic studies with follow-up as diagnostic outcome. If this is not applicable for an article (i.e. diagnostic study or no follow-up), then score Not applicable.)
If other, please specify	____________________<br>(list using (;) to separate if more than 1)
If other, please specify	____________________<br>(list using (;) to separate if more than 1)
If other, please specify	____________________<br>(list using (;) to separate if more than 1)
Development<br>For time-to-event endpoints, do the authors report how many have X-years of follow-up?	◯ Yes<br>◯ No<br>◯ NA
External validation<br>For time-to-event endpoints, do the authors report how many have X-years of follow-up?	◯ Yes<br>◯ No<br>◯ NA
Development<br>Basic demographics are reported	◯ Yes<br>◯ No<br>(At least age and sex are reported.)
External validation<br>Basic demographics are reported	◯ Yes<br>◯ No<br>(At least age and sex are reported.)
External validation<br>Basic demographics are reported	◯ Yes<br>◯ No<br>(At least age and sex are reported.)
Development<br>Number of predictors in the final model	____________________<br>(If the final predictors are unclear, please fill this question with a 'UN')

For peer review only

External validation
Number of predictors in the final model

____
(If the final predictors are unclear, please fill this question with a 'UN')

External validation
Number of predictors in the final model

____
(If the final predictors are unclear, please fill this question with a 'UN')

Development
Summary information is provided for all predictors included in the final developed/validated model

◯ Yes
◯ No

External validation
Summary information is provided for all predictors included in the final developed/validated model

◯ Yes
◯ No

External validation
Summary information is provided for all predictors included in the final developed/validated model

◯ Yes
◯ No

Development
The number of participants with missing data for predictors is reported

◯ Yes
◯ No
◯ NA
(When no missing values is reported, fill this with "NA")

External validation
The number of participants with missing data for predictors is reported

◯ Yes
◯ No
◯ NA
(When no missing values is reported, fill this with "NA")

External validation
The number of participants with missing data for predictors is reported

◯ Yes
◯ No
◯ NA
(When no missing values is reported, fill this with "NA")

Development
Final number of models developed/validated reported

____
(Please provide the number. If this is unclear, please fill this with "UN")

External validation
Final number of models developed/validated reported

____
(Please provide the number. If this is unclear, please fill this with "UN")

External validation
Final number of models developed/validated reported

____
(Please provide the number. If this is unclear, please fill this with "UN")

For peer review only

External validation<br>Demographic characteristics (at least age and gender) of the validation study participants are reported along with those of the original development study	◯ Yes<br>◯ No<br>◯ NA<br>(For incremental value reports, in case additional predictors are not added to a previously developed prediction model but rather added to conventional predictors in a newly fitted model, score Not applicable.)
External validation<br>Distributions of predictors in the model of the validation study participants are reported along with those of the original development study	◯ Yes<br>◯ No<br>◯ NA<br>(For incremental value reports, in case additional predictors are not added to a previously developed prediction model but rather added to conventional predictors in a newly fitted model, score Not applicable.)
External validation<br>Outcomes of the validation study participants are reported along with those of the original development study	◯ Yes<br>◯ No<br>◯ NA<br>(For incremental value reports, in case additional predictors are not added to a previously developed prediction model but rather added to conventional predictors in a newly fitted model, score Not applicable)

Model development

The number of participants in each analysis is specified	◯ Yes<br>◯ No<br>(e.g. in the analysis of each model if more than one model is developed)
What is the number of participants (included in the analysis) reported in the main model?	________________<br>(If the number of participants is not reported, please fill this with 'NR'.)
The number of outcome events in each analysis is specified	◯ Yes<br>◯ No<br>◯ NA<br>(e.g. in the analysis of each model if more than one model is developed. If outcomes are continuous, score Not applicable.)
What is the number of events (initial) reported in the main outcome?	________________<br>(If the number of events is not reported, please fill this with 'NR'.)
What is the number of events (included in the analysis) reported in the main model?	________________<br>(If the number of events is not reported, please fill this with 'NR'.)

For peer review only

The unadjusted associations between each predictor and outcome are reported	◯ Yes<br>◯ No<br>◯ NA<br>(If any univariable analysis is mentioned in the methods but not in the results, score No. If nothing on univariable analysis (in methods or results) is reported, score this item as Not applicable)

Model specification

Development<br>The regression coefficient (or a derivative such as hazard ratio, odds ratio, risk ratio) for each predictor in the model is reported	◯ Yes<br>◯ No<br>◯ NA
Development<br>The intercept or the cumulative baseline hazard (or baseline survival) for at least one time point is reported	◯ Yes<br>◯ No<br>◯ NA
Development<br>An explanation (e.g. a simplified scoring rule, chart, nomogram of the model, reference to online calculator, or worked example) is provided to explain how to use the model for individualised predictions.	◯ Yes<br>◯ No
Development<br>Is there enough information to calculate the risk of the outcome in a new individual?	◯ Yes<br>◯ No

Model Performance

Instructions

-Please extract the models in the order they are presented in the article.

-If more than 10 models were developed for the main outcome, only refer to the first 10.

-If a comparison with logistic regression was made, please include this model in the final count and extract information.

How many models were developed for the primary outcome?	____________________<br>(This should reflect the number of models you are going to extract on - primary outcome and primary timepoint. If more than 10 models were developed, please refer to the first 10 models. If a Logistic regression model was performed, please also extract data from this model)

For peer review only - http://bmjopen.bmj.com/site/about/guidelines.xhtml

Model 1

Is this the recommended model?	◯ Yes<br>◯ No<br>(If there is only one model, please state 'Yes')
Development<br>A discrimination measure is presented	◯ Yes<br>◯ No<br>(E.g. C-index / area under the ROC curve)
External validation<br>A discrimination measure is presented	◯ Yes<br>◯ No<br>(E.g. C-index / area under the ROC curve)
Development<br>Which discrimination measures are described?	☐ AUC/AUROC/Area under the curve<br>☐ C-statistic<br>☐ Harrell's c-index<br>☐ D-statistic
External validation<br>Which discrimination measures are described?	☐ AUC/AUROC/Area under the curve<br>☐ C-statistic<br>☐ Harrell's c-index<br>☐ D-statistic
The confidence interval (or standard error) of the AUC/AUROC/Area under the curve is presented	◯ Yes<br>◯ No
The confidence interval (or standard error) of the AUC/AUROC/Area under the curve is presented	◯ Yes<br>◯ No
What was the AUC/AUROC/Area under the curve apparent discrimination estimate (+95% CI, if given)?	____________________<br>(Please use the following format - estimate (lower CI - upper CI). For example 0.79 (0.75 - 0.81). If not reported fill this with "NR")
What was the AUC/AUROC/Area under the curve apparent discrimination estimate (+95% CI, if given)?	____________________<br>(Please use the following format - estimate (lower CI - upper CI). For example 0.79 (0.75 - 0.81). If not reported fill this with "NR")
Was the AUC/AUROC/Area under the curve corrected for optimism?	◯ Yes<br>◯ No<br>◯ Unclear
What was the optimism corrected AUC/AUROC/Area under the curve (+95% CI, if given)?	____________________<br>(Please use the following format - estimate (lower CI - upper CI). For example 0.79 (0.75 - 0.81). If not reported fill this with "NR")
The confidence interval (or standard error) of the C-statistic is presented	◯ Yes<br>◯ No
The confidence interval (or standard error) of the C-statistic is presented	◯ Yes<br>◯ No

For peer review only

What was the C-statistic apparent discrimination estimate (+95% CI, if given)?	__________________ (Please use the following format - estimate (lower CI - upper CI). For example 0.79 (0.75 - 0.81). If not reported fill this with "NR")
What was the C-statistic apparent discrimination estimate (+95% CI, if given)?	__________________ (Please use the following format - estimate (lower CI - upper CI). For example 0.79 (0.75 - 0.81). If not reported fill this with "NR")
Was the C-statistic corrected for optimism?	◯ Yes<br>◯ No<br>◯ Unclear
What was the optimism corrected C-statistic (+95% CI, if given)?	__________________ (Please use the following format - estimate (lower CI - upper CI). For example 0.79 (0.75 - 0.81). If not reported fill this with "NR")
The confidence interval (or standard error) of the Harrell's c-index is presented	◯ Yes<br>◯ No
The confidence interval (or standard error) of the Harrell's c-index is presented	◯ Yes<br>◯ No
What was the Harrell's c-index apparent discrimination estimate (+95% CI, if given)?	__________________ (Please use the following format - estimate (lower CI - upper CI). For example 0.79 (0.75 - 0.81). If not reported fill this with "NR")
What was the Harrell's c-index apparent discrimination estimate (+95% CI, if given)?	__________________ (Please use the following format - estimate (lower CI - upper CI). For example 0.79 (0.75 - 0.81). If not reported fill this with "NR")
Was the Harrell's c-index corrected for optimism?	◯ Yes<br>◯ No<br>◯ Unclear
What was the optimism corrected Harrell's c-index (+95% CI, if given)?	__________________ (Please use the following format - estimate (lower CI - upper CI). For example 0.79 (0.75 - 0.81). If not reported fill this with "NR")
The confidence interval (or standard error) of the d-statistic presented	◯ Yes<br>◯ No
The confidence interval (or standard error) of the d-statistic presented	◯ Yes<br>◯ No

For peer review only

What was the d-statistic apparent discrimination estimate (+95% CI, if given)?	__________________________________ (Please use the following format - estimate (lower CI - upper CI). For example 0.79 (0.75 - 0.81). If not reported fill this with "NR")
What was the d-statistic apparent discrimination estimate (+95% CI, if given)?	__________________________________ (Please use the following format - estimate (lower CI - upper CI). For example 0.79 (0.75 - 0.81). If not reported fill this with "NR")
Was the d-statistic corrected for optimism?	◯ Yes<br>◯ No<br>◯ Unclear
What was the optimism corrected d-statistic (+95% CI, if given)?	__________________________________ (Please use the following format - estimate (lower CI - upper CI). For example 0.79 (0.75 - 0.81). If not reported fill this with "NR")
Development<br>Measures for model calibration are described	◯ Yes<br>◯ No<br>(E.g. calibration plot, calibration slope or intercept, calibration table, Hosmer Lemeshow test, O/E ratio.)
External validation<br>Measures for model calibration are described	◯ Yes<br>◯ No<br>(E.g. calibration plot, calibration slope or intercept, calibration table, Hosmer Lemeshow test, O/E ratio.)
Development<br>How was calibration assessed?	☐ Calibration plot<br>☐ Calibration slope<br>☐ Calibration intercept<br>☐ Calibration in the large<br>☐ Calibration table<br>☐ Kappa<br>☐ Observed/expected ratio<br>☐ H-L
External validation<br>How was calibration assessed?	☐ Calibration plot<br>☐ Calibration slope<br>☐ Calibration intercept<br>☐ Calibration in the large<br>☐ Calibration table<br>☐ Kappa<br>☐ Observed/expected ratio<br>☐ H-L
What was the apparent [gen_methods_103:checked] estimate (+95% CI, if given)?	__________________________________ (list using (;) to separate if more than 1. If not reported, fill this with "NR")

For peer review only

What was the apparent [gen_methods_117:checked] estimate (+95% CI, if given)?	__________________ (list using (;) to separate if more than 1. If not reported, fill this with "NR")
Was the [gen_methods_103:checked] measure corrected for optimism?	◯ Yes<br>◯ No<br>◯ Unclear
What was the optimism corrected [gen_methods_103:checked] estimate (+95% CI, if given)?	__________________ (list using (;) to separate if more than 1. If measures were corrected for optimism but not reported, please fill this with "NR".)
Development<br>Other model performance measures are presented	◯ Yes<br>◯ No<br>(E.g. R2, Brier score, predictive values, sensitivity, specificity, AUC difference, decision curve analysis, net reclassification improvement, integrated discrimination improvement, AIC.)
External validation<br>Other model performance measures are presented	◯ Yes<br>◯ No<br>(E.g. R2, Brier score, predictive values, sensitivity, specificity, AUC difference, decision curve analysis, net reclassification improvement, integrated discrimination improvement, AIC.)
Development<br>If yes, please specify	☐ R2<br>☐ Brier score<br>☐ predictive values<br>☐ sensitivity<br>☐ specificity<br>☐ AUC difference<br>☐ decision curve analysis<br>☐ net reclassification improvement<br>☐ integrated discrimination improvement<br>☐ AIC<br>☐ Accuracy<br>☐ Other<br>(Multiples answers are possible)
External validation<br>If yes, please specify	☐ R2<br>☐ Brier score<br>☐ predictive values<br>☐ sensitivity<br>☐ specificity<br>☐ AUC difference<br>☐ decision curve analysis<br>☐ net reclassification improvement<br>☐ integrated discrimination improvement<br>☐ AIC<br>☐ Accuracy<br>☐ Other<br>(Multiples answers are possible)

For peer review only

If other, please specify	__________________________________ (list the names using (;) to separate if more than 1)
If other, please specify	__________________________________ (list the names using (;) to separate if more than 1)
What was the apparent [gen_methods_104:checked] estimate (+95% CI, if given)?	__________________________________ (list using (;) to separate if more than 1. If not reported, fill this with "NR")
What was the apparent [gen_methods_118:checked] estimate (+95% CI, if given)?	__________________________________ (list using (;) to separate if more than 1. If not reported, fill this with "NR")
Was the [gen_methods_104:checked] measure corrected for optimism?	◯ Yes<br>◯ No<br>◯ Unclear
What was the optimism corrected [gen_methods_104:checked] estimate (+95% CI, if given)?	__________________________________ (list using (;) to separate if more than 1. If not reported, fill this with "NR")

Model 2

Is this the recommended model?	◯ Yes<br>◯ No<br>(If there is only one model, please state 'Yes')
Development<br>A discrimination measure is presented	◯ Yes<br>◯ No<br>(E.g. C-index / area under the ROC curve)
External validation<br>A discrimination measure is presented	◯ Yes<br>◯ No<br>(E.g. C-index / area under the ROC curve)
Development<br>Which performance measures are described?	☐ AUC/AUROC/Area under the curve<br>☐ C-statistic<br>☐ Harrell's c-index<br>☐ D-statistic<br>(Multiples answers are possible)
External validation<br>Which performance measures are described?	☐ AUC/AUROC/Area under the curve<br>☐ C-statistic<br>☐ Harrell's c-index<br>☐ D-statistic<br>(Multiples answers are possible)

For peer review only

The confidence interval (or standard error) of the AUC/AUROC/Area under the curve is presented	◯ Yes<br>◯ No
The confidence interval (or standard error) of the AUC/AUROC/Area under the curve is presented	◯ Yes<br>◯ No
What was the AUC/AUROC/Area under the curve apparent discrimination estimate (+95%CI, if given)?	__________________<br>(Please use the following format - estimate (lower CI - upper CI). For example 0.79 (0.75 - 0.81). If not reported, fill this with "NR")
What was the AUC/AUROC/Area under the curve apparent discrimination estimate (+95%CI, if given)?	__________________<br>(Please use the following format - estimate (lower CI - upper CI). For example 0.79 (0.75 - 0.81). If not reported fill this with "NR")
Was the AUC/AUROC/Area under the curve corrected for optimism?	◯ Yes<br>◯ No<br>◯ Unclear
What was the optimism corrected AUC/AUROC/Area under the curve (95% CI, if given)?	__________________<br>(Please use the following format - estimate (lower CI - upper CI). For example 0.79 (0.75 - 0.81). If not reported fill this with "NR")
The confidence interval (or standard error) of the C-statistics is presented	◯ Yes<br>◯ No
The confidence interval (or standard error) of the C-statistics is presented	◯ Yes<br>◯ No
What was the C-statistics apparent discrimination estimate (+95%CI, if given)?	__________________<br>(Please use the following format - estimate (lower CI - upper CI). For example 0.79 (0.75 - 0.81). If not reported fill this with "NR")
What was the C-statistics apparent discrimination estimate (+95%CI, if given)?	__________________<br>(Please use the following format - estimate (lower CI - upper CI). For example 0.79 (0.75 - 0.81). If not reported fill this with "NR")
Was the C-statistics corrected for optimism?	◯ Yes<br>◯ No<br>◯ Unclear
What was the optimism corrected C-statistic (95% CI, if given)?	__________________<br>(Please use the following format - estimate (lower CI - upper CI). For example 0.79 (0.75 - 0.81). If not reported fill this with "NR")
The confidence interval (or standard error) of the Harrel's c-index is presented	◯ Yes<br>◯ No

For peer review only

The confidence interval (or standard error) of the Harrel's c-index is presented	◯ Yes<br>◯ No
What was the Harrel's c-index apparent discrimination estimate (+95%CI, if given)?	__________________<br>(Please use the following format - estimate (lower CI - upper CI). For example 0.79 (0.75 - 0.81). If not reported fill this with "NR")
What was the Harrel's c-index apparent discrimination estimate (+95%CI, if given)?	__________________<br>(Please use the following format - estimate (lower CI - upper CI). For example 0.79 (0.75 - 0.81). If not reported fill this with "NR")
Was the Harrel's c-index corrected for optimism?	◯ Yes<br>◯ No<br>◯ Unclear
What was the optimism corrected Harrel's c-index (95% CI, if given)?	__________________<br>(Please use the following format - estimate (lower CI - upper CI). For example 0.79 (0.75 - 0.81). If not reported fill this with "NR")
The confidence interval (or standard error) of the D-statistic is presented	◯ Yes<br>◯ No
The confidence interval (or standard error) of the D-statistic is presented	◯ Yes<br>◯ No
What was the D-statistics apparent discrimination estimate (+95%CI, if given)?	__________________<br>(Please use the following format - estimate (lower CI - upper CI). For example 0.79 (0.75 - 0.81). If not reported fill this with "NR")
What was the D-statistics apparent discrimination estimate (+95%CI, if given)?	__________________<br>(Please use the following format - estimate (lower CI - upper CI). For example 0.79 (0.75 - 0.81). If not reported fill this with "NR")
Was the D-statistics corrected for optimism?	◯ Yes<br>◯ No<br>◯ Unclear
What was the optimism corrected D-statistics (95% CI, if given)?	__________________<br>(Please use the following format - estimate (lower CI - upper CI). For example 0.79 (0.75 - 0.81). If not reported fill this with "NR")
Development<br>Measures for model calibration are described	◯ Yes<br>◯ No<br>(E.g. calibration plot, calibration slope or intercept, calibration table, Hosmer Lemeshow test, O/E ratio.)

For peer review only

External validation<br>Measures for model calibration are described	◯ Yes<br>◯ No<br>(E.g. calibration plot, calibration slope or intercept, calibration table, Hosmer Lemeshow test, O/E ratio.)
Development<br>How was calibration assessed?	☐ Calibration plot<br>☐ Calibration slope<br>☐ Calibration intercept<br>☐ Calibration in the large<br>☐ Calibration table<br>☐ Kappa<br>☐ Observed/expected ratio<br>☐ H-L
External validation<br>How was calibration assessed?	☐ Calibration plot<br>☐ Calibration slope<br>☐ Calibration intercept<br>☐ Calibration in the large<br>☐ Calibration table<br>☐ Kappa<br>☐ Observed/expected ratio<br>☐ H-L
What was the apparent [gen_methods_119:checked] estimate (+95% CI, if given)?	__________________________________<br>(list using (;) to separate if more than 1. If not reported, fill this with "NR")
What was the apparent [gen_methods_120:checked] estimate (+95% CI, if given)?	__________________________________<br>(list using (;) to separate if more than 1. If not reported, fill this with "NR")
Was the [gen_methods_119:checked] measure corrected for optimism?	◯ Yes<br>◯ No<br>◯ Unclear
What was the optimism corrected [gen_methods_119:checked] estimate (+95% CI, if given)?	__________________________________<br>(list using (;) to separate if more than 1. If not reported, fill this with "NR")
Development<br>Other model performance measures are presented	◯ Yes<br>◯ No<br>(E.g. R2, Brier score, predictive values, sensitivity, specificity, AUC difference, decision curve analysis, net reclassification improvement, integrated discrimination improvement, AIC.)
External validation<br>Other model performance measures are presented	◯ Yes<br>◯ No<br>(E.g. R2, Brier score, predictive values, sensitivity, specificity, AUC difference, decision curve analysis, net reclassification improvement, integrated discrimination improvement, AIC.)

For peer review only

Development<br>If yes, please specify	☐ R2<br>☐ Brier score<br>☐ predictive values<br>☐ sensitivity<br>☐ specificity<br>☐ AUC difference<br>☐ decision curve analysis<br>☐ net reclassification improvement<br>☐ integrated discrimination improvement<br>☐ AIC<br>☐ Accuracy<br>☐ Other<br>(Multiples answers are possible)
External validation<br>If yes, please specify	☐ R2<br>☐ Brier score<br>☐ predictive values<br>☐ sensitivity<br>☐ specificity<br>☐ AUC difference<br>☐ decision curve analysis<br>☐ net reclassification improvement<br>☐ integrated discrimination improvement<br>☐ AIC<br>☐ Accuracy<br>☐ Other<br>(Multiples answers are possible)
If other, please specify	__________________________________<br>(list using (;) to separate if more than 1)
If other, please specify	__________________________________<br>(list using (;) to separate if more than 1)
What was the apparent [gen_methods_121:checked] estimate (+95% CI, if given)?	__________________________________<br>(list using (;) to separate if more than 1. If not reported, fill this with "NR")
What was the apparent [gen_methods_122:checked] estimate (+95% CI, if given)?	__________________________________<br>(list using (;) to separate if more than 1. If not reported, fill this with "NR")
Was the [gen_methods_121:checked] measure corrected for optimism?	◯ Yes<br>◯ No<br>◯ Unclear
What was the optimism corrected [gen_methods_121:checked] estimate (+95% CI, if given)?	__________________________________<br>(list using (;) to separate if more than 1. If not reported, fill this with "NR")

For peer review only

Model 3

Is this the recommended model?	◯ Yes<br>◯ No<br>(If there is only one model, please state 'Yes')
Development<br>A discrimination measure is presented	◯ Yes<br>◯ No<br>(E.g. C-index / area under the ROC curve)
External validation<br>A discrimination measure is presented	◯ Yes<br>◯ No<br>(E.g. C-index / area under the ROC curve)
Development<br>Which performance measures are described?	☐ AUC/AUROC/Area under the curve<br>☐ C-statistic<br>☐ Harrell's c-index<br>☐ D-statistic<br>(Multiples answers are possible)
External validation<br>Which performance measures are described?	☐ AUC/AUROC/Area under the curve<br>☐ C-statistic<br>☐ Harrell's c-index<br>☐ D-statistic<br>(Multiples answers are possible)
The confidence interval (or standard error) of the AUC/AUROC/Area under the curve is presented	◯ Yes<br>◯ No
The confidence interval (or standard error) of the AUC/AUROC/Area under the curve is presented	◯ Yes<br>◯ No
What was the AUC/AUROC/Area under the curve apparent discrimination estimate (+95%CI, if given)?	________________________<br>(Please use the following format - estimate (lower CI - upper CI). For example 0.79 (0.75 - 0.81). If not reported fill this with "NR")
What was the AUC/AUROC/Area under the curve apparent discrimination estimate (+95%CI, if given)?	________________________<br>(Please use the following format - estimate (lower CI - upper CI). For example 0.79 (0.75 - 0.81). If not reported fill this with "NR")
Was the AUC/AUROC/Area under the curve corrected for optimism?	◯ Yes<br>◯ No<br>◯ Unclear
What was the optimism corrected AUC/AUROC/Area under the curve (95% CI, if given)?	________________________<br>(Please use the following format - estimate (lower CI - upper CI). For example 0.79 (0.75 - 0.81). If not reported fill this with "NR")
The confidence interval (or standard error) of the C-statistics is presented	◯ Yes<br>◯ No

For peer review only

The confidence interval (or standard error) of the C-statistics is presented	◯ Yes<br>◯ No
What was the C-statistics apparent discrimination estimate (+95%CI, if given)?	__________________<br>(Please use the following format - estimate (lower CI - upper CI). For example 0.79 (0.75 - 0.81). If not reported fill this with "NR")
What was the C-statistics apparent discrimination estimate (+95%CI, if given)?	__________________<br>(Please use the following format - estimate (lower CI - upper CI). For example 0.79 (0.75 - 0.81). If not reported fill this with "NR")
Was the C-statistics corrected for optimism?	◯ Yes<br>◯ No<br>◯ Unclear
What was the optimism corrected C-statistics (95% CI, if given)?	__________________<br>(Please use the following format - estimate (lower CI - upper CI). For example 0.79 (0.75 - 0.81). If not reported fill this with "NR")
The confidence interval (or standard error) of the Harrel's c-index is presented	◯ Yes<br>◯ No
The confidence interval (or standard error) of the Harrel's c-index is presented	◯ Yes<br>◯ No
What was the Harrel's c-index apparent discrimination estimate (+95%CI, if given)?	__________________<br>(Please use the following format - estimate (lower CI - upper CI). For example 0.79 (0.75 - 0.81). If not reported fill this with "NR")
What was the Harrel's c-index apparent discrimination estimate (+95%CI, if given)?	__________________<br>(Please use the following format - estimate (lower CI - upper CI). For example 0.79 (0.75 - 0.81). If not reported fill this with "NR")
Was the Harrel's c-index corrected for optimism?	◯ Yes<br>◯ No<br>◯ Unclear
What was the optimism corrected Harrel's c-index (95% CI, if given)?	__________________<br>(Please use the following format - estimate (lower CI - upper CI). For example 0.79 (0.75 - 0.81). If not reported fill this with "NR")
The confidence interval (or standard error) of the D-statistics is presented	◯ Yes<br>◯ No

For peer review only

The confidence interval (or standard error) of the D-statistics is presented	◯ Yes<br>◯ No
What was the D-statistics apparent discrimination estimate (+95%CI, if given)?	________________________<br>(Please use the following format - estimate (lower CI - upper CI). For example 0.79 (0.75 - 0.81). If not reported fill this with "NR")
What was the D-statistics apparent discrimination estimate (+95%CI, if given)?	________________________<br>(Please use the following format - estimate (lower CI - upper CI). For example 0.79 (0.75 - 0.81). If not reported fill this with "NR")
Was the D-statistics corrected for optimism?	◯ Yes<br>◯ No<br>◯ Unclear
What was the optimism corrected D-statistics (95% CI, if given)?	________________________<br>(Please use the following format - estimate (lower CI - upper CI). For example 0.79 (0.75 - 0.81). If not reported fill this with "NR")
Development<br>Measures for model calibration are described	◯ Yes<br>◯ No<br>(E.g. calibration plot, calibration slope or intercept, calibration table, Hosmer Lemeshow test, O/E ratio.)
External validation<br>Measures for model calibration are described	◯ Yes<br>◯ No<br>(E.g. calibration plot, calibration slope or intercept, calibration table, Hosmer Lemeshow test, O/E ratio.)
Development<br>How was calibration assessed?	☐ Calibration plot<br>☐ Calibration slope<br>☐ Calibration intercept<br>☐ Calibration in the large<br>☐ Calibration table<br>☐ Kappa<br>☐ Observed/expected ratio<br>☐ H-L
External validation<br>How was calibration assessed?	☐ Calibration plot<br>☐ Calibration slope<br>☐ Calibration intercept<br>☐ Calibration in the large<br>☐ Calibration table<br>☐ Kappa<br>☐ Observed/expected ratio<br>☐ H-L

For peer review only

What was the apparent [gen_methods_139:checked] estimate (+95% CI, if given)?	__________________ (Please use the following format - estimate (lower CI - upper CI). For example 0.79 (0.75 - 0.81). List using (;) if more than 1. If not reported, fill this with "NR")
What was the apparent [gen_methods_140:checked] estimate (+95% CI, if given)?	__________________ (Please use the following format - estimate (lower CI - upper CI). For example 0.79 (0.75 - 0.81). List using (;) if more than 1. If not reported, fill this with "NR")
Was the [gen_methods_139:checked] measure corrected for optimism?	◯ Yes<br>◯ No<br>◯ Unclear
What was the optimism corrected [gen_methods_139:checked] estimate (+95% CI, if given)?	__________________ (Please use the following format - estimate (lower CI - upper CI). For example 0.79 (0.75 - 0.81). List using (;) if more than 1. If not reported, fill this with "NR")
Development<br>Other model performance measures are presented	◯ Yes<br>◯ No<br>(E.g. R2, Brier score, predictive values, sensitivity, specificity, AUC difference, decision curve analysis, net reclassification improvement, integrated discrimination improvement, AIC.)
External validation<br>Other model performance measures are presented	◯ Yes<br>◯ No<br>(E.g. R2, Brier score, predictive values, sensitivity, specificity, AUC difference, decision curve analysis, net reclassification improvement, integrated discrimination improvement, AIC.)
Development<br>If yes, please specify	☐ R2<br>☐ Brier score<br>☐ predictive values<br>☐ sensitivity<br>☐ specificity<br>☐ AUC difference<br>☐ decision curve analysis<br>☐ net reclassification improvement<br>☐ integrated discrimination improvement<br>☐ AIC<br>☐ Accuracy<br>☐ Other<br>(Multiples answers are possible)

For peer review only

External validation<br>If yes, please specify	☐ R2<br>☐ Brier score<br>☐ predictive values<br>☐ sensitivity<br>☐ specificity<br>☐ AUC difference<br>☐ decision curve analysis<br>☐ net reclassification improvement<br>☐ integrated discrimination improvement<br>☐ AIC<br>☐ Accuracy<br>☐ Other<br>(Multiples answers are possible)
If other, please specify	________________________<br>(list using (;) to separate if more than 1)
If other, please specify	________________________<br>(list using (;) to separate if more than 1)
What was the apparent [gen_methods_123:checked] estimate (+95% CI, if given)?	________________________<br>(list using (;) to separate if more than 1. If not reported, fill this with "NR")
What was the apparent [gen_methods_123:checked] estimate (+95% CI, if given)?	________________________<br>(list using (;) to separate if more than 1. If not reported, fill this with "NR")
Was the [gen_methods_123:checked] measure corrected for optimism?	◯ Yes<br>◯ No<br>◯ Unclear
What was the optimism corrected [gen_methods_123:checked] estimate (+95% CI, if given)?	________________________<br>(list using (;) to separate if more than 1. If measures were corrected for optimism but not reported, please fill this with "NR".)

Model 4

Is this the recommended model?	◯ Yes<br>◯ No<br>(If there is only one model, please state 'Yes')
Development<br>A discrimination measure is presented	◯ Yes<br>◯ No<br>(E.g. C-index / area under the ROC curve)
External validation<br>A discrimination measure is presented	◯ Yes<br>◯ No<br>(E.g. C-index / area under the ROC curve)

For peer review only

Development<br>Which performance measures are described?	☐ AUC/AUROC/Area under the curve<br>☐ C-statistic<br>☐ Harrell's c-index<br>☐ D-statistic<br>(Multiples answers are possible)
External validation<br>Which performance measures are described?	☐ AUC/AUROC/Area under the curve<br>☐ C-statistic<br>☐ Harrell's c-index<br>☐ D-statistic<br>(Multiples answers are possible)
The confidence interval (or standard error) of the AUC/AUROC/Area under the curve is presented	◯ Yes<br>◯ No
The confidence interval (or standard error) of the AUC/AUROC/Area under the curve is presented	◯ Yes<br>◯ No
What was the AUC/AUROC/Area under the curve apparent discrimination estimate (+95%CI, if given)?	______________________<br>(Please use the following format - estimate (lower CI - upper CI). For example 0.79 (0.75 - 0.81). If not reported fill this with "NR")
What was the AUC/AUROC/Area under the curve apparent discrimination estimate (+95%CI, if given)?	______________________<br>(Please use the following format - estimate (lower CI - upper CI). For example 0.79 (0.75 - 0.81). If not reported fill this with "NR")
Was the AUC/AUROC/Area under the curve corrected for optimism?	◯ Yes<br>◯ No<br>◯ Unclear
What was the optimism corrected AUC/AUROC/Area under the curve (95% CI, if given)?	______________________<br>(Please use the following format - estimate (lower CI - upper CI). For example 0.79 (0.75 - 0.81). If not reported fill this with "NR")
The confidence interval (or standard error) of the C-statistics is presented	◯ Yes<br>◯ No
The confidence interval (or standard error) of the C-statistics is presented	◯ Yes<br>◯ No
What was the C-statistics apparent discrimination estimate (+95%CI, if given)?	______________________<br>(Please use the following format - estimate (lower CI - upper CI). For example 0.79 (0.75 - 0.81). If not reported fill this with "NR")

For peer review only

What was the C-statistics apparent discrimination estimate (+95%CI, if given)?	__________________ (Please use the following format - estimate (lower CI - upper CI). For example 0.79 (0.75 - 0.81). If not reported fill this with "NR")
Was the C-statistics corrected for optimism?	◯ Yes<br>◯ No<br>◯ Unclear
What was the optimism corrected C-statistics (95% CI, if given)?	__________________ (Please use the following format - estimate (lower CI - upper CI). For example 0.79 (0.75 - 0.81). If not reported fill this with "NR")
The confidence interval (or standard error) of the Harrel's c-index is presented	◯ Yes<br>◯ No
The confidence interval (or standard error) of the Harrel's c-index is presented	◯ Yes<br>◯ No
What was the Harrel's c-index apparent discrimination estimate (+95%CI, if given)?	__________________ (Please use the following format - estimate (lower CI - upper CI). For example 0.79 (0.75 - 0.81). If not reported fill this with "NR")
What was the Harrel's c-index apparent discrimination estimate (+95%CI, if given)?	__________________ (Please use the following format - estimate (lower CI - upper CI). For example 0.79 (0.75 - 0.81). If not reported fill this with "NR")
Was the Harrel's c-index corrected for optimism?	◯ Yes<br>◯ No<br>◯ Unclear
What was the optimism corrected Harrel's c-index (95% CI, if given)?	__________________ (Please use the following format - estimate (lower CI - upper CI). For example 0.79 (0.75 - 0.81). If not reported fill this with "NR")
The confidence interval (or standard error) of the D-statistic is presented	◯ Yes<br>◯ No
The confidence interval (or standard error) of the D-statistic is presented	◯ Yes<br>◯ No
What was the D-statistic apparent discrimination estimate (+95%CI, if given)?	__________________ (Please use the following format - estimate (lower CI - upper CI). For example 0.79 (0.75 - 0.81). If not reported fill this with "NR")

For peer review only

What was the D-statistic apparent discrimination estimate (+95%CI, if given)?	______________ (Please use the following format - estimate (lower CI - upper CI). For example 0.79 (0.75 - 0.81). If not reported fill this with "NR")
Was the D-statistic corrected for optimism?	◯ Yes<br>◯ No<br>◯ Unclear
What was the optimism corrected D-statistic (95% CI, if given)?	______________ (Please use the following format - estimate (lower CI - upper CI). For example 0.79 (0.75 - 0.81). If not reported fill this with "NR")
Development<br>Measures for model calibration are described	◯ Yes<br>◯ No<br>(E.g. calibration plot, calibration slope or intercept, calibration table, Hosmer Lemeshow test, O/E ratio.)
External validation<br>Measures for model calibration are described	◯ Yes<br>◯ No<br>(E.g. calibration plot, calibration slope or intercept, calibration table, Hosmer Lemeshow test, O/E ratio.)
Development<br>How was calibration assessed?	☐ Calibration plot<br>☐ Calibration slope<br>☐ Calibration intercept<br>☐ Calibration in the large<br>☐ Calibration table<br>☐ Kappa<br>☐ Observed/expected ratio<br>☐ H-L
External validation<br>How was calibration assessed?	☐ Calibration plot<br>☐ Calibration slope<br>☐ Calibration intercept<br>☐ Calibration in the large<br>☐ Calibration table<br>☐ Kappa<br>☐ Observed/expected ratio<br>☐ H-L
What was the apparent [gen_methods_141:checked] estimate (+95% CI, if given)?	______________ (Please use the following format - estimate (lower CI - upper CI). For example 0.79 (0.75 - 0.81). List using (;) if more than 1. If not reported, fill this with "NR")
What was the apparent [gen_methods_142:checked] estimate (+95% CI, if given)?	______________ (Please use the following format - estimate (lower CI - upper CI). For example 0.79 (0.75 - 0.81). List using (;) if more than 1. If not reported, fill this with "NR")

For peer review only

Was the [gen_methods_141:checked] measure corrected for optimism?	◯ Yes<br>◯ No<br>◯ Unclear
What was the optimism corrected [gen_methods_141:checked] estimate (+95% CI, if given)?	__________________________________<br>(Please use the following format - estimate (lower CI - upper CI). For example 0.79 (0.75 - 0.81). List using (;) if more than 1. If not reported, fill this with "NR")
Development<br>Other model performance measures are presented	◯ Yes<br>◯ No<br>(E.g. R2, Brier score, predictive values, sensitivity, specificity, AUC difference, decision curve analysis, net reclassification improvement, integrated discrimination improvement, AIC.)
External validation<br>Other model performance measures are presented	◯ Yes<br>◯ No<br>(E.g. R2, Brier score, predictive values, sensitivity, specificity, AUC difference, decision curve analysis, net reclassification improvement, integrated discrimination improvement, AIC.)
Development<br>If yes, please specify	☐ R2<br>☐ Brier score<br>☐ predictive values<br>☐ sensitivity<br>☐ specificity<br>☐ AUC difference<br>☐ decision curve analysis<br>☐ net reclassification improvement<br>☐ integrated discrimination improvement<br>☐ AIC<br>☐ Accuracy<br>☐ Other<br>(Multiples answers are possible)
External validation<br>If yes, please specify	☐ R2<br>☐ Brier score<br>☐ predictive values<br>☐ sensitivity<br>☐ specificity<br>☐ AUC difference<br>☐ decision curve analysis<br>☐ net reclassification improvement<br>☐ integrated discrimination improvement<br>☐ AIC<br>☐ Accuracy<br>☐ Other<br>(Multiples answers are possible)
If other, please specify	__________________________________<br>(list using (;) to separate if more than 1.)

For peer review only

If other, please specify	______________________ (list using (;) to separate if more than 1.)
What was the apparent [gen_methods_125:checked] estimate (+95% CI, if given)?	______________________ (Please use the following format - estimate (lower CI - upper CI). For example 0.79 (0.75 - 0.81). List using (;) if more than 1. If not reported, fill this with "NR")
What was the apparent [gen_methods_126:checked] estimate (+95% CI, if given)?	______________________ (Please use the following format - estimate (lower CI - upper CI). For example 0.79 (0.75 - 0.81). List using (;) if more than 1. If not reported, fill this with "NR")
Was the [gen_methods_125:checked] measure corrected for optimism?	◯ Yes<br>◯ No<br>◯ Unclear
What was the optimism corrected [gen_methods_125:checked] estimate (+95% CI, if given)?	______________________ (Please use the following format - estimate (lower CI - upper CI). For example 0.79 (0.75 - 0.81). List using (;) if more than 1. If not reported, fill this with "NR")

Model 5

Is this the recommended model?	◯ Yes<br>◯ No<br>(If there is only one model, please state 'Yes')
Development<br>A discrimination measure is presented	◯ Yes<br>◯ No<br>(E.g. C-index / area under the ROC curve)
External validation<br>A discrimination measure is presented	◯ Yes<br>◯ No<br>(E.g. C-index / area under the ROC curve)
Development<br>Which performance measures are described?	☐ AUC/AUROC/Area under the curve<br>☐ C-statistic<br>☐ Harrell's c-index<br>☐ D-statistic<br>(Multiples answers are possible)
External validation<br>Which performance measures are described?	☐ AUC/AUROC/Area under the curve<br>☐ C-statistic<br>☐ Harrell's c-index<br>☐ D-statistic<br>(Multiples answers are possible)

For peer review only

The confidence interval (or standard error) of the AUC/AUROC/Area under the curve is presented	◯ Yes<br>◯ No
The confidence interval (or standard error) of the AUC/AUROC/Area under the curve is presented	◯ Yes<br>◯ No
What was the AUC/AUROC/Area under the curve apparent discrimination estimate (+95%CI, if given)?	__________________<br>(Please use the following format - estimate (lower CI - upper CI). For example 0.79 (0.75 - 0.81). If not reported fill this with "NR")
What was the AUC/AUROC/Area under the curve apparent discrimination estimate (+95%CI, if given)?	__________________<br>(Please use the following format - estimate (lower CI - upper CI). For example 0.79 (0.75 - 0.81). If not reported fill this with "NR")
Was the AUC/AUROC/Area under the curve corrected for optimism?	◯ Yes<br>◯ No<br>◯ Unclear
What was the optimism corrected AUC/AUROC/Area under the curve (95% CI, if given)?	__________________<br>(Please use the following format - estimate (lower CI - upper CI). For example 0.79 (0.75 - 0.81). If not reported fill this with "NR")
The confidence interval (or standard error) of the C-statistic is presented	◯ Yes<br>◯ No
The confidence interval (or standard error) of the C-statistic is presented	◯ Yes<br>◯ No
What was the C-statistic apparent discrimination estimate (+95%CI, if given)?	__________________<br>(Please use the following format - estimate (lower CI - upper CI). For example 0.79 (0.75 - 0.81). If not reported fill this with "NR")
What was the C-statistic apparent discrimination estimate (+95%CI, if given)?	__________________<br>(Please use the following format - estimate (lower CI - upper CI). For example 0.79 (0.75 - 0.81). If not reported fill this with "NR")
Was the C-statistic corrected for optimism?	◯ Yes<br>◯ No<br>◯ Unclear
What was the optimism corrected C-statistic (95% CI, if given)?	__________________<br>(Please use the following format - estimate (lower CI - upper CI). For example 0.79 (0.75 - 0.81). If not reported fill this with "NR")
The confidence interval (or standard error) of the Harrel's c-index is presented	◯ Yes<br>◯ No

For peer review only

The confidence interval (or standard error) of the Harrel's c-index is presented	◯ Yes<br>◯ No
What was the Harrel's c-index apparent discrimination estimate (+95%CI, if given)?	__________________<br>(Please use the following format - estimate (lower CI - upper CI). For example 0.79 (0.75 - 0.81). If not reported fill this with "NR")
What was the Harrel's c-index apparent discrimination estimate (+95%CI, if given)?	__________________<br>(Please use the following format - estimate (lower CI - upper CI). For example 0.79 (0.75 - 0.81). If not reported fill this with "NR")
Was the Harrel's c-index corrected for optimism?	◯ Yes<br>◯ No<br>◯ Unclear
What was the optimism corrected Harrel's c-index (95% CI, if given)?	__________________<br>(Please use the following format - estimate (lower CI - upper CI). For example 0.79 (0.75 - 0.81). If not reported fill this with "NR")
The confidence interval (or standard error) of the D-statistic is presented	◯ Yes<br>◯ No
The confidence interval (or standard error) of the D-statistic is presented	◯ Yes<br>◯ No
What was the D-statistic apparent discrimination estimate (+95%CI, if given)?	__________________<br>(Please use the following format - estimate (lower CI - upper CI). For example 0.79 (0.75 - 0.81). If not reported fill this with "NR")
What was the D-statistic apparent discrimination estimate (+95%CI, if given)?	__________________<br>(Please use the following format - estimate (lower CI - upper CI). For example 0.79 (0.75 - 0.81). If not reported fill this with "NR")
Was the D-statistic corrected for optimism?	◯ Yes<br>◯ No<br>◯ Unclear
What was the optimism corrected D-statistic (95% CI, if given)?	__________________<br>(Please use the following format - estimate (lower CI - upper CI). For example 0.79 (0.75 - 0.81). If not reported fill this with "NR")
Development<br>Measures for model calibration are described	◯ Yes<br>◯ No<br>(E.g. calibration plot, calibration slope or intercept, calibration table, Hosmer Lemeshow test, O/E ratio.)

For peer review only

External validation<br>Measures for model calibration are described	◯ Yes<br>◯ No<br>(E.g. calibration plot, calibration slope or intercept, calibration table, Hosmer Lemeshow test, O/E ratio.)
Development<br>How was calibration assessed?	☐ Calibration plot<br>☐ Calibration slope<br>☐ Calibration intercept<br>☐ Calibration in the large<br>☐ Calibration table<br>☐ Kappa<br>☐ Observed/expected ratio<br>☐ H-L
External validation<br>How was calibration assessed?	☐ Calibration plot<br>☐ Calibration slope<br>☐ Calibration intercept<br>☐ Calibration in the large<br>☐ Calibration table<br>☐ Kappa<br>☐ Observed/expected ratio<br>☐ H-L
What was the apparent [gen_methods_143:checked] estimate (+95% CI, if given)?	__________________________________<br>(Please use the following format - estimate (lower CI - upper CI). For example 0.79 (0.75 - 0.81). List using (;) if more than 1. If not reported, fill this with "NR")
What was the apparent [gen_methods_144:checked] estimate (+95% CI, if given)?	__________________________________<br>(Please use the following format - estimate (lower CI - upper CI). For example 0.79 (0.75 - 0.81). List using (;) if more than 1. If not reported, fill this with "NR")
Was the [gen_methods_143:checked] measure corrected for optimism?	◯ Yes<br>◯ No<br>◯ Unclear
What was the optimism corrected [gen_methods_143:checked] estimate (+95% CI, if given)?	__________________________________<br>(Please use the following format - estimate (lower CI - upper CI). For example 0.79 (0.75 - 0.81). List using (;) if more than 1. If not reported, fill this with "NR")
Development<br>Other model performance measures are presented	◯ Yes<br>◯ No<br>(E.g. R2, Brier score, predictive values, sensitivity, specificity, AUC difference, decision curve analysis, net reclassification improvement, integrated discrimination improvement, AIC.)

For peer review only

External validation<br>Other model performance measures are presented	◯ Yes<br>◯ No<br>(E.g. R2, Brier score, predictive values, sensitivity, specificity, AUC difference, decision curve analysis, net reclassification improvement, integrated discrimination improvement, AIC.)
Development<br>If yes, please specify	☐ R2<br>☐ Brier score<br>☐ predictive values<br>☐ sensitivity<br>☐ specificity<br>☐ AUC difference<br>☐ decision curve analysis<br>☐ net reclassification improvement<br>☐ integrated discrimination improvement<br>☐ AIC<br>☐ Accuracy<br>☐ Other<br>(Multiples answers are possible)
External validation<br>If yes, please specify	☐ R2<br>☐ Brier score<br>☐ predictive values<br>☐ sensitivity<br>☐ specificity<br>☐ AUC difference<br>☐ decision curve analysis<br>☐ net reclassification improvement<br>☐ integrated discrimination improvement<br>☐ AIC<br>☐ Accuracy<br>☐ Other<br>(Multiples answers are possible)
If other, please specify	____________________<br>(list using (;) to separate if more than 1)
If other, please specify	____________________<br>(list using (;) to separate if more than 1)
What was the apparent [gen_methods_127:checked] estimate (+95% CI, if given)?	____________________<br>(Please use the following format - estimate (lower CI - upper CI). For example 0.79 (0.75 - 0.81). List using (;) if more than 1. If not reported, fill this with "NR")
What was the apparent [gen_methods_128:checked] estimate (+95% CI, if given)?	____________________<br>(Please use the following format - estimate (lower CI - upper CI). For example 0.79 (0.75 - 0.81). List using (;) if more than 1. If not reported, fill this with "NR")

For peer review only

Was the [gen_methods_127:checked] measure corrected for optimism?	◯ Yes<br>◯ No<br>◯ Unclear
What was the optimism corrected [gen_methods_127:checked] estimate (+95% CI, if given)?	______________________________<br>(Please use the following format - estimate (lower CI - upper CI). For example 0.79 (0.75 - 0.81). List using (;) if more than 1. If not reported, fill this with "NR")

Model 6

Is this the recommended model?	◯ Yes<br>◯ No<br>(If there is only one model, please state 'Yes')
Development<br>A discrimination measure is presented	◯ Yes<br>◯ No<br>(E.g. C-index / area under the ROC curve)
External validation<br>A discrimination measure is presented	◯ Yes<br>◯ No<br>(E.g. C-index / area under the ROC curve)
Development<br>Which performance measures are described?	☐ AUC/AUROC/Area under the curve<br>☐ C-statistic<br>☐ Harrell's c-index<br>☐ D-statistic<br>(Multiples answers are possible)
External validation<br>Which performance measures are described?	☐ AUC/AUROC/Area under the curve<br>☐ C-statistic<br>☐ Harrell's c-index<br>☐ D-statistic<br>(Multiples answers are possible)
The confidence interval (or standard error) of the AUC/AUROC/Area under the curve is presented	◯ Yes<br>◯ No
The confidence interval (or standard error) of the AUC/AUROC/Area under the curve is presented	◯ Yes<br>◯ No
What was the AUC/AUROC/Area under the curve apparent discrimination estimate (+95%CI, if given)?	______________________________<br>(Please use the following format - estimate (lower CI - upper CI). For example 0.79 (0.75 - 0.81). If not reported fill this with "NR")
What was the AUC/AUROC/Area under the curve apparent discrimination estimate (+95%CI, if given)?	______________________________<br>(Please use the following format - estimate (lower CI - upper CI). For example 0.79 (0.75 - 0.81). If not reported fill this with "NR")

For peer review only

Was the AUC/AUROC/Area under the curve corrected for optimism?	◯ Yes<br>◯ No<br>◯ Unclear
What was the optimism corrected AUC/AUROC/Area under the curve (95% CI, if given)?	__________________<br>(Please use the following format - estimate (lower CI - upper CI). For example 0.79 (0.75 - 0.81). If not reported fill this with "NR")
The confidence interval (or standard error) of the C-statistic is presented	◯ Yes<br>◯ No
The confidence interval (or standard error) of the C-statistic is presented	◯ Yes<br>◯ No
What was the C-statistic apparent discrimination estimate (+95%CI, if given)?	__________________<br>(Please use the following format - estimate (lower CI - upper CI). For example 0.79 (0.75 - 0.81). If not reported fill this with "NR")
What was the C-statistic apparent discrimination estimate (+95%CI, if given)?	__________________<br>(Please use the following format - estimate (lower CI - upper CI). For example 0.79 (0.75 - 0.81). If not reported fill this with "NR")
Was the C-statistic corrected for optimism?	◯ Yes<br>◯ No<br>◯ Unclear
What was the optimism corrected C-statistic (95% CI, if given)?	__________________<br>(Please use the following format - estimate (lower CI - upper CI). For example 0.79 (0.75 - 0.81). If not reported fill this with "NR")
The confidence interval (or standard error) of the Harrel's c-index is presented	◯ Yes<br>◯ No
The confidence interval (or standard error) of the Harrel's c-index is presented	◯ Yes<br>◯ No
What was the Harrel's c-index apparent discrimination estimate (+95%CI, if given)?	__________________<br>(Please use the following format - estimate (lower CI - upper CI). For example 0.79 (0.75 - 0.81). If not reported fill this with "NR")
What was the Harrel's c-index apparent discrimination estimate (+95%CI, if given)?	__________________<br>(Please use the following format - estimate (lower CI - upper CI). For example 0.79 (0.75 - 0.81). If not reported fill this with "NR")

For peer review only

Was the Harrel's c-index corrected for optimism?	◯ Yes<br>◯ No<br>◯ Unclear
What was the optimism corrected Harrel's c-index (95% CI, if given)?	__________________________<br>(Please use the following format - estimate (lower CI - upper CI). For example 0.79 (0.75 - 0.81). If not reported fill this with "NR")
The confidence interval (or standard error) of the D-statistic is presented	◯ Yes<br>◯ No
The confidence interval (or standard error) of the D-statistic is presented	◯ Yes<br>◯ No
What was the D-statistic apparent discrimination estimate (+95%CI, if given)?	__________________________<br>(Please use the following format - estimate (lower CI - upper CI). For example 0.79 (0.75 - 0.81). If not reported fill this with "NR")
What was the D-statistic apparent discrimination estimate (+95%CI, if given)?	__________________________<br>(Please use the following format - estimate (lower CI - upper CI). For example 0.79 (0.75 - 0.81). If not reported fill this with "NR")
Was the D-statistic corrected for optimism?	◯ Yes<br>◯ No<br>◯ Unclear
What was the optimism corrected D-statistic (95% CI, if given)?	__________________________<br>(Please use the following format - estimate (lower CI - upper CI). For example 0.79 (0.75 - 0.81). If not reported fill this with "NR")
Development<br>Measures for model calibration are described	◯ Yes<br>◯ No<br>(E.g. calibration plot, calibration slope or intercept, calibration table, Hosmer Lemeshow test, O/E ratio.)
External validation<br>Measures for model calibration are described	◯ Yes<br>◯ No<br>(E.g. calibration plot, calibration slope or intercept, calibration table, Hosmer Lemeshow test, O/E ratio.)
Development<br>How was calibration assessed?	☐ Calibration plot<br>☐ Calibration slope<br>☐ Calibration intercept<br>☐ Calibration in the large<br>☐ Calibration table<br>☐ Kappa<br>☐ Observed/expected ratio<br>☐ H-L

For peer review only

External validation<br>How was calibration assessed?	☐ Calibration plot<br>☐ Calibration slope<br>☐ Calibration intercept<br>☐ Calibration in the large<br>☐ Calibration table<br>☐ Kappa<br>☐ Observed/expected ratio<br>☐ H-L
What was the apparent [gen_methods_145:checked] estimate (+95% CI, if given)?	__________________<br>(Please use the following format - estimate (lower CI - upper CI). For example 0.79 (0.75 - 0.81). List using (;) if more than 1. If not reported, fill this with "NR")
What was the apparent [gen_methods_146:checked] estimate (+95% CI, if given)?	__________________<br>(Please use the following format - estimate (lower CI - upper CI). For example 0.79 (0.75 - 0.81). List using (;) if more than 1. If not reported, fill this with "NR")
Was the [gen_methods_145:checked] measure corrected for optimism?	◯ Yes<br>◯ No<br>◯ Unclear
What was the optimism corrected [gen_methods_145:checked] estimate (+95% CI, if given)?	__________________<br>(Please use the following format - estimate (lower CI - upper CI). For example 0.79 (0.75 - 0.81). List using (;) if more than 1. If not reported, fill this with "NR")
Development<br>Other model performance measures are presented	◯ Yes<br>◯ No<br>(E.g. R2, Brier score, predictive values, sensitivity, specificity, AUC difference, decision curve analysis, net reclassification improvement, integrated discrimination improvement, AIC.)
External validation<br>Other model performance measures are presented	◯ Yes<br>◯ No<br>(E.g. R2, Brier score, predictive values, sensitivity, specificity, AUC difference, decision curve analysis, net reclassification improvement, integrated discrimination improvement, AIC.)

For peer review only

Development<br>If yes, please specify	☐ R2<br>☐ Brier score<br>☐ predictive values<br>☐ sensitivity<br>☐ specificity<br>☐ AUC difference<br>☐ decision curve analysis<br>☐ net reclassification improvement<br>☐ integrated discrimination improvement<br>☐ AIC<br>☐ Accuracy<br>☐ Other<br>(Multiples answers are possible)
External validation<br>If yes, please specify	☐ R2<br>☐ Brier score<br>☐ predictive values<br>☐ sensitivity<br>☐ specificity<br>☐ AUC difference<br>☐ decision curve analysis<br>☐ net reclassification improvement<br>☐ integrated discrimination improvement<br>☐ AIC<br>☐ Accuracy<br>☐ Other<br>(Multiples answers are possible)
If other, please specify	________________________<br>(list using (;) to separate if more than 1)
If other, please specify	________________________<br>(list using (;) to separate if more than 1)
What was the apparent [gen_methods_129:checked] estimate (+95% CI, if given)?	________________________<br>(Please use the following format - estimate (lower CI - upper CI). For example 0.79 (0.75 - 0.81). List using (;) if more than 1. If not reported, fill this with "NR")
What was the apparent [gen_methods_138:checked] estimate (+95% CI, if given)?	________________________<br>(Please use the following format - estimate (lower CI - upper CI). For example 0.79 (0.75 - 0.81). List using (;) if more than 1. If not reported, fill this with "NR")
Was the [gen_methods_129:checked] measure corrected for optimism?	◯ Yes<br>◯ No<br>◯ Unclear

For peer review only

What was the optimism corrected [gen_methods_129:checked] estimate (+95% CI, if given)?	__________________________________ (Please use the following format - estimate (lower CI - upper CI). For example 0.79 (0.75 - 0.81). List using (;) if more than 1. If not reported, fill this with "NR")

Model 7

Is this the recommended model?	◯ Yes<br>◯ No<br>(If there is only one model, please state 'Yes')
Development<br>A discrimination measure is presented	◯ Yes<br>◯ No<br>(E.g. C-index / area under the ROC curve)
External validation<br>A discrimination measure is presented	◯ Yes<br>◯ No<br>(E.g. C-index / area under the ROC curve)
Development<br>Which performance measures are described?	☐ AUC/AUROC/Area under the curve<br>☐ C-statistic<br>☐ Harrell's c-index<br>☐ D-statistic<br>(Multiples answers are possible)
External validation<br>Which performance measures are described?	☐ AUC/AUROC/Area under the curve<br>☐ C-statistic<br>☐ Harrell's c-index<br>☐ D-statistic<br>(Multiples answers are possible)
The confidence interval (or standard error) of the AUC/AUROC/Area under the curve is presented	◯ Yes<br>◯ No
The confidence interval (or standard error) of the AUC/AUROC/Area under the curve is presented	◯ Yes<br>◯ No
What was the AUC/AUROC/Area under the curve apparent discrimination estimate (+95%CI, if given)?	__________________________________<br>(Please use the following format - estimate (lower CI - upper CI). For example 0.79 (0.75 - 0.81). If not reported fill this with "NR")
What was the AUC/AUROC/Area under the curve apparent discrimination estimate (+95%CI, if given)?	__________________________________<br>(Please use the following format - estimate (lower CI - upper CI). For example 0.79 (0.75 - 0.81). If not reported fill this with "NR")
Was the AUC/AUROC/Area under the curve corrected for optimism?	◯ Yes<br>◯ No<br>◯ Unclear

For peer review only

What was the optimism corrected AUC/AUROC/Area under the curve (95% CI, if given)?	__________________ (Please use the following format - estimate (lower CI - upper CI). For example 0.79 (0.75 - 0.81). If not reported fill this with "NR")
The confidence interval (or standard error) of the C-statistic is presented	◯ Yes<br>◯ No
The confidence interval (or standard error) of the C-statistic is presented	◯ Yes<br>◯ No
What was the C-statistic apparent discrimination estimate (+95%CI, if given)?	__________________ (Please use the following format - estimate (lower CI - upper CI). For example 0.79 (0.75 - 0.81). If not reported fill this with "NR")
What was the C-statistic apparent discrimination estimate (+95%CI, if given)?	__________________ (Please use the following format - estimate (lower CI - upper CI). For example 0.79 (0.75 - 0.81). If not reported fill this with "NR")
Was the C-statistic corrected for optimism?	◯ Yes<br>◯ No<br>◯ Unclear
What was the optimism corrected C-statistic (95% CI, if given)?	__________________ (Please use the following format - estimate (lower CI - upper CI). For example 0.79 (0.75 - 0.81). If not reported fill this with "NR")
The confidence interval (or standard error) of the Harrel's c-index is presented	◯ Yes<br>◯ No
The confidence interval (or standard error) of the Harrel's c-index is presented	◯ Yes<br>◯ No
What was the Harrel's c-index apparent discrimination estimate (+95%CI, if given)?	__________________ (Please use the following format - estimate (lower CI - upper CI). For example 0.79 (0.75 - 0.81). If not reported fill this with "NR")
What was the Harrel's c-index apparent discrimination estimate (+95%CI, if given)?	__________________ (Please use the following format - estimate (lower CI - upper CI). For example 0.79 (0.75 - 0.81). If not reported fill this with "NR")
Was the Harrel's c-index corrected for optimism?	◯ Yes<br>◯ No<br>◯ Unclear

For peer review only

What was the optimism corrected Harrel's c-index (95% CI, if given)?	__________ (Please use the following format - estimate (lower CI - upper CI). For example 0.79 (0.75 - 0.81). If not reported fill this with "NR")
The confidence interval (or standard error) of the D-statistic is presented	◯ Yes<br>◯ No
The confidence interval (or standard error) of the D-statistic is presented	◯ Yes<br>◯ No
What was the D-statistic apparent discrimination estimate (+95%CI, if given)?	__________ (Please use the following format - estimate (lower CI - upper CI). For example 0.79 (0.75 - 0.81). If not reported fill this with "NR")
What was the D-statistic apparent discrimination estimate (+95%CI, if given)?	__________ (Please use the following format - estimate (lower CI - upper CI). For example 0.79 (0.75 - 0.81). If not reported fill this with "NR")
Was the D-statistic corrected for optimism?	◯ Yes<br>◯ No<br>◯ Unclear
What was the optimism corrected D-statistic (95% CI, if given)?	__________ (Please use the following format - estimate (lower CI - upper CI). For example 0.79 (0.75 - 0.81). If not reported fill this with "NR")
Development<br>Measures for model calibration are described	◯ Yes<br>◯ No<br>(E.g. calibration plot, calibration slope or intercept, calibration table, Hosmer Lemeshow test, O/E ratio.)
External validation<br>Measures for model calibration are described	◯ Yes<br>◯ No<br>(E.g. calibration plot, calibration slope or intercept, calibration table, Hosmer Lemeshow test, O/E ratio.)
Development<br>How was calibration assessed?	☐ Calibration plot<br>☐ Calibration slope<br>☐ Calibration intercept<br>☐ Calibration in the large<br>☐ Calibration table<br>☐ Kappa<br>☐ Observed/expected ratio<br>☐ H-L

For peer review only

External validation<br>How was calibration assessed?	☐ Calibration plot<br>☐ Calibration slope<br>☐ Calibration intercept<br>☐ Calibration in the large<br>☐ Calibration table<br>☐ Kappa<br>☐ Observed/expected ratio<br>☐ H-L
What was the apparent [gen_methods_147:checked] estimate (+95% CI, if given)?	__________________________________<br>(Please use the following format - estimate (lower CI - upper CI). For example 0.79 (0.75 - 0.81). List using (;) if more than 1. If not reported, fill this with "NR")
What was the apparent [gen_methods_148:checked] estimate (+95% CI, if given)?	__________________________________<br>(Please use the following format - estimate (lower CI - upper CI). For example 0.79 (0.75 - 0.81). List using (;) if more than 1. If not reported, fill this with "NR")
Was the [gen_methods_147:checked] measure corrected for optimism?	◯ Yes<br>◯ No<br>◯ Unclear
What was the optimism corrected [gen_methods_147:checked] estimate (+95% CI, if given)?	__________________________________<br>(Please use the following format - estimate (lower CI - upper CI). For example 0.79 (0.75 - 0.81). List using (;) if more than 1. If not reported, fill this with "NR")
Development<br>Other model performance measures are presented	◯ Yes<br>◯ No<br>(E.g. R2, Brier score, predictive values, sensitivity, specificity, AUC difference, decision curve analysis, net reclassification improvement, integrated discrimination improvement, AIC.)
External validation<br>Other model performance measures are presented	◯ Yes<br>◯ No<br>(E.g. R2, Brier score, predictive values, sensitivity, specificity, AUC difference, decision curve analysis, net reclassification improvement, integrated discrimination improvement, AIC.)

For peer review only

Development<br>If yes, please specify	☐ R2<br>☐ Brier score<br>☐ predictive values<br>☐ sensitivity<br>☐ specificity<br>☐ AUC difference<br>☐ decision curve analysis<br>☐ net reclassification improvement<br>☐ integrated discrimination improvement<br>☐ AIC<br>☐ Accuracy<br>☐ Other<br>(Multiples answers are possible)
External validation<br>If yes, please specify	☐ R2<br>☐ Brier score<br>☐ predictive values<br>☐ sensitivity<br>☐ specificity<br>☐ AUC difference<br>☐ decision curve analysis<br>☐ net reclassification improvement<br>☐ integrated discrimination improvement<br>☐ AIC<br>☐ Accuracy<br>☐ Other<br>(Multiples answers are possible)
If other, please specify	______________________<br>(list using (;) to separate if more than 1.)
If other, please specify	______________________<br>(list using (;) to separate if more than 1.)
What was the apparent [gen_methods_130:checked] estimate (+95% CI, if given)?	______________________<br>(Please use the following format - estimate (lower CI - upper CI). For example 0.79 (0.75 - 0.81). List using (;) if more than 1. If not reported, fill this with "NR")
What was the apparent [gen_methods_137:checked] estimate (+95% CI, if given)?	______________________<br>(Please use the following format - estimate (lower CI - upper CI). For example 0.79 (0.75 - 0.81). List using (;) if more than 1. If not reported, fill this with "NR")
Was the [gen_methods_130:checked] measure corrected for optimism?	◯ Yes<br>◯ No<br>◯ Unclear

For peer review only

What was the optimism corrected [gen_methods_130:checked] estimate (+95% CI, if given)?

____
(Please use the following format - estimate (lower CI - upper CI). For example 0.79 (0.75 - 0.81). List using (;) if more than 1. If not reported, fill this with "NR")

Model 8

Is this the recommended model?

◯ Yes
◯ No
(If there is only one model, please state 'Yes')

Development
A discrimination measure is presented

◯ Yes
◯ No
(E.g. C-index / area under the ROC curve)

External validation
A discrimination measure is presented

◯ Yes
◯ No
(E.g. C-index / area under the ROC curve)

Development
Which performance measures are described?

☐ AUC/AUROC/Area under the curve
☐ C-statistic
☐ Harrell's c-index
☐ D-statistic
(Multiples answers are possible)

External validation
Which performance measures are described?

☐ AUC/AUROC/Area under the curve
☐ C-statistic
☐ Harrell's c-index
☐ D-statistic
(Multiples answers are possible)

The confidence interval (or standard error) of the AUC/AUROC/Area under the curve is presented

◯ Yes
◯ No

The confidence interval (or standard error) of the AUC/AUROC/Area under the curve is presented

◯ Yes
◯ No

What was the AUC/AUROC/Area under the curve apparent discrimination estimate (+95%CI, if given)?

____
(Please use the following format - estimate (lower CI - upper CI). For example 0.79 (0.75 - 0.81). If not reported fill this with "NR")

What was the AUC/AUROC/Area under the curve apparent discrimination estimate (+95%CI, if given)?

____
(Please use the following format - estimate (lower CI - upper CI). For example 0.79 (0.75 - 0.81). If not reported fill this with "NR")

Was the AUC/AUROC/Area under the curve corrected for optimism?

◯ Yes
◯ No
◯ Unclear

For peer review only

What was the optimism corrected AUC/AUROC/Area under the curve (95% CI, if given)?	__________________ (Please use the following format - estimate (lower CI - upper CI). For example 0.79 (0.75 - 0.81). If not reported fill this with "NR")
The confidence interval (or standard error) of the C-statistic is presented	◯ Yes ◯ No
The confidence interval (or standard error) of the C-statistic is presented	◯ Yes ◯ No
What was the C-statistic apparent discrimination estimate (+95%CI, if given)?	__________________ (Please use the following format - estimate (lower CI - upper CI). For example 0.79 (0.75 - 0.81). If not reported fill this with "NR")
What was the C-statistic apparent discrimination estimate (+95%CI, if given)?	__________________ (Please use the following format - estimate (lower CI - upper CI). For example 0.79 (0.75 - 0.81). If not reported fill this with "NR")
Was the C-statistic corrected for optimism?	◯ Yes ◯ No ◯ Unclear
What was the optimism corrected C-statistic (95% CI, if given)?	__________________ (Please use the following format - estimate (lower CI - upper CI). For example 0.79 (0.75 - 0.81). If not reported fill this with "NR")
The confidence interval (or standard error) of the Harrel's c-index is presented	◯ Yes ◯ No
The confidence interval (or standard error) of the Harrel's c-index is presented	◯ Yes ◯ No
What was the Harrel's c-index apparent discrimination estimate (+95%CI, if given)?	__________________ (Please use the following format - estimate (lower CI - upper CI). For example 0.79 (0.75 - 0.81). If not reported fill this with "NR")
What was the Harrel's c-index apparent discrimination estimate (+95%CI, if given)?	__________________ (Please use the following format - estimate (lower CI - upper CI). For example 0.79 (0.75 - 0.81). If not reported fill this with "NR")
Was the Harrel's c-index corrected for optimism?	◯ Yes ◯ No ◯ Unclear

For peer review only

Was the Harrel's c-index corrected for optimism?	◯ Yes<br>◯ No<br>◯ Unclear
What was the optimism corrected Harrel's c-index (95% CI, if given)?	__________________<br>(Please use the following format - estimate (lower CI - upper CI). For example 0.79 (0.75 - 0.81). If not reported fill this with "NR")
What was the optimism corrected Harrel's c-index (95% CI, if given)?	__________________<br>(Please use the following format - estimate (lower CI - upper CI). For example 0.79 (0.75 - 0.81). If not reported fill this with "NR")
The confidence interval (or standard error) of the D-statistic is presented	◯ Yes<br>◯ No
The confidence interval (or standard error) of the D-statistic is presented	◯ Yes<br>◯ No
What was the D-statistic apparent discrimination estimate (+95%CI, if given)?	__________________<br>(Please use the following format - estimate (lower CI - upper CI). For example 0.79 (0.75 - 0.81). If not reported fill this with "NR")
What was the D-statistic apparent discrimination estimate (+95%CI, if given)?	__________________<br>(Please use the following format - estimate (lower CI - upper CI). For example 0.79 (0.75 - 0.81). If not reported fill this with "NR")
Was the D-statistic corrected for optimism?	◯ Yes<br>◯ No<br>◯ Unclear
What was the optimism corrected D-statistic (95% CI, if given)?	__________________<br>(Please use the following format - estimate (lower CI - upper CI). For example 0.79 (0.75 - 0.81). If not reported fill this with "NR")
Development<br>Measures for model calibration are described	◯ Yes<br>◯ No<br>(E.g. calibration plot, calibration slope or intercept, calibration table, Hosmer Lemeshow test, O/E ratio.)
External validation<br>Measures for model calibration are described	◯ Yes<br>◯ No<br>(E.g. calibration plot, calibration slope or intercept, calibration table, Hosmer Lemeshow test, O/E ratio.)

For peer review only

Development<br>How was calibration assessed?	☐ Calibration plot<br>☐ Calibration slope<br>☐ Calibration intercept<br>☐ Calibration in the large<br>☐ Calibration table<br>☐ Kappa<br>☐ Observed/expected ratio<br>☐ H-L
External validation<br>How was calibration assessed?	☐ Calibration plot<br>☐ Calibration slope<br>☐ Calibration intercept<br>☐ Calibration in the large<br>☐ Calibration table<br>☐ Kappa<br>☐ Observed/expected ratio<br>☐ H-L
What was the apparent [gen_methods_149:checked] estimate (+95% CI, if given)?	______________________<br>(Please use the following format - estimate (lower CI - upper CI). For example 0.79 (0.75 - 0.81). List using (;) if more than 1. If not reported, fill this with "NR")
What was the apparent [gen_methods_150:checked] estimate (+95% CI, if given)?	______________________<br>(Please use the following format - estimate (lower CI - upper CI). For example 0.79 (0.75 - 0.81). List using (;) if more than 1. If not reported, fill this with "NR")
Was the [gen_methods_149:checked] measure corrected for optimism?	◯ Yes<br>◯ No<br>◯ Unclear
What was the optimism corrected [gen_methods_149:checked] estimate (+95% CI, if given)?	______________________<br>(Please use the following format - estimate (lower CI - upper CI). For example 0.79 (0.75 - 0.81). List using (;) if more than 1. If not reported, fill this with "NR")
Development<br>Other model performance measures are presented	◯ Yes<br>◯ No<br>(E.g. R2, Brier score, predictive values, sensitivity, specificity, AUC difference, decision curve analysis, net reclassification improvement, integrated discrimination improvement, AIC.)
External validation<br>Other model performance measures are presented	◯ Yes<br>◯ No<br>(E.g. R2, Brier score, predictive values, sensitivity, specificity, AUC difference, decision curve analysis, net reclassification improvement, integrated discrimination improvement, AIC.)

For peer review only

Development<br>If yes, please specify	☐ R2<br>☐ Brier score<br>☐ predictive values<br>☐ sensitivity<br>☐ specificity<br>☐ AUC difference<br>☐ decision curve analysis<br>☐ net reclassification improvement<br>☐ integrated discrimination improvement<br>☐ AIC<br>☐ Accuracy<br>☐ Other<br>(Multiples answers are possible)
External validation<br>If yes, please specify	☐ R2<br>☐ Brier score<br>☐ predictive values<br>☐ sensitivity<br>☐ specificity<br>☐ AUC difference<br>☐ decision curve analysis<br>☐ net reclassification improvement<br>☐ integrated discrimination improvement<br>☐ AIC<br>☐ Accuracy<br>☐ Other<br>(Multiples answers are possible)
If other, please specify	__________________________________<br>(list using (;) to separate if more than 1.)
If other, please specify	__________________________________<br>(list using (;) to separate if more than 1.)
What was the apparent [gen_methods_131:checked] estimate (+95% CI, if given)?	__________________________________<br>(Please use the following format - estimate (lower CI - upper CI). For example 0.79 (0.75 - 0.81). List using (;) if more than 1. If not reported, fill this with "NR")
What was the apparent [gen_methods_136:checked] estimate (+95% CI, if given)?	__________________________________<br>(Please use the following format - estimate (lower CI - upper CI). For example 0.79 (0.75 - 0.81). List using (;) if more than 1. If not reported, fill this with "NR")
Was the [gen_methods_131:checked] measure corrected for optimism?	◯ Yes<br>◯ No<br>◯ Unclear

For peer review only

What was the optimism corrected [gen_methods_131:checked] estimate (+95% CI, if given)?	______________________ (Please use the following format - estimate (lower CI - upper CI). For example 0.79 (0.75 - 0.81). List using (;) if more than 1. If not reported, fill this with "NR")

Model 9

Is this the recommended model?	◯ Yes<br>◯ No<br>(If there is only one model, please state 'Yes')
Development<br>A discrimination measure is presented	◯ Yes<br>◯ No<br>(E.g. C-index / area under the ROC curve)
External validation<br>A discrimination measure is presented	◯ Yes<br>◯ No<br>(E.g. C-index / area under the ROC curve)
Development<br>Which performance measures are described?	☐ AUC/AUROC/Area under the curve<br>☐ C-statistic<br>☐ Harrell's c-index<br>☐ D-statistic<br>(Multiples answers are possible)
External validation<br>Which performance measures are described?	☐ AUC/AUROC/Area under the curve<br>☐ C-statistic<br>☐ Harrell's c-index<br>☐ D-statistic<br>(Multiples answers are possible)
The confidence interval (or standard error) of the AUC/AUROC/Area under the curve is presented	◯ Yes<br>◯ No
The confidence interval (or standard error) of the AUC/AUROC/Area under the curve is presented	◯ Yes<br>◯ No
What was the AUC/AUROC/Area under the curve apparent discrimination estimate (+95%CI, if given)?	______________________ (Please use the following format - estimate (lower CI - upper CI). For example 0.79 (0.75 - 0.81). If not reported fill this with "NR")
What was the AUC/AUROC/Area under the curve apparent discrimination estimate (+95%CI, if given)?	______________________ (Please use the following format - estimate (lower CI - upper CI). For example 0.79 (0.75 - 0.81). If not reported fill this with "NR")
Was the AUC/AUROC/Area under the curve corrected for optimism?	◯ Yes<br>◯ No<br>◯ Unclear

For peer review only

What was the optimism corrected AUC/AUROC/Area under the curve (95% CI, if given)?	__________________ (Please use the following format - estimate (lower CI - upper CI). For example 0.79 (0.75 - 0.81). If not reported fill this with "NR")
The confidence interval (or standard error) of the C-statistic is presented	◯ Yes<br>◯ No
The confidence interval (or standard error) of the C-statistic is presented	◯ Yes<br>◯ No
What was the C-statistic apparent discrimination estimate (+95%CI, if given)?	__________________ (Please use the following format - estimate (lower CI - upper CI). For example 0.79 (0.75 - 0.81). If not reported fill this with "NR")
What was the C-statistic apparent discrimination estimate (+95%CI, if given)?	__________________ (Please use the following format - estimate (lower CI - upper CI). For example 0.79 (0.75 - 0.81). If not reported fill this with "NR")
Was the C-statistic corrected for optimism?	◯ Yes<br>◯ No<br>◯ Unclear
What was the optimism corrected C-statistic (95% CI, if given)?	__________________ (Please use the following format - estimate (lower CI - upper CI). For example 0.79 (0.75 - 0.81). If not reported fill this with "NR")
The confidence interval (or standard error) of the Harrel's c-index is presented	◯ Yes<br>◯ No
The confidence interval (or standard error) of the Harrel's c-index is presented	◯ Yes<br>◯ No
What was the Harrel's c-index apparent discrimination estimate (+95%CI, if given)?	__________________ (Please use the following format - estimate (lower CI - upper CI). For example 0.79 (0.75 - 0.81). If not reported fill this with "NR")
What was the Harrel's c-index apparent discrimination estimate (+95%CI, if given)?	__________________ (Please use the following format - estimate (lower CI - upper CI). For example 0.79 (0.75 - 0.81). If not reported fill this with "NR")
Was the Harrel's c-index corrected for optimism?	◯ Yes<br>◯ No<br>◯ Unclear

For peer review only

What was the optimism corrected Harrel's c-index (95% CI, if given)?	__________________ (Please use the following format - estimate (lower CI - upper CI). For example 0.79 (0.75 - 0.81). If not reported fill this with "NR")
The confidence interval (or standard error) of the D-statistic is presented	◯ Yes<br>◯ No
The confidence interval (or standard error) of the D-statistic is presented	◯ Yes<br>◯ No
What was the D-statistic apparent discrimination estimate (+95%CI, if given)?	__________________ (Please use the following format - estimate (lower CI - upper CI). For example 0.79 (0.75 - 0.81). If not reported fill this with "NR")
What was the D-statistic apparent discrimination estimate (+95%CI, if given)?	__________________ (Please use the following format - estimate (lower CI - upper CI). For example 0.79 (0.75 - 0.81). If not reported fill this with "NR")
Was the D-statistic corrected for optimism?	◯ Yes<br>◯ No<br>◯ Unclear
What was the optimism corrected D-statistic (95% CI, if given)?	__________________ (Please use the following format - estimate (lower CI - upper CI). For example 0.79 (0.75 - 0.81). If not reported fill this with "NR")
Development<br>Measures for model calibration are described	◯ Yes<br>◯ No<br>(E.g. calibration plot, calibration slope or intercept, calibration table, Hosmer Lemeshow test, O/E ratio.)
External validation<br>Measures for model calibration are described	◯ Yes<br>◯ No<br>(E.g. calibration plot, calibration slope or intercept, calibration table, Hosmer Lemeshow test, O/E ratio.)
Development<br>How was calibration assessed?	☐ Calibration plot<br>☐ Calibration slope<br>☐ Calibration intercept<br>☐ Calibration in the large<br>☐ Calibration table<br>☐ Kappa<br>☐ Observed/expected ratio<br>☐ H-L

For peer review only

External validation<br>How was calibration assessed?	☐ Calibration plot<br>☐ Calibration slope<br>☐ Calibration intercept<br>☐ Calibration in the large<br>☐ Calibration table<br>☐ Kappa<br>☐ Observed/expected ratio<br>☐ H-L
What was the apparent [gen_methods_155:checked] estimate (+95% CI, if given)?	__________________<br>(Please use the following format - estimate (lower CI - upper CI). For example 0.79 (0.75 - 0.81). List using (;) if more than 1. If not reported, fill this with "NR")
What was the apparent [gen_methods_166:checked] estimate (+95% CI, if given)?	__________________<br>(Please use the following format - estimate (lower CI - upper CI). For example 0.79 (0.75 - 0.81). List using (;) if more than 1. If not reported, fill this with "NR")
Was the [gen_methods_155:checked] measure corrected for optimism?	◯ Yes<br>◯ No<br>◯ Unclear
What was the optimism corrected [gen_methods_155:checked] estimate (+95% CI, if given)?	__________________<br>(Please use the following format - estimate (lower CI - upper CI). For example 0.79 (0.75 - 0.81). List using (;) if more than 1. If not reported, fill this with "NR")
Development<br>Other model performance measures are presented	◯ Yes<br>◯ No<br>(E.g. R2, Brier score, predictive values, sensitivity, specificity, AUC difference, decision curve analysis, net reclassification improvement, integrated discrimination improvement, AIC.)
External validation<br>Other model performance measures are presented	◯ Yes<br>◯ No<br>(E.g. R2, Brier score, predictive values, sensitivity, specificity, AUC difference, decision curve analysis, net reclassification improvement, integrated discrimination improvement, AIC.)

For peer review only

Development<br>If yes, please specify	☐ R2<br>☐ Brier score<br>☐ predictive values<br>☐ sensitivity<br>☐ specificity<br>☐ AUC difference<br>☐ decision curve analysis<br>☐ net reclassification improvement<br>☐ integrated discrimination improvement<br>☐ AIC<br>☐ Accuracy<br>☐ Other<br>(Multiples answers are possible)
External validation<br>If yes, please specify	☐ R2<br>☐ Brier score<br>☐ predictive values<br>☐ sensitivity<br>☐ specificity<br>☐ AUC difference<br>☐ decision curve analysis<br>☐ net reclassification improvement<br>☐ integrated discrimination improvement<br>☐ AIC<br>☐ Accuracy<br>☐ Other<br>(Multiples answers are possible)
If other, please specify	__________________________________<br>(list using (;) to separate if more than 1.)
If other, please specify	__________________________________<br>(list using (;) to separate if more than 1.)
What was the apparent [gen_methods_132:checked] estimate (+95% CI, if given)?	__________________________________<br>(Please use the following format - estimate (lower CI - upper CI). For example 0.79 (0.75 - 0.81). List using (;) if more than 1. If not reported, fill this with "NR")
What was the apparent [gen_methods_135:checked] estimate (+95% CI, if given)?	__________________________________<br>(Please use the following format - estimate (lower CI - upper CI). For example 0.79 (0.75 - 0.81). List using (;) if more than 1. If not reported, fill this with "NR")
Was the [gen_methods_132:checked] measure corrected for optimism?	◯ Yes<br>◯ No<br>◯ Unclear

For peer review only

What was the optimism corrected [gen_methods_132:checked] estimate (+95% CI, if given)?	______________________ (Please use the following format - estimate (lower CI - upper CI). For example 0.79 (0.75 - 0.81). List using (;) if more than 1. If not reported, fill this with "NR")

Model 10

Is this the recommended model?	◯ Yes<br>◯ No<br>(If there is only one model, please state 'Yes')
Development<br>A discrimination measure is presented	◯ Yes<br>◯ No<br>(E.g. C-index / area under the ROC curve)
External validation<br>A discrimination measure is presented	◯ Yes<br>◯ No<br>(E.g. C-index / area under the ROC curve)
Development<br>Which performance measures are described?	☐ AUC/AUROC/Area under the curve<br>☐ C-statistic<br>☐ Harrell's c-index<br>☐ D-statistic<br>(Multiples answers are possible)
External validation<br>Which performance measures are described?	☐ AUC/AUROC/Area under the curve<br>☐ C-statistic<br>☐ Harrell's c-index<br>☐ D-statistic<br>(Multiples answers are possible)
The confidence interval (or standard error) of the AUC/AUROC/Area under the curve is presented	◯ Yes<br>◯ No
The confidence interval (or standard error) of the AUC/AUROC/Area under the curve is presented	◯ Yes<br>◯ No
What was the AUC/AUROC/Area under the curve apparent discrimination estimate (+95%CI, if given)?	______________________<br>(Please use the following format - estimate (lower CI - upper CI). For example 0.79 (0.75 - 0.81). If not reported fill this with "NR")
What was the AUC/AUROC/Area under the curve apparent discrimination estimate (+95%CI, if given)?	______________________<br>(Please use the following format - estimate (lower CI - upper CI). For example 0.79 (0.75 - 0.81). If not reported fill this with "NR")
Was the AUC/AUROC/Area under the curve corrected for optimism?	◯ Yes<br>◯ No<br>◯ Unclear

For peer review only

What was the optimism corrected AUC/AUROC/Area under the curve (95% CI, if given)?	__________________ (Please use the following format - estimate (lower CI - upper CI). For example 0.79 (0.75 - 0.81). If not reported fill this with "NR")
The confidence interval (or standard error) of the C-statistic is presented	◯ Yes<br>◯ No
The confidence interval (or standard error) of the C-statistic is presented	◯ Yes<br>◯ No
What was the C-statistic apparent discrimination estimate (+95%CI, if given)?	__________________ (Please use the following format - estimate (lower CI - upper CI). For example 0.79 (0.75 - 0.81). If not reported fill this with "NR")
What was the C-statistic apparent discrimination estimate (+95%CI, if given)?	__________________ (Please use the following format - estimate (lower CI - upper CI). For example 0.79 (0.75 - 0.81). If not reported fill this with "NR")
Was the C-statistic corrected for optimism?	◯ Yes<br>◯ No<br>◯ Unclear
What was the optimism corrected C-statistic (95% CI, if given)?	__________________ (Please use the following format - estimate (lower CI - upper CI). For example 0.79 (0.75 - 0.81). If not reported fill this with "NR")
The confidence interval (or standard error) of the Harrel's c-index is presented	◯ Yes<br>◯ No
The confidence interval (or standard error) of the Harrel's c-index is presented	◯ Yes<br>◯ No
What was the Harrel's c-index apparent discrimination estimate (+95%CI, if given)?	__________________ (Please use the following format - estimate (lower CI - upper CI). For example 0.79 (0.75 - 0.81). If not reported fill this with "NR")
What was the Harrel's c-index apparent discrimination estimate (+95%CI, if given)?	__________________ (Please use the following format - estimate (lower CI - upper CI). For example 0.79 (0.75 - 0.81). If not reported fill this with "NR")
Was the Harrel's c-index corrected for optimism?	◯ Yes<br>◯ No<br>◯ Unclear

For peer review only

What was the optimism corrected Harrel's c-index (95% CI, if given)?	__________ (Please use the following format - estimate (lower CI - upper CI). For example 0.79 (0.75 - 0.81). If not reported fill this with "NR")
The confidence interval (or standard error) of the D-statistic is presented	◯ Yes<br>◯ No
The confidence interval (or standard error) of the D-statistic is presented	◯ Yes<br>◯ No
What was the D-statistic apparent discrimination estimate (+95%CI, if given)?	__________ (Please use the following format - estimate (lower CI - upper CI). For example 0.79 (0.75 - 0.81). If not reported fill this with "NR")
What was the D-statistic apparent discrimination estimate (+95%CI, if given)?	__________ (Please use the following format - estimate (lower CI - upper CI). For example 0.79 (0.75 - 0.81). If not reported fill this with "NR")
Was the D-statistic corrected for optimism?	◯ Yes<br>◯ No<br>◯ Unclear
What was the optimism corrected D-statistic (95% CI, if given)?	__________ (Please use the following format - estimate (lower CI - upper CI). For example 0.79 (0.75 - 0.81). If not reported fill this with "NR")
Development<br>Measures for model calibration are described	◯ Yes<br>◯ No<br>(E.g. calibration plot, calibration slope or intercept, calibration table, Hosmer Lemeshow test, O/E ratio.)
External validation<br>Measures for model calibration are described	◯ Yes<br>◯ No<br>(E.g. calibration plot, calibration slope or intercept, calibration table, Hosmer Lemeshow test, O/E ratio.)
Development<br>How was calibration assessed?	☐ Calibration plot<br>☐ Calibration slope<br>☐ Calibration intercept<br>☐ Calibration in the large<br>☐ Calibration table<br>☐ Kappa<br>☐ Observed/expected ratio<br>☐ H-L

For peer review only

External validation<br>How was calibration assessed?	☐ Calibration plot<br>☐ Calibration slope<br>☐ Calibration intercept<br>☐ Calibration in the large<br>☐ Calibration table<br>☐ Kappa<br>☐ Observed/expected ratio<br>☐ H-L
What was the apparent [gen_methods_167:checked] estimate (+95% CI, if given)?	__________________<br>(Please use the following format - estimate (lower CI - upper CI). For example 0.79 (0.75 - 0.81). List using (;) if more than 1. If not reported, fill this with "NR")
What was the apparent [gen_methods_168:checked] estimate (+95% CI, if given)?	__________________<br>(Please use the following format - estimate (lower CI - upper CI). For example 0.79 (0.75 - 0.81). List using (;) if more than 1. If not reported, fill this with "NR")
Was the [gen_methods_167:checked] measure corrected for optimism?	◯ Yes<br>◯ No<br>◯ Unclear
What was the optimism corrected [gen_methods_167:checked] estimate (+95% CI, if given)?	__________________<br>(Please use the following format - estimate (lower CI - upper CI). For example 0.79 (0.75 - 0.81). List using (;) if more than 1. If not reported, fill this with "NR")
Development<br>Other model performance measures are presented	◯ Yes<br>◯ No<br>(E.g. R2, Brier score, predictive values, sensitivity, specificity, AUC difference, decision curve analysis, net reclassification improvement, integrated discrimination improvement, AIC.)
External validation<br>Other model performance measures are presented	◯ Yes<br>◯ No<br>(E.g. R2, Brier score, predictive values, sensitivity, specificity, AUC difference, decision curve analysis, net reclassification improvement, integrated discrimination improvement, AIC.)

For peer review only

Development<br>If yes, please specify	☐ R2<br>☐ Brier score<br>☐ predictive values<br>☐ sensitivity<br>☐ specificity<br>☐ AUC difference<br>☐ decision curve analysis<br>☐ net reclassification improvement<br>☐ integrated discrimination improvement<br>☐ AIC<br>☐ Accuracy<br>☐ Other<br>(Multiples answers are possible)
External validation<br>If yes, please specify	☐ R2<br>☐ Brier score<br>☐ predictive values<br>☐ sensitivity<br>☐ specificity<br>☐ AUC difference<br>☐ decision curve analysis<br>☐ net reclassification improvement<br>☐ integrated discrimination improvement<br>☐ AIC<br>☐ Accuracy<br>☐ Other<br>(Multiples answers are possible)
If other, please specify	______________________<br>(list using (;) to separate if more than 1.)
If other, please specify	______________________<br>(list using (;) to separate if more than 1.)
What was the apparent [gen_methods_133:checked] estimate (+95% CI, if given)?	______________________<br>(Please use the following format - estimate (lower CI - upper CI). For example 0.79 (0.75 - 0.81). List using (;) if more than 1. If not reported, fill this with "NR")
What was the apparent [gen_methods_134:checked] estimate (+95% CI, if given)?	______________________<br>(Please use the following format - estimate (lower CI - upper CI). For example 0.79 (0.75 - 0.81). List using (;) if more than 1. If not reported, fill this with "NR")
Was the [gen_methods_133:checked] measure corrected for optimism?	◯ Yes<br>◯ No<br>◯ Unclear

For peer review only - http://bmjopen.bmj.com/site/about/guidelines.xhtml

What was the optimism corrected [gen_methods_133:checked] estimate (+95% CI, if given)?

____
(Please use the following format - estimate (lower CI - upper CI). For example 0.79 (0.75 - 0.81). List using (;) if more than 1. If not reported, fill this with "NR")

SPIN

Is there use of leading words/strong statement in the results section to describe model and/or model performance/accuracy/effectiveness?

◯ Yes
◯ No
(The prediction estimate is described with a value judgement like "statistically significant", "significant")

If yes, please specify the leading word/strong statement

☐ Novel
☐ Excellent
☐ Accurate
☐ Optimal
☐ Perfect
☐ Significant
☐ Other

If other, please specify

____
(list using (;) to separate if more than 1)

If yes, please copy the statement below

____

Is at least ONE non-significant/non relevant model reported?

◯ Yes
◯ No
◯ NA
(Select NA when only 1 model was developed)

If yes, did the authors make use of leading word to reject those non predictive models reported?

◯ Yes
◯ No
(E.g. The effect is said to be significant, although the 95% confidence interval of the adjusted odds ratio crosses 1; OR Words like "trend" or "borderline, "significance", "statistically significant" are used)

If yes, please copy the statement below

____

Is the prediction model defined in multiples ways?

◯ Yes
◯ No
(E.g. Different thresholds of categorization AND continuous, or absolute value and relative value)

For peer review only - http://bmjopen.bmj.com/site/about/guidelines.xhtml

Presence of spin in the presentation of tables and figures	◯ Yes<br>◯ No<br>(E.g. Non-significant p values adjusted for multiple comparisons are written below the table, whereas significant unadjusted p values are highly visible)

Model Updating

The updated regression coefficients for each predictor in the model are reported	◯ Yes<br>◯ No<br>◯ NA<br>(If model updating was described as 'not needed', score Yes)
The updated intercept or cumulative baseline hazard or baseline survival (for at least one time point) is reported	◯ Yes<br>◯ No<br>◯ NA<br>(If model updating was described as 'not needed', score Yes)
The discrimination of the updated model is reported	◯ Yes<br>◯ No
The confidence interval (or standard error) of the discrimination measure of the updated model is reported	◯ Yes<br>◯ No
The calibration of the updated model is reported	◯ Yes<br>◯ No
The confidence interval (or standard error) of the calibration measure of the updated model is reported	◯ Yes<br>◯ No

Comments

Any additional comment about the results section of this article?	______________________________<br>(If there is something in the "Results" section that does not fit into the questions of this form - please use this space to detail. Also use this space to detail anything you are unsure about.)

For peer review only

Discussion / Conclusion

Article ID ______________________

Limitation

Limitations of the study are discussed
- ◯ Yes
- ◯ No

(Discuss any limitations of the study is sufficient (such as non-representative sample, few events per predictor, missing data).)

Interpretation

Comparison of results to reported performance in previous development studies and/or other validation studies is given
- ◯ Yes
- ◯ No

If yes, is the comparison in favor of similar prediction models?
- ◯ Yes
- ◯ No
- ◯ Some outcomes in favour and not in favour for others
- ◯ Unclear

Overall Interpretation

An overall interpretation of the results is given
- ◯ Yes
- ◯ No

(Considering objectives, limitations, results from similar studies, and other relevant evidence.)

Is there emphasis on model relevance while there is not enough information given to concluded results are predictive?
- ◯ Yes
- ◯ No

Is there use of leading words/strong statement in the discussion section to describe model and/or model performance/accuracy/effectiveness?
- ◯ Yes
- ◯ No

If yes, which leading word/strong statement?
- ☐ Novel
- ☐ Excellent
- ☐ Accurate
- ☐ Optimal
- ☐ Perfect
- ☐ Significant
- ☐ Other

(Multiples answers are possible)

If other, please specify ______________________

(list using (;) to separate if more than 1)

If yes, plese copy the statement below ______________________

For peer review only

Is at least (1) non-significant/non relevant model discussed?	◯ Yes<br>◯ No<br>◯ NA<br>(Select NA when only 1 model was developed)

Implications

The potential clinical use is discussed	◯ Yes<br>◯ No<br>(An explicit description of the context in which the prediction model is to be used (e.g. to identify high risk groups to help direct treatment, or to triage patients for referral to subsequent care).)
Does the recommendation include using the model in a difference clinical setting/population?	◯ Yes<br>◯ No
If yes, please provide the statement below	__________________________________
Implications for future research are discussed	◯ Yes<br>◯ No<br>(E.g. a description of what the next stage of investigation of the prediction model should be, such as "We suggest further external validation".)
If yes, please provide the statement below	__________________________________
Were there any other perspectives addressed in the discussion section?	◯ Yes<br>◯ No<br>(E.g. unexpected finding from the analyses.)
If yes, please provide the statement below or a short description	__________________________________
Is uncertainty reported in the discussion?	◯ Yes<br>◯ No<br>(The use of any verbs as "may" or "could", or any words as "likely to" or "maybe")
Is there any other misleading strategy reported in the discussion/conclusion section?	◯ Yes<br>◯ No
If yes, please provide the statement below	__________________________________

For peer review only

Other Information

Information about supplementary resources is provided	◯ Yes<br>◯ No
If yes, what type of supplementary material?	☐ Web calculator<br>☐ repository for dataset<br>☐ repository for R code<br>☐ extra results<br>☐ main results<br>☐ missing data<br>☐ predictors<br>☐ Protocol<br>☐ Details on statistics<br>☐ Other<br>(Multiples answers are possible. Protocol includes registered/published protocol as well.)
If other, please specify	____________________<br>(list using (;) to separate if more than 1)

Funding

The source of funding is reported or there is explicit mention that there was no external funding involved	◯ Yes<br>◯ No
If yes, which type of funding?	◯ Profit<br>◯ Non-Profit<br>◯ Both<br>◯ Unclear
The role of funders is reported	◯ Yes<br>◯ No<br>◯ NA<br>(If there is no external funding, please select "NA")
Is there a "Disclosure of authors' potential conflicts of interest (COI)" section in the journal?	◯ Yes<br>◯ No
If yes, are COI reported?	◯ Yes<br>◯ No
If yes, how many authors declared COI?	____________________<br>(Number only the ones who declare having conflicts)
Any additional comment about the discussion/conclusion section of this article?	____________________<br>(If there is something in the "Discussion/conclusion" section that does not fit into the questions of this form - please use this space to detail. Also use this space to detail anything you are unsure about.)

For peer review only

Title

Article ID
____

The words developing/development, validation/validating, incremental/added value (or synonyms) are reported in the title
◯ Yes
◯ No

Is the title supportive of the clinical relevance of the model, despite the study reporting non-significant/relevant measures?
◯ Yes
◯ No
(Title is inconsistent with the study results)

Is there use of leading words/strong statement in the title to describe model and/or model performance?
◯ Yes
◯ No
(E.g. Novel, excellent, acurrate, significant, promising, breakthrough, etc.)

If yes, please select the leading word(s)/strong statement(s) used
☐ Novel
☐ Excellent
☐ Accurate
☐ Optimal
☐ Perfect
☐ Significant
☐ Other
(Multiples answers are possible)

If other, please specify
____
(list using (;) to separate if more than 1)

The words prediction, risk prediction, prediction model, risk models, prognostic models, prognostic indices, risk scores (or synonyms) are reported in the title
◯ Yes
◯ No

The target population is reported in the title
◯ Yes
◯ No

The outcome to be predicted is reported in the title
◯ Yes
◯ No

Any additional comments about the title of this article?
____
(If there is something in the "Title" section that does not fit into the questions of this form - please use this space to detail. Also use this space to detail anything you are unsure about.)

For peer review only

Abstract

Article ID	____________________________
The objectives are reported in the abstract	◯ Yes<br>◯ No
Sources of data are reported in the abstract	◯ Yes<br>◯ No<br>(E.g. Prospective cohort, registry data, RCT data.)
The setting is reported in the abstract	◯ Yes<br>◯ No<br>(E.g. Primary care, secondary care, general population, adult care, or paediatric care. The setting should be reported for both the development and validation datasets, if applicable.)
A general definition of the study participants is reported in the abstract	◯ Yes<br>◯ No<br>(E.g. patients with suspicion of certain disease, patients with a specific disease, or general eligibility criteria.)
The overall sample size is reported in the abstract	◯ Yes<br>◯ No
The number of events (or % outcome together with overall sample size) is reported in the abstract	◯ Yes<br>◯ No<br>◯ NA<br>(If a continuous outcome was studied, score Not applicable)
Predictors included in the final model are reported in the abstract. For validation studies of well-known models, at least the name/acronym of the validated model is reported	◯ Yes<br>◯ No<br>(Broad descriptions are sufficient, e.g. 'all information from patient history and physical examination'. Check in the main text whether all predictors of the final model are indeed reported in the abstract.)
The outcome is reported in the abstract	◯ Yes<br>◯ No
Statistical methods are described in the abstract	◯ Yes<br>◯ No<br>(For model development, at least the type of statistical model should be reported. For validation studies a quote like "model's discrimination and calibration was assessed" is considered adequate. If done, methods of updating should be reported.)
ML techniques that will be used are reported in the abstract	◯ Yes<br>◯ No

For peer review only

For peer review only - http://bmjopen.bmj.com/site/about/guidelines.xhtml

Results for model discrimination are reported in the abstract	◯ Yes<br>◯ No<br>(This should be reported separately for development and validation if a study includes both development and validation.)
If yes, which measures are described in the abstract?	__________________________________<br>(list the names using (;) to separate if more than 1)
If yes, are confidence interval reported?	◯ Yes<br>◯ No
Results for model calibration are reported in the abstract	◯ Yes<br>◯ No<br>(This should be reported separately for development and validation if a study includes both development and validation.)
If yes, which measures are described in the abstract?	__________________________________<br>(list the names using (;) to separate if more than 1)
If yes, are precision estimates reported?	◯ Yes<br>◯ No
Is there any other type of measures reported?	◯ Yes<br>◯ No
If yes, please specify	__________________________________<br>(list the names using (;) to separate if more than 1)
Conclusions are reported in the abstract	◯ Yes<br>◯ No<br>(In publications addressing both model development and validation, there is no need for separate conclusions for both; one conclusion is sufficient.)
If yes, are the conclusion consistent with the reported results in the abstract section?	◯ Yes<br>◯ No
Does the conclusion statement focus solely on significant/relevant results?	◯ Yes<br>◯ No<br>(absence of non signicant results reports)
Is there emphasis on model relevance in the conclusion section of the abstract while there is not enough information given to concluded results are predictive?	◯ Yes<br>◯ No

For peer review only

Is there discrepancies between the full-text (discussion) and abstract (conclusion) explanations of the study findings?	◯ Yes<br>◯ No<br>(The discussion is consistent with the study findings, whereas the abstract conclusion is not [+/−]; OR The discussion is not consistent with the study findings, whereas the abstract conclusion is [−/+])
If yes, please copy the statement below	__________________________________
What is the recommended next step for the prediction model?	☐ To be used in clinical practice<br>☐ Validate the models in a different setting/population<br>☐ Other recommendations for further studies<br>☐ None reported<br>(Multiples answers are possible)
If other recommendations, please specify	__________________________________<br>(list using (;) to separate if more than 1)
Is there any reference to previous prediction model in literature in the abstract section?	◯ Yes<br>◯ No
If yes, please copy the statement below	__________________________________
Is there use of leading words/strong statement in the abstract section to describe model and/or model performance/accuracy/effectiveness?	◯ Yes<br>◯ No<br>(E.g. Novel, excellent, acurrate, significant, promising, breakthrough, etc.)
If yes, which leading words/strong statement?	☐ Novel<br>☐ Excellent<br>☐ Accurate<br>☐ Optimal<br>☐ Perfect<br>☐ Significant<br>☐ Other<br>(Multiples answers are possible)
If other, please specify	__________________________________<br>(list using (;) to separate if more than 1)
Please copy the statement below	__________________________________
Is uncertainty reported in the abstract?	◯ Yes<br>◯ No<br>(The use of any verbs as "may" or "could", nor any words as "likely to" or "maybe")
Limitations are reported in the abstract	◯ Yes<br>◯ No

For peer review only

Please copy the statement below

Any additional comment about the "abstract" section of this article?

(If there is something in the "Abstract" section that does not fit into the questions of this form - please use this space to detail. Also use this space to detail anything you are unsure about.)

For peer review only

Probast

Article ID

Instructions When assessing ROB using PROBAST, please refer to the "best performance" model for the primary outcome suggested by the authors

What type of study is reported?

◯ Development (including internal validation)
◯ Development with external validation (same model)
◯ Development with external validation (different model)
◯ External validation only
(This questions is repeated due to branching logic. Please answer again.)

Domain 1 : Participants

Development
Were appropriate data sources used, e.g. cohort, RCT or nested case-control study data?

◯ Yes / Probably yes
◯ No / Probably no
◯ No information

External validation
Were appropriate data sources used, e.g. cohort, RCT or nested case-control study data?

◯ Yes / Probably yes
◯ No / Probably no
◯ No information

Development
Were all inclusions and exclusions of participants appropriate?

◯ Yes / Probably yes
◯ No / Probably no
◯ No information
(Yes/probably yes: If inclusion and exclusion of participants was appropriate, so participants correspond to unselected participants of interest. No/probably no: If participants are included who would already have been identified as having the outcome and so are no longer participants at suspicion of disease (diagnostic studies) or at risk of developing outcome (prognostic studies), or if specific subgroups are excluded that may have altered the performance of the prediction model for the intended target population. No information: When there is no information on whether inappropriate inclusions or exclusions took place.)

For peer review only - http://bmjopen.bmj.com/site/about/guidelines.xhtml

External validation<br>Were all inclusions and exclusions of participants appropriate?	◯ Yes / Probably yes<br>◯ No / Probably no<br>◯ No information<br>(Yes/probably yes: If inclusion and exclusion of participants was appropriate, so participants correspond to unselected participants of interest. No/probably no: If participants are included who would already have been identified as having the outcome and so are no longer participants at suspicion of disease (diagnostic studies) or at risk of developing outcome (prognostic studies), or if specific subgroups are excluded that may have altered the performance of the prediction model for the intended target population. No information: When there is no information on whether inappropriate inclusions or exclusions took place.)
Development<br>Risk of bias introduced by Participants	◯ Low ROB<br>◯ High ROB<br>◯ Unclear ROB<br>(Low risk of bias: If the answer to all signaling questions is "Yes" or "Probably yes," then risk of bias can be considered low. If ≥1 of the answers is "No" or "Probably no," the judgment could still be "Low risk of bias" but specific reasons should be provided why the risk of bias can be considered low. High risk of bias: If the answer to any of the signaling questions is "No" or "Probably no," there is a potential for bias, except if defined at low risk of bias above. Unclear risk of bias: If relevant information is missing for some of the signaling questions and none of the signaling questions is judged to put this domain at high risk of bias.)
External validation<br>Risk of bias introduced by Participants	◯ Low ROB<br>◯ High ROB<br>◯ Unclear ROB<br>(Low risk of bias: If the answer to all signaling questions is "Yes" or "Probably yes," then risk of bias can be considered low. If ≥1 of the answers is "No" or "Probably no," the judgment could still be "Low risk of bias" but specific reasons should be provided why the risk of bias can be considered low. High risk of bias: If the answer to any of the signaling questions is "No" or "Probably no," there is a potential for bias, except if defined at low risk of bias above. Unclear risk of bias: If relevant information is missing for some of the signaling questions and none of the signaling questions is judged to put this domain at high risk of bias.)
Development<br>Support for Judgement	__________________________________
External validation<br>Support for Judgement	__________________________________

For peer review only

Domain 2 : Predictors

Development<br>Were predictors defined and assessed in a similar way for all participants?	◯ Yes / Probably yes<br>◯ No / Probably No<br>◯ No information<br>(Yes/probably yes: If definitions of predictors and their assessment were similar for all participants. No/probably no: If different definitions were used for the same predictor or if predictors requiring subjective interpretation were assessed by differently experienced assessors. No information: If there is no information on how predictors were defined or assessed.)
External validation<br>Were predictors defined and assessed in a similar way for all participants?	◯ Yes / Probably yes<br>◯ No / Probably No<br>◯ No information<br>(Yes/probably yes: If definitions of predictors and their assessment were similar for all participants. No/probably no: If different definitions were used for the same predictor or if predictors requiring subjective interpretation were assessed by differently experienced assessors. No information: If there is no information on how predictors were defined or assessed.)
Development<br>Were predictor assessments made without knowledge of outcome data?	◯ Yes / Probably yes<br>◯ No / Probably no<br>◯ No information
External validation<br>Were predictor assessments made without knowledge of outcome data?	◯ Yes / Probably yes<br>◯ No / Probably no<br>◯ No information
Development<br>Are all predictors available at the time the model is intended to be used?	◯ Yes / Probably Yes<br>◯ No / Probably No<br>◯ No information
External validation<br>Are all predictors available at the time the model is intended to be used?	◯ Yes / Probably Yes<br>◯ No / Probably No<br>◯ No information

For peer review only

Development<br>Risk of bias introduced by predictors	◯ Low ROB<br>◯ High ROB<br>◯ Unclear ROB<br>(Low risk of bias: If the answer to all signaling questions is "Yes" or "Probably Yes," then risk of bias can be considered low. If ≥1 of the answers is "No" or "Probably no," the judgment could still be "Low risk of bias" but specific reasons should be provided why the risk of bias can be considered low, e.g., use of objective predictors not requiring subjective interpretation. High risk of bias: If the answer to any of the signaling questions is "No" or "Probably no," there is a potential for bias. Unclear risk of bias: If relevant information is missing for some of the signaling questions and none of the signaling questions is judged to put the domain at high risk of bias.)
External validation<br>Risk of bias introduced by predictors	◯ Low ROB<br>◯ High ROB<br>◯ Unclear ROB<br>(Low risk of bias: If the answer to all signaling questions is "Yes" or "Probably Yes," then risk of bias can be considered low. If ≥1 of the answers is "No" or "Probably no," the judgment could still be "Low risk of bias" but specific reasons should be provided why the risk of bias can be considered low, e.g., use of objective predictors not requiring subjective interpretation. High risk of bias: If the answer to any of the signaling questions is "No" or "Probably no," there is a potential for bias. Unclear risk of bias: If relevant information is missing for some of the signaling questions and none of the signaling questions is judged to put the domain at high risk of bias.)
Development<br>Support for Judgement	__________________________________
External validation<br>Support for Judgement	__________________________________

Domain 3 : Outcome

Development<br>Was the outcome determined appropriately?	◯ Yes / Probably yes<br>◯ No / Probably no<br>◯ No information
External validation<br>Was the outcome determined appropriately?	◯ Yes / Probably yes<br>◯ No / Probably no<br>◯ No information
Development<br>Was a pre-specified or standard outcome definition used?	◯ Yes / Probably yes<br>◯ No / Probably no<br>◯ No information

For peer review only

External validation<br>Was a pre-specified or standard outcome definition used?	◯ Yes / Probably yes<br>◯ No / Probably no<br>◯ No information
Development<br>Were predictors excluded from the outcome definition?	◯ Yes / Probably yes<br>◯ No / Probably no<br>◯ No information
External validation<br>Were predictors excluded from the outcome definition?	◯ Yes / Probably yes<br>◯ No / Probably no<br>◯ No information
Development<br>Was the outcome defined and determined in a similar way for all participants?	◯ Yes / Probably yes<br>◯ No / Probably no<br>◯ No information
External validation<br>Was the outcome defined and determined in a similar way for all participants?	◯ Yes / Probably yes<br>◯ No / Probably no<br>◯ No information
Development<br>Was the outcome determined without knowledge of predictor information?	◯ Yes / Probably yes<br>◯ No / Probably no<br>◯ No information
External validation<br>Was the outcome determined without knowledge of predictor information?	◯ Yes / Probably yes<br>◯ No / Probably no<br>◯ No information
Development<br>Was the time interval between predictor assessment and outcome determination appropriate?	◯ Yes / Probably yes<br>◯ No / Probably no<br>◯ No information
External validation<br>Was the time interval between predictor assessment and outcome determination appropriate?	◯ Yes / Probably yes<br>◯ No / Probably no<br>◯ No information
Development<br>Risk of bias introduced by the outcome	◯ Low ROB<br>◯ High ROB<br>◯ Unclear ROB<br>(Low risk of bias: If the answer to all signaling questions is "Yes" or "Probably yes," then risk of bias can be considered low. If ≥1 of the answers is "No" or "Probably no," the judgment could still be low risk of bias, but specific reasons should be provided why the risk of bias can be considered low, e.g., when the outcome was determined with knowledge of predictor information but the outcome assessment did not require much interpretation by the assessor (e.g., death regardless of cause). High risk of bias: If the answer to any of the signaling questions is "No" or "Probably no," there is a potential for bias. Unclear risk of bias: If relevant information about the outcome is missing for some of the signaling questions and none of the signaling questions is judged to put this domain at high risk of bias.)

For peer review only

External validation<br>Risk of bias introduced by the outcome	◯ Low ROB<br>◯ High ROB<br>◯ Unclear ROB<br>(Low risk of bias: If the answer to all signaling questions is "Yes" or "Probably yes," then risk of bias can be considered low. If ≥1 of the answers is "No" or "Probably no," the judgment could still be low risk of bias, but specific reasons should be provided why the risk of bias can be considered low, e.g., when the outcome was determined with knowledge of predictor information but the outcome assessment did not require much interpretation by the assessor (e.g., death regardless of cause). High risk of bias: If the answer to any of the signaling questions is "No" or "Probably no," there is a potential for bias. Unclear risk of bias: If relevant information about the outcome is missing for some of the signaling questions and none of the signaling questions is judged to put this domain at high risk of bias.)
Development<br>Support for Judgement	__________________________________
External validation<br>Support for Judgement	__________________________________

Domain 4 : Analysis

Development<br>Were there a reasonable number of participants with the outcome?	◯ Yes / Probably yes<br>◯ No / Probably no<br>◯ No information<br>(Yes/probably yes: For model development studies, if the number of participants with the outcome relative to the number of candidate predictor parameters is ≥20 (EPV ≥20).* For model validation studies, if the number of participants with the outcome is ≥100. No/probably no: For model development studies, if the number of participants with the outcome relative to the number of candidate predictor parameters is < 10 (EPV < 10).* For model validation studies, if the number of participants with the outcome is < 100. No information: For model development studies, no information on the number of candidate predictor parameters or number of participants with the outcome, such that the EPV cannot be calculated. For model validation studies, no information on the number of participants with the outcome.)

For peer review only

External validation<br>Were there a reasonable number of participants with the outcome?	◯ Yes / Probably yes<br>◯ No / Probably no<br>◯ No information<br>(Yes/probably yes: For model development studies, if the number of participants with the outcome relative to the number of candidate predictor parameters is ≥20 (EPV ≥20).* For model validation studies, if the number of participants with the outcome is ≥100. No/probably no: For model development studies, if the number of participants with the outcome relative to the number of candidate predictor parameters is < 10 (EPV < 10).* For model validation studies, if the number of participants with the outcome is < 100. No information: For model development studies, no information on the number of candidate predictor parameters or number of participants with the outcome, such that the EPV cannot be calculated. For model validation studies, no information on the number of participants with the outcome.)
Development<br>Were continuous and categorical handled appropriately?	◯ Yes / Probably yes<br>◯ No / Probably no<br>◯ No information<br>(Yes/probably yes: If continuous predictors are not converted into ≥2 categories when included in the model (i.e., dichotomized or categorized), or if continuous predictors are examined for nonlinearity using, for example, fractional polynomials or restricted cubic splines, or if categorical predictor groups are defined using a prespecified method. For model validation studies, if continuous predictors are included using the same definitions or transformations, and categorical variables are categorized using the same cut points, as compared with the development study. No/probably no: If categorical predictor group definitions do not use a prespecified method. For model development studies, if continuous predictors are converted into ≥2 categories when included in the model. For model validation studies, if continuous predictors are included using different definitions or transformations, or categorical variables are categorized using different cut points, as compared with the development study. No information: No information on whether continuous predictors are examined for nonlinearity and no information on how categorical predictor groups are defined. For model validation studies, no information on whether the same definitions or transformations and the same cut points are used, as compared with the development study.)

For peer review only

External validation<br>Were continuous and categorical handled appropriately?	◯ Yes / Probably yes<br>◯ No / Probably no<br>◯ No information<br>(Yes/probably yes: If continuous predictors are not converted into ≥2 categories when included in the model (i.e., dichotomized or categorized), or if continuous predictors are examined for nonlinearity using, for example, fractional polynomials or restricted cubic splines, or if categorical predictor groups are defined using a prespecified method. For model validation studies, if continuous predictors are included using the same definitions or transformations, and categorical variables are categorized using the same cut points, as compared with the development study. No/probably no: If categorical predictor group definitions do not use a prespecified method. For model development studies, if continuous predictors are converted into ≥2 categories when included in the model. For model validation studies, if continuous predictors are included using different definitions or transformations, or categorical variables are categorized using different cut points, as compared with the development study. No information: No information on whether continuous predictors are examined for nonlinearity and no information on how categorical predictor groups are defined. For model validation studies, no information on whether the same definitions or transformations and the same cut points are used, as compared with the development study.)
Development<br>Were enrolled participants included in the analysis?	◯ Yes / Probably yes<br>◯ No / Probably no<br>◯ No information
External validation<br>Were enrolled participants included in the analysis?	◯ Yes / Probably yes<br>◯ No / Probably no<br>◯ No information
Development<br>Were participants with missing data handled appropriately?	◯ Yes / Probably yes<br>◯ No / Probably no<br>◯ No information
External validation<br>Were participants with missing data handled appropriately?	◯ Yes / Probably yes<br>◯ No / Probably no<br>◯ No information
Development<br>Was selection of predictors based on univariable analysis avoided?	◯ Yes / Probably yes<br>◯ No / Probably no<br>◯ No information
Development<br>Were complexities in the data (e.g., censoring, competing risks, sampling of control participants) accounted appropriately?	◯ Yes / Probably yes<br>◯ No / Probably no<br>◯ No information

For peer review only

External validation<br>Were complexities in the data (e.g., censoring, competing risks, sampling of control participants) accounted appropriately?	◯ Yes / Probably yes<br>◯ No / Probably no<br>◯ No information
Development<br>Were relevant model performance measures evaluated appropriately?	◯ Yes / Probably yes<br>◯ No / Probably no<br>◯ No information<br>(Yes/probably yes: If both calibration and discrimination are evaluated appropriately (including relevant measures tailored for models predicting survival outcomes). No/probably no: If both calibration and discrimination are not evaluated, or if only goodness-of-fit tests, such as the Hosmer-Lemeshow test, are used to evaluate calibration, or if for models predicting survival outcomes performance measures accounting for censoring are not used, or if classification measures (like sensitivity, specificity, or predictive values) were presented using predicted probability thresholds derived from the data set at hand. No information: Either calibration or discrimination are not reported, or no information is provided as to whether appropriate performance measures for survival outcomes are used (e.g., references to relevant literature or specific mention of methods, such as using Kaplan-Meier estimates), or no information on thresholds for estimating classification measures is given.)
External validation<br>Were relevant model performance measures evaluated appropriately?	◯ Yes / Probably yes<br>◯ No / Probably no<br>◯ No information<br>(Yes/probably yes: If both calibration and discrimination are evaluated appropriately (including relevant measures tailored for models predicting survival outcomes). No/probably no: If both calibration and discrimination are not evaluated, or if only goodness-of-fit tests, such as the Hosmer-Lemeshow test, are used to evaluate calibration, or if for models predicting survival outcomes performance measures accounting for censoring are not used, or if classification measures (like sensitivity, specificity, or predictive values) were presented using predicted probability thresholds derived from the data set at hand. No information: Either calibration or discrimination are not reported, or no information is provided as to whether appropriate performance measures for survival outcomes are used (e.g., references to relevant literature or specific mention of methods, such as using Kaplan-Meier estimates), or no information on thresholds for estimating classification measures is given.)
Development<br>Were model overfitting, under-fitting, and optimism in model performance accounted for?	◯ Yes / Probably yes<br>◯ No / Probably no<br>◯ No information

For peer review only

External validation<br>Were model overfitting, under-fitting, and optimism in model performance accounted for?	◯ Yes / Probably yes<br>◯ No / Probably no<br>◯ No information
Development<br>Do predictors and their assigned weights in the final model correspond to the results from the reported multivariable analysis	◯ Yes / Probably yes<br>◯ No / Probably no<br>◯ No information
External validation<br>Do predictors and their assigned weights in the final model correspond to the results from the reported multivariable analysis	◯ Yes / Probably yes<br>◯ No / Probably no<br>◯ No information
Development<br>Risk of bias introduced by the analysis	◯ Low ROB<br>◯ High ROB<br>◯ Unclear ROB<br>(Low risk of bias: If the answer to all signaling questions is "Yes" or "Probably yes," then risk of bias can be considered low. If ≥1 of the answers is "No" or "Probably no," the judgment could still be low risk of bias, but specific reasons should be provided why the risk of bias can be considered low. High risk of bias: If the answer to any of the signaling questions is "No" or "Probably no," there is a potential for bias. Unclear risk of bias: If relevant information about the analysis is missing for some of the signaling questions but none of the signaling question answers is judged to put the analysis at high risk of bias)
External validation<br>Risk of bias introduced by the analysis	◯ Low ROB<br>◯ High ROB<br>◯ Unclear ROB<br>(Low risk of bias: If the answer to all signaling questions is "Yes" or "Probably yes," then risk of bias can be considered low. If ≥1 of the answers is "No" or "Probably no," the judgment could still be low risk of bias, but specific reasons should be provided why the risk of bias can be considered low. High risk of bias: If the answer to any of the signaling questions is "No" or "Probably no," there is a potential for bias. Unclear risk of bias: If relevant information about the analysis is missing for some of the signaling questions but none of the signaling question answers is judged to put the analysis at high risk of bias)
Development<br>Support for Judgement	__________________________________
External validation<br>Support for Judgement	__________________________________

For peer review only

Overall assessment of ROB

Development<br>Overall risk of bias	◯ Low risk of bias<br>◯ High risk of bias<br>◯ Unclear risk of bias<br>(Low ROB: If all domains were rated low risk of bias. If a prediction model was developed without any external validation, and it was rated as low risk of bias for all domains, consider downgrading to high risk of bias. Such a model evaluation can only be considered as low risk of bias, if the development was based on a very large data set and included some form of internal validation. High ROB: If ≥1 domain is judged to be at high risk of bias. Unclear ROB: If an unclear risk of bias was noted in ≥1 domain and it was low risk for all other domains.)
External validation<br>Overall risk of bias	◯ Low risk of bias<br>◯ High risk of bias<br>◯ Unclear risk of bias<br>(Low ROB: If all domains were rated low risk of bias. If a prediction model was developed without any external validation, and it was rated as low risk of bias for all domains, consider downgrading to high risk of bias. Such a model evaluation can only be considered as low risk of bias, if the development was based on a very large data set and included some form of internal validation. High ROB: If ≥1 domain is judged to be at high risk of bias. Unclear ROB: If an unclear risk of bias was noted in ≥1 domain and it was low risk for all other domains.)
Any additional comments about ROB on this article?	__________________________________<br>(If there is something in the "PROBAST" that does not fit into the questions of this form - please use this space to detail. Also use this space to detail anything you are unsure about.)

For peer review only

Reporting checklist for protocol of a systematic review.

Based on the PRISMA-P guidelines.

Instructions to authors

Complete this checklist by entering the page numbers from your manuscript where readers will find each of the items listed below.

Your article may not currently address all the items on the checklist. Please modify your text to include the missing information. If you are certain that an item does not apply, please write "n/a" and provide a short explanation.

Upload your completed checklist as an extra file when you submit to a journal.

In your methods section, say that you used the PRISMA-Preporting guidelines, and cite them as:

Moher D, Shamseer L, Clarke M, Ghersi D, Liberati A, Petticrew M, Shekelle P, Stewart LA. Preferred Reporting Items for Systematic Review and Meta-Analysis Protocols (PRISMA-P) 2015 statement. Syst Rev. 2015;4(1):1.

		Reporting Item	Page Number
**Title**			
Identification	#1a	Identify the report as a protocol of a systematic review	1
Update	#1b	If the protocol is for an update of a previous systematic review, identify as such	NA
**Registration**			
	#2	If registered, provide the name of the registry (such as PROSPERO) and registration number	2
**Authors**			
Contact	#3a	Provide name, institutional affiliation, e-mail address of all protocol authors; provide physical mailing address of corresponding author	1
Contribution	#3b	Describe contributions of protocol authors and identify the	11-12

For peer review only

		guarantor of the review	
**Amendments**			
	#4	If the protocol represents an amendment of a previously completed or published protocol, identify as such and list changes; otherwise, state plan for documenting important protocol amendments	10
**Support**			
Sources	#5a	Indicate sources of financial or other support for the review	12
Sponsor	#5b	Provide name for the review funder and / or sponsor	12
Role of sponsor or funder	#5c	Describe roles of funder(s), sponsor(s), and / or institution(s), if any, in developing the protocol	12
**Introduction**			
Rationale	#6	Describe the rationale for the review in the context of what is already known	4-5
Objectives	#7	Provide an explicit statement of the question(s) the review will address with reference to participants, interventions, comparators, and outcomes (PICO)	5
**Methods**			
Eligibility criteria	#8	Specify the study characteristics (such as PICO, study design, setting, time frame) and report characteristics (such as years considered, language, publication status) to be used as criteria for eligibility for the review	6-7
Information sources	#9	Describe all intended information sources (such as electronic databases, contact with study authors, trial registers or other grey literature sources) with planned dates of coverage	7
Search strategy	#10	Present draft of search strategy to be used for at least one electronic database, including planned limits, such that it could be repeated	7-8
Study records - data management	#11a	Describe the mechanism(s) that will be used to manage records and data throughout the review	8
Study records -	#11b	State the process that will be used for selecting studies (such	8-9

For peer review only

selection process		as two independent reviewers) through each phase of the review (that is, screening, eligibility and inclusion in meta-analysis)	
Study records - data collection process	#11c	Describe planned method of extracting data from reports (such as piloting forms, done independently, in duplicate), any processes for obtaining and confirming data from investigators	8-9
Data items	#12	List and define all variables for which data will be sought (such as PICO items, funding sources), any pre-planned data assumptions and simplifications	9
Outcomes and prioritization	#13	List and define all outcomes for which data will be sought, including prioritization of main and additional outcomes, with rationale	9
Risk of bias in individual studies	#14	Describe anticipated methods for assessing risk of bias of individual studies, including whether this will be done at the outcome or study level, or both; state how this information will be used in data synthesis	9
Data synthesis	#15a	Describe criteria under which study data will be quantitatively synthesised	9-10
Data synthesis	#15b	If data are appropriate for quantitative synthesis, describe planned summary measures, methods of handling data and methods of combining data from studies, including any planned exploration of consistency (such as I2, Kendall's τ)	9-10
Data synthesis	#15c	Describe any proposed additional analyses (such as sensitivity or subgroup analyses, meta-regression)	10
Data synthesis	#15d	If quantitative synthesis is not appropriate, describe the type of summary planned	9-10
Meta-bias(es)	#16	Specify any planned assessment of meta-bias(es) (such as publication bias across studies, selective reporting within studies)	10
Confidence in cumulative evidence	#17	Describe how the strength of the body of evidence will be assessed (such as GRADE)	10

For peer review only

The PRISMA-P checklist is distributed under the terms of the Creative Commons Attribution License CC-BY 4.0. This checklist was completed on 06. March 2020 using https://www.goodreports.org/, a tool made by the EQUATOR Network in collaboration with Penelope.ai

For peer review only
